# Large-scale genome-wide enrichment analyses identify new trait-associated genes and pathways across 31 human phenotypes

**DOI:** 10.1038/s41467-018-06805-x

**Published:** 2018-10-19

**Authors:** Xiang Zhu, Matthew Stephens

**Affiliations:** 1 Department of Statistics, Stanford University, Stanford, 94305 CA USA; 2 Department of Statistics, The University of Chicago, Chicago, 60637 IL USA; 3 Department of Human Genetics, The University of Chicago, Chicago, 60637 IL USA

## Abstract

Genome-wide association studies (GWAS) aim to identify genetic factors associated with phenotypes. Standard analyses test variants for associations individually. However, variant-level associations are hard to identify and can be difficult to interpret biologically. Enrichment analyses help address both problems by targeting sets of biologically related variants. Here we introduce a new model-based enrichment method that requires only GWAS summary statistics. Applying this method to interrogate 4,026 gene sets in 31 human phenotypes identifies many previously-unreported enrichments, including enrichments of endochondral ossification pathway for height, NFAT-dependent transcription pathway for rheumatoid arthritis, brain-related genes for coronary artery disease, and liver-related genes for Alzheimer’s disease. A key feature of our method is that inferred enrichments automatically help identify new trait-associated genes. For example, accounting for enrichment in lipid transport genes highlights association between *MTTP* and low-density lipoprotein levels, whereas conventional analyses of the same data found no significant variants near this gene.

## Abstract (web summary)

In genome-wide association studies, variant-level associations are hard to identify and can be difficult to interpret biologically. Here, the authors develop a new model-based enrichment analysis method, and apply it to identify new associated genes, pathways and tissues across 31 human phenotypes.

## Introduction

Genome-wide association studies (GWAS) have successfully identified many genetic variants—typically single-nucleotide polymorphisms (SNPs)—underlying a wide range of complex traits^1,2^. GWAS are typically analyzed using single-SNP association tests, which assess the marginal correlation between the genotypes of each SNP and the trait of interest. This approach can work well for identifying common variants with sufficiently-large effects. However, for complex traits, most variants have small effects, making them difficult to identify even with large sample sizes^3^. Further, because many associated variants are noncoding it can be difficult to identify the biological mechanisms by which they may act.

Enrichment analysis—also referred to as pathway^4^ or gene set^5^ analysis—can help tackle both these problems. Instead of analyzing one variant at a time, enrichment analysis assesses groups of related variants. The idea—borrowed from enrichment analysis of gene expression^6^—is to identify groups of biologically related variants that are enriched for associations with the trait: that is, they contain a higher fraction of associated variants than would be expected by chance. By pooling information across many genetic variants this approach has the potential to detect enrichments even when individual genetic variants fail to reach a stringent significance threshold^4^. And because the sets of variants to be analyzed are often defined based on existing biological knowledge, an observed enrichment automatically suggests potentially relevant biological processes or mechanisms.

Although the idea of enrichment analysis is simple, there are many ways to implement it in practice, each with its own advantages and disadvantages. Here we build on a previous approach^7^ that has several attractive features not shared by most methods. These features include: it accounts for linkage disequilibrium (LD) among associated SNPs; it assesses SNP sets for enrichment directly, without requiring intermediate steps like imposing a significance cut-off or assigning SNP-level associations to specific genes; and it can reassess (“prioritize”) variant-level associations in light of inferred enrichments to identify which genetic factors are driving the enrichment.

Despite these advantages, this approach has a major limitation: it requires individual-level genotypes and phenotypes, which are often difficult or impossible to obtain, especially for large GWAS meta analyses combining many studies. Our major contribution here is to overcome this limitation, and provide a new method that requires only GWAS summary statistics (plus LD estimates from a suitable reference panel). This allows the method to be applied on a scale that would be otherwise impractical. Here, we exploit this to perform enrichment analyses of 3913 biological pathways and 113 tissue-based gene sets for 31 human phenotypes. Our results identify many novel pathways and tissues relevant to these phenotypes, as well as some that have been previously identified. By prioritizing variants within the enriched pathways we identify several trait-associated genes that do not reach genome-wide significance in conventional analyses of the same data. The results highlighted here demonstrate the potential for these enrichment analyses to yield novel insights from existing GWAS summary data.

## Results

### Method overview

**Fig. 1:**
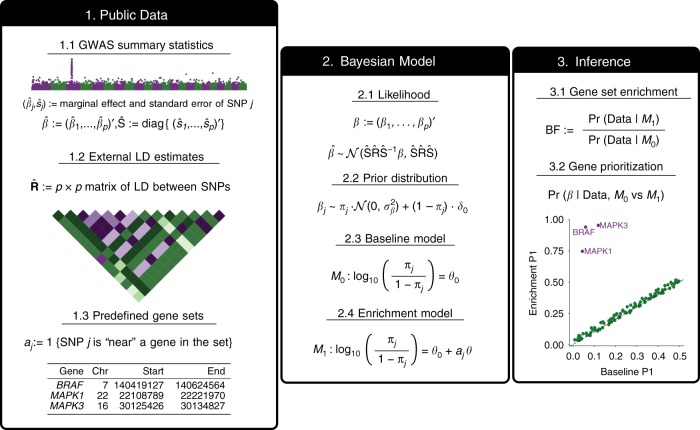
Schematic overview of RSS-E, a model-based enrichment analysis method for GWAS summary statistics. RSS-E combines three types of public data: GWAS summary statistics (1.1), external LD estimates (1.2), and predefined SNP sets (1.3). GWAS summary statistics consist of a univariate effect size estimate ($\hat \beta _j$) and corresponding standard error ($\hat s_j$) for each SNP, which are routinely generated in GWAS. External LD estimates are obtained from an external reference panel with ancestry matching the population of GWAS cohorts. SNP sets here are derive from gene sets based on biological pathways or sequencing data. We combine these three types of data by fitting a Bayesian multiple regression (2.1–2.2) under two models about the enrichment parameter (*θ*): the baseline model (2.3) that each SNP has equal chance of being associated with the trait (*M*_0_: *θ* = 0), and the enrichment model (2.4) that SNPs in the SNP set are more often associated with the trait (*M*_1_: *θ* > 0). To test enrichment, RSS-E computes a Bayes factor (BF) comparing these two models (3.1). RSS-E also automatically prioritizes loci within an enriched set by comparing the posterior distributions of genetic effects (***β***) under *M*_0_ and *M*_1_ (3.2). Here we summarize the posterior of ***β*** as *P*_1_, the posterior probability that at least one SNP in a locus is trait-associated. Differences between *P*_1_ estimated under *M*_0_ and *M*_1_ reflect the influence of enrichment on genetic associations, which can help identify new trait-associated genes (3.2)

Figure 1 provides a schematic method overview. In brief, we combine an enrichment model^7^ with regression with summary statistics (RSS)^8^, a multiple regression likelihood for single-SNP association summary statistics, to create a model-based enrichment method for GWAS summary data. We refer to this enrichment method as RSS-E.

Specifically RSS-E requires single-SNP effect estimates and their standard errors from GWAS, and LD estimates from an external reference panel with similar ancestry to the GWAS cohort. Then, for any given set of SNPs, RSS-E estimates an enrichment parameter, *θ*, which measures the extent to which SNPs in the set are more often associated with the phenotype. This enrichment parameter is on a log10 scale, so *θ* = 2 means that the rate at which associations occur inside the set is ~100 times higher than the rate of associations outside the set, whereas *θ* = 0 means that these rates are the same. When estimating *θ* RSS-E uses a multiple regression model^8^ to account for LD among SNPs. For example, RSS-E will (correctly) treat data from several SNPs that are in perfect LD as effectively a single observation, and not multiple independent observations. RSS-E ultimately summarizes the evidence for enrichment by a Bayes factor (BF) comparing the enrichment model (*M*_1_: *θ* > 0) against the baseline model (*M*_0_: *θ* = 0). RSS-E also provides posterior distributions of genetic effects (***β***) under *M*_0_ and *M*_1_, and uses them to prioritize variants within enriched sets.

Although enrichment analysis could be applied to any SNP set, here we focus on SNP sets derived from gene sets such as biological pathways. Specifically, for a given gene set, we define a corresponding SNP set as the set of SNPs within 100 kb of the transcribed region of any member gene; we refer to such SNPs as “inside” the gene set. If a gene set plays an important role in a trait then genetic associations may tend to occur more often near these genes than expected by chance; our method is designed to detect this signal.

To facilitate large-scale analyses, we designed an efficient, parallel algorithm implementing RSS-E. Our algorithm exploits variational inference^9^, banded matrix approximation^10^ and an expectation-maximization accelerator^11^. Software is available at https://github.com/stephenslab/rss.

### Method comparison based on simulations

The novelty of RSS-E lies in its use of whole-genome association summary statistics to infer enrichments, and more importantly, its automatic prioritization of genes in light of inferred enrichments. We are not aware of any published method with similar features. However, there are methods that can learn either enrichments or gene-level associations from GWAS summary statistics, but not both. We compare RSS-E to them through simulations using real genotypes^12^.

**Fig. 2:**
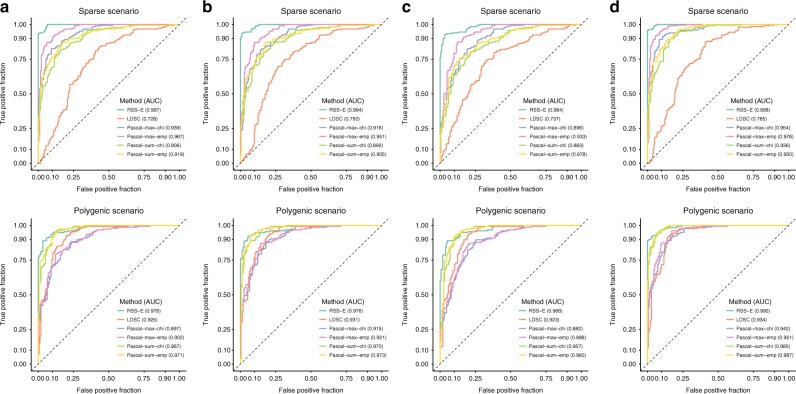
Comparison of RSS-E to other methods for identifying enrichments from GWAS summary statistics. We used real genotypes^12^ to simulate individual-level data under two genetic architectures (“sparse” and “polygenic”) with four baseline-enrichment patterns: **a** baseline and enrichment datasets followed baseline (*M*_0_) and enrichment (*M*_1_) models in RSS-E; **b** baseline datasets assumed that a random set of near-gene SNPs were enriched for genetic associations and enrichment datasets followed *M*_1_; **c** baseline datasets assumed that a random set of coding SNPs were enriched for genetic associations and enrichment datasets followed *M*_1_; **d** baseline datasets followed *M*_0_ and enrichment datasets assumed that trait-associated SNPs were both more frequent, and had larger effects, inside than outside the target gene set. We computed the corresponding single-SNP summary statistics, and, on these summary data, we compared RSS-E with Pascal^13^ and LDSC^14^ using their default setups. Pascal includes two gene scoring options: maximum-of-*χ*^2^ (-max) and sum-of-*χ*^2^ (-sum), and two pathway scoring options: *χ*^2^ approximation (-chi) and empirical sampling (-emp). For each simulated dataset, both Pascal and LDSC produced enrichment *p* values, whereas RSS-E produced an enrichment BF; these statistics were used to rank the significance of enrichments. Each panel displays the trade-off between false and true enrichment discoveries for all methods in 200 baseline and 200 enrichment datasets of a given simulation scenario, and also reports the corresponding areas under the curve (AUCs), where a higher value indicates better performance. Simulation details and additional results are provided in Supplementary Figures 1–4

To benchmark its enrichment component, we compared RSS-E with a suite of conventional pathway methods, Pascal^13^, and a polygenic approach, LD score regression (LDSC)^14^. We started with simulations without model mis-specification, where baseline and enrichment datasets were generated from corresponding models (*M*_0_ and *M*_1_). Figure 2a and Supplementary Figure 1 show the trade-off between false and true enrichment discoveries for each method. All methods are powerful when the true underlying genetic architecture is polygenic, whereas LDSC performs worse when the truth is sparse. In both polygenic and sparse scenarios RSS-E is the most powerful method.

Next, to assess its robustness to mis-specification, we performed three sets of simulations where either the baseline (*M*_0_) or enrichment (*M*_1_) model of RSS-E were mis-specified. Specifically, we considered scenarios where (i) baseline data contained enrichments of random near-gene SNPs (Fig. 2b, Supplementary Fig. 2); (ii) baseline data contained enrichments of random coding SNPs (Fig. 2c, Supplementary Fig. 3); and (iii) enrichment data contained enrichments of effect sizes (Fig. 2d, Supplementary Fig. 4). The results show that RSS-E is highly robust to model mis-specification, and still consistently outperforms Pascal and LDSC.

Recent analyses using LDSC focus on genotype–phenotype associations of HapMap Project Phase 3 (HapMap3) SNPs^15^, even though summary statistics of 1000 Genomes Project SNPs^16^ are often available. We used this “SNP subsetting” strategy in data analyses to reduce computation, since computational costs of RSS-E decrease as the number of SNPs analyzed decreases (Methods). However, when subsetting GWAS summary statistics to HapMap3 SNPs, RSS-E also subsets LD estimates to HapMap3 SNPs (Fig. 1), whereas LDSC still uses LD estimates of 1000 Genomes SNPs. To assess the impact of “SNP subsetting” on RSS-E, we simulated data using all 1000 Genome SNPs, applied the enrichment methods to summary statistics of HapMap3 SNPs only, and then compared HapMap3-based results with results of analyzing all 1000 Genome SNPs. As above, RSS-E is robust to “SNP subsetting” and more powerful than other methods (Supplementary Fig. 5).

**Fig. 3:**
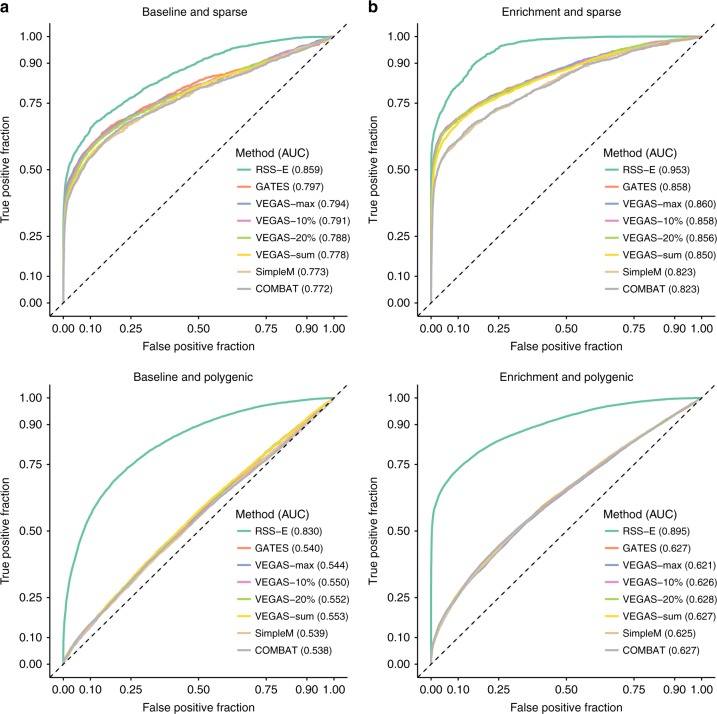
Comparison of RSS-E to other methods for identifying gene-level associations from GWAS summary statistics. We used real genotypes^12^ to simulate individual-level data with and without enrichment in the target gene set (**a** “baseline”; **b** “enrichment”), each under two genetic architectures (“sparse” and “polygenic”), and then computed corresponding single-SNP summary statistics. On these summary data, we compared RSS-E with four other methods: SimpleM^17^, VEGAS^18^, GATES^19^, and COMBAT^20^. We applied VEGAS to the full set of SNPs (-sum), to a specified percentage of the most significant SNPs (−10% and −20%), and to the single most significant SNP (-max), within 100 kb of the transcribed region of each gene. All methods are available in the package COMBAT (Methods). For each simulated dataset, we defined a gene as “trait-associated” if at least one SNP within 100 kb of the transcribed region of this gene had nonzero effect. For each gene in each dataset, RSS-E produced the posterior probability that the gene was trait-associated. whereas the other methods produced association *p* values; these statistics were used to rank the significance of gene-level associations. Each panel displays the trade-off between false and true gene-level associations for all methods in 100 datasets of a given simulation scenario, and reports the corresponding AUCs. Simulation details and additional results are provided in Supplementary Figures 6, 7

Finally, to benchmark its prioritization component, we compared RSS-E with four gene-based association methods^17–20^. Figure 3 and Supplementary Figures 6, 7 show the power of each method to identify gene-level associations. RSS-E substantially outperforms existing methods even in the absence of enrichments (Fig. 3a), especially in the polygenic scenario. This is because RSS-E exploits a multiple regression framework^8^ to learn the genetic architecture from data of all genes and assesses their effects jointly, whereas other methods implicitly assume a fixed, sparse architecture and only use data of a single gene to estimate its effect. When datasets contain enrichments (Fig. 3b), RSS-E further leverages them, which existing methods ignore, to improve power.

In conclusion, RSS-E outperforms existing methods in both enrichment and prioritization analysis, and is robust to a wide range of model mis-specification. To further investigate its real-world benefit, we applied RSS-E to analyze 31 complex traits and 4026 gene sets.

### Multiple regression on 1.1 million variants across 31 traits

**Fig. 4:**
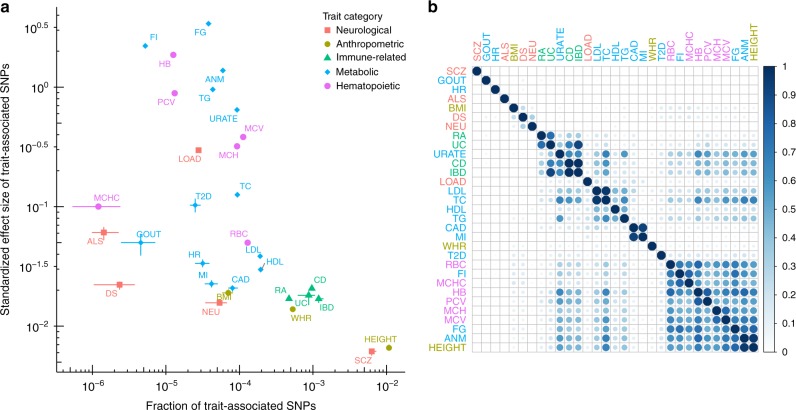
Baseline and enrichment analyses of GWAS summary statistics for 31 complex traits. References of these data are provided in Supplementary Notes. **a** Summary of inferred effect size distributions of 31 traits. Results are from fitting the baseline model (*M*_0_) to GWAS summary statistics of 1.1 million common HapMap3 SNPs for each trait using variational inference (Methods). We summarize effect size distribution using two statistics: the estimated fraction of trait-associated SNPs (average posterior probability of a SNP being trait-associated; *x*-axis) and the standardized effect size of trait-associated SNPs (average posterior mean effect size of all SNPs, normalized by phenotypic standard deviation and fraction of trait-associated SNPs; *y*-axis). Each dot represents a trait, with horizontal and vertical point ranges indicating posterior mean and 95% credible interval. See Supplementary Notes for more details. Note that some intervals are too small to be visible due to log10 scales. See Supplementary Table 2 for numerical values of all intervals. **b** Pairwise sharing of 3913 pathway enrichments among 31 traits. For each pair of traits, we estimate the proportion of pathways that are enriched in both traits, among pathways enriched in at least one of the traits (Methods). Traits are clustered by hierarchical clustering as implemented in the package corrplot (Methods). Darker color and larger shape represent higher sharing. The sharing estimates are provided in Supplementary Table 3. ALS amyotrophic lateral sclerosis; DS depressive symptoms; LOAD late-onset Alzheimer’s disease; NEU neuroticism; SCZ schizophrenia; BMI body mass index; HEIGHT adult height; WHR waist-to-hip ratio; CD Crohn’s disease; IBD inflammatory bowel disease; RA rheumatoid arthritis; UC ulcerative colitis; ANM age at natural menopause; CAD coronary artery disease; FG fasting glucose; FI fasting insulin; GOUT gout; HDL high-density lipoprotein; HR heart rate; LDL low-density lipoprotein; MI myocardial infarction; T2D type 2 diabetes; TC total cholesterol; TG triglycerides; URATE serum urate; HB hemoglobin; MCH mean cell HB; MCHC MCH concentration; MCV mean cell volume; PCV packed cell volume; RBC red blood cell count

The first step of our analysis is multiple regression of 1.1 million HapMap3 common SNPs for 31 traits, using GWAS summary statistics from 20,883 to 253,288 European ancestry individuals (Supplementary Table 1; Supplementary Fig. 8). This step essentially estimates, for each trait, a baseline model (*M*_0_) against which enrichment models (*M*_1_) can be compared. The fitted baseline model captures both the size and abundance (“polygenicity”) of the genetic effects on each trait, effectively providing a two-dimensional summary of the genetic architecture of each trait (Fig. 4a; Supplementary Fig. 9; Supplementary Table 2).

The results emphasize that genetic architecture varies considerably among phenotypes: estimates of both polygenicity and effect sizes vary by several orders of magnitude. Height and schizophrenia stand out as being particularly polygenic, showing approximately ten times as many estimated associated variants as any other phenotype. Along the other axis, fasting glucose, fasting insulin and hemoglobin show the highest estimates of effect sizes, with correspondingly lower estimates for the number of associated variants. Although not our main focus, these results highlight the potential for multiple regression models like ours to learn about effect size distributions and genetic architectures from GWAS summary statistics.

Fitting the baseline model also yields an estimate of effect size for each SNP. These can be used to identify trait-associated SNPs and loci. Reassuringly, these multiple-SNP results recapitulate many associations detected in single-SNP analyses of the same data (Supplementary Figs. 10–12). For several traits, these results also identify additional associations (Supplementary Figs. 13, 14). These additional findings, while potentially interesting, may be difficult to validate and interpret. Enrichment analysis can help here: if the additional signals tend to be enriched in a plausible pathway, it may both increase confidence in the statistical results and provide some biological framework to interpret them.

### Enrichment analyses of 3913 pathways across 31 traits

We next performed enrichment analyses of SNP sets derived from 3913 expert-curated pathways, ranging in size from 2 to 500 genes (Supplementary Figs. 15, 16). For each trait-pathway pair we computed a BF testing the enrichment model (*M*_1_), and estimated the enrichment parameter (*θ*).

Since these analyses involve large-scale computations that are subject to approximation error, we developed some sanity checks for confirming enrichments identified by RSS-E. Specifically these simple methods confirm that the *z*-scores for SNPs inside a putatively enriched pathway have a different distribution from those outside the pathway (with more *z*-scores away from 0)—using both a visual check and a likelihood ratio statistic (Supplementary Fig. 17). Of note, these methods cannot replace RSS-E in the present study. The visual check requires human input, and thus is not suitable for large-scale analyses like ours. The likelihood ratio does not account for LD, and is expected to be less powerful (Supplementary Fig. 18).

Since genic regions may be generally enriched for associations compared with nongenic regions, we confirmed that top-ranked pathways often showed stronger evidence for enrichment than did the set containing all genes (Supplementary Fig. 19). We also created “null” (nonenriched) SNP sets by randomly drawing near-gene SNPs, and performed enrichment analyses of these “null” sets on real GWAS summary data. Enrichment signals of these simulated genic sets are substantially weaker than the actual top-ranked sets (Supplementary Fig. 20). Further, to check whether observed enrichments could be driven by other functional annotations (e.g., coding), we computed the correlation between enrichment BFs and proportions of gene-set SNPs falling into each of 52 functional categories^14^. Among 1612 trait-category pairs, we did not observe any strong correlation (median 7.3 × 10^−3^; 95% interval [−0.08 to 0.21]; Supplementary Fig. 21). Together, these results suggest that observed enrichments are unlikely to be artifacts driven by model mis-specification.

For most traits our analyses identify many pathways with strong evidence for enrichment—for example, 20 traits have enrichment BFs ≥ 10^8^ in more than 100 pathways per trait (Supplementary Fig. 22). Although the top enriched pathways for a given trait often substantially overlap (i.e., share many genes), several traits show enrichments with multiple nonoverlapping or minimally overlapping pathways (Supplementary Fig. 23). Table 1 gives examples of top enriched pathways, with full results available online (Methods).

**Table 1:** Top enriched biological pathways in complex traits

Phenotype	Top enriched pathway	Database	# of signals (genes)	log_10_BF
*Neurological traits*				
Depressive symptoms	Eicosapentaenoate biosynthesis	HumanCyc (PC)	2 (12)	36.9
Alzheimer’s disease	Golgi associated vesicle biogenesis	Reactome (PC)	3 (49)	83.7
*Anthropometric traits*				
Adult height	Endochondral ossification	WikiPathways (BS)	57 (65)	68.9
*Immune-related traits*				
Crohn’s disease	Inflammatory bowel disease	KEGG (BS)	24 (61)	25.6
Inflammatory bowel disease	Inflammatory bowel disease	KEGG (BS)	26 (61)	24.2
Rheumatoid arthritis	NFAT-dependent transcription^a^	PID (BS)	11 (45)	10.0
Ulcerative colitis	Inflammatory bowel disease	KEGG (BS)	16 (61)	11.8
*Metabolic traits*				
Age at natural menopause	IL-2R*β* in T-cell activation	BioCarta	2 (37)	866.7
Coronary artery disease	p75(NTR)-mediated signaling	PID (BS)	4 (55)	16.0
Fasting glucose	Hexose transport	Reactome (BS)	4 (47)	1,898.4
Gout	Osteoblast signaling	WikiPathways (BS)	2 (13)	30.6
High-density lipoprotein	Statin pathway	WikiPathways (BS)	18 (30)	113.9
Low-density lipoprotein	Chylomicron-mediated lipid transport	Reactome (PC)	11 (17)	65.5
Myocardial infarction	Glutathione synthesis and recycling	Reactome (PC)	2 (11)	9.6
Total cholesterol	Glucose transport	Reactome (BS)	2 (41)	833.2
Triglycerides	Targets of C-MYC activation^b^	PID (BS)	3 (79)	604.9
Serum urate	Transport of glucose and others^c^	Reactome (PC)	4 (95)	1,558.1
*Hematopoietic traits*				
Hemoglobin (HB)	RNA polymerase I transcription	Reactome (BS)	27 (107)	2,641.3
Mean cell HB (MCH)	Meiotic synapsis	Reactome (PC)	21 (72)	2,334.3
MCH concentration	SIRT1 negative regulation of rRNA^d^	Reactome (PC)	3 (63)	700.8
Mean cell volume	DNA methylation	Reactome (PC)	28 (61)	2,077.3
Packed cell volume	RNA polymerase I promoter opening	Reactome (PC)	27 (59)	217.5
Red blood cell count	GSL biosynthesis (neolacto series)	KEGG (PC)	2 (21)	391.2

For each trait here we report the most enriched pathway (if any) that (i) has an enrichment Bayes factor (BF) greater than 10^8^; (ii) has at least 10 and at most 200 member genes; (iii) has at least two member genes with enrichment *P*_1_ > 0.9 (denoted as “signals”); and (iv) passes the visual and likelihood ratio sanity checks (Supplementary Fig. 17). All BFs reported here are larger than corresponding BFs that SNPs within 100 kb of transcribed regions of all genes are enriched (Supplementary Fig. 19). The corresponding baseline and enrichment parameter estimates are provided in online results (Methods). *P*_*1*_ posterior probability that at least one SNP within 100 kb of the transcribed region of a given gene has nonzero effect on the target trait; CaN: calcineurin; NFAT: nuclear factor of activated T cells; IL-2Rβ: interleukin-2 receptor beta chain; p75(NTR): p75 neurotrophin receptor; SIRT1: Sirtuin 1; GSL: glycosphingolipid; PC: Pathway Commons^63^; BS: NCBI BioSystems^64^

^a^ The full name of this pathway is “calcineurin-regulated NFAT-dependent transcription in lymphocytes”

^b^ The full name of this pathway is “validated targets of C-MYC transcriptional activation”

^c^ The full name of this pathway is “transport of glucose and other sugars, bile salts and organic acids, metal ions and amine compounds”

^d^ The full name of this pathway is “SIRT1 negatively regulates ribosomal RNA expression”

Our results highlight many previously reported trait-pathway links. For example, Hedgehog pathway is enriched for associations with adult height (BF = 1.9 × 10^40^), consistent with both pathway function^21^ and previous GWAS^22^. Other examples include interleukin-23 mediated signaling pathway with inflammatory bowel disease (BF = 3.1 × 10^23^; ref. ^23^), T helper cell surface molecule pathway with rheumatoid arthritis (BF = 3.2 × 10^8^; ref. ^24^), statin pathway with levels of high-density lipoprotein cholesterol (BF = 8.4 × 10^113^; ref. ^25^), and glucose transporter pathway with serum urate (BF = 1.2 × 10^1558^; ref. ^26^).

The results also highlight several pathway enrichments that were not reported in corresponding GWAS publications. For example, the top pathway for rheumatoid arthritis is calcineurin-regulated nuclear factor of activated T cells (NFAT)-dependent transcription in lymphocytes (BF = 1.1 × 10^10^). This result adds to the considerable existing evidence linking NFAT-regulated transcription to immune function^27^ and bone pathology^28^. Other examples of novel pathway enrichments include endochondral ossification pathway with adult height (BF = 7.7 × 10^68^; ref. ^29^), p75 neurotrophin receptor-mediated signaling pathway with coronary artery disease (BF = 9.6 × 10^15^; ref. ^30^), and osteoblast signaling pathway with gout (BF = 3.8 × 10^30^; ref. ^31^).

### Overlapping pathway enrichments among related traits

Some pathways show enrichment in multiple traits. To gain a global picture of shared pathway enrichments among traits we estimated the proportions of shared pathway enrichments for all pairs of traits (Fig. 4b; Supplementary Table 3). Clustering these pairwise sharing results highlights four main clusters of traits: immune-related diseases, blood lipids, heart disorders, and red blood cell phenotypes. Blood cholesterol shows strong pairwise sharing with serum urate (0.67), hemoglobin (0.66), and fasting glucose (0.53), which could be interpreted as a set of blood elements. Serum urate shows moderate to strong sharing with rheumatoid arthritis (0.19) and inflammatory bowel diseases (0.38–0.63), possibly due to the function of urate crystals in immune responses^32^. Further, Alzheimer’s disease shows moderate sharing with blood lipids (0.17–0.23), heart diseases (0.15–0.21), and inflammatory bowel diseases (0.10–0.13). This seems consistent with recent data linking Alzheimer’s disease to lipid metabolism^33^, vascular disorder^34^, and immune activation^35^. The biologically relevant clustering of shared pathway enrichments helps demonstrate the potential of large-scale GWAS data to highlight similarities among traits, complementing other approaches such as clustering of shared genetic effects^36^ and coheritability analyses^37^.

### Novel trait-associated genes informed by enriched pathways

A key feature of RSS-E is that pathway enrichments, once identified, are automatically used to prioritize associations at variants near genes in the pathway. Specifically, RSS-E gives almost identical estimates of the background parameter (*θ*_0_) in both baseline and enrichment analyses (Supplementary Fig. 24), and yields a positive estimate of the enrichment parameter (*θ*) if the pathway is identified as enriched (Supplementary Fig. 25). The positive estimate of *θ* increases the prior probability of association for SNPs in the pathway, which in turn increases the posterior probability of association for these SNPs.

This ability to prioritize associations, which is not shared by most enrichment methods, has several important benefits. Most obviously, prioritization analyses can detect additional genetic associations that may otherwise be missed. Furthermore, prioritization facilitates the identification of genes influencing a phenotype in two ways. First, it helps identify genes that may explain individual variant associations, which is itself an important and challenging problem^38^. Second, prioritization helps identify genes that drive observed pathway enrichments. This can be useful to check whether a pathway enrichment may actually reflect signal from just a few key genes, and to understand enrichments of pathways with generic functions.

To illustrate, we performed prioritization analyses on the trait-pathway pairs showing strongest evidence for enrichment. Following previous Bayesian analyses^7,39^, here we evaluated genetic associations at the level of loci, rather than individual SNPs. Specifically, for each locus we compute *P*_1_, the posterior probability that at least one SNP in the locus is associated with the trait, under both the baseline and enrichment hypothesis. Differences in these two *P*_1_ estimates reflect the influence of enrichment on the locus (Fig. 1).

**Fig. 5:**
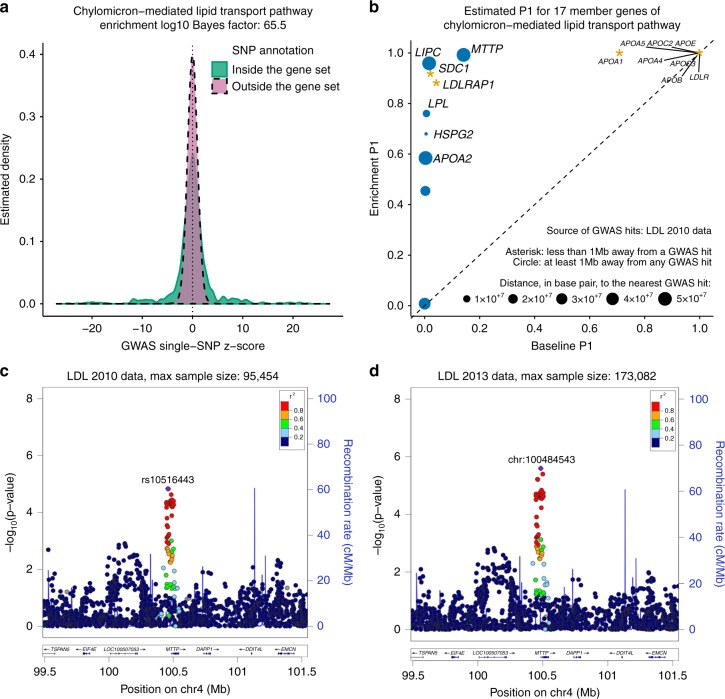
Enrichment of chylomicron-mediated lipid transport pathway informs a strong association between a member gene *MTTP* and levels of low-density lipoprotein (LDL) cholesterol. **a** Distribution of GWAS single-SNP *z*-scores from summary data published in 2010^41^, stratified by gene set annotations. The solid green curve is estimated from *z*-scores of SNPs within 100 kb of the transcribed region of genes in the chylomicron-mediated lipid transport pathway (“inside”), and the dashed reddish purple curve is estimated from *z*-scores of remaining SNPs (“outside”). This panel serves as a visual sanity check to confirm the observed enrichment. **b** Estimated posterior probability (*P*_1_) that there is at least one associated SNP within 100 kb of the transcribed region of each pathway-member gene under the enrichment model (*M*_1_) versus estimated *P*_1_ under the baseline model (*M*_0_). These gene-level *P*_1_ estimates and corresponding SNP-level statistics are provided in Supplementary Table 4. Yellow asterisks denote genes that are less than 1 Mb away from a GWAS hit. Blue circles denote genes that are at least 1 Mb away from any GWAS hit. **c** Regional association plot for *MTTP* based on summary data published in 2010^41^. **d** Regional association plot for *MTTP* based on summary data published in 2013^42^

The results show that prioritization analysis typically increases the inferred number of genetic associations (Supplementary Fig. 26), and uncovers putative associations that were not previously reported in GWAS. For example, enrichment in chylomicron-mediated lipid transport pathway (BF = 3.4 × 10^65^; Fig. 5a) informs a strong association between gene *MTTP* and levels of low-density lipoprotein (LDL) cholesterol (baseline *P*_1_: 0.14; enrichment *P*_1_: 0.99; Fig. 5b). This gene is a strong candidate for harboring associations with LDL: *MTTP* encodes microsomal triglyceride transfer protein, which has been shown to involve in lipoprotein assembly; mutations in *MTTP* cause abetalipoproteinemia, a rare disease characterized by low levels of apolipoprotein B and LDL cholesterol; and *MTTP* is a potential pharmacological target for lowering LDL cholesterol levels^40^. However, no genome-wide significant SNPs near *MTTP* were reported in single-SNP analyses of either the same data^41^ (Fig. 5c), or more recent data^42^ with larger sample size (Fig. 5d).

Prioritization analysis of the same pathway (chylomicron-mediated lipid transport) also yields several additional plausible associations (Fig. 5b; Supplementary Table 4). These include *LIPC* (baseline *P*_1_: 0.02; enrichment *P*_1_: 0.96) and *LPL* (baseline *P*_1_: 0.01; enrichment *P*_1_: 0.76). These genes play important roles in lipid metabolism and both reach genome-wide significance in single-SNP analyses of high-density lipoprotein cholesterol and triglycerides^41^ although not for LDL cholesterol (Supplementary Fig. 27); and a multiple-trait, single-SNP analysis^43^ of the same data also did not detect associations of these genes with LDL.

Several other examples of putatively novel associations that arise from our gene prioritization analyses are summarized in Table 2, with related literature reported in Supplementary Notes.

**Table 2:** Select putative gene-level associations from prioritization analyses

Phenotype	Pathway (# of genes, log_10_BF)	Gene	Baseline *P*_1_	Enrichment *P*_1_
Adult height	Endochondral ossification (65, 68.9)	*HDAC4*	0.98	1.00
		*PTH1R*	0.94	1.00
		*FGFR1*	0.67	0.97
		*MMP13*	0.45	0.93
Inflammatory bowel disease	Cytokine receptor interaction^a^ (253, 21.3)	*TNFRSF14*	0.98	1.00
		*FAS*	0.82	0.99
		*IL6*	0.27	0.87
Rheumatoid arthritis	NFAT-dependent transcription^b^ (45, 10.0)	*PTGS2*	0.74	0.98
		*PPARG*	0.28	0.98
Coronary artery disease	p75(NTR)-mediated signaling (55, 16.0)	*FURIN*	0.69	0.99
		*MMP3*	0.43	0.97
High-density lipoprotein	Lipid digestion and transport^c^ (58, 89.8)	*CUBN*	0.24	1.00
		*ABCG1*	0.01	0.89

BF: enrichment Bayes factor; *P*_*1*_: posterior probability that at least one SNP within 100 kb of the transcribed region of a given gene has nonzero effect on the target trait. NFAT: nuclear factor of activated T cells; p75(NTR): p75 neurotrophin receptor

^a^ The full name of this pathway is “cytokine-cytokine receptor interaction”

^b^ The full name of this pathway is “calcineurin-regulated NFAT-dependent transcription in lymphocytes”

^c^ The full name of this pathway is “lipid digestion, mobilization, and transport”

### Enrichment analyses of 113 tissue-based gene sets

RSS-E is not restricted to pathways, and can be applied more generally. Here, we use it to assess enrichment among tissue-based gene sets that we define based on gene expression data. Specifically, we use RNA sequencing data from the Genotype-Tissue Expression project^44^ to define sets of the most “relevant” genes in each tissue, based on expression patterns across tissues. The idea is that enrichment of GWAS signals near genes that are most relevant to a particular tissue may suggest an important role for that tissue in the trait.

A challenge here is how to define “relevant” genes. For example, are the highest expressed genes in a tissue the most relevant, even if the genes is ubiquitously expressed^45^? Or is a gene that is moderately expressed in that tissue, but less expressed in all other tissues, more relevant? To address this we considered three complementary methods to define tissue-relevant genes (Methods). The first method (“highly expressed”, HE) uses the highest expressed genes in each tissue. The second method (“selectively expressed”, SE) uses a tissue-selectivity score^46^ designed to identify genes that are much more strongly expressed in that tissue than in other tissues. The third method (“distinctively expressed”, DE) clusters tissue samples and identifies genes that are most informative for distinguishing each cluster from others^47^. This last method yields a list of “relevant” genes for each cluster, but most clusters are primarily associated with one tissue, and so we use this to assign genes to tissues. These methods often yield minimally overlapped gene sets for the same tissue (median overlap proportion: 4%; Supplementary Fig. 28).

Despite the small number of tissue-based gene sets relative to the pathway analyses above, this analysis identifies many strong enrichments. The top enriched tissues vary considerably among traits (Table 3), highlighting the benefits of analyzing a wide range of tissues. In addition, traits vary in which strategy for defining gene sets (HE, SE, or DE) yields the strongest enrichment results. For example, HE genes in heart show strongest enrichment for heart rate; SE genes in liver show strongest enrichment for LDL. This highlights the benefits of considering different annotation strategies, and suggests that, unsurprisingly, there is no single answer to the question of which genes are most “relevant” to a tissue.

**Table 3:** Top enriched tissue-based gene sets in complex traits

Phenotype	Tissue (method)		log_10_BF	Select top driving genes (# of signals)	
Alzheimer’s disease	Adrenal gland	(SE)	45.6	*APOE*, *APOC1*	(2)
Neuroticism	Brain	(SE)	26.3	*LINGO1*, *KCNC2*	(2)
Adult height	Nerve tibial	(DE)	25.2^b^	*PTCH1*, *SFRP4*, *FLNB*	(59)
Crohn’s disease	Cluster 1^a^	(DE)	15.4	*SMAD3*, *ZMIZ1*, *NUPR1*	(6)
Inflammatory bowel disease	Cluster 1^a^	(DE)	15.8	*SMAD3*, *ZMIZ1*, *NUPR1*	(10)
Ulcerative colitis	Heart	(HE)	7.0	*PLA2G2A*, *TCAP*, *ALDOA*	(4)
Age at natural menopause	Brain	(DE)	1053.2	*BRSK1*, *PPP1R1B*, *NPTXR*	(6)
Coronary artery disease	Brain	(DE)	8.5	*PSRC1*, *ZEB2*, *PTPN11*	(3)
Fasting glucose	Pancreas	(SE)	2396.8	*G6PC2*, *PDX1*, *SLC30A8*	(5)
Fasting insulin	Testis	(SE)	866.7	*ABHD1*, *PRR30*, *C2orf16*	(3)
Heart rate	Heart	(HE)	4.1	*MYH6*, *PLN*	(5)
High-density lipoprotein	Liver	(HE)	20.2	*APOA1*, *APOE*, *MT1G*, *FTH1*	(10)
Low-density lipoprotein	Liver	(SE)	33.4	*ABCG5*, *LPA*, *ANGPTL3*, *HP*	(13)
Total cholesterol	Liver	(DE)	56.0	*APOA1*, *APOE*, *HP*	(9)
Triglycerides	Liver	(HE)	93.2	*APOA1*, *APOE*, *FTH1*	(7)
Serum urate	Kidney	(SE)	210.8^b^	*SLC17A1*, *SLC22A11*, *PDZK1*	(7)
Hemoglobin (HB)	Whole blood	(DE)	2078.1	*HIST1H1E*, *HIST1H1C*	(4)
Mean cell HB	Whole blood	(DE)	1363.0	*NPRL3*, *FBXO7*, *UBXN6*	(11)
Mean cell volume	Whole blood	(DE)	1019.6^b^	*UBXN6*, *RBM38*, *NPRL3*	(11)
Packed cell volume	Heart	(HE)	945.4	*RPL19*, *TCAP*	(2)
Red blood cell count	Breast	(SE)	141.7	*OBP2B*, *STAC2*	(2)

Each tissue-based gene set contains 100 transcribed genes used in the Genotype-Tissue Expression project. For each trait we report the most enriched tissue-based gene set (if any) that has a Bayes factor (BF) greater than 1000 and has more than two member genes with enrichment *P*_1_ > 0.9 (denoted as “signals”). All trait-tissue pairs reported above pass the sanity checks (Supplementary Fig. 17) The corresponding baseline and enrichment parameter estimates are provided in online results (Methods). *P*_*1*_: posterior probability that at least one SNP within 100 kb of the transcribed region of a given gene has nonzero effect on the target trait; HE: highly expressed; SE: selectively expressed; DE: distinctively expressed

^a^ Multiple tissues show partial membership in “Cluster 1”, including ovary, thyroid, spleen, breast, and stomach^47^

^b^ These three BFs are smaller than corresponding BFs that SNPs within 100 kb of transcribed regions of all genes are enriched (Supplementary Fig. 19)

For some traits, the top enriched results (Table 3) recapitulate previously known trait-tissue connections (e.g., lipids and liver, glucose and pancreas), supporting the potential for our approach to identify trait-relevant tissues. Further, many traits show enrichments in multiple tissues. For example, associations in coronary artery disease are strongly enriched in genes related to both heart (SE, BF = 6.6 × 10^7^) and brain (DE, BF = 3.5 × 10^8^). The multiple-tissue enrichments highlight the potential for our approach to also produce novel biological insights, which we illustrate through an in-depth analysis of late-onset Alzheimer’s disease (LOAD).

**Fig. 6:**
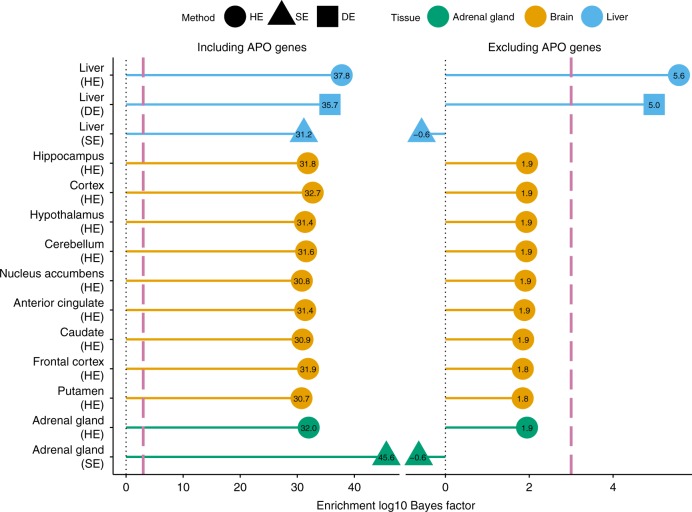
Enrichment analyses of genes related to liver, brain and adrenal gland for Alzheimer’s disease. Shown are the tissue-based gene sets with the strongest enrichment signals for Alzheimer’s disease. Each gene set was analyzed twice: the left panel corresponds to the analysis based on the original gene set; the right panel corresponds to the analysis where SNPs within 100 kb of the transcribed region of any gene in Apolipoproteins (APO) family (Methods) were excluded from the original gene set. Dashed reddish purple lines in both panel denote the same Bayes factor threshold (1000) used in the tissue-based analysis of all 31 traits (Table 3). HE highly expressed; SE selectively expressed; DE distinctively expressed

Tissue-based analysis of LOAD identified three tissues with very strong evidence for enrichment (BF > 10^30^): liver, brain and adrenal gland. Because of the well-known connection between gene *APOE* and LOAD^48^, and the fact that *APOE* is highly expressed in these three tissues (Supplementary Notes), we hypothesized that *APOE* and related genes might be driving these results. To assess this we reanalyzed these strongly enriched gene sets after removing the entire apolipoproteins (APO) gene family from them. Of three tissues, only liver remains (moderately) enriched after excluding APO genes (Fig. 6), suggesting a possible role for non-APO liver-related genes in the etiology of LOAD.

To identify additional genes underlying the liver enrichment, we performed prioritization analysis for non-APO liver-related genes. This highlighted an association of LOAD with gene *TTR* (baseline *P*_1_: 0.64; enrichment *P*_1_: 1.00; Supplementary Notes). *TTR* encodes transthyretin, which has been shown to inhibit LOAD-related protein from forming harmful aggregation and toxicity^49,50^. Indeed, transthyretin is considered a biomarker for LOAD: patients show reduced transthyretin levels in plasma^51^ and cerebrospinal fluid^52^. Rare variants in *TTR* have recently been found to be associated with LOAD^53,54^. By integrating GWAS with expression data our analysis identifies association of LOAD with *TTR* based on common variants.

## Discussion

We have presented RSS-E, a new method for simultaneous enrichment and prioritization analysis of GWAS summary data, and illustrated its potential to yield novel insights by extensive analyses involving 31 phenotypes and 4026 gene sets. We have space to highlight only select findings, and expect that researchers will find the full results (Methods) to contain further insights.

Enrichment tests, sometimes known as “competitive tests”, have several advantages over alternative approaches—sometimes known as “self-contained tests”—that simply test whether a SNP set contains at least one association^4,5^. For example, for complex polygenic traits any large pathway will likely contain at least one association, making self-contained tests unappealing. Enrichment tests are also more robust to confounding effects such as population stratification, because confounders that affect the whole genome will generally not create artifactual enrichments. Indeed, in this sense enrichment results can be more robust than single-SNP results. (Nonetheless, most summary data analyzed here were corrected for confounding; see Supplementary Table 5.)

Compared with other enrichment approaches, RSS-E has several particularly attractive features. First, unlike many methods (e.g., ^4,55^.) RSS-E uses data from genome-wide common variants, and not only those that pass some significance threshold. This increases the potential to identify subtle enrichments even in GWAS with few significant results. Second, RSS-E models enrichment directly as an increased rate of association of variants within a SNP set. This contrasts with alternative two-stage approaches (e.g., ^13,56^.) that first collapse SNP-level association statistics into gene-level statistics, and then assess enrichment at the gene level. Our direct modeling approach has important advantages, most obviously that it avoids the difficult and error-prone steps of assigning SNP associations to individual genes, and collapsing SNP-level associations into gene-level statistics. For example, simply assigning SNP associations to the nearest gene may highlight the “wrong” gene and miss the “correct” gene (e.g., ^38^). Although our analyses do involve assessing proximity of SNPs to genes in a gene set, they avoid uniquely assigning each SNP to a single gene, which is a subtle but important distinction.

Perhaps the most important feature of RSS-E is that enrichment leads naturally to prioritization that highlights which genes in an enriched pathways are most likely to be trait-associated. We know of only two published methods^7,57^ with similar features, but both require individual-level data and so could not perform the analyses presented here. With candidate loci prioritized by RSS-E, researchers can further use off-the-shelf fine-mapping methods^58^ to pinpoint associations to single causal variants.

Although previous studies have noted potential benefits of integrating gene expression with GWAS data, our enrichment analyses of expression-based gene sets are different from, and complementary to, this previous work. For example, many studies have used expression quantitative trait loci (eQTL) data to help inform GWAS results (e.g., ^59,60^). In contrast we bypass the issue of detecting (tissue-specific) eQTLs by focusing only on differences in gene expression levels among tissues. And, unlike methods that attempt to (indirectly) relate expression levels to phenotype (e.g.,^61,62^), our approach focuses firmly on genotype–phenotype associations. Nonetheless, as our results from different tissue-based annotations demonstrate, it can be extremely beneficial to consider multiple approaches, and we view these methods as complimentary rather than competing.

Like any method, RSS-E also has limitations that need to be considered when interpreting results. For example, annotating variants as being “inside” a gene set based on proximity to a relevant gene, while often effective, can occasionally give misleading results. We saw an example of this when our method identified an enrichment of SE genes in testis with both total cholesterol and triglycerides. Further prioritization analysis revealed that this enrichment was driven by a single gene, *C2orf16* which is (a) uniquely expressed in testis, and (b) physically close (53 kb) to another gene, *GCKR*, that is strongly associated with lipid traits (Supplementary Fig. 29). This highlights the need for careful examination of results, and also the utility of our prioritization analyses. Generally we view enrichments that are driven by a single gene as less reliable and useful than enrichments driven by multiple genes; indeed, enrichments driven by a single gene seem better represented as a gene association than as a gene set enrichment.

Other limitations of RSS-E stem from its use of variational inference for Bayesian calculations. Although these methods are computationally convenient in large datasets, and often produce reliable results, they also have features to be aware of. One feature is that when multiple SNPs in strong LD are associated with a trait, variational approximations tend to select one of them and ignore the others^9^. This feature will not greatly affect enrichment inference provided that SNPs that are in strong LD tend to have the same annotation (because then it will not matter which SNP is selected). And this holds for the gene-based annotations in the present study. However, it would not hold for finer-scale annotations (e.g., appearance in a DNase peak), and so in that setting the use of the variational approximation may need more care. More generally the accuracy of the variational approximation can be difficult to assess, especially since the underlying coordinate ascent algorithm only guarantees convergence to a local optimum. This said, the main alternative for making Bayesian calculations, Markov chain Monte Carlo, can experience similar difficulties.

Finally, the present study examines a single annotation (i.e., gene set) at a time. Practical issues that can occur in single-annotation analyses (not only ours) include: (a) an enrichment signal in one pathway can be caused by overlap with another pathway that is genuinely involved in the phenotype; and (b) for some traits (e.g., height), genetic associations may be strongly enriched near all genes, which will cause many gene sets to appear enriched. Extending RSS-E to jointly analyze multiple annotations like^14^ could help address these issues. However, this extension would increase computation costs, and we view the development of more efficient multiple-annotation enrichment methods as an important direction for future work.

## Methods

### GWAS summary statistics and LD estimates

We analyze GWAS summary statistics of 31 traits, in particular, the estimated single-SNP effect and standard error for each SNP. Following^14^, we use the same set of HapMap3 SNPs^15^ for all 31 traits, even though some traits have summary statistics available on all 1000 Genomes SNPs^16^. We use this “SNP subsetting” strategy to reduce computation, since the computational complexity of RSS-E (per iteration) is linear with the total number of SNPs (Supplementary Notes).

Among the HapMap3 SNPs, we also exclude SNPs on sex chromosomes, SNPs with minor allele frequency less than 1%, SNPs in the major histocompatibility complex region, and SNPs measured on custom arrays (e.g., Metabochip and Immunochip) from analyses. The final set of analyzed variants consists of 1.1 million SNPs (Supplementary Table 1, Supplementary Fig. 8).

Since GWAS summary statistics used here were all generated from European ancestry cohorts, we use haplotypes of individuals with European ancestry from the 1000 Genomes Project, Phase 3^16^ to estimate LD^10^.

### SNP annotations

To create SNP-level annotations for a given gene set, we use a distance-based approach from previous enrichment analyses^7,56^. Specifically, we annotate each SNP as being “inside” a gene set if it is within 100 kb of the transcribed region of a gene in the gene set. The relatively broad region is chosen to capture signals from nearby regulatory variants, since many GWAS hits are noncoding.

### Biological pathways

Biological pathway definitions are retrieved from nine databases (BioCarta, BioCyc, HumanCyc, KEGG, miRTarBase, PANTHER, PID, Reactome, WikiPathways) that are archived by four repositories: Pathway Commons (version 7)^63^, NCBI Biosystems^64^, PANTHER (version 3.3)^65^, and BioCarta (used in ref. ^7^). Gene definitions are based on *Homo sapiens* reference genome GRCh37. Both pathway and gene data were downloaded on August 24, 2015. Following^7^, we compile a list of 3913 pathways that contains 2–500 autosomal protein-coding genes for the present study. We summarize pathway and gene information in Supplementary Figures 15, 16.

### Tissue-based gene sets derived from transcriptome

Complex traits are often affected by multiple tissues, and it is not obvious a priori what the most relevant tissues are for the trait. Hence, it is necessary to examine a comprehensive set of tissues. The breadth of tissues in genotype-tissue expression (GTEx) project^44^ provides such an opportunity.

Here, we use RNA sequencing data to create 113 tissue-based gene sets. Due to the complex nature of extracting tissue relevance from sequencing data, we consider three different methods to derive tissue-based gene sets.

The HE method ranks the mean reads per kilobase per million mapped reads (RPKM) of all genes based on data of a given tissue, and then selects the top 100 genes with the largest mean RPKM values to represent the target tissue. We downloaded HE gene lists of 44 tissues with sample sizes greater than 70 from the GTEx Portal on November 21, 2016.

The SE method computes a tissue-selectivity (TS) score^46^ in each tissue for each gene, and then uses the top 100 genes with the largest TS scores to represent the target tissue. We obtained SE gene lists of 49 tissues from authors of ref. ^46^ on February 13, 2017.

The DE method summarizes 53 tissues as 20 clusters using admixture models^47^, computes a cluster-distinctiveness (CD) score in each cluster for each gene, and then uses the top 100 genes with the largest CD scores to represent the target cluster. We obtained DE gene lists of 20 clusters from authors of ref. ^47^ on May 19, 2016.

### Bayesian statistical models

Consider a GWAS with *n* unrelated individuals typed on *p* SNPs. For each SNP *j*, we denote its estimated single-SNP effect size and standard error as $\hat \beta _j$ and $\hat s_j$, respectively. To model $\{ \hat \beta _j,\hat s_j\}$, we use the RSS likelihood^8^:

1 $${\hat{\boldsymbol \beta }}\sim {\cal N}\left( {\hat {\bf{S}}\hat {\bf{R}}\hat {\bf{S}}^{ - {\mathrm{1}}}{\boldsymbol{\beta }},\hat {\bf{S}}\hat {\bf{R}}\hat {\bf{S}}} \right)$$

where ${\hat{\boldsymbol \beta }}: = (\hat \beta _1, \ldots ,\hat \beta _p)\prime$ is a *p* × 1 vector, ${\hat{\mathbf S}}: = {\mathrm{diag}}({\hat{\mathbf s}})$ is a *p* × *p* diagonal matrix with diagonal elements being ${\hat{\mathbf s}}: = (\hat s_1, \ldots ,\hat s_p)\prime$, ${\hat{\mathbf R}}$ is a *p* × *p* LD matrix estimated from an external reference panel with ancestry matching the GWAS cohort, ***β***: = (*β*_1_,…,*β*_*p*_)′ are the true effects of each SNP on phenotype, and ${\cal N}$ denotes normal distributions.

To model enrichment of genetic associations within a given gene set, we borrow the idea from refs. ^7,39^, to specify the following prior on ***β***:

2 $$\beta _j\sim \pi _j \cdot {\cal N}\left( {0,\sigma _\beta ^2} \right) + (1 - \pi _j) \cdot \delta _0,$$

3 $$\sigma _\beta ^2 = h \cdot \left( {{\sum} _{j = 1}^p\pi _jn^{ - 1}\hat s_j^{ - 2}} \right)^{ - 1},$$

4 $$\pi _j = \left( {1 + 10^{ - (\theta _0 + a_j\theta )}} \right)^{ - 1},$$

where *δ*_0_ denotes point mass at zero, *θ*_0_ reflects the background proportion of trait-associated SNPs, *θ* reflects the increase in probability, on the log10-odds scale, that a SNP inside the gene set has nonzero effect, *h* approximates the proportion of phenotypic variation explained by genotypes of all available SNPs, and *a*_*j*_ indicates whether SNP *j* is inside the gene set. Following^7^, we place independent uniform grid priors on the hyper-parameters {*θ*_0_, *θ*, *h*}; see Supplementary Tables 6, 7. (If one had specific information about hyper-parameters in a given application then this could be incorporated here.)

### Posterior computation

We combine the likelihood function (Eq. (1)) and prior distribution (Eqs. (2)–(4)) above to perform Bayesian inference. The posterior computation procedures largely follow those developed in ref. ^9^. First, for each set of hyper-parameters {*θ*_0_, *θ*, *h*} from a predefined grid, we approximate the (conditional) posterior of ***β*** using a variational Bayes algorithm. Next, we approximate the posterior of {*θ*_0_, *θ*, *h*} by a discrete distribution on the predefined grid, using the variational lower bounds from the first step to compute the discrete probabilities. Finally, we integrate out the conditional posterior of ***β*** over the posterior of {*θ*_0_, *θ*, *h*} to obtain the full posterior of ***β***.

Following^7^, we set random initialization as a default for the variational Bayes algorithm. Specifically, we randomly select an initialization, and then use this same initial value for all variational approximations over the grid of {*θ*_0_, *θ*, *h*}. This simple approach was used in all simulations and data analyses for the present study, and yielded satisfying results in most cases.

To facilitate large-scale analyses, we employ several computational tricks. First, we use squared iterative methods^11^ to accelerate the fixed point iterations in the variational approximation. Second, we exploit the banded LD matrix^10^ to parallelize the algorithm. Third, we use a simplification in ref. ^7^ that scales the enrichment analysis to thousands of gene sets by reusing expensive genome-wide calculations. See Supplementary Notes for details.

For one trait, the total computational cost of our analyses is determined by the number of whole-genome SNPs, the number of gene sets and the grid size for hyper-parameters, all of which can vary considerably among studies. It is thus hard to make general statements about computational time. However, to give a specific example, we finished baseline and enrichment analyses of 1.1 million HapMap3 SNPs and 3913 pathways for LDL within 36 h in a standard computer cluster (48 nodes, 12–16 CPUs per node).

All computations in the present study were performed on a Linux system with multiple (4–22) Intel E5–2670 2.6 GHz, Intel E5–2680 2.4 GHz or AMD Opteron 6386 SE processors.

### Assess gene set enrichment

To assess whether a gene set is enriched for genetic associations with a target trait, we evaluate a Bayes factor (BF):

5 $${\mathrm{BF}}: = \frac{{p({\hat{\boldsymbol \beta }}|{\hat{\mathbf S}},{\hat{\mathbf R}},{\mathbf a},\theta \,> \, 0)}}{{p({\hat{\boldsymbol \beta }}|{\hat{\mathbf S}},{\hat{\mathbf R}},{\mathbf a},\theta = 0)}},$$

where *p*(⋅) denotes probability densities, **a**: = (*a*_1_,…,*a*_*p*_)′ and *a*_*j*_ indicates whether SNP *j* is inside the gene set. The observed data are BF times more likely under the enrichment model (*M*_1_: *θ* > 0) than under the baseline model (*M*_0_: *θ* = 0), and so the larger the BF, the stronger evidence for gene set enrichment. See Supplementary Notes for details of computing enrichment BF.

### Detect association between a locus and a trait

To identify trait-associated loci, we consider two statistics derived from the posterior distribution of ***β***. The first statistic is *P*_1_, the posterior probability that at least one SNP in the locus is associated with the trait:

6 $$P_1: = 1 - {\mathrm{Pr}}\left( {\beta _j = 0,\forall \,{\mathrm{SNP}}\,j\, \in \,{\mathrm{locus}}|{\mathbf{D}}} \right),$$

where **D** is a shorthand for the input data of RSS-E including GWAS summary statistics $\left( {{\hat{\boldsymbol \beta }},{\hat{\mathbf S}}} \right)$, LD estimates $\left( {{\hat{\mathbf R}}} \right)$ and SNP annotations (**a**, if any). The second statistic is ENS, the posterior expected number of associated SNPs in the locus:

7 $${\mathrm{ENS}}: = {\sum} _{j \in {\mathrm{locus}}}{\mathrm{Pr}}(\beta _j \,\ne \,0|{\mathbf{D}}).$$

See Supplementary Notes for details of computing *P*_1_ and ENS.

### Estimate pairwise sharing of pathway enrichments

To capture pairwise sharing of enrichments, we define **π** = (*π*_00_, *π*_01_, *π*_10_, *π*_11_)′:

8 $$\pi _{ab}: = {\mathrm{Pr}}\left( {z_{1j} = a,z_{2j} = b} \right),a \in \{ 0,1\} ,b \in \{ 0,1\} ,$$

where *z*_*ij*_ equals one if pathway *j* is enriched in trait *i* and zero otherwise. Assuming independence among pathways and phenotypes, we estimate **π** by

9 $${\hat{\boldsymbol \pi }}: = \begin{array}{*{20}{c}} {{\mathrm{arg}}\,{\mathrm{max}}} \\ {\boldsymbol{\pi }} \end{array}{\prod} _j\left( {\pi _{00} + \pi _{01}{\mathrm{BF}}_{2j} + \pi _{10}{\mathrm{BF}}_{1j} + \pi _{11}{\mathrm{BF}}_{1j}{\mathrm{BF}}_{2j}} \right),$$

where BF_*ij*_ is the enrichment BF for trait *i* and pathway *j*. We solve this optimization problem using an expectation-maximization algorithm implemented in the package ashr^66^. The conditional probability that a pathway is enriched in a pair of traits given that it is enriched in at least one trait, as plotted in Fig. 4b, is estimated as $\hat \pi _{11}/(1 - \hat \pi _{00})$.

### Connection with enrichment analysis of individual-level data

RSS-E has close connection with previous work^7^ developed for individual-level data. Under certain conditions^8^, we can show that these two methods are mathematically equivalent, in the sense that they have the same fix point iteration scheme and lower bound in variational approximations. See Supplementary Notes for proofs. In addition to their theoretical connections, we also compared two methods through simulations, and observed similar inferential results (Supplementary Fig. 30).

### Code availability

The RSS-E software is publicly available at https://github.com/stephenslab/rss. Illustrations of using RSS-E are provided in https://stephenslab.github.io/rss/Example-5. The RSS-E software has been tested in the following versions of MATLAB for 64-bit Linux: 9.3.0.713579 (R2017b), 8.4.0.150421 (R2014b), 8.2.0.701 (R2013b) and version 8.1.0.604 (R2013a). Results of the present study were generated from version 8.4.0.150421 (R2014b).

This study also used the following software packages: Pascal (https://www2.unil.ch/cbg/index.php?title=Pascal), LDSC (version 1.0.0, https://github.com/bulik/ldsc), COMBAT (version 0.0.2, https://cran.r-project.org/web/packages/COMBAT), corrplot (version 0.84, https://cran.r-project.org/web/packages/corrplot), and ashr (version 2.0.5, https://cran.r-project.org/web/packages/ashr). Default setups of these packages were used.

## Electronic supplementary material

Supplementary Information

## Data Availability

Analysis results and all 4026 gene sets for the present study are publicly available at 10.5281/zenodo.1412872. The 4026 gene sets consist of 3913 biological pathways retrieved from the following four repositories: Pathway Commons (version 7, http://www.pathwaycommons.org/archives/PC2/v7), NCBI Biosystems (ftp://ftp.ncbi.nih.gov/pub/biosystems), PANTHER (version 3.3, ftp://ftp.pantherdb.org/pathway), BioCarta (used in ref. ^7^), and 113 tissue-based gene sets derived from GTEx transcriptome data (https://www.gtexportal.org/home/). Links to download GWAS summary statistics of 31 human phenotypes are provided in Supplementary Notes. The list of HapMap3 SNPs is available at https://data.broadinstitute.org/alkesgroup/LDSCORE/w_hm3.snplist.bz2. The 1000 Genomes Phase 3 data are available at ftp://ftp.1000genomes.ebi.ac.uk/vol1/ftp/release/20130502. The Wellcome Trust Case Control Consortium data are available at the European Genome-phenome Archive (https://www.ebi.ac.uk/ega/). The APO gene family is available at https://www.genenames.org/cgi-bin/genefamilies/set/405.
