# Supplementary material for: Large-scale genome-wide enrichment analyses identify new trait-associated genes and pathways across 31 human phenotypes
Source: Nat Commun. 2018 Oct 19;9:4361. doi: 10.1038/s41467-018-06805-x (PMC6195536; doi:10.1038/s41467-018-06805-x)
Supplement: Supplementary file 1 — Supplementary Information [file 41467_2018_6805_MOESM1_ESM.pdf]

Supplementary information cover page

Large-scale genome-wide enrichment analyses identify new trait-associated genes and pathways across 31 human phenotypes.

Zhu et al.

SUPPLEMENTARY INFORMATION OF "LARGE-SCALE GENOME-WIDE ENRICHMENT ANALYSES IDENTIFY NEW TRAIT-ASSOCIATED GENES AND PATHWAYS ACROSS 31 HUMAN PHENOTYPES"

By Xiang Zhu and Matthew Stephens

Stanford University and University of Chicago

CONTENTS

1 Supplementary Notes 4
1.1 Posterior computation 4
1.1.1 Estimate $p(\beta,\gamma|\mathbf{D},\theta_0,\theta,h)$ 4
1.1.2 Estimate $p(\theta_0,\theta,h|\mathbf{D})$ 5
1.1.3 Squared iterative method 6
1.2 Bayes factor for gene set enrichment 6
1.3 Posterior statistics of genetic associations 6
1.4 Estimate the fraction of trait-associated SNPs 7
1.5 Estimate the standardized effect size of trait-associated SNPs 7
1.6 Compute credible intervals 8
1.7 Scaling computation to many gene sets 8
1.8 Parallel implementation 8
1.9 Connection with variational inference based on individual-level data 9
1.10 Related literature of Table 2 11
1.11 Expression patterns of *APOE* and *TTR* across human tissues. 12
1.12 Acknowledgments and data sources 12
2 Supplementary Table 1 15
3 Supplementary Table 2 16
4 Supplementary Table 3 17
5 Supplementary Table 4 19
6 Supplementary Table 5 21
7 Supplementary Table 6 22
8 Supplementary Table 7 23
9 Supplementary Figure 1 24
10 Supplementary Figure 2 28
11 Supplementary Figure 3 31
12 Supplementary Figure 4 34
13 Supplementary Figure 5 37
14 Supplementary Figure 6 39
15 Supplementary Figure 7 41
16 Supplementary Figure 8 43
17 Supplementary Figure 9 45
18 Supplementary Figure 10 47
19 Supplementary Figure 11 49
19.1 Heart-related traits 49
19.2 Anthropometric traits 50
19.3 Immune-related traits 51

19.4 Blood lipid traits . . . 52
19.5 Hematopoietic traits . . . 53
19.6 Neurological phenotypes . . . 54
19.7 Insulin-related traits . . . 55
19.8 Other traits . . . 56
20 Supplementary Figure 12 . . . 57
20.1 All hits & $P_1 > 0.5$ . . . 58
20.2 Analyzed hits & $P_1 > 0.5$ . . . 59
20.3 All hits & $P_1 > 0.9$ . . . 60
20.4 Analyzed hits & $P_1 > 0.9$ . . . 61
20.5 All hits & ENS > 1 . . . 62
20.6 Analyzed hits & ENS > 1 . . . 63
21 Supplementary Figure 13 . . . 64
22 Supplementary Figure 14 . . . 65
22.1 All hits & $P_1 > 0.5$ . . . 66
22.2 Analyzed hits & $P_1 > 0.5$ . . . 67
22.3 All hits & $P_1 > 0.9$ . . . 68
22.4 Analyzed hits & $P_1 > 0.9$ . . . 69
22.5 All hits & ENS > 1 . . . 70
22.6 Analyzed hits & ENS > 1 . . . 71
23 Supplementary Figure 15 . . . 72
24 Supplementary Figure 16 . . . 73
25 Supplementary Figure 17 . . . 74
26 Supplementary Figure 18 . . . 109
27 Supplementary Figure 19 . . . 114
28 Supplementary Figure 20 . . . 116
28.1 Example 1 . . . 116
28.2 Example 2 . . . 117
29 Supplementary Figure 21 . . . 118
30 Supplementary Figure 22 . . . 171
31 Supplementary Figure 23 . . . 172
32 Supplementary Figure 24 . . . 187
33 Supplementary Figure 25 . . . 188
34 Supplementary Figure 26 . . . 189
35 Supplementary Figure 27 . . . 190
36 Supplementary Figure 28 . . . 191
37 Supplementary Figure 29 . . . 194
38 Supplementary Figure 30 . . . 195
References . . . 196
Author's addresses . . . 198

1. Supplementary Notes.

1.1. *Posterior computation.* Consider a GWAS with n unrelated individuals typed on p SNPs. For each SNP j, we denote its estimated single-SNP effect size and standard error as $\hat{\beta}_j$ and s_j respectively. To model $\{\hat{\beta}_j, s_j\}$, we use Regression with Summary Statistics (RSS) likelihood [1]:

$$\widehat{\boldsymbol{\beta}} \sim \mathcal{N}(\mathbf{SRS}^{-1}\boldsymbol{\beta},\ \mathbf{SRS}), \tag{1.1}$$

where $\widehat{\boldsymbol{\beta}} := (\hat{\beta}_1, \ldots, \hat{\beta}_p)'$ is a $p \times 1$ vector, $\mathbf{S} := \text{diag}(\mathbf{s})$ is a $p \times p$ diagonal matrix with diagonal elements being $\mathbf{s} := (s_1, \ldots, s_p)'$, $\mathbf{R}$ is a $p \times p$ LD matrix estimated from an external reference panel with ancestry matching the GWAS cohort, $\boldsymbol{\beta} := (\beta_1, \ldots, \beta_p)'$ are the true effects of SNPs under the multiple-SNP model, and $\mathcal{N}$ denotes normal distributions. (Note that we replace $\{\widehat{\mathbf{S}}, \widehat{\mathbf{R}}\}$ in the main text with $\{\mathbf{S}, \mathbf{R}\}$ throughout **Supplementary Notes** to simplify notation.) We then specify the following prior on the multiple regression coefficients $\boldsymbol{\beta}$:

$$\beta_j \sim \pi_j \cdot \mathcal{N}(0, \sigma_\beta^2) + (1 - \pi_j) \cdot \delta_0, \tag{1.2}$$

$$\sigma_\beta^2 = h \cdot \left(\textstyle\sum_{j=1}^{p} \pi_j n^{-1} \hat{s}_j^{-2} \right)^{-1}, \tag{1.3}$$

$$\pi_j = (1 + 10^{-(\theta_0 + a_j \theta)})^{-1}, \tag{1.4}$$

where δ_0 denotes point mass at zero, θ_0 reflects the background proportion of trait-associated SNPs under the multiple-SNP model, θ reflects the increase in probability, on the log10-odds scale, that a SNP inside the gene set has nonzero effect, h approximates the proportion of phenotypic variation explained by genotypes of all available SNPs, and a_j indicates whether SNP j is inside the gene set. Following [2], we place independent uniform grid priors on the hyper-parameters $\{\theta_0, \theta, h\}$ (**Supplementary Tables 6-7**).

We use variational inference to estimate the posterior distribution of $\boldsymbol{\beta}$ based on the input data $\mathbf{D} := \{\widehat{\boldsymbol{\beta}}, \mathbf{S}, \mathbf{R}, \mathbf{a}\}$, which includes GWAS summary statistics $(\widehat{\boldsymbol{\beta}}, \mathbf{S})$, LD estimates $(\mathbf{R})$ and SNP-level annotations $\mathbf{a} := (a_1, \ldots, a_p)'$. Before outlining the computation scheme, we first introduce a binary vector $\boldsymbol{\gamma} := (\gamma_1, \ldots, \gamma_p)' \in \{0,1\}^p$, where γ_j is a Bernoulli random variable which takes the value 1 with probability π_j and the value 0 with probability $1 - \pi_j$; $\gamma_j = 1$ when β_j is drawn from $\mathcal{N}(0, \sigma_\beta^2)$, and $\gamma_j = 0$ when β_j is drawn from δ_0.

The posterior computation procedures largely follow those developed in [3]. Firstly, for each set of hyper-parameters $\{\theta_0, \theta, h\}$ from a predefined grid, we approximate $p(\boldsymbol{\beta}, \boldsymbol{\gamma} | \mathbf{D}, \theta_0, \theta, h)$ using a mean-filed variational Bayes algorithm (Section 1.1.1). Next, we approximate $p(\theta_0, \theta, h | \mathbf{D})$ by a discrete distribution on the predefined grid, using the variational solutions from the first step to compute the posterior probabilities (Section 1.1.2). Finally, we integrate out $p(\boldsymbol{\beta}, \boldsymbol{\gamma} | \mathbf{D}, \theta_0, \theta, h)$ over the posterior of $p(\theta_0, \theta, h | \mathbf{D})$ to obtain the posterior of $\{\boldsymbol{\beta}, \boldsymbol{\gamma}\}$:

$$p(\boldsymbol{\beta}, \boldsymbol{\gamma} | \mathbf{D}) = \int p(\boldsymbol{\beta}, \boldsymbol{\gamma} | \mathbf{D}, \theta_0, \theta, h) p(\theta_0, \theta, h | \mathbf{D}) \mathrm{d}\theta_0 \mathrm{d}\theta \mathrm{d}h. \tag{1.5}$$

1.1.1. *Estimate $p(\boldsymbol{\beta}, \boldsymbol{\gamma} | \mathbf{D}, \theta_0, \theta, h)$.* The aim of the first step is to search for a distribution $q(\boldsymbol{\beta}, \boldsymbol{\gamma})$ that minimize the Kullback-Leibler (KL) divergence between $q(\boldsymbol{\beta}, \boldsymbol{\gamma})$ and $p(\boldsymbol{\beta}, \boldsymbol{\gamma} | \mathbf{D}, \theta_0, \theta, h)$.

For any distribution $q(\boldsymbol{\beta}, \boldsymbol{\gamma})$, we have the following decomposition:

$$\log p(\widehat{\boldsymbol{\beta}} | \mathbf{S}, \mathbf{R}, \mathbf{a}, \theta_0, \theta, h) = \underbrace{\mathrm{E}_q \log \left[\frac{q(\boldsymbol{\beta}, \boldsymbol{\gamma})}{p(\boldsymbol{\beta}, \boldsymbol{\gamma} | \mathbf{D}, \theta_0, \theta, h)} \right]}_{\text{Kullback-Leibler divergence}} + \underbrace{\mathrm{E}_q \log \left[\frac{p(\widehat{\boldsymbol{\beta}}, \boldsymbol{\beta}, \boldsymbol{\gamma} | \mathbf{S}, \mathbf{R}, \mathbf{a}, \theta_0, \theta, h)}{q(\boldsymbol{\beta}, \boldsymbol{\gamma})} \right]}_{\text{Variational lower bound}}. \tag{1.6}$$

Because the left-hand side of Equation (1.6) does not dependent on $\{\boldsymbol{\beta}, \boldsymbol{\gamma}\}$, minimizing KL divergence is equivalent to maximizing the variational lower bound. In the present study, we restrict the family of $q(\boldsymbol{\beta}, \boldsymbol{\gamma})$ to be of fully-factorized form (a.k.a. mean-field approximation):

$$q(\boldsymbol{\beta}, \boldsymbol{\gamma}) = \prod_{j=1}^{p} q_j(\beta_j, \gamma_j), \tag{1.7}$$

and do not make any additional assumption for q.

Straightforward algebra shows that for each q_j, the optimal variational solution $q_j^\star$ is given by

$$q_j^\star(\beta_j, \gamma_j) = \left[\alpha_j^\star \cdot \mathcal{N}(\beta_j; \mu_j^\star, (\sigma_j^\star)^2)\right]^{\gamma_j} \left[(1-\alpha_j^\star) \cdot \delta_0(\beta_j)\right]^{1-\gamma_j}, \tag{1.8}$$

implying that with probability $\alpha_j^\star$, β_j is normally distributed with mean $\mu_j^\star$ and variance $(\sigma_j^\star)^2$, and with probability $1-\alpha_j^\star$, β_j is zero. Following [3], we use a coordinate descent algorithm to estimate the variational parameters $\left\{\alpha_j^\star, \mu_j^\star, \sigma_j^\star\right\}$:

$$(\sigma_j^\star)^2 = (s_j^{-2} + \sigma_\beta^{-2})^{-1} \tag{1.9}$$

$$\mu_j^\star = (\sigma_j^\star)^2 \cdot \left(\frac{\hat{\beta}_j}{s_j^2} - \sum_{i \neq j} \frac{\mathbf{R}_{ij} \alpha_i^\star \mu_i^\star}{s_i s_j}\right) \tag{1.10}$$

$$\frac{\alpha_j^\star}{1-\alpha_j^\star} = \frac{\pi_j}{1-\pi_j} \cdot \frac{\sigma_j^\star}{\sigma_\beta} \cdot \exp\left\{\frac{(\mu_j^\star)^2}{2(\sigma_j^\star)^2}\right\} \tag{1.11}$$

Although not explicitly shown above, the optimal solution $q^\star$ depends on the values of hyper-parameters $\{\theta_0, \theta, h\}$, because π_j is a function of $\{\theta_0, \theta, h\}$.

1.1.2. *Estimate* $p(\theta_0, \theta, h|\mathbf{D})$. Since we use independent uniform grid priors for hyper-parameters $\{\theta_0, \theta, h\}$ (**Supplementary Tables 6-7**), the posterior distribution of $\{\theta_0, \theta, h\}$ is proportional to the marginal likelihood:

$$p(\theta_0, \theta, h|\mathbf{D}) = p(\theta_0, \theta, h|\widehat{\boldsymbol{\beta}}, \mathbf{S}, \mathbf{R}, \mathbf{a}) \propto p(\widehat{\boldsymbol{\beta}}|\mathbf{S}, \mathbf{R}, \mathbf{a}, \theta_0, \theta, h). \tag{1.12}$$

Noting that the marginal likelihood $p(\widehat{\boldsymbol{\beta}}|\mathbf{S}, \mathbf{R}, \mathbf{a}, \theta_0, \theta, h)$ is analytically intractable, we thus use variational lower bound as an approximation.

Using Jensen's inequality, we can see that the marginal log likelihood of (θ_0, θ, h) is bounded from below by the variational lower bound,

$$\log p(\widehat{\boldsymbol{\beta}}|\mathbf{S}, \mathbf{R}, \mathbf{a}, \theta_0, \theta, h) \geq \mathrm{E}_q \log\left[\frac{p(\widehat{\boldsymbol{\beta}}, \boldsymbol{\beta}, \boldsymbol{\gamma}|\mathbf{S}, \mathbf{R}, \mathbf{a}, \theta_0, \theta, h)}{q(\boldsymbol{\beta}, \boldsymbol{\gamma})}\right]. \tag{1.13}$$

Furthermore, if the distribution $q(\boldsymbol{\beta}, \boldsymbol{\gamma})$ takes the form (1.8), then the variational lower bound is analytically available:

$$\mathrm{E}_q \log\left[\frac{p(\widehat{\boldsymbol{\beta}}, \boldsymbol{\beta}, \boldsymbol{\gamma}|\mathbf{S}, \mathbf{R}, \mathbf{a}, \theta_0, \theta, h)}{q(\boldsymbol{\beta}, \boldsymbol{\gamma})}\right] = F_0(\mathbf{D}) + F(\mathbf{D}, \theta_0, \theta, h), \tag{1.14}$$

where $F_0(\mathbf{D})$ is a constant term with respect to $\{\theta_0, \theta, h\}$,

$$F_0(\mathbf{D}) = -\frac{1}{2}\log|2\pi \cdot \mathbf{SRS}| - \frac{1}{2}\widehat{\boldsymbol{\beta}}'(\mathbf{SRS})^{-1}\widehat{\boldsymbol{\beta}}, \tag{1.15}$$

and

$$F(\mathbf{D},\theta_0,\theta,h) = \widehat{\beta}'\mathbf{S}^{-2}\mathrm{E}_q(\beta) - \frac{1}{2}\mathrm{E}_q'(\beta)\mathbf{S}^{-1}\mathbf{R}\mathbf{S}^{-1}\mathrm{E}_q(\beta) - \frac{1}{2}\sum_{j=1}^{p}\frac{\mathrm{Var}_q(\beta_j)}{s_j^2} - \sum_{j=1}^{p}\alpha_j\log\left(\frac{\alpha_j}{\pi_j}\right)$$

$$- \sum_{j=1}^{p}(1-\alpha_j)\log\left(\frac{1-\alpha_j}{1-\pi_j}\right) + \sum_{j=1}^{p}\frac{\alpha_j}{2}\left[1+\log\left(\frac{\sigma_j^2}{\sigma_\beta^2}\right) - \frac{\sigma_j^2+\mu_j^2}{\sigma_\beta^2}\right], \tag{1.16}$$

$\mathrm{E}_q(\beta) = (\mathrm{E}_q(\beta_1),\ldots,\mathrm{E}_q(\beta_p))'$, $\mathrm{E}_q(\beta_j) = \alpha_j\mu_j$ and $\mathrm{Var}_q(\beta_j) = \alpha_j(\sigma_j^2+\mu_j^2)-(\alpha_j\mu_j)^2$. Note that the analytic form of lower bound (1.14) holds even when parameters $\{\alpha_j,\mu_j,\sigma_j\}$ are *not* constrained by the optimal variational solution (1.9)-(1.11).

Finally, $p(\theta_0,\theta,h|\mathbf{D})$ is estimated as a discrete distribution $\widetilde{w}(\theta_0,\theta,h)$:

$$p(\theta_0,\theta,h|\mathbf{D}) \approx \widetilde{w}(\theta_0,\theta,h) \propto \exp\{F(\mathbf{D},\theta_0,\theta,h)\}. \tag{1.17}$$

Note that $\widetilde{w}(\theta_0,\theta,h)$ is discrete because the support of $\{\theta_0,\theta,h\}$ is discrete (i.e. a uniform grid).

1.1.3. *Squared iterative method.* When estimating $p(\beta,\gamma|\mathbf{D},\theta_0,\theta,h)$, the coordinate descent updates (1.9)-(1.11) essentially define a fixed-point mapping. To improve the convergence, we embed an "off-the-shelf" accelerator, squared iterative methods (SQUAREM, [4]), in this fixed-point mapping. Simulations show that variational inference with and without the SQUAREM accelerator often yield almost identical results (**Supplementary Figure 30**).

1.2. *Bayes factor for gene set enrichment.* To measure the evidence for the enrichment hypothesis that a candidate gene set is enriched ($\theta > 0$) for phenotype-genotype associations against the baseline hypothesis ($\theta = 0$), we evaluate the following Bayes factor (BF):

$$\mathrm{BF} = \frac{p(\widehat{\beta}|\mathbf{S},\mathbf{R},\mathbf{a},\theta>0)}{p(\widehat{\beta}|\mathbf{S},\mathbf{R},\mathbf{a},\theta=0)}. \tag{1.18}$$

To compute BF (1.18), we approximate intractable marginal likelihoods by corresponding variational lower bounds (1.16):

$$\begin{aligned}\mathrm{BF} &= \frac{\int p(\widehat{\beta}|\mathbf{S},\mathbf{R},\mathbf{a},\theta_0,\theta,h)p(\theta_0)p(\theta)p(h)\mathrm{d}\theta\mathrm{d}\theta_0\mathrm{d}h}{\int p(\widehat{\beta}|\mathbf{S},\mathbf{R},\mathbf{a},\theta_0,\theta=0,h)p(\theta_0)p(h)\mathrm{d}\theta_0\mathrm{d}h}\\ &\approx \frac{\int \exp\{F_0(\mathbf{D})+F(\mathbf{D},\theta_0,\theta,h)\}p(\theta_0)p(\theta)p(h)\mathrm{d}\theta\mathrm{d}\theta_0\mathrm{d}h}{\int \exp\{F_0(\mathbf{D})+F(\mathbf{D},\theta_0,\theta=0,h)\}p(\theta_0)p(h)\mathrm{d}\theta_0\mathrm{d}h}\\ &\approx \frac{n_1^{-1}\sum_{s=1}^{n_1}\exp\{F(\mathbf{D},\theta_0^{(s)},\theta^{(s)},h^{(s)})\}}{n_0^{-1}\sum_{t=1}^{n_0}\exp\{F(\mathbf{D},\theta_0^{(t)},\theta=0,h^{(t)})\}},\end{aligned} \tag{1.19}$$

where $\left\{\theta_0^{(s)},\theta^{(s)},h^{(s)}\right\}$ and $\left\{\theta_0^{(t)},h^{(t)}\right\}$ are evenly spaced points on a regular grid over finite intervals.

1.3. *Posterior statistics of genetic associations.* To identify loci associated with a given phenotype, we consider two posterior statistics derived from the variational inference.

The first statistic is P_1, the posterior probability that at least one SNP in a given locus is associated with the phenotype:

$$P_1 := 1 - \Pr\left(\beta_j = 0,\ \forall \text{ SNP } j \in \text{locus}|\mathbf{D}\right). \tag{1.20}$$

Given a grid $\left\{\theta_0^{(s)}, \theta^{(s)}, h^{(s)}\right\}$, P_1 is estimated as

$$\begin{aligned} P_1 &= 1 - \int \Pr(\beta_j = 0,\ \forall j \in \text{locus}|\mathbf{D}, \theta_0, \theta, h) p(\theta_0, \theta, h|\mathbf{D}) \mathrm{d}\theta_0 \mathrm{d}\theta \mathrm{d}h \\ &\approx 1 - \sum_{s=1}^{n_1} \Pr(\beta_j = 0,\ \forall j \in \text{locus}|\mathbf{D}, \theta_0^{(s)}, \theta^{(s)}, h^{(s)}) \cdot \widetilde{w}(\theta_0^{(s)}, \theta^{(s)}, h^{(s)}). \end{aligned}$$

Since the posterior of $\boldsymbol{\beta}$ is approximated by a fully-factorized distribution (1.7), for any (θ_0, θ, h),

$$\Pr(\beta_j = 0,\ \forall j \in \text{locus}|\mathbf{D}, \theta_0, \theta, h) \approx \prod_{j \in \text{locus}} q_j^\star(\beta_j = 0) = \prod_{j \in \text{locus}} \left[1 - \alpha_j^\star(\theta_0, \theta, h)\right].$$

Hence, the final estimate of P_1 averaged over the grid $\left\{\theta_0^{(s)}, \theta^{(s)}, h^{(s)}\right\}$ is

$$P_1 \approx 1 - \sum_{s=1}^{n_1} \prod_{j \in \text{locus}} \left[1 - \alpha_j^\star(\theta_0^{(s)}, \theta^{(s)}, h^{(s)})\right] \cdot \widetilde{w}(\theta_0^{(s)}, \theta^{(s)}, h^{(s)}). \tag{1.21}$$

The second statistic is ENS, the posterior expected number of associated SNPs in the locus:

$$\text{ENS} := \sum_{j \in \text{locus}} \Pr(\beta_j \neq 0|\mathbf{D}). \tag{1.22}$$

Given a grid $\left\{\theta_0^{(s)}, \theta^{(s)}, h^{(s)}\right\}$ and corresponding variational estimates, ENS is estimated as

$$\text{ENS} \approx \sum_{j \in \text{locus}} \sum_{s=1}^{n_1} \alpha_j^\star(\theta_0^{(s)}, \theta^{(s)}, h^{(s)}) \cdot \widetilde{w}(\theta_0^{(s)}, \theta^{(s)}, h^{(s)}). \tag{1.23}$$

Note that unlike P_1, the estimate of ENS does not require the fully-factorized assumption (1.7).

We compute P_1 (1.21) and ENS (1.23) above under the enrichment hypothesis that the candidate gene set is enriched ($\theta > 0$). Similarly, under the baseline hypothesis that no gene set is enriched ($\theta = 0$), the numerical estimates of P_1 and ENS are given by:

$$P_1 \approx 1 - \sum_{t=1}^{n_0} \prod_{j \in \text{locus}} \left[1 - \alpha_j^\star(\theta_0^{(t)}, \theta = 0, h^{(t)})\right] \cdot \widetilde{w}(\theta_0^{(t)}, \theta = 0, h^{(t)}), \tag{1.24}$$

$$\text{ENS} \approx \sum_{j \in \text{locus}} \sum_{t=1}^{n_0} \alpha_j^\star(\theta_0^{(t)}, \theta = 0, h^{(t)}) \cdot \widetilde{w}(\theta_0^{(t)}, \theta = 0, h^{(t)}). \tag{1.25}$$

1.4. *Estimate the fraction of trait-associated SNPs.* The fraction of trait-associated SNPs is one of the two quantities that we use to summarize the effect size distribution of a trait (x-axes of **Figure 4(a)** and **Supplementary Figure 9**). This quantity is estimated as:

$$\frac{1}{p} \sum_{j=1}^{p} \sum_{t=1}^{n_0} \alpha_j^\star(\theta_0^{(t)}, \theta = 0, h^{(t)}) \cdot \widetilde{w}(\theta_0^{(t)}, \theta = 0, h^{(t)}). \tag{1.26}$$

1.5. *Estimate the standardized effect size of trait-associated SNPs.* The standardized effect size of trait-associated SNPs is another quantity that we use to summarize the effect size distribution of a trait (y-axes of **Figure 4(a)** and **Supplementary Figure 9**). For a given variational approximation $q^\star$, we estimate this quantity as

$$\frac{\sum_{j=1}^{p} \alpha_j^\star \mu_j^\star}{\hat{\sigma}_y \cdot \sum_{j=1}^{p} \alpha_j^\star}. \tag{1.27}$$

Here $\hat{\sigma}_y^2$ is the sample variance of phenotype measurements, which is often not publicly available but can be estimated as follows:

$$\hat{\sigma}_y^2 \approx 2n_j f_j(1-f_j)s_j^2, \tag{1.28}$$

where f_j and n_j are the minor allele frequency and sample size of SNP j. Although the approximated values of $\hat{\sigma}_y^2$ sometimes differ across SNPs because of different $\{n_j, f_j, s_j\}$, they often fall into a small range, and thus we use the median across SNPs as a final estimate.

1.6. *Compute credible intervals.* Following [2], we use $\tilde{w}(\theta_0,\theta,h)$, the variational estimate of $p(\theta_0,\theta,h|\mathbf{D})$, to compute a credible interval for any quantity that depends on $\{\theta_0,\theta,h\}$. Specifically, for a predefined grid $\left\{\theta_0^{(s)},\theta^{(s)},h^{(s)}\right\}$ and a quantity $Q(\theta_0,\theta,h)$, we add up the variational estimates $\tilde{w}(\theta_0^{(s)},\theta^{(s)},h^{(s)})$ over successively wider intervals of $Q(\theta_0,\theta,h)$, beginning at the posterior mean, until the sum of $\tilde{w}(\theta_0^{(s)},\theta^{(s)},h^{(s)})$ reaches a given interval coverage (e.g. 0.95).

1.7. *Scaling computation to many gene sets.* When performing genome-wide enrichment analysis of thousands of gene sets in the present study, we exploit a simplification introduced in [2], which allows us to reuse "expensive" whole-genome calculations and thus reduce computing time. Specifically, we assume that SNPs that are not near any member gene of the enriched gene set (i.e. "outside") are unaffected by the inferred enrichment *a posteriori*:

$$q^\star(\beta_{\bar{A}};\mathbf{D},\theta_0,\theta,h) = q^\star(\beta_{\bar{A}};\mathbf{D},\theta_0,\theta=0,h), \tag{1.29}$$

where $q^\star$ is the estimated variational posterior distribution of $\boldsymbol{\beta}$, A is the set of SNPs assigned to the enriched gene set (i.e. "inside"), and $\bar{A}$ is complement of A. Since the outside set $\bar{A}$ typically contains most of SNPs in the whole genome, we only need to re-estimate the variational posterior distribution for a relatively small number of "inside" SNPs under assumption (1.29).

Following [2], we approximate variational lower bound as follows:

$$F(\mathbf{D},\theta_0,\theta,h) \approx F(\mathbf{D},\theta_0,\theta=0,h) + F_A(\mathbf{D}_A,\theta_0,\theta,h) - F_A(\mathbf{D}_A,\theta_0,\theta=0,h), \tag{1.30}$$

where for each set $I \in \{A,\bar{A}\}$,

$$\begin{aligned} F_I(\mathbf{D}_I,\theta_0,\theta,h) &= \widehat{\beta}_I'\mathbf{S}_I^{-2}\mathrm{E}_q(\beta_I) - \frac{1}{2}(\mathrm{E}_q(\beta_I))'\mathbf{S}_I^{-1}\mathbf{R}_I\mathbf{S}_I^{-1}\mathrm{E}_q(\beta_I) - \frac{1}{2}\sum_{j\in I}\frac{\mathrm{Var}_q(\beta_j)}{s_j^2} - \sum_{j\in I}\alpha_j\log\left(\frac{\alpha_j}{\pi_j}\right) \\ &\quad - \sum_{j\in I}(1-\alpha_j)\log\left(\frac{1-\alpha_j}{1-\pi_j}\right) + \sum_{j\in I}\frac{\alpha_j}{2}\left[1+\log\left(\frac{\sigma_j^2}{\sigma_\beta^2}\right) - \frac{\sigma_j^2+\mu_j^2}{\sigma_\beta^2}\right], \end{aligned} \tag{1.31}$$

$\mathbf{D}_I := \{\widehat{\beta}_I, \mathbf{S}_I, \mathbf{R}_I, \mathbf{a}_I\}$, $\mathbf{S}_I := \mathrm{diag}(\mathbf{s}_I)$, $\widehat{\beta}_I$ and $\mathbf{s}_I$ are the vectors of single-SNP effect size estimates and corresponding standard errors that are restricted to SNPs in the set I, and $\mathbf{R}_I$ is the LD matrix of SNPs in the set I. Note that $F(\mathbf{D},\theta_0,\theta=0,h)$ has already been computed when we fit the baseline model on whole-genome summary data $(M_0:\theta=0)$. Hence, calculation of (1.30) only requires refitting the baseline $(M_0:\theta=0)$ and enrichment $(M_1:\theta>0)$ models on a relatively small dataset $\mathbf{D}_A$ to obtain corresponding lower bounds $F_A(\mathbf{D}_A,\theta_0,\theta=0,h)$ and $F_A(\mathbf{D}_A,\theta_0,\theta,h)$.

1.8. *Parallel implementation.* To speed up the whole genome analysis, we implement the estimation of $p(\boldsymbol{\beta},\boldsymbol{\gamma}|\mathbf{D},\theta_0,\theta,h)$ in parallel across multiple threads.

Our parallel implementation is built on the assumption that $\mathbf{R}_{ij}=0$ if SNPs i and j are on different chromosomes. If this assumption holds, the coordinate descent updates (1.9)-(1.11) for the variational

parameters $\{\alpha_j, \mu_j, \sigma_j\}$ of SNP j on Chromosome c only requires $\mathbf{D}_c$, where $\mathbf{D}_c := \{\widehat{\beta}_c, \mathbf{S}_c, \mathbf{R}_c, \mathbf{a}_c\}$ denotes the input data from Chromosome c. Further, the variational lower bound $F(\mathbf{D}, \theta_0, \theta, h)$ based on whole genome data has the following decomposition:

$$F(\mathbf{D}, \theta_0, \theta, h) = \sum_{c=1}^{22} F_c(\mathbf{D}_c, \theta_0, \theta, h), \tag{1.32}$$

where F_c is defined in (1.31).

Algorithm 1 outlines the parallel implementation. First, we partition the whole-genome input data $\mathbf{D}$ into 22 sub-data $\{\mathbf{D}_c\}$ by chromosomes. To perform parallel calculation, we request 22 threads from a single computer, each of which is responsible for updating the variational parameters $\{\alpha_j, \mu_j, \sigma_j\}$ and computing the variational lower bound $F_c(\mathbf{D}_c, \theta_0, \theta, h)$ on each chromosome c; see line 6 of Algorithm 1. Finally, we aggregate the per-chromosome updated variational parameters and lower bounds in the "reduce" step; see line 8 of Algorithm 1.

Algorithm 1 Parallel implementation

1: **for** $s = 1$ to N **do** ▷ outer loop
2: initialize $\boldsymbol{\alpha}^{(s)}$ and $\boldsymbol{\mu}^{(s)}$ randomly
3: compute $\boldsymbol{\sigma}^{(s)}$ by (1.9)
4: **repeat** ▷ inner loop
5: **for** Chromosome $c = 1$ to 22 **do** ▷ parallel step
6: use sub-data $\mathbf{D}_c$ to update $\boldsymbol{\alpha}^{(c,s)}$, $\boldsymbol{\mu}^{(c,s)}$ and $F_c(\mathbf{D}_c, \theta_0^{(s)}, \theta^{(s)}, h^{(s)})$ by (1.11), (1.10) and (1.31)
7: **end for**
8: aggregate chromosome-level results: ▷ reduce step

$$\boldsymbol{\alpha}^{(s)} = \left[\boldsymbol{\alpha}^{(1,s)}; \ldots; \boldsymbol{\alpha}^{(22,s)}\right] \tag{1.33}$$

$$\boldsymbol{\mu}^{(s)} = \left[\boldsymbol{\mu}^{(1,s)}; \ldots; \boldsymbol{\mu}^{(22,s)}\right] \tag{1.34}$$

$$F(\mathbf{D}, \theta_0^{(s)}, \theta^{(s)}, h^{(s)}) = \sum_{c=1}^{22} F_c(\mathbf{D}_c, \theta_0^{(s)}, \theta^{(s)}, h^{(s)}) \tag{1.35}$$

9: **until** convergence criteria are met
10: compute the posterior weight $\widetilde{w}(\theta_0^{(s)}, \theta^{(s)}, h^{(s)})$ by (1.17)
11: **end for**
12: integrate out hyper-parameters $\{\theta_0, \theta, h\}$:

$$\widetilde{\boldsymbol{\alpha}} = \sum_{s=1}^{N} \widetilde{w}(\theta_0^{(s)}, \theta^{(s)}, h^{(s)}) \cdot \boldsymbol{\alpha}^{(s)} \tag{1.36}$$

$$\widetilde{\boldsymbol{\mu}} = \sum_{s=1}^{N} \widetilde{w}(\theta_0^{(s)}, \theta^{(s)}, h^{(s)}) \cdot \boldsymbol{\mu}^{(s)} \tag{1.37}$$

$$\widetilde{\boldsymbol{\sigma}} = \sum_{s=1}^{N} \widetilde{w}(\theta_0^{(s)}, \theta^{(s)}, h^{(s)}) \cdot \boldsymbol{\sigma}^{(s)} \tag{1.38}$$

1.9. *Connection with variational inference based on individual-level data.* In our previous work [1], we derived the conditions under which regression likelihood based on individual-level data is equivalent to regression likelihood based on summary-level data . Under the same conditions, here we show that variational inferences based on individual-level data [3] and summary-level data [1] are also equivalent.

PROPOSITION 1.1. Let $\hat{\sigma}_y^2$ denote the sample variance of individual-level phenotypes $\mathbf{y}$, $\widehat{\mathbf{R}}^{\mathsf{sam}}$ denote the sample correlation matrix of individual-level genotypes $\mathbf{X}$, and σ^2 denote the residual

variance in the following multiple linear regression model:

$$\mathbf{y} = \mathbf{X}\beta + \epsilon,\ \epsilon \sim \mathcal{N}(\mathbf{0}, \sigma^2\mathbf{I}).$$

If $\sigma^2 = \hat{\sigma}_y^2$ and $\mathbf{R} = \widehat{\mathbf{R}}^{\text{sam}}$, the coordinate descent equations (1.9)-(1.11) based on summary-level data yields the *same* solution of $\{\alpha_j, \mu_j, \sigma_j\}$ as the equations based on individual-level data (Equations 8-10 in [3]).

PROOF. Following the notation in [3], we write $\sigma_\beta^2 = \sigma_a^2\sigma^2$, and write the coordinate updates of $\{\alpha_j, \mu_j, \sigma_j\}$ based on individual-level data (Equations 8-10 in [3]) as follows:

$$\sigma_j^2 = \frac{\sigma^2}{\mathbf{X}_j'\mathbf{X}_j + \sigma_a^{-2}}, \tag{1.39}$$

$$\mu_j = \frac{\sigma_j^2}{\sigma^2} \cdot \left(\mathbf{X}_j'\mathbf{y} - \sum_{i \neq j} \mathbf{X}_j'\mathbf{X}_i \alpha_i \mu_i \right), \tag{1.40}$$

$$\frac{\alpha_j}{1-\alpha_j} = \frac{\pi_j}{1-\pi_j} \cdot \frac{\sigma_j}{\sigma_a \sigma} \cdot \exp\left\{ \frac{\mu_j^2}{2\sigma_j^2} \right\}. \tag{1.41}$$

Based on the definition of $\widehat{\beta}$, $\mathbf{S}$ and $\widehat{\mathbf{R}}^{\text{sam}}$, we have:

$$\mathbf{X}_j^\mathsf{T}\mathbf{y} = (\mathbf{X}_j^\mathsf{T}\mathbf{X}_j) \cdot \hat{\beta}_j,\ \ \mathbf{X}_j^\mathsf{T}\mathbf{X}_j = s_j^{-2}\hat{\sigma}_y^2,\ \ \mathbf{X}_j^\mathsf{T}\mathbf{X}_i = ||\mathbf{X}_j|| \cdot ||\mathbf{X}_i|| \cdot \widehat{\mathbf{R}}_{ij}^{\text{sam}}, \tag{1.42}$$

and then we can rewrite the updates above as follows:

$$\sigma_j^2 = \frac{\sigma^2}{s_j^{-2}\hat{\sigma}_y^2 + \sigma_a^{-2}}, \tag{1.43}$$

$$\mu_j = \frac{\sigma_j^2 \hat{\sigma}_y^2}{\sigma^2} \cdot \left(\frac{\hat{\beta}_j}{s_j^2} - \sum_{i \neq j} \frac{\widehat{\mathbf{R}}_{ij}^{\text{sam}} \alpha_i \mu_i}{s_i s_j} \right), \tag{1.44}$$

$$\frac{\alpha_j}{1-\alpha_j} = \frac{\pi_j}{1-\pi_j} \cdot \frac{\sigma_j}{\sigma_a \sigma} \cdot \exp\left\{ \frac{\mu_j^2}{2\sigma_j^2} \right\}. \tag{1.45}$$

Finally, if $\sigma^2 = \hat{\sigma}_y^2$ and $\mathbf{R}_{ij} = \widehat{\mathbf{R}}_{ij}^{\text{sam}}$, these coordinate descent updates based on individual-level data are the same as the coordinate descent updates (1.9), (1.10) and (1.11) that are based on summary-level data. □

Under the same conditions ($\sigma^2 = \hat{\sigma}_y^2$ and $\mathbf{R} = \widehat{\mathbf{R}}^{\text{sam}}$), we can also show that the variational lower bound based on individual-level data and summary-level data are equivalent.

PROPOSITION 1.2. If $\sigma^2 = \hat{\sigma}_y^2$ and $\mathbf{R} = \widehat{\mathbf{R}}^{\text{sam}}$, the difference between the variational lower bound based on summary-level data (1.14) and individual-level data (Equation 13 in [3]) is a constant with respect to the variational parameters $\{\alpha_j, \mu_j, \sigma_j\}$.

The proof is similar to Proposition 1.1, so it is omitted here.

1.10. *Related literature of Table 2.* Below is a list of related literature for **Table 2**.

Adult height [5] and endochondral ossification pathway (65 genes, $\log_{10}$ BF = 68.9)

- *HDAC4* (baseline P_1: 0.98; enrichment P_1: 1.00)
  HDAC4 encodes a critical regulator of chondrocyte hypertrophy during skeletogenesis [6] and osteoclast differentiation [7]. Haploinsufficiency of *HDAC4* results in chromosome 2q37 deletion syndrome (OMIM: 600430) with highly variable clinical manifestations including developmental delay and skeletal malformations.
- *PTH1R* (baseline P_1: 0.94; enrichment P_1: 1.00)
  PTH1R encodes a receptor that regulates skeletal development, bone turnover and mineral ion homeostasis [8]. Mutations in *PTH1R* cause several rare skeletal disorders (OMIM: 215045, 600002, 156400).
- *FGFR1* (baseline P_1: 0.67; enrichment P_1: 0.97)
  FGFR1 encodes a receptor that regulates limb development, bone formation and phosphorus metabolism [9]. Mutations in *FGFR1* cause several skeletal disorders (OMIM: 101600, 123150, 190440, 166250).
- *MMP13* (baseline P_1: 0.45; enrichment P_1: 0.93)
  MMP13 encodes a protein that is required for osteocytic perilacunar remodeling and bone quality maintenance [10]. Mutations in *MMP13* cause a type of metaphyseal anadysplasia (OMIM: 602111) with reduced stature.

Inflammatory bowel disease [11] and cytokine-cytokine receptor interaction pathway (253 genes, $\log_{10}$ BF = 21.3)

- *TNFRSF14* (a.k.a. *HVEM*; baseline P_1: 0.98; enrichment P_1: 1.00)
  TNFRSF14 encodes a receptor that functions in signal transduction pathways activating inflammatory and inhibitory T-cell immune response. *TNFRSF14* expression plays a crucial role in preventing intestinal inflammation [12]. *TNFRSF14* is near a GWAS hit of celiac disease (rs3748816, $p = 3.3 \times 10^{-9}$) [13] and two hits of ulcerative colitis (rs734999, $p = 3.3 \times 10^{-9}$ [14]; rs10797432, $p = 3.0 \times 10^{-12}$ [15]).
- *FAS* (baseline P_1: 0.82; enrichment P_1: 0.99)
  FAS plays many important roles in the immune system [16]. Mutations in *FAS* cause autoimmune lymphoproliferative syndrome (OMIM: 601859).
- *IL6* (baseline P_1: 0.27; enrichment P_1: 0.87)
  IL6 encodes a cytokine that functions in inflammation and the maturation of B cells, and has been suggested as a potential therapeutic target in IBD [17].

Coronary artery disease [18] and p75(NTR)-mediated signaling pathway (55 genes, $\log_{10}$ BF = 16.0)

- *FURIN* (baseline P_1: 0.69; enrichment P_1: 0.99)
  FURIN encodes the major processing enzyme of a cardiac-specific growth factor, which plays a critical role in heart development [19]. *FURIN* is near a GWAS hit (rs2521501 [20]) of both systolic blood pressure ($p = 5.2 \times 10^{-19}$) and hypertension ($p = 1.9 \times 10^{-15}$).
- *MMP3* (baseline P_1: 0.43; enrichment P_1: 0.97)
  A polymorphism in the promoter region of *MMP3* is associated with susceptibility to coronary heart disease-6 (OMIM: 614466). Inactivating *MMP3* in mice increases atherosclerotic plaque accumulation while reducing aneurysm [21].

High-density lipoprotein [22] and lipid digestion, mobilization and transport pathway

(58 genes, $\log_{10}\mathrm{BF} = 89.8$)

- *CUBN* (baseline P_1: 0.24; enrichment P_1: 1.00)
  CUBN encodes a receptor for intrinsic factor-vitamin B12 complexes (cubilin) that maintains blood levels of HDL [23]. Mutations in *CUBN* cause a form of congenital megaloblastic anemia due to vitamin B12 deficiency (OMIM: 261100). *CUBN* is near a GWAS hit of total cholesterol (rs10904908, $p = 3.0 \times 10^{-11}$ [24]).
- *ABCG1* (baseline P_1: 0.01; enrichment P_1: 0.89)
  ABCG1 encodes an ATP-binding cassette transporter that plays a critical role in mediating efflux of cellular cholesterol to HDL [25].

Rheumatoid arthritis [26] and lymphocyte NFAT-dependent transcription pathway (45 genes, $\log_{10}\mathrm{BF} = 10.0$)

- *PTGS2* (a.k.a. *COX2*; baseline P_1: 0.74; enrichment P_1: 0.98)
  PTGS2-specific inhibitors have shown efficacy in reducing joint inflammation in both mouse models [27] and clinical trials [28]. *PTGS2* is near a GWAS hit of Crohn's disease (rs10798069, $p = 4.3 \times 10^{-9}$ [11])
- *PPARG* (baseline P_1: 0.28; enrichment P_1: 0.98)
  PPARG has important roles in regulating inflammatory and immune responses with potential applications in treating chronic inflammatory diseases including RA [29, 30].

1.11. *Expression patterns of APOE and TTR across human tissues.*. Here we provide links to public data browsers for viewing the expression patterns of *APOE* and *TTR* across human tissues.

The cross-tissue expression pattern of *APOE* is publicly available at

- GeneAtlas microarray data [31]: `http://biogps.org/#goto=genereport&id=348`;
- NCBI RNA-seq data [32, 33]: `https://www.ncbi.nlm.nih.gov/gene/348`;
- GTEx RNA-seq data [34]: `http://www.gtexportal.org/home/gene/APOE`.

The cross-tissue expression pattern of *TTR* is publicly available at

- GeneAtlas microarray data [31]: `http://biogps.org/#goto=genereport&id=7276`;
- NCBI RNA-seq data [32, 33]: `https://www.ncbi.nlm.nih.gov/gene/7276`;
- GTEx RNA-seq data [34]: `http://www.gtexportal.org/home/gene/TTR`.

1.12. *Acknowledgments and data sources.* We thank all the GWAS consortia for making their summary statistics publicly available. We also thank the GTEx consortium for making their RNA-sequencing data publicly available. Below is a full list of acknowledgments and links to data sources. We verified that all links below were valid on September 13, 2018.

- **Genetic Investigation of ANthropometric Traits (GIANT) Consortium**
  Data on adult human height [5], body mass index [35] and body fat distribution [36] have been contributed by GIANT investigators and have been downloaded from `http://portals.broadinstitute.org/collaboration/giant`.

- **Psychiatric Genomics Consortium (PGC)**
  Data on schizophrenia [37] have been contributed by PGC investigators and have been downloaded from `http://www.med.unc.edu/pgc`.

- **International Inflammatory Bowel Disease Genetics Consortium (IIBDGC)**
  Data on inflammatory bowel disease [11], including Crohn's disease and ulcerative colitis have been contributed by IIBDGC investigators and have been downloaded from https://www.ibdgenetics.org.

- **Coronary ARtery DIsease Genome wide Replication and Meta-analysis (CARDIoGRAM) plus The Coronary Artery Disease (C4D) Genetics (CARDIoGRAMplusC4D) Consortium**
  Data on coronary artery disease and myocardial infarction [18] have been contributed by CARDIoGRAMplusC4D investigators and downloaded from http://www.cardiogramplusc4d.org/.

- **GWAS summary statistics of heart rate [38]**
  Data on heart rate have been contributed by authors of [38] and have been downloaded from https://walker05.u.hpc.mssm.edu/.

- **International Genomics of Alzheimer's Project (IGAP)**
  Data on Alzheimer's disease [39] have been contributed by IGAP investigators and have been downloaded from http://web.pasteur-lille.fr/en/recherche/u744/igap/igap_download.php. We thank the International Genomics of Alzheimer's Project (IGAP) for providing summary results data for these analyses. The investigators within IGAP contributed to the design and implementation of IGAP and/or provided data but did not participate in analysis or writing of this report. IGAP was made possible by the generous participation of the control subjects, the patients, and their families. The i-Select chips was funded by the French National Foundation on Alzheimer's disease and related disorders. EADI was supported by the LABEX (laboratory of excellence program investment for the future) DISTALZ grant, Inserm, Institut Pasteur de Lille, Université de Lille 2 and the Lille University Hospital. GERAD was supported by the Medical Research Council (Grant no. 503480), Alzheimer's Research UK (Grant no. 503176), the Wellcome Trust (Grant no. 082604/2/07/Z) and German Federal Ministry of Education and Research (BMBF): Competence Network Dementia (CND) grant no. 01GI0102, 01GI0711, 01GI0420. CHARGE was partly supported by the NIH/NIA grant R01 AG033193 and the NIA AG081220 and AGES contract N01-AG-12100, the NHLBI grant R01 HL105756, the Icelandic Heart Association, and the Erasmus Medical Center and Erasmus University. ADGC was supported by the NIH/NIA grants: U01 AG032984, U24 AG021886, U01 AG016976, and the Alzheimer's Association grant ADGC-10-196728.

- **Social Science Genetic Association Consortium (SSGAC)**
  Data on neuroticism and depressive symptoms [40] have been contributed by SSGAC investigators and have been downloaded from https://www.thessgac.org. For financial support, the SSGAC thanks the U.S. National Science Foundation, the U.S. National Institutes of Health (National Institute on Aging, and the Office for Behavioral and Social Science Research), the Ragnar Söderberg Foundation, the Swedish Research Council, The Jan Wallander and Tom Hedelius Foundation, the European Research Council, and the Pershing Square Fund of the Foundations of Human Behavior.

- **GWAS summary statistics of rheumatoid arthritis [26]**
  Data on rheumatoid arthritis have been contributed by authors of [26] and have been downloaded from http://plaza.umin.ac.jp/yokada/datasource/software.htm.

- **DIAbetes Genetics Replication And Meta-analysis (DIAGRAM) Consortium**
  Data on type 2 diabetes [41] have been contributed by DIAGRAM investigators and have been downloaded from `http://www.diagram-consortium.org`.

- **Reproductive Genetics (ReproGen) Consortium**
  Data on age at natural menopause [42] have been contributed by ReproGen investigators and have been downloaded from `http://www.reprogen.org`.

- **Global Urate Genetics Consortium (GUGC)**
  Data on serum urate concentrations and gout [43] have been contributed by GUGC investigators and have been downloaded from `http://metabolomics.helmholtz-muenchen.de/gugc`.

- **Global Lipids Genetics Consortium (GLGC)**
  Data on blood lipids [22], including levels of total cholesterol, low-density lipoprotein cholesterol, high-density lipoprotein cholesterol and triglycerides have been contributed by GLGC investigators and have been downloaded from `http://csg.sph.umich.edu//abecasis/public/lipids2010`.

- **Glucose and Insulin-related traits Consortium (MAGIC)**
  Data on glycaemic traits [44], including fasting glucose and insulin results accounting for body mass index, have been contributed by MAGIC investigators and have been downloaded from `https://www.magicinvestigators.org`.

- **Project MinE**
  Data on amyotrophic lateral sclerosis [45] have been contributed by Project MinE and have been downloaded from `http://databrowser.projectmine.com`.

- **GWAS summary statistics of red blood cell phenotypes [46]**
  Data on six red blood cell phenotypes have been contributed by authors of [46] and have been downloaded from the European Genome-Phenome Archive (EGA, `http://www.ebi.ac.uk/ega`) under accession number EGAS00000000132.

- **Genotype-Tissue Expression (GTEx) Project**
  The GTEx Project was supported by the Common Fund of the Office of the Director of the National Institutes of Health, and by NCI, NHGRI, NHLBI, NIDA, NIMH, and NINDS. The data used for the analyses described in this manuscript were obtained from the GTEx Portal (`https://gtexportal.org`) on November 21, 2016.

2. Supplementary Table 1. Sample sizes and numbers of genetic variants in GWAS of 31 human phenotypes. For each phenotype, the number of "total" SNPs is the number of SNPs reported in the corresponding publication and/or summary statistics file, and the number of "analyzed" SNPs is the number of SNPs used in this study. Both columns are visualized in **Supplementary Figure 8**.

		Number of SNPs		Sample size
Phenotype (abbreviation)	PMID	Total	Analyzed	(cases+controls)
**Neurological phenotypes**				
Amyotrophic lateral sclerosis (ALS)	27455348	8,709,433	1,162,845	12,577+23,475
Depressive symptoms (DS)	27089181	6,524,474	1,119,108	161,460
Alzheimer's disease (LOAD)	24162737	7,055,881	1,136,997	17,008+37,154
Neuroticism (NEU)	27089181	6,524,432	1,119,108	170,911
Schizophrenia (SCZ)	25056061	9,444,230	1,113,442	152,805
**Anthropometric traits**				
Body mass index (BMI)	25673413	2,554,637	1,012,465	234,069
Height (HEIGHT)	25282103	2,550,858	1,064,575	253,288
Waist-to-hip ratio (WHR)	25673412	2,542,431	1,008,898	142,762
**Immune-related traits**				
Crohn's disease (CD)	26192919	12,276,505	1,064,533	5,956+14,927
Inflammatory bowel disease (IBD)	26192919	12,716,083	1,081,481	12,882+21,770
Rheumatoid arthritis (RA)	24390342	8,747,962	1,158,064	14,361+43,923
Ulcerative colitis (UC)	26192919	12,255,196	1,092,170	6,968+20,464
**Metabolic phenotypes**				
Age at natural menopause (ANM)	26414677	2,418,695	1,047,412	69,360
Coronary artery disease (CAD)	26343387	9,455,778	1,121,322	60,801+123,504
Fasting glucose (FG)	22581228	2,628,879	1,114,610	58,074
Fasting insulin (FI)	22581228	2,627,848	1,114,592	51,750
Gout (GOUT)	23263486	2,538,056	1,061,037	2,115+67,259
High-density lipoprotein (HDL)	20686565	2,692,429	1,032,214	99,900
Heart rate (HR)	23583979	2,516,789	1,066,168	92,355
Low-density lipoprotein (LDL)	20686565	2,692,564	1,030,397	95,454
Myocardial infarction (MI)	26343387	9,289,491	1,111,568	42,561+123,504
Type 2 diabetes (T2D)	22885922	2,473,441	1,047,618	12,171+56,862
Total cholesterol (TC)	20686565	2,692,413	1,032,272	100,184
Triglycerides (TG)	20686565	2,692,560	1,030,671	96,598
Serum urate (URATE)	23263486	2,450,547	1,050,253	110,347
**Hematopoietic traits**				
Haemoglobin (HB)	23222517	2,593,078	1,116,281	61,155
Mean cell HB (MCH)	23222517	2,586,784	1,114,901	51,711
Mean cell HB concentration (MCHC)	23222517	2,588,875	1,115,595	56,475
Mean cell volume (MCV)	23222517	2,591,132	1,116,066	58,114
Packed cell volume (PCV)	23222517	2,591,079	1,115,725	53,089
Red blood cell count (RBC)	23222517	2,589,454	1,115,397	53,661

3. Supplementary Table 2. Posterior statistics displayed in **Figure 4(a)** and **Supplementary Figure 9**. Each entry below corresponds to a point range shown in **Figure 4(a)** and **Supplementary Figure 9**. Note that "Round 2" results in **Supplementary Figure 9** are the same as **Figure 4(a)**. For each trait, "Round 1" results are based on a wide and coarse grid of hyper-parameters $\{h, \theta_0\}$, and "Round 2" results are based on a narrow and fine grid of $\{h, \theta_0\}$ (informed by Round 1 results). See **Supplementary Table 6** for details of grids.

	Fraction of trait-associated SNPs		Standardized effect size of trait-associated SNPs	
Trait	Round 1	Round 2	Round 1	Round 2
ALS	1.39e-06, [9.90e-07, 2.42e-06]	1.43e-06, [9.90e-07, 2.27e-06]	6.15e-02, [5.14e-02, 6.75e-02]	6.09e-02, [5.23e-02, 6.75e-02]
ANM	6.41e-05, [6.03e-05, 6.41e-05]	5.96e-05, [5.81e-05, 5.96e-05]	1.28e+00, [1.28e+00, 1.30e+00]	1.38e+00, [1.38e+00, 1.41e+00]
BMI	7.13e-05, [6.77e-05, 7.14e-05]	7.06e-05, [5.87e-05, 8.18e-05]	1.89e-02, [1.89e-02, 1.93e-02]	1.90e-02, [1.79e-02, 2.02e-02]
CAD	7.32e-05, [7.32e-05, 9.18e-05]	8.06e-05, [7.08e-05, 9.51e-05]	2.15e-02, [1.98e-02, 2.15e-02]	2.08e-02, [1.95e-02, 2.18e-02]
CD	9.67e-04, [9.67e-04, 9.67e-04]	9.67e-04, [9.67e-04, 1.02e-03]	2.07e-02, [2.07e-02, 2.07e-02]	2.07e-02, [1.99e-02, 2.07e-02]
DS	2.34e-06, [1.05e-06, 4.42e-06]	2.35e-06, [1.05e-06, 3.79e-06]	2.22e-02, [1.96e-02, 2.36e-02]	2.22e-02, [2.02e-02, 2.36e-02]
FG	4.25e-05, [4.25e-05, 4.25e-05]	3.80e-05, [3.80e-05, 3.80e-05]	2.76e+00, [2.76e+00, 2.76e+00]	3.40e+00, [3.40e+00, 3.40e+00]
FI	5.62e-06, [5.62e-06, 5.66e-06]	5.25e-06, [5.21e-06, 5.37e-06]	2.05e+00, [2.03e+00, 2.05e+00]	2.21e+00, [2.16e+00, 2.23e+00]
GOUT	4.38e-06, [2.46e-06, 5.96e-06]	4.56e-06, [2.46e-06, 7.16e-06]	5.11e-02, [4.35e-02, 6.38e-02]	5.00e-02, [3.90e-02, 5.92e-02]
HB	1.41e-05, [1.41e-05, 1.41e-05]	1.26e-05, [1.25e-05, 1.28e-05]	1.67e+00, [1.66e+00, 1.67e+00]	1.86e+00, [1.83e+00, 1.88e+00]
HDL	2.19e-04, [2.05e-04, 2.19e-04]	1.95e-04, [1.93e-04, 2.09e-04]	2.75e-02, [2.75e-02, 2.91e-02]	2.98e-02, [2.84e-02, 2.99e-02]
HEIGHT	9.93e-03, [9.42e-03, 9.93e-03]	1.08e-02, [9.93e-03, 1.09e-02]	7.00e-03, [7.00e-03, 7.71e-03]	6.58e-03, [6.53e-03, 7.00e-03]
HR	3.24e-05, [2.51e-05, 3.61e-05]	3.17e-05, [2.51e-05, 3.91e-05]	3.34e-02, [3.20e-02, 3.61e-02]	3.35e-02, [3.11e-02, 3.61e-02]
IBD	9.29e-04, [9.29e-04, 1.43e-03]	1.20e-03, [1.17e-03, 1.39e-03]	1.93e-02, [1.77e-02, 1.93e-02]	1.69e-02, [1.57e-02, 1.71e-02]
LDL	1.55e-04, [1.55e-04, 1.92e-04]	1.92e-04, [1.91e-04, 1.99e-04]	4.21e-02, [3.79e-02, 4.21e-02]	3.85e-02, [3.76e-02, 3.85e-02]
LOAD	2.99e-05, [2.73e-05, 3.00e-05]	2.78e-05, [2.78e-05, 2.83e-05]	2.76e-01, [2.75e-01, 3.01e-01]	2.97e-01, [2.91e-01, 2.98e-01]
MCH	1.27e-04, [1.27e-04, 1.27e-04]	9.30e-05, [9.30e-05, 9.30e-05]	2.44e-01, [2.44e-01, 2.44e-01]	3.20e-01, [3.20e-01, 3.20e-01]
MCHC	1.12e-06, [5.37e-07, 2.40e-06]	1.21e-06, [5.37e-07, 2.40e-06]	9.99e-02, [9.86e-02, 1.01e-01]	1.00e-01, [9.86e-02, 1.02e-01]
MCV	1.33e-04, [1.10e-04, 1.33e-04]	1.13e-04, [1.13e-04, 1.13e-04]	3.04e-01, [3.04e-01, 3.16e-01]	3.83e-01, [3.83e-01, 3.83e-01]
MI	3.97e-05, [3.97e-05, 4.90e-05]	4.18e-05, [3.39e-05, 5.07e-05]	2.30e-02, [2.30e-02, 2.34e-02]	2.26e-02, [2.11e-02, 2.42e-02]
NEU	5.94e-05, [4.37e-05, 6.75e-05]	5.38e-05, [3.45e-05, 6.75e-05]	1.54e-02, [1.50e-02, 1.62e-02]	1.57e-02, [1.50e-02, 1.68e-02]
PCV	1.47e-05, [1.47e-05, 1.49e-05]	1.32e-05, [1.32e-05, 1.33e-05]	7.62e-01, [7.35e-01, 7.62e-01]	8.90e-01, [8.79e-01, 8.90e-01]
RA	4.21e-04, [4.21e-04, 6.26e-04]	4.73e-04, [4.72e-04, 5.00e-04]	1.77e-02, [1.64e-02, 1.77e-02]	1.70e-02, [1.66e-02, 1.70e-02]
RBC	2.20e-04, [2.01e-04, 2.45e-04]	1.29e-04, [1.29e-04, 1.36e-04]	3.46e-02, [3.09e-02, 3.76e-02]	4.99e-02, [4.81e-02, 4.99e-02]
SCZ	8.60e-03, [8.11e-03, 8.62e-03]	6.31e-03, [6.12e-03, 7.26e-03]	5.15e-03, [5.14e-03, 5.69e-03]	6.15e-03, [5.68e-03, 6.25e-03]
T2D	2.84e-05, [2.81e-05, 3.00e-05]	2.51e-05, [2.14e-05, 2.97e-05]	9.26e-02, [8.83e-02, 9.36e-02]	1.03e-01, [8.99e-02, 1.16e-01]
TC	8.98e-05, [8.98e-05, 9.05e-05]	9.38e-05, [9.11e-05, 9.67e-05]	1.31e-01, [1.30e-01, 1.31e-01]	1.26e-01, [1.22e-01, 1.29e-01]
TG	4.40e-05, [4.40e-05, 4.40e-05]	4.35e-05, [4.34e-05, 4.37e-05]	9.34e-01, [9.34e-01, 9.34e-01]	9.56e-01, [9.53e-01, 9.57e-01]
UC	8.92e-04, [8.91e-04, 1.41e-03]	8.76e-04, [6.23e-04, 1.07e-03]	1.79e-02, [1.68e-02, 1.79e-02]	1.81e-02, [1.50e-02, 1.89e-02]
URATE	9.35e-05, [9.35e-05, 9.35e-05]	9.29e-05, [9.29e-05, 9.29e-05]	7.07e-01, [7.07e-01, 7.07e-01]	6.46e-01, [6.46e-01, 6.46e-01]
WHR	4.94e-04, [4.94e-04, 4.94e-04]	5.32e-04, [4.94e-04, 5.32e-04]	1.38e-02, [1.38e-02, 1.38e-02]	1.39e-02, [1.38e-02, 1.39e-02]

4. Supplementary Table 3. Estimated pairwise sharing of pathway enrichments between two traits. Each entry below corresponds to a cell displayed in **Figure 4(b)**.

	ALS	ANM	BMI	CAD	CD	DS	FG	FI	GOUT	HB	HDL	HEIGHT	HR	IBD	LDL
ALS	1	0.00	0.00	0.00	0.00	0.00	0.00	0.00	0.00	0.00	0.00	0.00	0.00	0.00	0.00
ANM	0	1.00	0.07	0.11	0.45	0.08	0.76	0.60	0.02	0.78	0.24	0.95	0.02	0.46	0.36
BMI	0	0.07	1.00	0.03	0.02	0.24	0.11	0.15	0.01	0.12	0.04	0.07	0.01	0.02	0.04
CAD	0	0.11	0.03	1.00	0.17	0.01	0.08	0.09	0.02	0.11	0.12	0.11	0.01	0.16	0.27
CD	0	0.45	0.02	0.17	1.00	0.01	0.35	0.28	0.03	0.46	0.17	0.44	0.03	1.00	0.41
DS	0	0.08	0.24	0.01	0.01	1.00	0.10	0.13	0.07	0.12	0.04	0.03	0.06	0.01	0.03
FG	0	0.76	0.11	0.08	0.35	0.10	1.00	0.76	0.03	0.74	0.24	0.79	0.02	0.31	0.30
FI	0	0.60	0.15	0.09	0.28	0.13	0.76	1.00	0.04	0.64	0.13	0.54	0.02	0.26	0.19
GOUT	0	0.02	0.01	0.02	0.03	0.07	0.03	0.04	1.00	0.02	0.02	0.02	0.00	0.03	0.02
HB	0	0.78	0.12	0.11	0.46	0.12	0.74	0.64	0.02	1.00	0.23	0.68	0.02	0.45	0.36
HDL	0	0.24	0.04	0.12	0.17	0.04	0.24	0.13	0.02	0.23	1.00	0.23	0.02	0.15	0.45
HEIGHT	0	0.95	0.07	0.11	0.44	0.03	0.79	0.54	0.02	0.68	0.23	1.00	0.01	0.45	0.36
HR	0	0.02	0.01	0.01	0.03	0.06	0.02	0.02	0.00	0.02	0.02	0.01	1.00	0.02	0.01
IBD	0	0.46	0.02	0.16	1.00	0.01	0.31	0.26	0.03	0.45	0.15	0.45	0.02	1.00	0.34
LDL	0	0.36	0.04	0.27	0.41	0.03	0.30	0.19	0.02	0.36	0.45	0.36	0.01	0.34	1.00
LOAD	0	0.11	0.02	0.21	0.13	0.02	0.09	0.10	0.00	0.09	0.18	0.11	0.00	0.12	0.23
MCH	0	0.56	0.10	0.11	0.31	0.10	0.57	0.49	0.02	0.55	0.18	0.55	0.02	0.32	0.27
MCHC	0	0.44	0.17	0.09	0.15	0.15	0.63	0.80	0.04	0.56	0.07	0.47	0.02	0.18	0.17
MCV	0	0.71	0.09	0.11	0.43	0.07	0.68	0.54	0.01	0.69	0.18	0.70	0.01	0.43	0.38
MI	0	0.04	0.03	0.92	0.05	0.01	0.04	0.05	0.01	0.04	0.07	0.03	0.00	0.05	0.08
NEU	0	0.02	0.10	0.00	0.00	0.33	0.03	0.04	0.00	0.04	0.06	0.02	0.00	0.00	0.03
PCV	0	0.59	0.09	0.10	0.47	0.09	0.48	0.43	0.03	0.82	0.20	0.54	0.02	0.46	0.36
RA	0	0.12	0.02	0.12	0.33	0.01	0.07	0.07	0.06	0.14	0.08	0.12	0.00	0.34	0.16
RBC	0	0.61	0.15	0.09	0.29	0.14	0.70	0.71	0.03	0.78	0.14	0.56	0.03	0.29	0.22
SCZ	0	0.00	0.00	0.00	0.00	0.00	0.00	0.00	0.00	0.00	0.00	0.00	0.00	0.00	0.00
T2D	0	0.19	0.13	0.07	0.13	0.12	0.19	0.17	0.03	0.18	0.13	0.16	0.03	0.14	0.13
TC	0	0.69	0.05	0.19	0.60	0.03	0.53	0.33	0.02	0.66	0.37	0.67	0.01	0.53	0.96
TG	0	0.40	0.09	0.17	0.20	0.07	0.35	0.26	0.02	0.37	0.49	0.40	0.03	0.18	0.48
UC	0	0.19	0.02	0.13	0.68	0.01	0.12	0.14	0.04	0.21	0.06	0.19	0.03	0.92	0.16
URATE	0	0.56	0.06	0.12	0.63	0.04	0.46	0.36	0.05	0.65	0.19	0.55	0.02	0.63	0.37
WHR	0	0.09	0.08	0.05	0.06	0.06	0.07	0.08	0.02	0.07	0.06	0.09	0.00	0.08	0.11

	LOAD	MCH	MCHC	MCV	MI	NEU	PCV	RA	RBC	SCZ	T2D	TC	TG	UC	URATE	WHR
ALS	0.00	0.00	0.00	0.00	0.00	0.00	0.00	0.00	0.00	0	0.00	0.00	0.00	0.00	0.00	0.00
ANM	0.11	0.56	0.44	0.71	0.04	0.02	0.59	0.12	0.61	0	0.19	0.69	0.40	0.19	0.56	0.09
BMI	0.02	0.10	0.17	0.09	0.03	0.10	0.09	0.02	0.15	0	0.13	0.05	0.09	0.02	0.06	0.08
CAD	0.21	0.11	0.09	0.11	0.92	0.00	0.10	0.12	0.09	0	0.07	0.19	0.17	0.13	0.12	0.05
CD	0.13	0.31	0.15	0.43	0.05	0.00	0.47	0.33	0.29	0	0.13	0.60	0.20	0.68	0.63	0.06
DS	0.02	0.10	0.15	0.07	0.01	0.33	0.09	0.01	0.14	0	0.12	0.03	0.07	0.01	0.04	0.06
FG	0.09	0.57	0.63	0.68	0.04	0.03	0.48	0.07	0.70	0	0.19	0.53	0.35	0.12	0.46	0.07
FI	0.10	0.49	0.80	0.54	0.05	0.04	0.43	0.07	0.71	0	0.17	0.33	0.26	0.14	0.36	0.08
GOUT	0.00	0.02	0.04	0.01	0.01	0.00	0.03	0.06	0.03	0	0.03	0.02	0.02	0.04	0.05	0.02
HB	0.09	0.55	0.56	0.69	0.04	0.04	0.82	0.14	0.78	0	0.18	0.66	0.37	0.21	0.65	0.07
HDL	0.18	0.18	0.07	0.18	0.07	0.06	0.20	0.08	0.14	0	0.13	0.37	0.49	0.06	0.19	0.06
HEIGHT	0.11	0.55	0.47	0.70	0.03	0.02	0.54	0.12	0.56	0	0.16	0.67	0.40	0.19	0.55	0.09
HR	0.00	0.02	0.02	0.01	0.00	0.00	0.02	0.00	0.03	0	0.03	0.01	0.03	0.03	0.02	0.00
IBD	0.12	0.32	0.18	0.43	0.05	0.00	0.46	0.34	0.29	0	0.14	0.53	0.18	0.92	0.63	0.08
LDL	0.23	0.27	0.17	0.38	0.08	0.03	0.36	0.16	0.22	0	0.13	0.96	0.48	0.16	0.37	0.11
LOAD	1.00	0.10	0.07	0.11	0.15	0.03	0.09	0.08	0.09	0	0.06	0.17	0.21	0.10	0.13	0.03
MCH	0.10	1.00	0.47	0.78	0.04	0.03	0.47	0.09	0.59	0	0.14	0.42	0.32	0.14	0.39	0.07
MCHC	0.07	0.47	1.00	0.50	0.05	0.04	0.39	0.05	0.63	0	0.15	0.26	0.19	0.10	0.29	0.08
MCV	0.11	0.78	0.50	1.00	0.06	0.02	0.52	0.10	0.65	0	0.14	0.58	0.33	0.18	0.49	0.06
MI	0.15	0.04	0.05	0.06	1.00	0.01	0.04	0.02	0.06	0	0.05	0.05	0.05	0.03	0.04	0.02
NEU	0.03	0.03	0.04	0.02	0.01	1.00	0.02	0.00	0.04	0	0.03	0.03	0.00	0.00	0.01	0.03
PCV	0.09	0.47	0.39	0.52	0.04	0.02	1.00	0.16	0.51	0	0.14	0.50	0.25	0.27	0.55	0.09
RA	0.08	0.09	0.05	0.10	0.02	0.00	0.16	1.00	0.08	0	0.05	0.18	0.06	0.76	0.19	0.01
RBC	0.09	0.59	0.63	0.65	0.06	0.04	0.51	0.08	1.00	0	0.15	0.37	0.28	0.12	0.33	0.07
SCZ	0.00	0.00	0.00	0.00	0.00	0.00	0.00	0.00	0.00	1	0.00	0.00	0.00	0.00	0.00	0.00
T2D	0.06	0.14	0.15	0.14	0.05	0.03	0.14	0.05	0.15	0	1.00	0.15	0.15	0.10	0.16	0.07
TC	0.17	0.42	0.26	0.58	0.05	0.03	0.50	0.18	0.37	0	0.15	1.00	0.63	0.22	0.67	0.09
TG	0.21	0.32	0.19	0.33	0.05	0.00	0.25	0.06	0.28	0	0.15	0.63	1.00	0.08	0.57	0.08
UC	0.10	0.14	0.10	0.18	0.03	0.00	0.27	0.76	0.12	0	0.10	0.22	0.08	1.00	0.38	0.08
URATE	0.13	0.39	0.29	0.49	0.04	0.01	0.55	0.19	0.33	0	0.16	0.67	0.57	0.38	1.00	0.08
WHR	0.03	0.07	0.08	0.06	0.02	0.03	0.09	0.01	0.07	0	0.07	0.09	0.08	0.08	0.08	1.00

5. Supplementary Table 4. All SNP information and genomic positions in the following tables are based on Genome Reference Consortium GRCh37.

(a) Gene-level posterior statistics displayed in **Figure 5(b)**. For each dot (gene) shown in **Figure 5(b)**, its coordinates on x-axis and y-axis are determined by corresponding values in columns "Baseline P_1" and "Enrichment P_1" respectively. The size of each dot (gene) is determined by the physical distance between the gene and the nearest GWAS hit (if any), in base pairs (bp), which is shown in column "Distance (bp)". The "NA" values in column "Distance (bp)" indicate that there is no GWAS hit on the same chromosome as the gene.

Gene	Chr.	Start	End	Baseline P_1	Enrichment P_1	Distance (bp)	Nearest hit
APOB	2	21224301	21266945	1.0000000	1.0000000	0	rs1367117
APOC2	19	45449239	45452822	1.0000000	1.0000000	26293	rs4420638
APOE	19	45409039	45412650	1.0000000	1.0000000	10296	rs4420638
LDLR	19	11200038	11244506	1.0000000	1.0000000	0	rs6511720
APOA5	11	116660086	116663136	1.0000000	1.0000000	11169	rs964184
APOC3	11	116700624	116703787	0.9998878	1.0000000	51707	rs964184
APOA4	11	116691418	116694011	0.9998877	1.0000000	42501	rs964184
APOA1	11	116706469	116708338	0.7034787	0.9995003	57552	rs964184
MTTP	4	100485240	100545154	0.1415537	0.9923219	NA	none
LIPC	15	58724175	58861073	0.0163921	0.9589340	NA	none
SDC1	2	20400558	20425194	0.0231079	0.9153864	838706	rs1367117
LDLRAP1	1	25870076	25895377	0.0419188	0.8828886	94343	rs12027135
LPL	8	19796582	19824770	0.0066880	0.7603627	10611436	rs2126259
HSPG2	1	22148737	22263750	0.0052681	0.6798969	3511983	rs12027135
APOA2	1	161192083	161193418	0.0033433	0.5842822	51373777	rs629301
SAR1B	5	133936839	133968533	0.0027561	0.4542058	22421764	rs6882076
P4HB	17	79801034	79818544	0.0000308	0.0081494	34409230	rs7225700

(b) SNP-level posterior statistics related to **Figure 5(b)**. To assess SNP-level associations, we use Posterior Inclusion Probability (PIP), which is the posterior probability that a SNP has nonzero effect on the phenotype. Shown below are the SNPs with baseline PIP less than 0.5 and enrichment PIP greater than 0.5.

SNP	Chr.	Position (bp)	Baseline PIP	Enrichment PIP
rs1713222	2	21271323	0.0000461	1.0000000
rs7026	19	45324516	0.0008064	1.0000000
rs12981050	19	11200412	0.0000854	1.0000000
rs2304181	19	11238975	0.0085668	1.0000000
rs4803760	19	45333834	0.0014801	1.0000000
rs562338	2	21288321	0.0000321	0.9999706
rs439401	19	45414451	0.0000797	0.9990809
rs2075650	19	45395619	0.0001276	0.9989899
rs892114	19	11266584	0.0004527	0.9904336
rs1942478	11	116651463	0.0000614	0.9894956
rs2228671	19	11210912	0.0019893	0.9837481
rs5929	19	11226800	0.0002287	0.9823478
rs3826810	19	11242133	0.0001926	0.9819899
rs5930	19	11224265	0.0000598	0.9819222
rs673548	2	21237544	0.0008667	0.9817970
rs10030937	4	100473723	0.0215764	0.9799749
rs2163839	19	11256982	0.0068029	0.9799565
rs3935557	2	21141725	0.0116221	0.9796790
rs12983316	19	11114352	0.0008380	0.9203831
rs540156	2	21295065	0.0000716	0.8551321
rs12096438	1	25889422	0.0024555	0.8121214
rs2889490	19	45550407	0.0002372	0.8061891
rs5110	11	116691634	0.0168215	0.7588280

6. Supplementary Table 5. Confounding adjustment in GWAS of 31 human phenotypes. Columns left to right: (1) phenotype and its abbreviation; (2) genomic control (GC) factor [47]; (3) LD score (LDSC) regression intercept [48]; (4) the number of top genotype-derived principal components (PCs) that were included as covariates in the single-SNP association testing [49]; (5) other covariates included in the single-SNP association testing. The genomic control factor λ_{GC} and the LD score regression intercept λ_{LDSC} are two measures of confounding biases such as population stratification. Values of $\lambda_{GC} \approx 1$ or $\lambda_{LDSC} \approx 1$ indicate little confounding effects, whereas $\lambda_{GC} \geq 1$ or $\lambda_{LDSC} \geq 1$ suggest possible existence of confounding biases. The "cohort" covariate denotes all factors that are specific to study cohorts (e.g. genotyping array, study site).

Phenotype (abbreviation)	λ_{GC}	λ_{LDSC}	# of PCs	Other covariates
**Neurological phenotypes**				
Amyotrophic lateral sclerosis (ALS)	1.12	1.10	1-4	not shown
Depressive symptoms (DS)	1.17	1.01	4-15	sex, age, cohort
Alzheimer's disease (LOAD)	1.09	1.04	2-8	sex, age
Neuroticism (NEU)	1.32	1.00	4-15	sex, age, cohort
Schizophrenia (SCZ)	1.47	1.07	10	not shown
**Anthropometric traits**				
Body mass index (BMI)	1.08	1.02	not shown	sex, age, cohort
Height (HEIGHT)	1.94	1.05	not shown	sex, age, cohort
Waist-to-hip ratio (WHR)	1.01	0.93	not shown	sex, age, cohort, BMI
**Immune-related traits**				
Crohn's disease (CD)	1.13	1.03	10	not shown
Inflammatory bowel disease (IBD)	1.16	1.06	15	not shown
Rheumatoid arthritis (RA)	1.07	0.98	5-10	not shown
Ulcerative colitis (UC)	1.11	1.04	7	not shown
**Metabolic phenotypes**				
Age at natural menopause (ANM)	not shown	not shown	not shown	cohort
Coronary artery disease (CAD)	1.18	1.05	not shown	not shown
Fasting glucose (FG)	not shown	not shown	not shown	sex, age, cohort, BMI
Fasting insulin (FI)	1.07	1.02	not shown	sex, age, cohort, BMI
Gout (GOUT)	1.03	not shown	2-10	sex, age, cohort
High-density lipoprotein cholesterol (HDL)	1.14	1.01	not shown	sex, age, cohort
Heart rate (HR)	1.11	1.01	not shown	sex, age, cohort, BMI
Low-density lipoprotein cholesterol (LDL)	1.10	1.00	not shown	sex, age, cohort
Myocardial infarction (MI)	not shown	not shown	not shown	not shown
Type 2 diabetes (T2D)	1.10	1.03	not shown	cohort
Total cholesterol (TC)	1.11	1.01	not shown	sex, age, cohort
Triglycerides (TG)	1.12	1.00	not shown	sex, age, cohort
Serum urate (URATE)	1.12	1.01	2-10	sex, age, cohort
**Hematopoietic traits**				
Haemoglobin (HB)	1.10	not shown	2-10	sex, age, cohort
Mean cell HB (MCH)	1.13	not shown	2-10	sex, age, cohort
Mean cell HB concentration (MCHC)	1.08	not shown	2-10	sex, age, cohort
Mean cell volume (MCV)	1.14	not shown	2-10	sex, age, cohort
Packed cell volume (PCV)	1.10	not shown	2-10	sex, age, cohort
Red blood cell count (RBC)	1.14	not shown	2-10	sex, age, cohort

7. Supplementary Table 6. Grids of hyper-parameters used in genome-wide multiple-SNP analyses of 31 human phenotypes, assuming no pathways are enriched (i.e. the baseline model $M_0 : \theta = 0$). Here `(j:i:k)` denotes a regularly-spaced vector that starts at `j`, uses `i` as the increment between elements, and (roughly) stops at `k`.

	Round 1 analysis		Round 2 analysis	
Phenotype (abbreviation)	h	θ_0	h	θ_0
**Neurological phenotypes**				
Amyotrophic lateral sclerosis (ALS)	(0.3:0.1:0.6)	(-6:0.25:-3)	(0.3:0.1:0.6)	(-6:0.05:-5)
Depressive symptoms (DS)	(0.3:0.1:0.6)	(-6:0.25:-2)	(0.3:0.1:0.6)	(-6:0.05:-5)
Alzheimer's disease (LOAD)	(0.3:0.1:0.6)	(-5.25:0.25:-3.25)	0.3	(-5.25:0.025:-4.75)
Neuroticism (NEU)	(0.3:0.1:0.6)	(-4.5:0.25:-2)	0.3	(-4.5:0.025:-4)
Schizophrenia (SCZ)	(0.3:0.1:0.6)	(-4:0.25:-1)	0.3	(-2.25:0.025:-1.75)
**Anthropometric traits**				
Body mass index (BMI)	(0.3:0.1:0.6)	(-5:0.25:-1)	0.3	(-4.25:0.025:-3.75)
Height (HEIGHT)	(0.3:0.1:0.6)	(-4:0.25:-1)	(0.3:0.1:0.4)	(-2.25:0.025:-1.75)
Waist-to-hip ratio (WHR)	(0.3:0.1:0.6)	(-6:0.25:-3)	(0.3:0.1:0.6)	(-6:0.05:-5)
**Immune-related traits**				
Crohn's disease (CD)	(0.3:0.1:0.6)	(-4:0.25:-2)	0.3	(-3.25:0.025:-2.75)
Inflammatory bowel disease (IBD)	(0.3:0.1:0.6)	(-4:0.25:-2)	0.3	(-3.25:0.025:-2.75)
Rheumatoid arthritis (RA)	(0.3:0.1:0.6)	(-4.5:0.25:-2)	0.3	(-3.5:0.025:-3)
Ulcerative colitis (UC)	(0.3:0.1:0.6)	(-4:0.25:-2)	0.3	(-3.25:0.025:-2.75)
**Metabolic phenotypes**				
Age at natural menopause (ANM)	(0.3:0.1:0.6)	(-6:0.25:-2)	0.4	(-5.75:0.025:-5.25)
Coronary artery disease (CAD)	(0.3:0.1:0.6)	(-5:0.25:-2)	0.3	(-4.25:0.25:-3.75)
Fasting glucose (FG)	(0.3:0.1:0.6)	(-6:0.25:-3)	0.6	(-6:0.05:-5)
Fasting insulin (FI)	(0.3:0.1:0.6)	(-6:0.25:-3)	0.6	(-6.25:0.025:-5.75)
Gout (GOUT)	(0.3:0.1:0.6)	(-5.5:0.25:-2)	(0.3:0.1:0.6)	(-5.5:0.05:-4.5)
High-density lipoprotein (HDL)	(0.3:0.1:0.6)	(-5:0.25:-2)	0.3	(-3.75:0.025:-3.25)
Heart rate (HR)	(0.3:0.1:0.6)	(-5:0.25:-2)	(0.3:0.1:0.4)	(-4.5:0.025:-4)
Low-density lipoprotein (LDL)	(0.3:0.1:0.6)	(-5:0.25:-2)	0.3	(-4:0.025:-3.5)
Myocardial infarction (MI)	(0.3:0.1:0.6)	(-5:0.25:-2)	0.3	(-4.5:0.025:-4)
Type 2 diabetes (T2D)	(0.3:0.1:0.6)	(-5:0.25:-2)	(0.3:0.1:0.6)	(-4.75:0.025:-4.25)
Total cholesterol (TC)	(0.3:0.1:0.6)	(-5:0.25:-2)	0.6	(-5:0.025:-4.5)
Triglycerides (TG)	(0.3:0.1:0.6)	(-6:0.25:-3)	0.5	(-6.25:0.025:-5.75)
Serum urate (URATE)	(0.3:0.1:0.6)	(-5.5:0.25:-2)	0.5	(-5.5:0.025:-5)
**Hematopoietic traits**				
Haemoglobin (HB)	(0.3:0.1:0.6)	(-6:0.25:-3)	0.6	(-6.25:0.025:-5.75)
Mean cell HB (MCH)	(0.3:0.1:0.6)	(-5:0.25:-2)	0.6	(-4.75:0.05:-3.75)
Mean cell HB concentration (MCHC)	(0.3:0.1:0.6)	(-6:0.25:-3)	(0.3:0.1:0.6)	(-6:0.05:-5)
Mean cell volume (MCV)	(0.3:0.1:0.6)	(-5:0.25:-2)	0.6	(-4.25:0.025:-3.75)
Packed cell volume (PCV)	(0.3:0.1:0.6)	(-5:0.25:-2)	0.6	(-5.25:0.025:-4.75)
Red blood cell count (RBC)	(0.3:0.1:0.6)	(-5:0.25:-2)	(0.3:0.1:0.6)	(-3.75:0.025:-3.25)

8. Supplementary Table 7. Grids of hyper-parameters used in genome-wide multiple-SNP analyses of 31 human phenotypes, assuming a candidate pathway is enriched (i.e. the enrichment model $M_1 : \theta > 0$). Here `(j:i:k)` denotes a regularly-spaced vector that starts at `j`, uses `i` as the increment between elements, and (roughly) stops at `k`.

	Round 1 analysis			Round 2 analysis		
Phenotype (abbreviation)	h	θ_0	θ	h	θ_0	θ
**Neurological phenotypes**						
Amyotrophic lateral sclerosis (ALS)	(0.3:0.1:0.6)	(-6:0.25:-5)	(0:0.6:6)	(0.3:0.1:0.6)	(-6:0.05:-5)	(0:0.1:4)
Depressive symptoms (DS)	(0.3:0.1:0.6)	(-6:0.25:-5)	(0:0.6:6)	(0.3:0.1:0.6)	(-6:0.05:-5)	(0:0.15:6)
Alzheimer's disease (LOAD)	0.6	-5	(0:0.025:5)	0.6	(-5.150:0.025:-5.075)	(0:0.01:4)
Neuroticism (NEU)	(0.3:0.1:0.4)	(-4.5:0.25:-4)	(0:0.1:4)	0.3	(-4.5:0.025:-4)	(0:0.037:3.7)
Schizophrenia (SCZ)	0.3	-2	(0:0.01:2)	0.3	(-2.2:0.025:-2.05)	(0:0.01:2)
**Anthropometric traits**						
Body mass index (BMI)	0.3	-4	(0:0.02:4)	0.3	(-4.2:0.025:-3.8)	(0:0.018:3.5)
Height (HEIGHT)	0.3	-2	(0:0.01:2)	0.3	(-2.075:0.025:-1.925)	(0:0.01:1)
Waist-to-hip ratio (WHR)	0.3	-3	(0:0.015:3)	0.3	(-3:0.025:-2.95)	(0:0.01:3)
**Immune-related traits**						
Crohn's disease (CD)	0.3	-3	(0:0.015:3)	0.3	-3	(0:0.01:2)
Inflammatory bowel disease (IBD)	0.3	-3	(0:0.015:3)	0.3	(-3:0.025:-2.8)	(0:0.02:2)
Rheumatoid arthritis (RA)	0.3	-3.25	(0:0.016:3.25)	0.3	(-3.25:0.025:-3.175)	(0:0.01:2)
Ulcerative colitis (UC)	0.3	-3	(0:0.015:3)	0.3	(-3.175:0.025:-2.775)	(0:0.025:2.5)
**Metabolic phenotypes**						
Age at natural menopause (ANM)	0.4	-5.5	(0:0.028:5.5)	0.4	(-5.75:0.025:-5.7)	(0:0.04:4.5)
Coronary artery disease (CAD)	0.3	-4	(0:0.02:4)	0.3	(-4.025:0.025:-3.775)	(0:0.035:3.5)
Fasting glucose (FG)	0.6	-5.25	(0:0.026:5.25)	0.6	(-6:0.05:-5.75)	(0.1:0.1:4.5)
Fasting insulin (FI)	0.6	-6	(0:0.03:6)	0.6	(-6.25:0.025:-6)	(0:0.019:3.8)
Gout (GOUT)	(0.3:0.1:0.6)	(-5.5:0.25:-4.75)	(0:0.46:5.5)	(0.3:0.1:0.6)	(-5.5:0.05:-4.6)	(0:0.1:5)
High-density lipoprotein (HDL)	0.3	-3.5	(0:0.018:3.5)	0.3	(-3.575:0.025:-3.5)	(0:0.01:3)
Heart rate (HR)	(0.3:0.1:0.4)	(-4.5:0.25:-4.25)	(0:(4.5/50):4.5)	(0.3:0.1:0.4)	(-4.5:0.025:-4.1)	(0:0.038:3.8)
Low-density lipoprotein (LDL)	0.3	-3.75	(0:0.019:3.75)	0.3	(-3.625:0.025:-3.55)	(0:0.01:3)
Myocardial infarction (MI)	0.3	(-4.5:0.25:-4)	(0:0.067:4.5)	0.3	(-4.475:0.025:-4)	(0:0.045:4.5)
Type 2 diabetes (T2D)	(0.4:0.1:0.6)	-4.5	(0:0.067:4.5)	(0.3:0.1:0.6)	(-4.75:0.025:-4.35)	(0:0.3:3)
Total cholesterol (TC)	0.6	-4.75	(0:0.024:4.75)	0.6	(-4.8:0.025:-4.55)	(0:0.02:4)
Triglycerides (TG)	0.5	-4	(0:0.03:4)	0.5	(-6.25:0.025:-6.1)	(0:0.02:5.2)
Serum urate (URATE)	0.5	-5.25	(0:0.026:5.25)	0.5	(-5.4:0.025:-5)	(0:0.1:4.7)
**Hematopoietic traits**						
Haemoglobin (HB)	0.6	-6	(0:0.03:6)	0.6	(-6.25:0.025:-5.9)	(0:0.04:4.4)
Mean cell HB (MCH)	0.6	-4	(0:0.02:4)	0.6	(-4.7:0.05:-4.35)	(0:0.015:3)
Mean cell HB concentration (MCHC)	(0.3:0.1:0.6)	(-6:0.25:-5.25)	(0:0.5:6)	(0.3:0.1:0.6)	(-6:0.05:-5)	(0:0.1:5)
Mean cell volume (MCV)	0.6	-4	(0:0.02:4)	0.6	(-4.225:0.025:-4.125)	(0:0.02:3)
Packed cell volume (PCV)	0.6	-5	(0:0.025:5)	0.6	(-5.25:0.025:-5.15)	(0:0.02:4.5)
Red blood cell count (RBC)	(0.3:0.1:0.6)	-3.5	(0:0.07:3.5)	(0.5:0.1:0.6)	(-3.7:0.025:-3.6)	(0:0.035:3.5)

9. Supplementary Figure 1. Simulation details and additional results of **Figure 2(a)**. We use real genotypes of 12,758 SNPs on chromosome 16 from 1,458 individuals in the UK Blood Service Control Group [50] to simulate phenotype data, and then compute single-SNP association summary statistics. On these summary data, we compare RSS-E with existing enrichment methods.

We use *Signal Transduction Pathway* (Biosystem, Reactome) to create SNP-level annotation for these 12,758 SNPs. Specifically, we let $a_j = 1$ if SNP j is within ±100 kb of the transcribed region of any *Signal Transduction Pathway* gene, and let $a_j = 0$ otherwise. There are 36 *Signal Transduction Pathway* genes on chromosome 16. There are 676 SNPs assigned to this gene set (i.e. $a_j = 1$).

We simulate baseline and enrichment datasets in a paired way. We first simulate the causal indicator γ_j of each SNP j for an enrichment dataset as follows:

$$\begin{aligned} \gamma_j &\sim \text{Bernoulli}(\pi_j), \\ \pi_j &= \left(1 + 10^{-(\theta_0 + a_j\theta)}\right)^{-1}, \end{aligned}$$

where θ_0 is the background parameter and θ is the enrichment parameter. We count the number of causal SNPs in this enrichment dataset as $n_c := \sum_j \gamma_j$, and then randomly choose n_c SNPs from chromosome 16 as causal SNPs for the baseline dataset.

Given the causal indicators $\{\gamma_j\}$, we simulate the genetic effect β_j of each SNP j as follows:

$$\begin{aligned} \beta_j | \gamma_j = 0 &\sim \delta_0, \\ \beta_j | \gamma_j = 1 &\sim \text{Normal}(0, 1), \end{aligned}$$

where δ_0 denotes point mass at zero. Next, we simulate the phenotype y_i of Individual i as follows:

$$\begin{aligned} y_i &= \sum_j x_{ij}\beta_j + \epsilon_i, \\ \epsilon_i &\sim \text{Normal}(0, \tau^{-1}), \end{aligned}$$

where x_{ij} is the genotype of SNP j for Individual i, $j = 1, \ldots, 12{,}758$ and $i = 1, \ldots, 1{,}458$. The true value of residual variance τ^{-1} is determined by the true value of PVE (total proportion of variance in phenotype $\mathbf{y}$ explained by effects of all available SNPs $\mathbf{X}$) as follows:

$$\text{PVE} = \frac{V(\mathbf{X}\beta)}{\tau^{-1} + V(\mathbf{X}\beta)},$$

where $V(\mathbf{X}\beta)$ is the sample variance of $\mathbf{X}\beta$. We adopt this phenotype simulation scheme from previous work, notably [1, 51, 52].

In this set of simulations, the true values of background parameter θ_0 are $\{-2, -3, -4\}$, the true value of enrichment parameter θ is 2, and the true values of PVE are $\{0.05, 0.1, 0.2, 0.6\}$. For each combination of $(\theta_0, \theta, \text{PVE})$, we simulate 200 baseline and 200 enrichment independent datasets. The "sparse scenario" in **Figure 2(a)** corresponds to simulations with true $\theta_0 = -4$ and PVE = 0.05. The "polygenic scenario" in **Figure 2(a)** corresponds to simulations with true $\theta_0 = -2$ and PVE = 0.05.

We apply RSS-E to the simulated datasets, using the program `rss_varbvsr_squarem.m` available at https://github.com/stephenslab/rss. The input LD matrix is estimated from the 1,480 control individuals in the WTCCC 1958 British Birth Cohort, using a shrinkage estimator [53]. The grid on θ is `(0:0.05:1)` for baseline datasets, and is `(1:0.05:3)` for enrichment datasets. The grid on θ_0 is `(true_theta0-0.5:0.05:true_theta0+0.5)`, where `true_theta0` is the true value of θ_0 that was used to generate the dataset.

For each simulated dataset, we also perform enrichment analysis using two existing approaches with their default settings: Pascal [54] and LDSC [55]. Note that Pascal includes two gene scoring

options: maximum-of-χ^2 (-max) and sum-of-χ^2 (-sum), and two pathway scoring options: χ^2 approximation (-chi) and empirical sampling (-emp).

The candidate pathway for testing is *Signal Transduction Pathway* (Biosystem, Reactome) in both baseline and enrichment datasets. If a method identifies a baseline dataset as enriched, then it is a "false positive". If a method identifies an enrichment dataset as enriched, then it is a "true positive".

We evaluate the performance of these enrichment methods by plotting the receiver operating characteristic (ROC) curve and computing the area under the curve (AUC) for each method. Both metrics are implemented in the package plotROC [56].

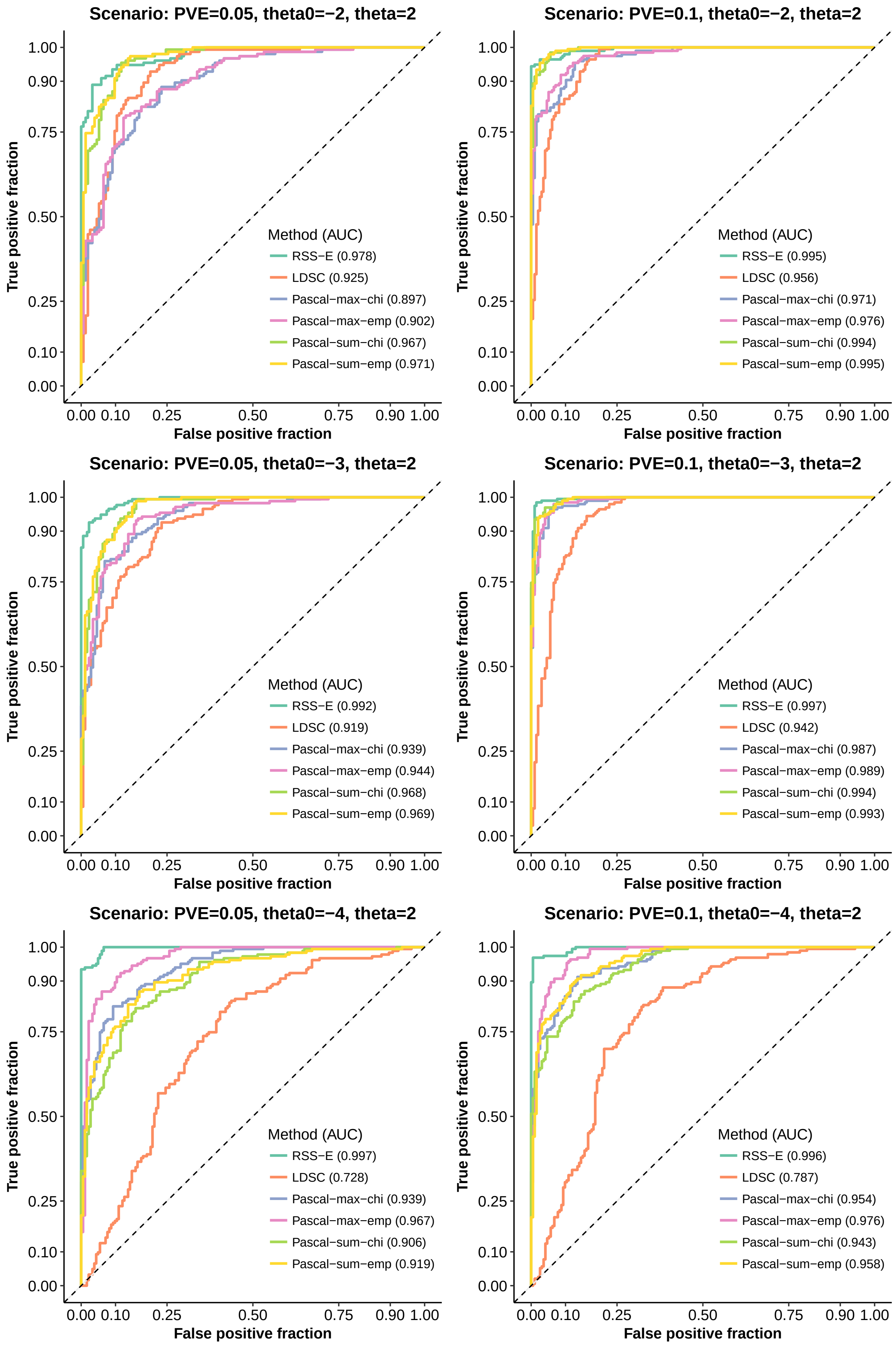

Scenario: PVE=0.05, theta0=−2, theta=2
True positive fraction
False positive fraction
1.00
0.90
0.75
0.50
0.25
0.10
0.00
0.00 0.10 0.25 0.50 0.75 0.90 1.00
Method (AUC)
RSS−E (0.978)
LDSC (0.925)
Pascal−max−chi (0.897)
Pascal−max−emp (0.902)
Pascal−sum−chi (0.967)
Pascal−sum−emp (0.971)
Scenario: PVE=0.1, theta0=−2, theta=2
True positive fraction
False positive fraction
1.00
0.90
0.75
0.50
0.25
0.10
0.00
0.00 0.10 0.25 0.50 0.75 0.90 1.00
Method (AUC)
RSS−E (0.995)
LDSC (0.956)
Pascal−max−chi (0.971)
Pascal−max−emp (0.976)
Pascal−sum−chi (0.994)
Pascal−sum−emp (0.995)
Scenario: PVE=0.05, theta0=−3, theta=2
True positive fraction
False positive fraction
1.00
0.90
0.75
0.50
0.25
0.10
0.00
0.00 0.10 0.25 0.50 0.75 0.90 1.00
Method (AUC)
RSS−E (0.992)
LDSC (0.919)
Pascal−max−chi (0.939)
Pascal−max−emp (0.944)
Pascal−sum−chi (0.968)
Pascal−sum−emp (0.969)
Scenario: PVE=0.1, theta0=−3, theta=2
True positive fraction
False positive fraction
1.00
0.90
0.75
0.50
0.25
0.10
0.00
0.00 0.10 0.25 0.50 0.75 0.90 1.00
Method (AUC)
RSS−E (0.997)
LDSC (0.942)
Pascal−max−chi (0.987)
Pascal−max−emp (0.989)
Pascal−sum−chi (0.994)
Pascal−sum−emp (0.993)
Scenario: PVE=0.05, theta0=−4, theta=2
True positive fraction
False positive fraction
1.00
0.90
0.75
0.50
0.25
0.10
0.00
0.00 0.10 0.25 0.50 0.75 0.90 1.00
Method (AUC)
RSS−E (0.997)
LDSC (0.728)
Pascal−max−chi (0.939)
Pascal−max−emp (0.967)
Pascal−sum−chi (0.906)
Pascal−sum−emp (0.919)
Scenario: PVE=0.1, theta0=−4, theta=2
True positive fraction
False positive fraction
1.00
0.90
0.75
0.50
0.25
0.10
0.00
0.00 0.10 0.25 0.50 0.75 0.90 1.00
Method (AUC)
RSS−E (0.996)
LDSC (0.787)
Pascal−max−chi (0.954)
Pascal−max−emp (0.976)
Pascal−sum−chi (0.943)
Pascal−sum−emp (0.958)

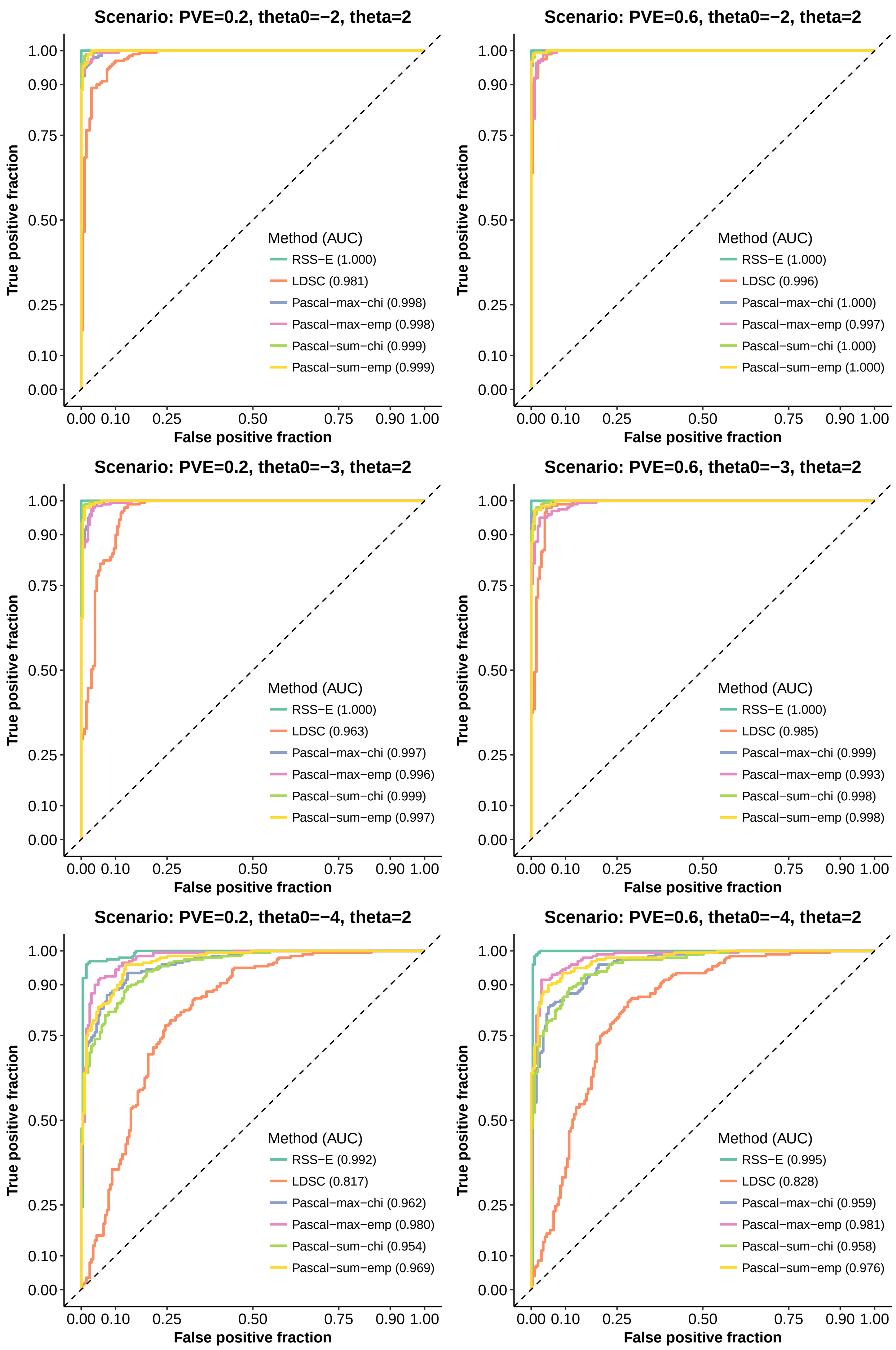
Scenario: PVE=0.2, theta0=−2, theta=2
Method (AUC)
RSS−E (1.000)
LDSC (0.981)
Pascal−max−chi (0.998)
Pascal−max−emp (0.998)
Pascal−sum−chi (0.999)
Pascal−sum−emp (0.999)
Scenario: PVE=0.6, theta0=−2, theta=2
Method (AUC)
RSS−E (1.000)
LDSC (0.996)
Pascal−max−chi (1.000)
Pascal−max−emp (0.997)
Pascal−sum−chi (1.000)
Pascal−sum−emp (1.000)
Scenario: PVE=0.2, theta0=−3, theta=2
Method (AUC)
RSS−E (1.000)
LDSC (0.963)
Pascal−max−chi (0.997)
Pascal−max−emp (0.996)
Pascal−sum−chi (0.999)
Pascal−sum−emp (0.997)
Scenario: PVE=0.6, theta0=−3, theta=2
Method (AUC)
RSS−E (1.000)
LDSC (0.985)
Pascal−max−chi (0.999)
Pascal−max−emp (0.993)
Pascal−sum−chi (0.998)
Pascal−sum−emp (0.998)
Scenario: PVE=0.2, theta0=−4, theta=2
Method (AUC)
RSS−E (0.992)
LDSC (0.817)
Pascal−max−chi (0.962)
Pascal−max−emp (0.980)
Pascal−sum−chi (0.954)
Pascal−sum−emp (0.969)
Scenario: PVE=0.6, theta0=−4, theta=2
Method (AUC)
RSS−E (0.995)
LDSC (0.828)
Pascal−max−chi (0.959)
Pascal−max−emp (0.981)
Pascal−sum−chi (0.958)
Pascal−sum−emp (0.976)
True positive fraction
False positive fraction
0.00 0.10 0.25 0.50 0.75 0.90 1.00

10. Supplementary Figure 2. Simulation details and additional results of **Figure 2(b)**. Details of this set of simulations are almost identical to those in **Supplementary Figure 1**. Here we only highlight the differences.

This set of simulations aims to assess the robustness of RSS-E to model misspecification where a random set of "near-gene" SNPs is enriched for genetic association in baseline datasets.

We define a SNP as "near-gene" if this SNP is within ±100 kb of the transcribed region of any gene. In total, there are 878 genes and 9,356 near-gene SNPs on chromosome 16.

We first simulate an enrichment dataset as in **Supplementary Figure 1**. For this enrichment dataset, we count the total number of causal SNPs as $n_c = \sum_j \gamma_j$, and count the number of causal SNPs in the target pathway as $n_p = \sum_j \gamma_j a_j$. We randomly choose n_p SNPs from the 9,356 near-gene SNPs and $n_c - n_p$ SNPs from the remaining 3,402 SNPs, and use them as causal SNPs to create a baseline dataset.

The "sparse scenario" in **Figure 2(b)** corresponds to simulations with true $\theta_0 = -4$ and PVE = 0.05. The "polygenic scenario" in **Figure 2(b)** corresponds to simulations with true $\theta_0 = -2$ and PVE = 0.05.

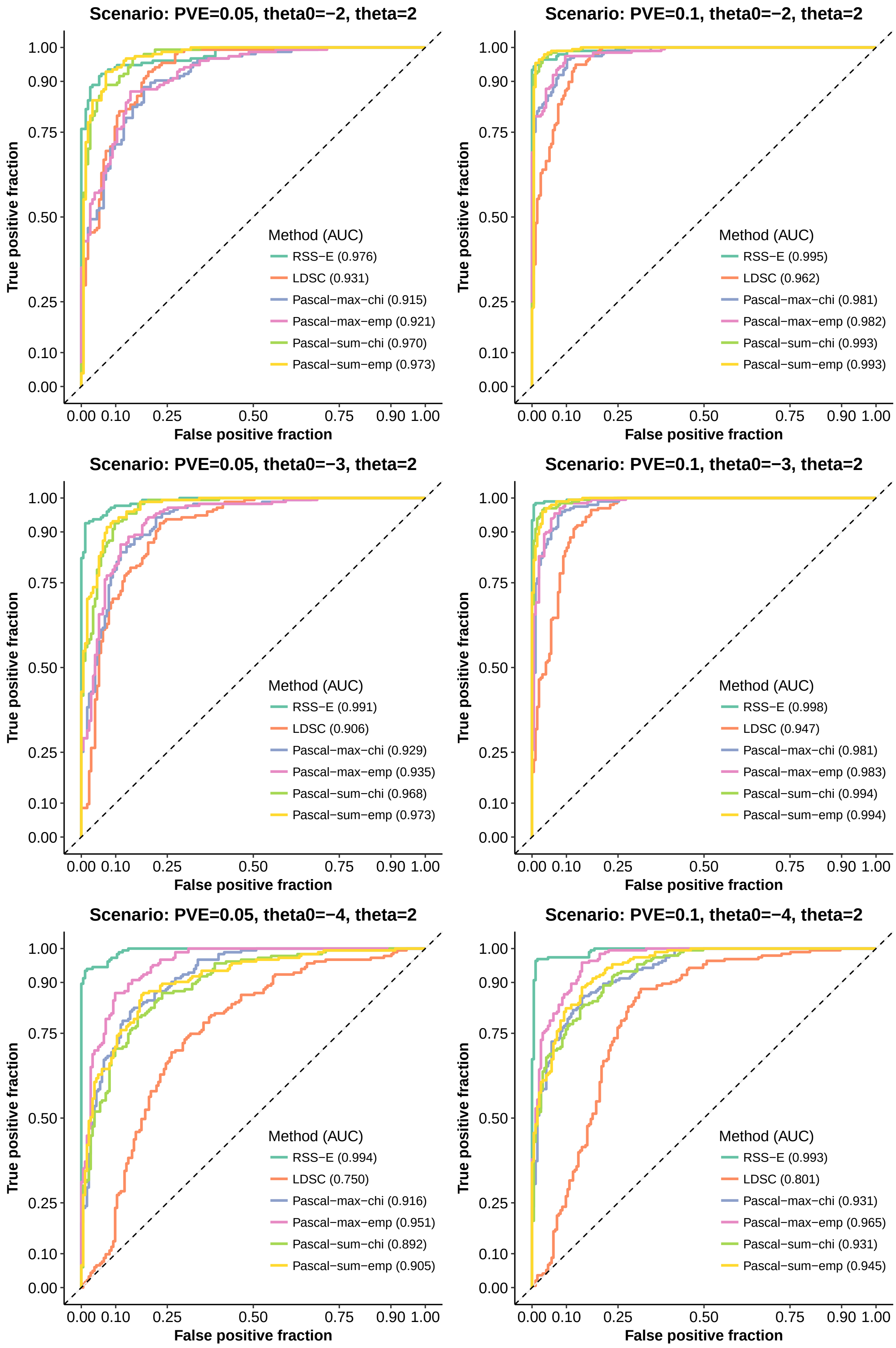
Scenario: PVE=0.05, theta0=−2, theta=2
True positive fraction
False positive fraction
Method (AUC)
RSS−E (0.976)
LDSC (0.931)
Pascal−max−chi (0.915)
Pascal−max−emp (0.921)
Pascal−sum−chi (0.970)
Pascal−sum−emp (0.973)
Scenario: PVE=0.1, theta0=−2, theta=2
Method (AUC)
RSS−E (0.995)
LDSC (0.962)
Pascal−max−chi (0.981)
Pascal−max−emp (0.982)
Pascal−sum−chi (0.993)
Pascal−sum−emp (0.993)
Scenario: PVE=0.05, theta0=−3, theta=2
Method (AUC)
RSS−E (0.991)
LDSC (0.906)
Pascal−max−chi (0.929)
Pascal−max−emp (0.935)
Pascal−sum−chi (0.968)
Pascal−sum−emp (0.973)
Scenario: PVE=0.1, theta0=−3, theta=2
Method (AUC)
RSS−E (0.998)
LDSC (0.947)
Pascal−max−chi (0.981)
Pascal−max−emp (0.983)
Pascal−sum−chi (0.994)
Pascal−sum−emp (0.994)
Scenario: PVE=0.05, theta0=−4, theta=2
Method (AUC)
RSS−E (0.994)
LDSC (0.750)
Pascal−max−chi (0.916)
Pascal−max−emp (0.951)
Pascal−sum−chi (0.892)
Pascal−sum−emp (0.905)
Scenario: PVE=0.1, theta0=−4, theta=2
Method (AUC)
RSS−E (0.993)
LDSC (0.801)
Pascal−max−chi (0.931)
Pascal−max−emp (0.965)
Pascal−sum−chi (0.931)
Pascal−sum−emp (0.945)
0.00 0.10 0.25 0.50 0.75 0.90 1.00

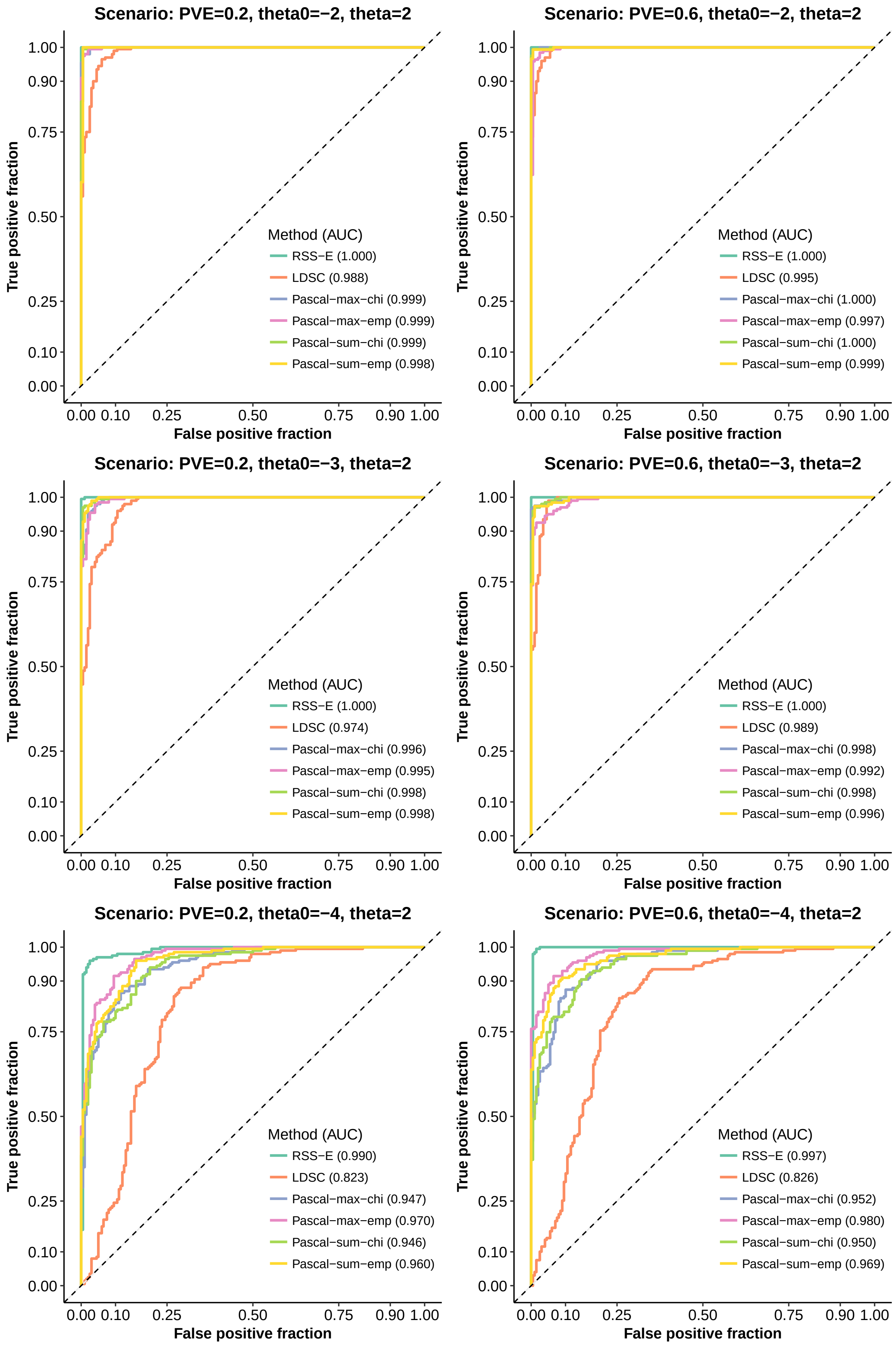
Scenario: PVE=0.2, theta0=−2, theta=2
Scenario: PVE=0.6, theta0=−2, theta=2
Scenario: PVE=0.2, theta0=−3, theta=2
Scenario: PVE=0.6, theta0=−3, theta=2
Scenario: PVE=0.2, theta0=−4, theta=2
Scenario: PVE=0.6, theta0=−4, theta=2
True positive fraction
False positive fraction
0.00 0.10 0.25 0.50 0.75 0.90 1.00
Method (AUC)
RSS−E (1.000)
LDSC (0.988)
Pascal−max−chi (0.999)
Pascal−max−emp (0.999)
Pascal−sum−chi (0.999)
Pascal−sum−emp (0.998)
Method (AUC)
RSS−E (1.000)
LDSC (0.995)
Pascal−max−chi (1.000)
Pascal−max−emp (0.997)
Pascal−sum−chi (1.000)
Pascal−sum−emp (0.999)
Method (AUC)
RSS−E (1.000)
LDSC (0.974)
Pascal−max−chi (0.996)
Pascal−max−emp (0.995)
Pascal−sum−chi (0.998)
Pascal−sum−emp (0.998)
Method (AUC)
RSS−E (1.000)
LDSC (0.989)
Pascal−max−chi (0.998)
Pascal−max−emp (0.992)
Pascal−sum−chi (0.998)
Pascal−sum−emp (0.996)
Method (AUC)
RSS−E (0.990)
LDSC (0.823)
Pascal−max−chi (0.947)
Pascal−max−emp (0.970)
Pascal−sum−chi (0.946)
Pascal−sum−emp (0.960)
Method (AUC)
RSS−E (0.997)
LDSC (0.826)
Pascal−max−chi (0.952)
Pascal−max−emp (0.980)
Pascal−sum−chi (0.950)
Pascal−sum−emp (0.969)

11. Supplementary Figure 3. Simulation details and additional results of **Figure 2(c)**. Details of this set of simulations are almost identical to those in **Supplementary Figure 1**. Here we only highlight the differences.

This set of simulations aims to assess the robustness of RSS-E to model misspecification where a random set of "coding" SNPs is enriched for genetic association in baseline datasets.

We define a SNP as "coding" if it is under the Sequence Ontology term `coding_sequence_variant` or its children terms (http://www.sequenceontology.org/miso/release_2.5/term/SO:0001580). In total, there are 124 coding SNPs on chromosome 16.

We first simulate an enrichment dataset as in **Supplementary Figure 1**. For this enrichment dataset, we count the total number of causal SNPs as $n_c = \sum_j \gamma_j$, and count the number of causal SNPs in the target pathway as $n_p = \sum_j \gamma_j a_j$. We randomly choose $\min(n_p, 124)$ SNPs from the 124 coding SNPs and $n_c - \min(n_p, 124)$ SNPs from the 12,634 non-coding SNPs, and use them as causal SNPs to create a baseline dataset.

The "sparse scenario" in **Figure 2(c)** corresponds to simulations with true $\theta_0 = -4$ and PVE = 0.05. The "polygenic scenario" in **Figure 2(c)** corresponds to simulations with true $\theta_0 = -2$ and PVE = 0.05.

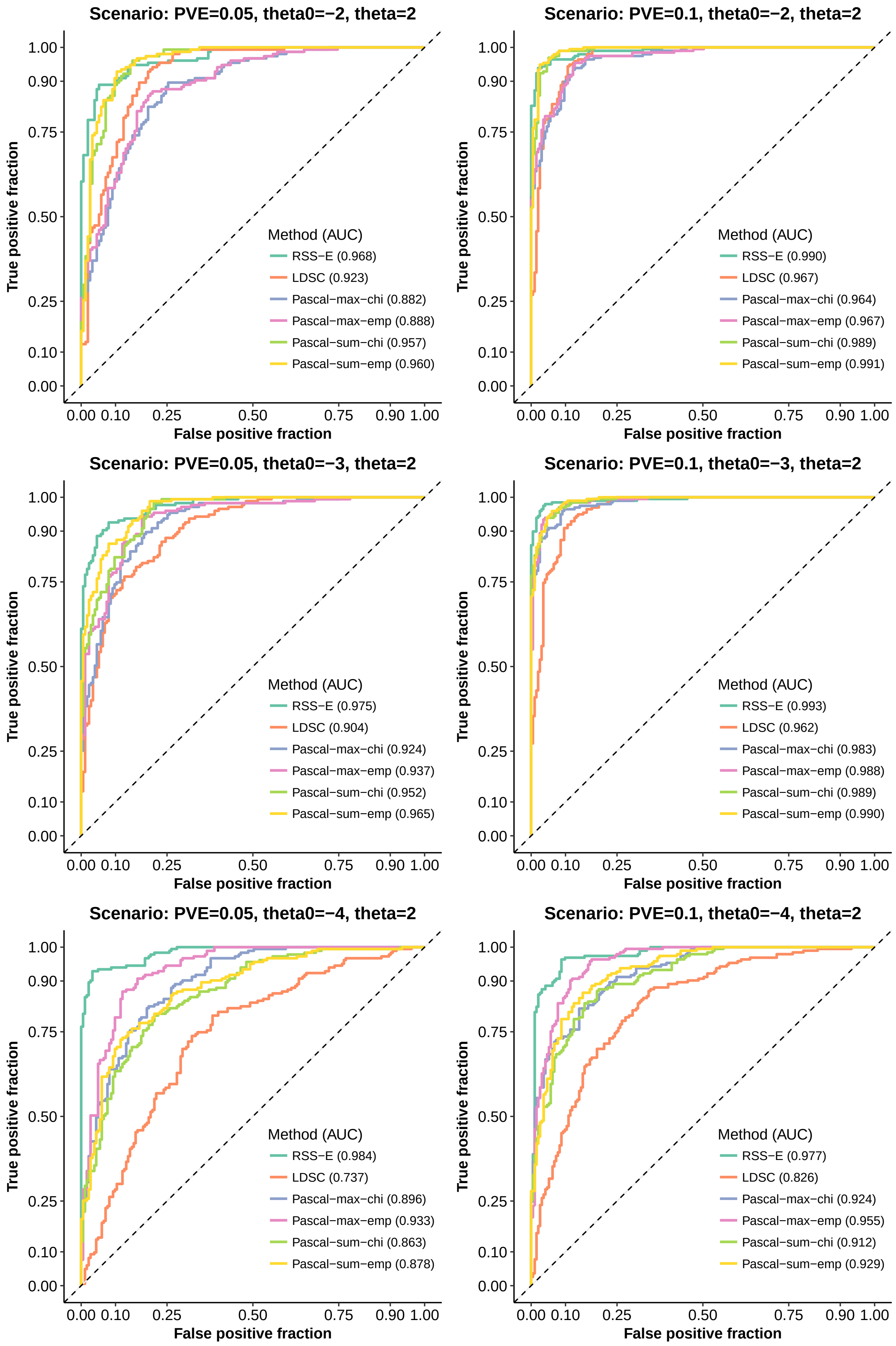
Scenario: PVE=0.05, theta0=−2, theta=2
True positive fraction
False positive fraction
Method (AUC)
RSS−E (0.968)
LDSC (0.923)
Pascal−max−chi (0.882)
Pascal−max−emp (0.888)
Pascal−sum−chi (0.957)
Pascal−sum−emp (0.960)
Scenario: PVE=0.1, theta0=−2, theta=2
True positive fraction
False positive fraction
Method (AUC)
RSS−E (0.990)
LDSC (0.967)
Pascal−max−chi (0.964)
Pascal−max−emp (0.967)
Pascal−sum−chi (0.989)
Pascal−sum−emp (0.991)
Scenario: PVE=0.05, theta0=−3, theta=2
True positive fraction
False positive fraction
Method (AUC)
RSS−E (0.975)
LDSC (0.904)
Pascal−max−chi (0.924)
Pascal−max−emp (0.937)
Pascal−sum−chi (0.952)
Pascal−sum−emp (0.965)
Scenario: PVE=0.1, theta0=−3, theta=2
True positive fraction
False positive fraction
Method (AUC)
RSS−E (0.993)
LDSC (0.962)
Pascal−max−chi (0.983)
Pascal−max−emp (0.988)
Pascal−sum−chi (0.989)
Pascal−sum−emp (0.990)
Scenario: PVE=0.05, theta0=−4, theta=2
True positive fraction
False positive fraction
Method (AUC)
RSS−E (0.984)
LDSC (0.737)
Pascal−max−chi (0.896)
Pascal−max−emp (0.933)
Pascal−sum−chi (0.863)
Pascal−sum−emp (0.878)
Scenario: PVE=0.1, theta0=−4, theta=2
True positive fraction
False positive fraction
Method (AUC)
RSS−E (0.977)
LDSC (0.826)
Pascal−max−chi (0.924)
Pascal−max−emp (0.955)
Pascal−sum−chi (0.912)
Pascal−sum−emp (0.929)

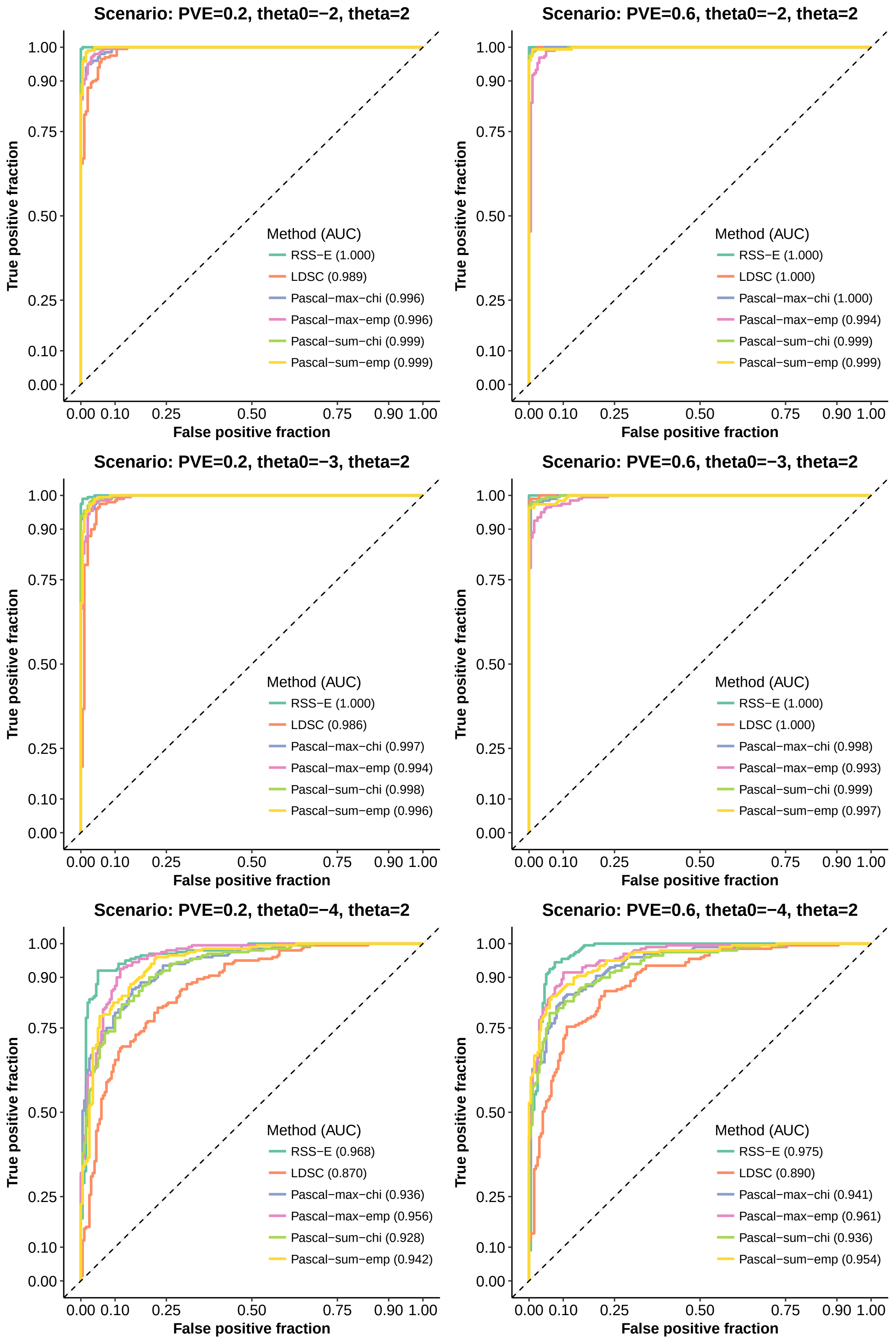

Scenario: PVE=0.2, theta0=−2, theta=2
Method (AUC)
RSS−E (1.000)
LDSC (0.989)
Pascal−max−chi (0.996)
Pascal−max−emp (0.996)
Pascal−sum−chi (0.999)
Pascal−sum−emp (0.999)
Scenario: PVE=0.6, theta0=−2, theta=2
Method (AUC)
RSS−E (1.000)
LDSC (1.000)
Pascal−max−chi (1.000)
Pascal−max−emp (0.994)
Pascal−sum−chi (0.999)
Pascal−sum−emp (0.999)
Scenario: PVE=0.2, theta0=−3, theta=2
Method (AUC)
RSS−E (1.000)
LDSC (0.986)
Pascal−max−chi (0.997)
Pascal−max−emp (0.994)
Pascal−sum−chi (0.998)
Pascal−sum−emp (0.996)
Scenario: PVE=0.6, theta0=−3, theta=2
Method (AUC)
RSS−E (1.000)
LDSC (1.000)
Pascal−max−chi (0.998)
Pascal−max−emp (0.993)
Pascal−sum−chi (0.999)
Pascal−sum−emp (0.997)
Scenario: PVE=0.2, theta0=−4, theta=2
Method (AUC)
RSS−E (0.968)
LDSC (0.870)
Pascal−max−chi (0.936)
Pascal−max−emp (0.956)
Pascal−sum−chi (0.928)
Pascal−sum−emp (0.942)
Scenario: PVE=0.6, theta0=−4, theta=2
Method (AUC)
RSS−E (0.975)
LDSC (0.890)
Pascal−max−chi (0.941)
Pascal−max−emp (0.961)
Pascal−sum−chi (0.936)
Pascal−sum−emp (0.954)
True positive fraction
False positive fraction
0.00 0.10 0.25 0.50 0.75 0.90 1.00

12. Supplementary Figure 4. Simulation details and additional results of **Figure 2(d)**. Details of this set of simulations are almost identical to those in **Supplementary Figure 1**. Here we only highlight the differences.

This set of simulations aims to assess the robustness of RSS-E to model misspecification where SNPs inside the target pathway are not only more likely to be associated with the trait, but also have larger effect on the trait, compared with SNPs outside the target pathway.

We create this type of enrichment data as follows. We first simulate the causal indicators for an enrichment dataset as in **Supplementary Figure 1**, and then simulate effect sizes as follows:

$$\begin{aligned} \beta_j | \gamma_j = 0 &\sim \delta_0, \\ \beta_j | \gamma_j = 1, a_j = 0 &\sim \text{Normal}(0, 0.01^2), \\ \beta_j | \gamma_j = 1, a_j = 1 &\sim \text{Normal}(0, 1). \end{aligned}$$

The corresponding baseline dataset is simulated as in **Supplementary Figure 1**.

The "sparse scenario" in **Figure 2(d)** corresponds to simulations with true $\theta_0 = -4$ and PVE = 0.05. The "polygenic scenario" in **Figure 2(d)** corresponds to simulations with true $\theta_0 = -2$ and PVE = 0.05.

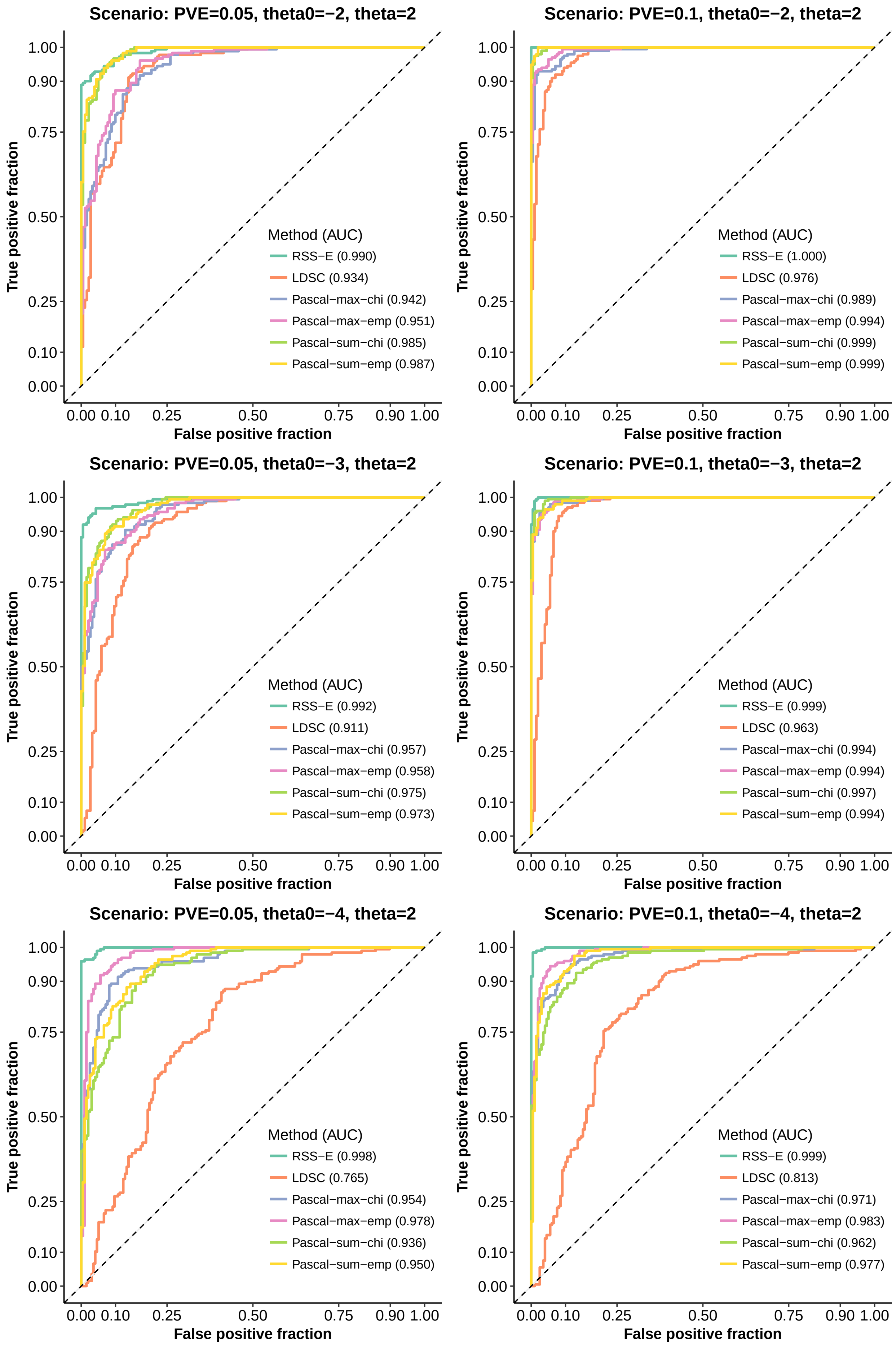
Scenario: PVE=0.05, theta0=−2, theta=2
Method (AUC)
RSS−E (0.990)
LDSC (0.934)
Pascal−max−chi (0.942)
Pascal−max−emp (0.951)
Pascal−sum−chi (0.985)
Pascal−sum−emp (0.987)
Scenario: PVE=0.1, theta0=−2, theta=2
Method (AUC)
RSS−E (1.000)
LDSC (0.976)
Pascal−max−chi (0.989)
Pascal−max−emp (0.994)
Pascal−sum−chi (0.999)
Pascal−sum−emp (0.999)
Scenario: PVE=0.05, theta0=−3, theta=2
Method (AUC)
RSS−E (0.992)
LDSC (0.911)
Pascal−max−chi (0.957)
Pascal−max−emp (0.958)
Pascal−sum−chi (0.975)
Pascal−sum−emp (0.973)
Scenario: PVE=0.1, theta0=−3, theta=2
Method (AUC)
RSS−E (0.999)
LDSC (0.963)
Pascal−max−chi (0.994)
Pascal−max−emp (0.994)
Pascal−sum−chi (0.997)
Pascal−sum−emp (0.994)
Scenario: PVE=0.05, theta0=−4, theta=2
Method (AUC)
RSS−E (0.998)
LDSC (0.765)
Pascal−max−chi (0.954)
Pascal−max−emp (0.978)
Pascal−sum−chi (0.936)
Pascal−sum−emp (0.950)
Scenario: PVE=0.1, theta0=−4, theta=2
Method (AUC)
RSS−E (0.999)
LDSC (0.813)
Pascal−max−chi (0.971)
Pascal−max−emp (0.983)
Pascal−sum−chi (0.962)
Pascal−sum−emp (0.977)
True positive fraction
False positive fraction
0.00 0.10 0.25 0.50 0.75 0.90 1.00

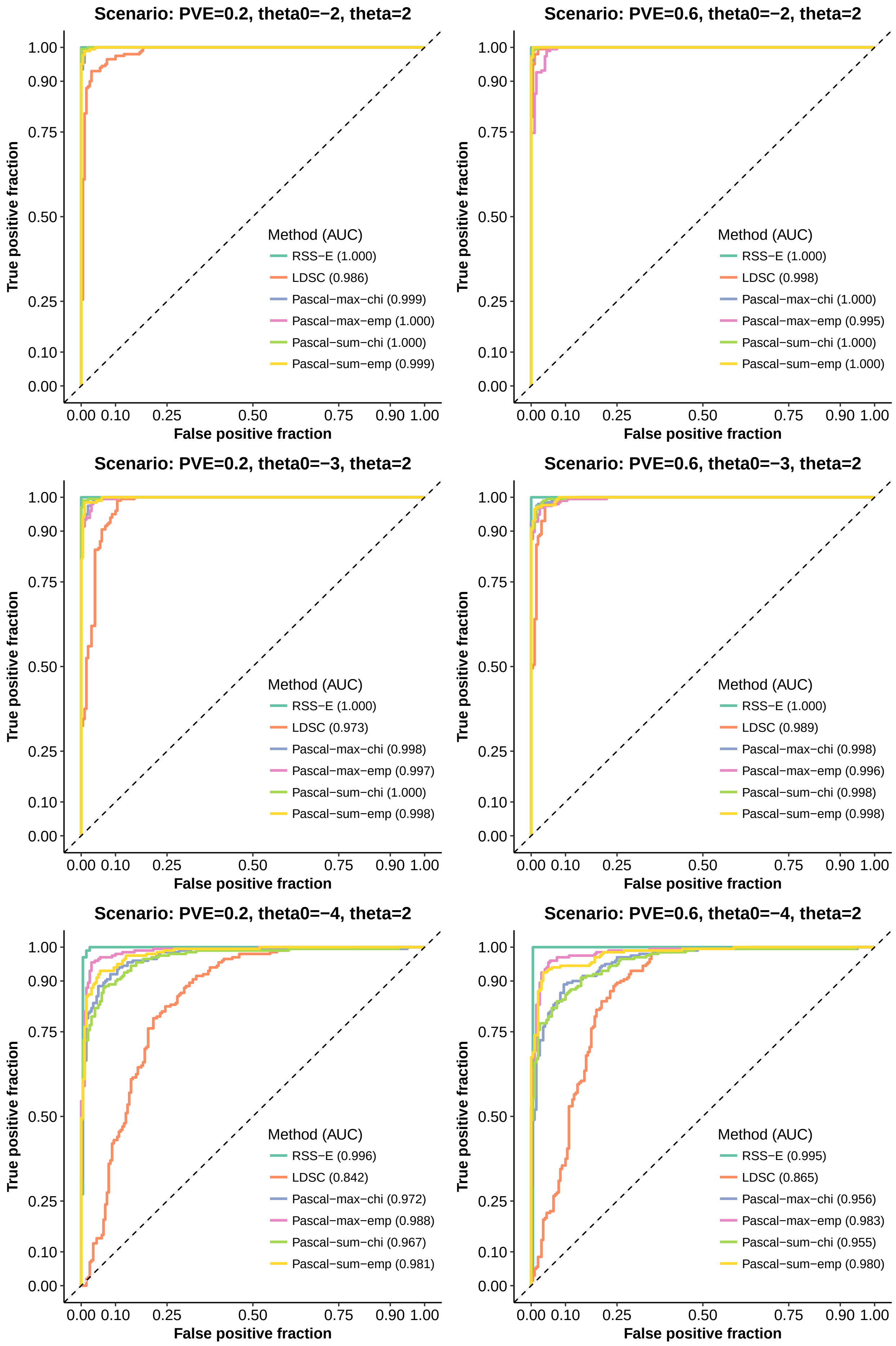
Scenario: PVE=0.2, theta0=−2, theta=2
Method (AUC)
RSS−E (1.000)
LDSC (0.986)
Pascal−max−chi (0.999)
Pascal−max−emp (1.000)
Pascal−sum−chi (1.000)
Pascal−sum−emp (0.999)
Scenario: PVE=0.6, theta0=−2, theta=2
Method (AUC)
RSS−E (1.000)
LDSC (0.998)
Pascal−max−chi (1.000)
Pascal−max−emp (0.995)
Pascal−sum−chi (1.000)
Pascal−sum−emp (1.000)
Scenario: PVE=0.2, theta0=−3, theta=2
Method (AUC)
RSS−E (1.000)
LDSC (0.973)
Pascal−max−chi (0.998)
Pascal−max−emp (0.997)
Pascal−sum−chi (1.000)
Pascal−sum−emp (0.998)
Scenario: PVE=0.6, theta0=−3, theta=2
Method (AUC)
RSS−E (1.000)
LDSC (0.989)
Pascal−max−chi (0.998)
Pascal−max−emp (0.996)
Pascal−sum−chi (0.998)
Pascal−sum−emp (0.998)
Scenario: PVE=0.2, theta0=−4, theta=2
Method (AUC)
RSS−E (0.996)
LDSC (0.842)
Pascal−max−chi (0.972)
Pascal−max−emp (0.988)
Pascal−sum−chi (0.967)
Pascal−sum−emp (0.981)
Scenario: PVE=0.6, theta0=−4, theta=2
Method (AUC)
RSS−E (0.995)
LDSC (0.865)
Pascal−max−chi (0.956)
Pascal−max−emp (0.983)
Pascal−sum−chi (0.955)
Pascal−sum−emp (0.980)
True positive fraction
False positive fraction
0.00
0.10
0.25
0.50
0.75
0.90
1.00

13. Supplementary Figure 5. Simulations to assess the impact of "HapMap3 SNP subsetting" on enrichment analyses. Details of this set of simulations are almost identical to those in **Supplementary Figure 1**. Here we only highlight the differences.

This set of simulations aims to assess the impact of "SNP subsetting" on enrichment analyses. The "SNP subsetting" strategy uses only the summary data of HapMap3 SNPs, even though the summary data of all 1000 Genomes SNPs are available. This strategy has been widely used in recent analyses of GWAS summary statistics [48, 55], and it is also used in our data analyses (merely to reduce computations). Of note, this is the **only** simulation study that uses "SNP subsetting" strategy.

To mimic "SNP subsetting" used in our data analyses, we use genotypes of 255,584 SNPs on chromosome 16 from 503 individuals of European ancestry in the 1000 Genomes Phase 3 [57] to simulate phenotype data, and then compute single-SNP association summary statistics for these 255,584 1000 Genomes SNPs. The remaining simulation details are the same as **Supplementary Figure 1**.

For each simulated dataset, we use each enrichment method to perform two analyses:

- analysis "with SNP subsetting" that uses summary data of 36,121 HapMap3 SNPs **only**;
- analysis "without SNP subsetting" that uses summary data of **all** 255,584 1000 Genomes SNPs.

RSS-E with SNP subsetting slightly outperforms RSS-E without SNP subsetting. This is expected, since the variational inference algorithm underlying RSS-E performs better when less variables (SNPs) are highly correlated (in high LD), and there are more highly correlated SNPs among 255,584 1000 Genomes SNPs than 36,121 HapMap3 SNPs. See [3] for more extensive discussion.

Similarly, Pascal with SNP subsetting also outperforms Pascal without SNP subsetting. We speculate that the large number of highly correlated 1000 Genomes SNPs has a negative impact on gene-score calculations and "gene-fusion" strategy used in Pascal [54].

For LDSC, results with and without SNP subsetting are almost identical. This is because for both analyses, LDSC (by default) only uses LD scores of HapMap3 SNPs as regression weights (downloaded from `https://data.broadinstitute.org/alkesgroup/LDSCORE/weights_hm3_no_hla.tgz`).

Finally, we clarify the following subtle but important detail. The "SNP subsetting" strategy used in LDSC [55] consists of two steps:

1. using GWAS summary statistics of HapMap3 SNPs (i.e. "regression SNPs") to compute marginal association test statistics $\{\chi_j^2\}$;
2. using LD estimates of 1000 Genomes SNPs (i.e. "reference SNPs") to compute LD scores $\{\ell_j\}$ for "regression SNPs".

Unlike LDSC, both RSS-E and Pascal only have "regression SNPs" in their statistical models. Hence, we only use Step 1 above in this set of simulations and, as a result, for analyses with "SNP subsetting", RSS-E and Pascal only use LD estimates of HapMap3 SNPs, whereas LDSC still uses LD estimates of 1000 Genomes SNPs.

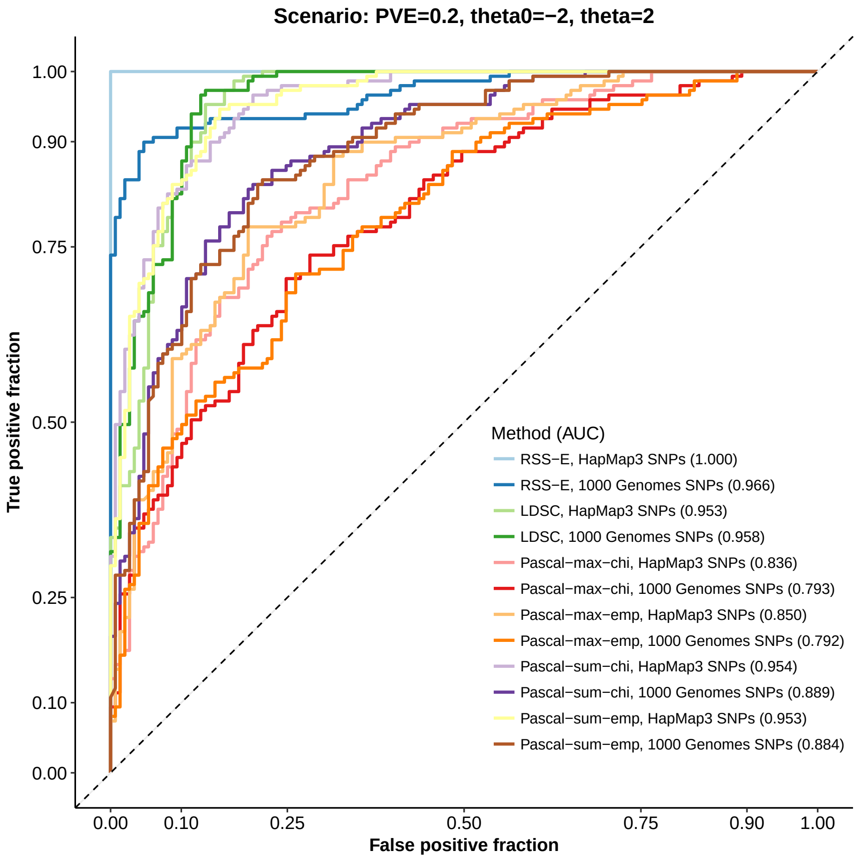
Scenario: PVE=0.2, theta0=−2, theta=2
True positive fraction
False positive fraction
Method (AUC)
RSS−E, HapMap3 SNPs (1.000)
RSS−E, 1000 Genomes SNPs (0.966)
LDSC, HapMap3 SNPs (0.953)
LDSC, 1000 Genomes SNPs (0.958)
Pascal−max−chi, HapMap3 SNPs (0.836)
Pascal−max−chi, 1000 Genomes SNPs (0.793)
Pascal−max−emp, HapMap3 SNPs (0.850)
Pascal−max−emp, 1000 Genomes SNPs (0.792)
Pascal−sum−chi, HapMap3 SNPs (0.954)
Pascal−sum−chi, 1000 Genomes SNPs (0.889)
Pascal−sum−emp, HapMap3 SNPs (0.953)
Pascal−sum−emp, 1000 Genomes SNPs (0.884)

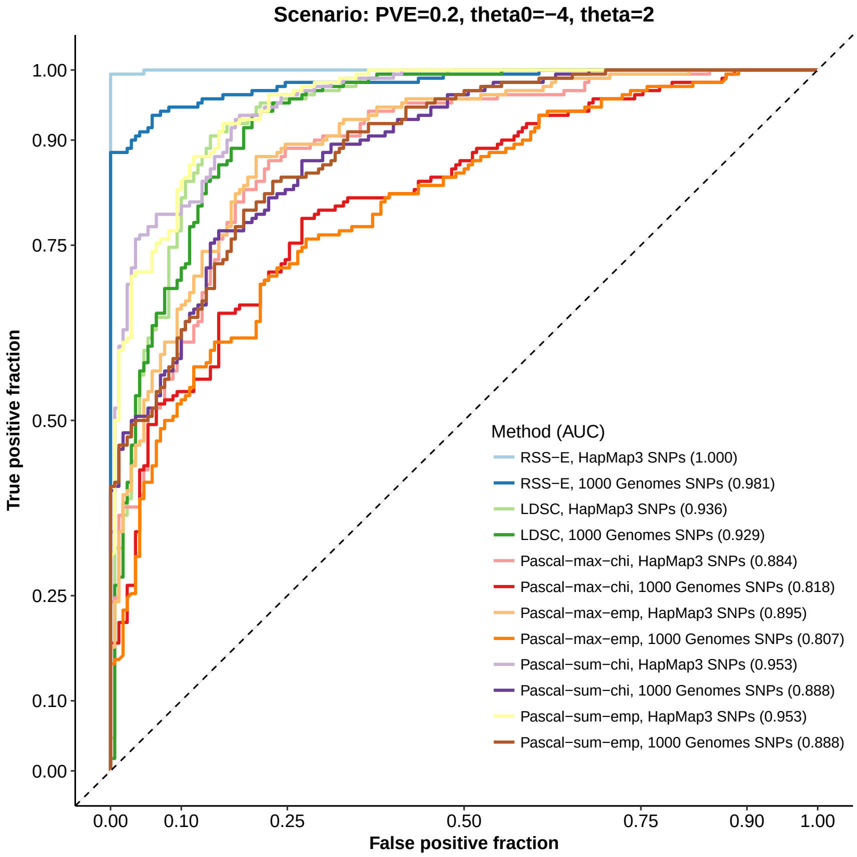
Scenario: PVE=0.2, theta0=−4, theta=2
True positive fraction
False positive fraction
Method (AUC)
RSS−E, HapMap3 SNPs (1.000)
RSS−E, 1000 Genomes SNPs (0.981)
LDSC, HapMap3 SNPs (0.936)
LDSC, 1000 Genomes SNPs (0.929)
Pascal−max−chi, HapMap3 SNPs (0.884)
Pascal−max−chi, 1000 Genomes SNPs (0.818)
Pascal−max−emp, HapMap3 SNPs (0.895)
Pascal−max−emp, 1000 Genomes SNPs (0.807)
Pascal−sum−chi, HapMap3 SNPs (0.953)
Pascal−sum−chi, 1000 Genomes SNPs (0.888)
Pascal−sum−emp, HapMap3 SNPs (0.953)
Pascal−sum−emp, 1000 Genomes SNPs (0.888)

14. Supplementary Figure 6. Simulation details and additional results of **Figure 3(a)**. Here we use real genotypes of 12,758 SNPs on chromosome 16 from 1,458 individuals in the UK Blood Service Control Group [50] to simulate phenotype data, and then compute single-SNP association summary statistics. On these summary data, we compare RSS-E with existing gene-level testing methods.

We first simulate the genetic effect β_j of each SNP j as follows:

$$\beta_j \sim (1-\pi)\cdot\delta_0 + \pi\cdot\text{Normal}(0,1),$$
$$\pi = \left(1+10^{-\theta_0}\right)^{-1},$$

where δ_0 denotes point mass at zero. We then simulate the phenotype y_i of Individual i as

$$y_i = \sum_j x_{ij}\beta_j + \epsilon_i,$$
$$\epsilon_i \sim \text{Normal}(0,\tau^{-1}),$$

where x_{ij} is the genotype of SNP j for Individual i, $j = 1,\ldots,12,758$ and $i = 1,\ldots,1,458$. The true value of residual variance τ^{-1} is determined by the true value of PVE (total proportion of variance in phenotype $\mathbf{y}$ explained by effects of all available SNPs $\mathbf{X}$) as follows:

$$\text{PVE} = \frac{V(\mathbf{X}\beta)}{\tau^{-1}+V(\mathbf{X}\beta)},$$

where $V(\mathbf{X}\beta)$ is the sample variance of $\mathbf{X}\beta$. We adopt the phenotype simulation scheme from previous work, notably [1, 51, 52].

In this set of simulations, the true values of background parameter θ_0 are $\{-1,-2,-3\}$, and the true values of PVE are $\{0.2, 0.6\}$. For each combination of true values of (θ_0, PVE), we simulate 100 independent datasets. The "Baseline & sparse" panel in **Figure 3(a)** corresponds to simulations with true $\theta_0 = -3$ and PVE = 0.2. The "Baseline & polygenic" panel in **Figure 3(a)** corresponds to simulations with true $\theta_0 = -1$ and PVE = 0.2.

We apply RSS-E to the simulated datasets, using the program `rss_varbvsr_squarem.m` available at `https://github.com/stephenslab/rss`. The input LD matrix is estimated from the 1,480 control individuals in the WTCCC 1958 British Birth Cohort, using a shrinkage estimator [53]. The enrichment parameter θ is set as zero. The grid on the background parameter θ_0 is `(-5:0.1:-1)`.

For each simulated dataset, we also perform gene-level association analysis using four existing approaches with their default settings: SimpleM [58], VEGAS [59], GATES [60] and COMBAT [61] that combines SimpleM, VEGAS and GATES results. Note that VEGAS is applied to the full set of SNPs within a gene (`-sum`), on a specified percentage of the most significant SNPs (`-10%` and `-20%`), or on the single most significant SNP (`-max`).

For each simulated dataset, we define a gene as "trait-associated" if at lease one SNP within ±100 kb of the transcribed region of this gene has non-zero effect ($\beta_j \neq 0$). For each gene in each simulated dataset, RSS-E produces P_1, the posterior probability that the gene is trait-associated. whereas the other methods produce p-value with the null hypothesis that the gene is not trait-associated; these statistics are used to rank the significance of gene-level associations. If a method identifies association between a non-trait-associated gene and the trait, then it is a "false positive". If a method identifies association between a trait-associated gene and the trait, then it is a "true positive".

We evaluate the performance of these gene-level association methods by plotting the receiver operating characteristic (ROC) curve and computing the area under the curve (AUC) for each method. Both metrics are implemented in the package `plotROC` [56].

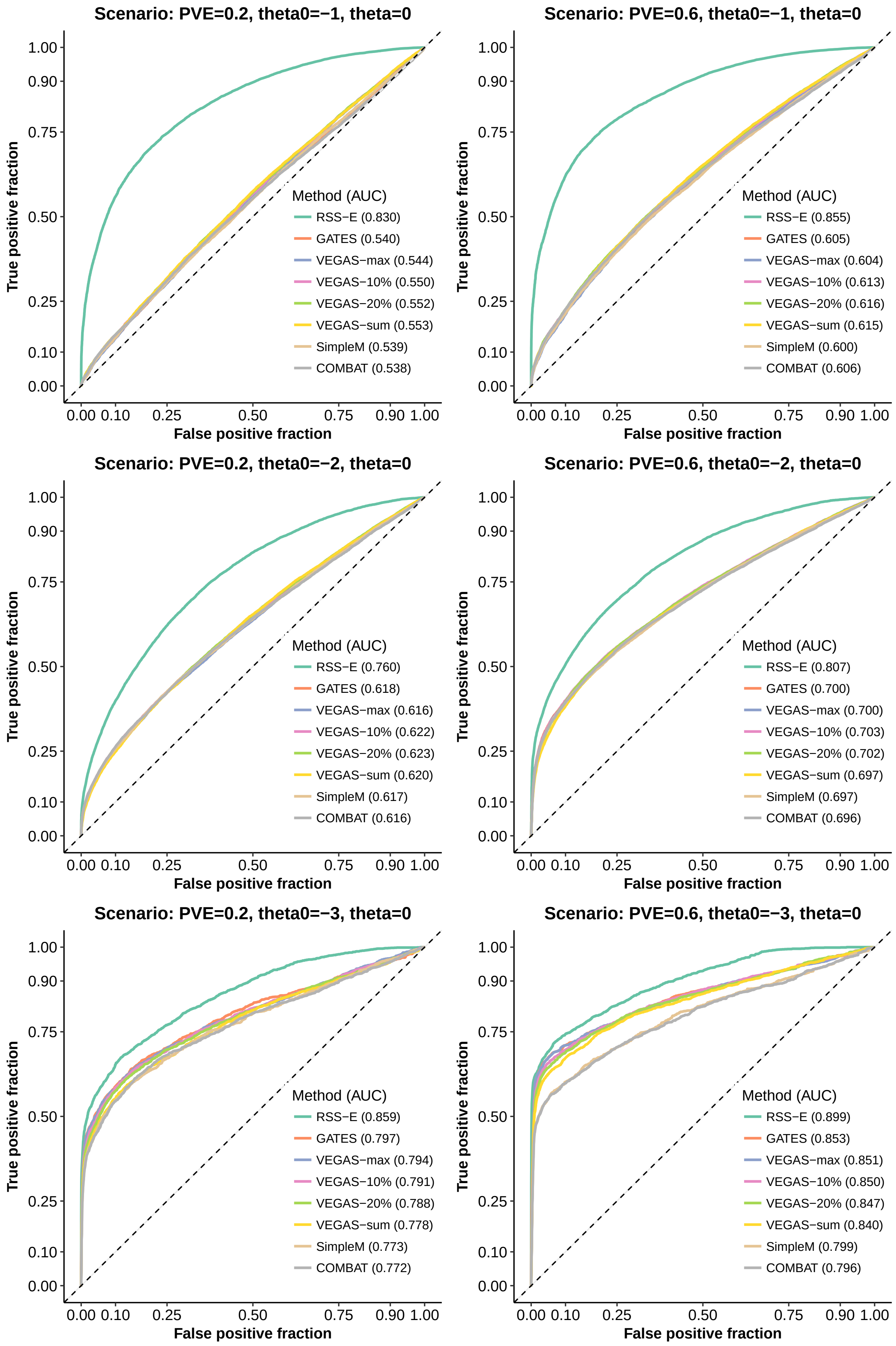
Scenario: PVE=0.2, theta0=−1, theta=0
Method (AUC)
RSS−E (0.830)
GATES (0.540)
VEGAS−max (0.544)
VEGAS−10% (0.550)
VEGAS−20% (0.552)
VEGAS−sum (0.553)
SimpleM (0.539)
COMBAT (0.538)
Scenario: PVE=0.6, theta0=−1, theta=0
Method (AUC)
RSS−E (0.855)
GATES (0.605)
VEGAS−max (0.604)
VEGAS−10% (0.613)
VEGAS−20% (0.616)
VEGAS−sum (0.615)
SimpleM (0.600)
COMBAT (0.606)
Scenario: PVE=0.2, theta0=−2, theta=0
Method (AUC)
RSS−E (0.760)
GATES (0.618)
VEGAS−max (0.616)
VEGAS−10% (0.622)
VEGAS−20% (0.623)
VEGAS−sum (0.620)
SimpleM (0.617)
COMBAT (0.616)
Scenario: PVE=0.6, theta0=−2, theta=0
Method (AUC)
RSS−E (0.807)
GATES (0.700)
VEGAS−max (0.700)
VEGAS−10% (0.703)
VEGAS−20% (0.702)
VEGAS−sum (0.697)
SimpleM (0.697)
COMBAT (0.696)
Scenario: PVE=0.2, theta0=−3, theta=0
Method (AUC)
RSS−E (0.859)
GATES (0.797)
VEGAS−max (0.794)
VEGAS−10% (0.791)
VEGAS−20% (0.788)
VEGAS−sum (0.778)
SimpleM (0.773)
COMBAT (0.772)
Scenario: PVE=0.6, theta0=−3, theta=0
Method (AUC)
RSS−E (0.899)
GATES (0.853)
VEGAS−max (0.851)
VEGAS−10% (0.850)
VEGAS−20% (0.847)
VEGAS−sum (0.840)
SimpleM (0.799)
COMBAT (0.796)
True positive fraction
False positive fraction
0.00 0.10 0.25 0.50 0.75 0.90 1.00

15. Supplementary Figure 7. Simulation details and additional results of **Figure 3(b)**. Details of this set of simulations are almost identical to those in **Supplementary Figure 6**. Here we only highlight the differences.

For this set of simulations, we generate the genetic effect of each SNP j as follows:

$$\beta_j \sim (1-\pi_j)\cdot\delta_0 + \pi_j \cdot \text{Normal}(0,1),$$
$$\pi_j = \left(1+10^{-(\theta_0+a_j\theta)}\right)^{-1},$$

where $\{\theta_0, \theta, a_j\}$ are defined in **Supplementary Figure 1**.

In this set of simulations, the true values of background parameter θ_0 are $\{-2,-3,-4\}$, the true value of enrichment parameter θ is 2, and the true values of PVE are $\{0.2, 0.6\}$.

When applying RSS-E to the simulated datasets, we fix the the background parameter θ_0 as the true value, and use a grid `(1:0.05:3)` for the enrichment parameter θ.

The "Enrichment & sparse" panel in **Figure 3(b)** corresponds to simulations with true $\theta_0 = -4$ and PVE = 0.2. The "Enrichment & polygenic" panel in **Figure 3(b)** corresponds to simulations with true $\theta_0 = -2$ and PVE = 0.2.

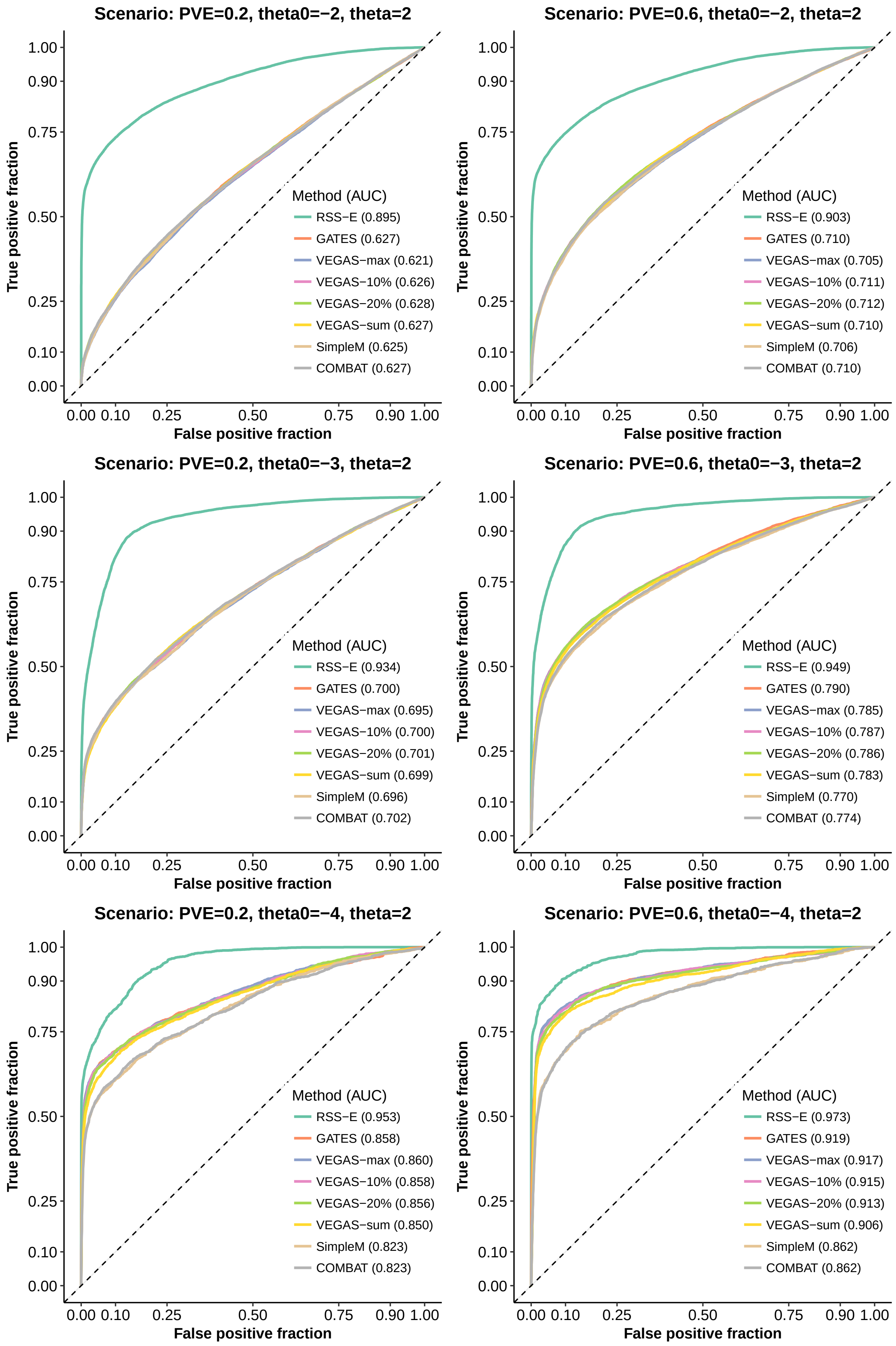
Scenario: PVE=0.2, theta0=−2, theta=2
Method (AUC)
RSS−E (0.895)
GATES (0.627)
VEGAS−max (0.621)
VEGAS−10% (0.626)
VEGAS−20% (0.628)
VEGAS−sum (0.627)
SimpleM (0.625)
COMBAT (0.627)
Scenario: PVE=0.6, theta0=−2, theta=2
Method (AUC)
RSS−E (0.903)
GATES (0.710)
VEGAS−max (0.705)
VEGAS−10% (0.711)
VEGAS−20% (0.712)
VEGAS−sum (0.710)
SimpleM (0.706)
COMBAT (0.710)
Scenario: PVE=0.2, theta0=−3, theta=2
Method (AUC)
RSS−E (0.934)
GATES (0.700)
VEGAS−max (0.695)
VEGAS−10% (0.700)
VEGAS−20% (0.701)
VEGAS−sum (0.699)
SimpleM (0.696)
COMBAT (0.702)
Scenario: PVE=0.6, theta0=−3, theta=2
Method (AUC)
RSS−E (0.949)
GATES (0.790)
VEGAS−max (0.785)
VEGAS−10% (0.787)
VEGAS−20% (0.786)
VEGAS−sum (0.783)
SimpleM (0.770)
COMBAT (0.774)
Scenario: PVE=0.2, theta0=−4, theta=2
Method (AUC)
RSS−E (0.953)
GATES (0.858)
VEGAS−max (0.860)
VEGAS−10% (0.858)
VEGAS−20% (0.856)
VEGAS−sum (0.850)
SimpleM (0.823)
COMBAT (0.823)
Scenario: PVE=0.6, theta0=−4, theta=2
Method (AUC)
RSS−E (0.973)
GATES (0.919)
VEGAS−max (0.917)
VEGAS−10% (0.915)
VEGAS−20% (0.913)
VEGAS−sum (0.906)
SimpleM (0.862)
COMBAT (0.862)
True positive fraction
False positive fraction
0.00 0.10 0.25 0.50 0.75 0.90 1.00

16. Supplementary Figure 8. Summary of genetic variants. Panel **(a)** reports numbers of all genetic variants available in corresponding GWAS. Panel **(b)** reports numbers of GWAS hits (i.e. loci or SNPs reaching genome-wide significance) reported in corresponding publications. For both panels, “total SNPs” denote SNPs that were available in corresponding publications and/or summary data files (bar charts with higher transparency; blue numbers); “analyzed SNPs” denote SNPs analyzed in the present study (bar charts with lower transparency; black numbers).

(a) Total numbers of SNPs available in corresponding GWAS.

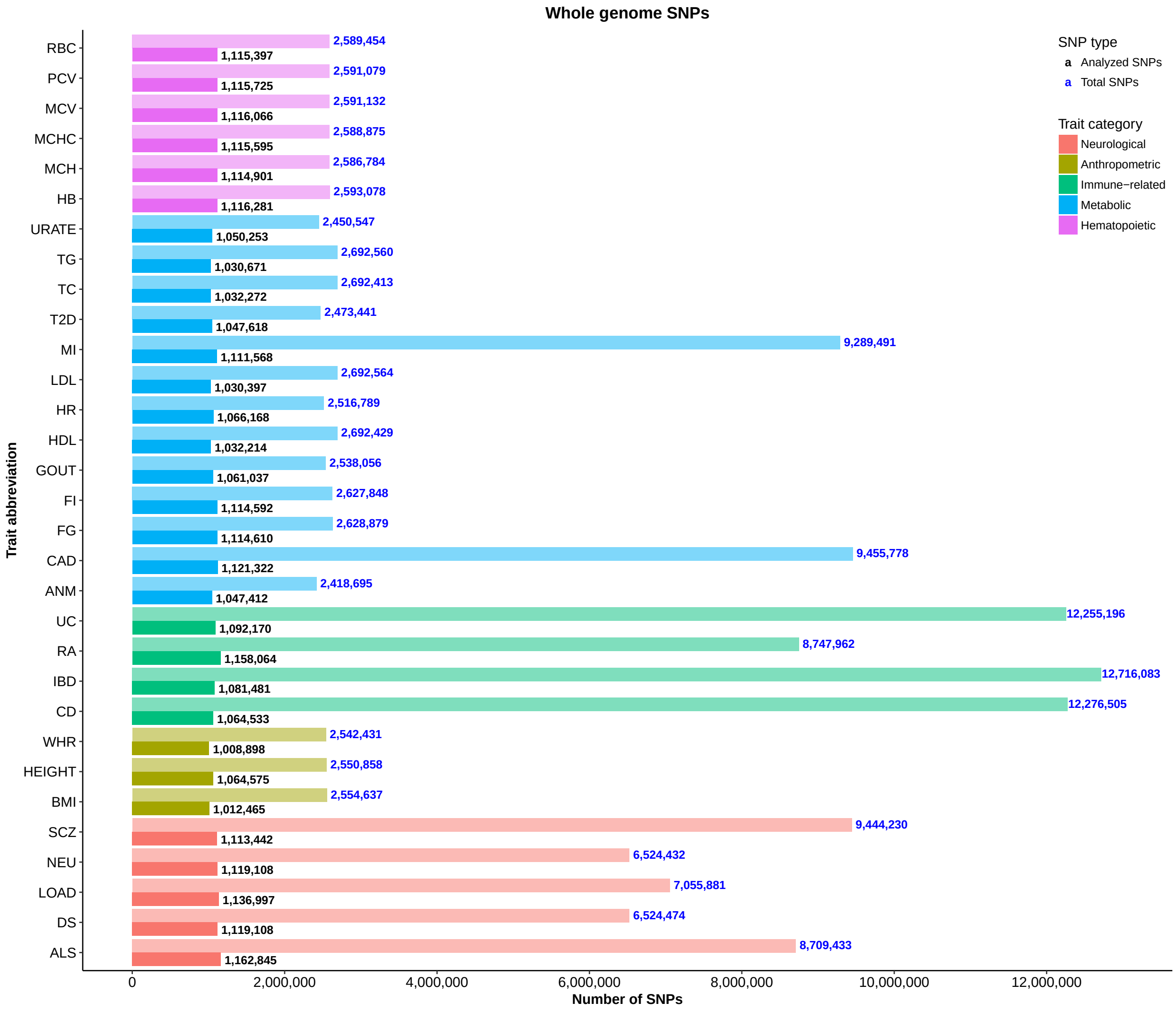

(b) Total numbers of genome-wide significant SNPs reported in corresponding publications ($p < 5 \times 10^{-8}$). The x-axis uses a logarithmic scale (base 10).

Please note that the numbers of "analyzed" genome-wide significant SNPs for body mass index (BMI, [35]) and waist-to-hip ratio (WHR) adjusted for BMI [36] are extremely small. This is because summary data from both studies were combined results of GWAS arrays and custom arrays (e.g. Metabochip, [62]), and we excluded SNPs on custom arrays from our analyses. Custom arrays harbored almost all GWAS hits for these two traits, so there are only **zero** and **one** GWAS hit left for WHR and BMI in our analyses. This explains why the number of "analyzed hits" is **zero** for WHR and **one** for BMI as shown in **Supplementary Figure 12**.

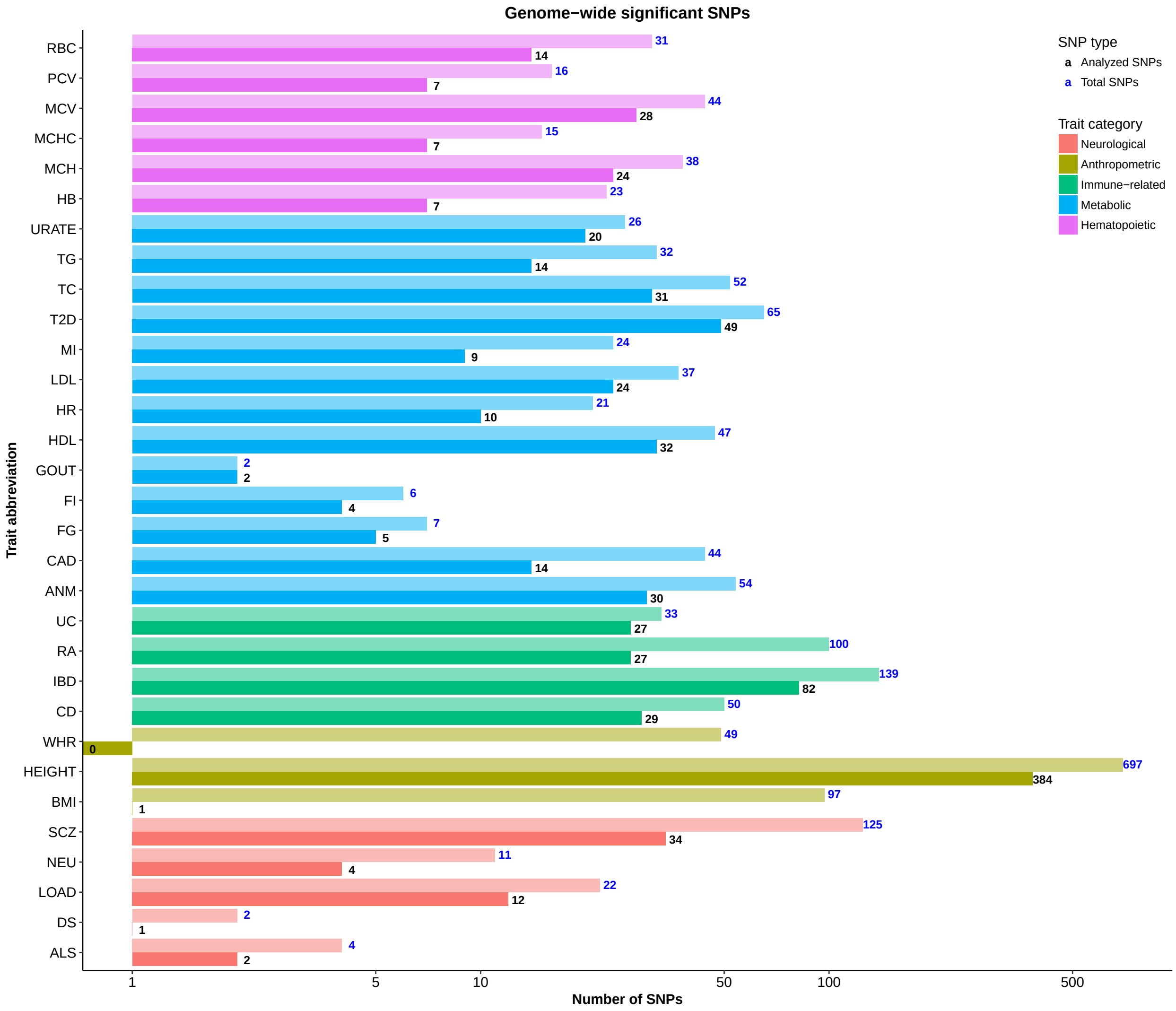

17. Supplementary Figure 9. Inferred effect size distributions of 31 human traits, assuming that no pathways are enriched (i.e. the baseline model $M_0 : \theta = 0$). For each trait, its effect size distribution is summarized as the fraction of trait-associated SNPs and the standardized effect size of trait-associated SNPs. See **Supplementary Notes** for details of these two quantities. Each dot represents a trait, where the horizontal point range indicates the posterior mean and 95% credible interval (C.I.) of fraction of trait-associated SNPs, and the vertical point range indicates the posterior mean and 95% C.I. of standardized effect size. Both axes use a logarithmic scale (base 10). The numerical values of these posterior statistics are provided in **Supplementary Table 2**. The details of "Round 1" and "Round 2" analyses are provided in **Supplementary Table 6**. Note that **Figure 4(a)** is the same as the "Round 2" panel below.

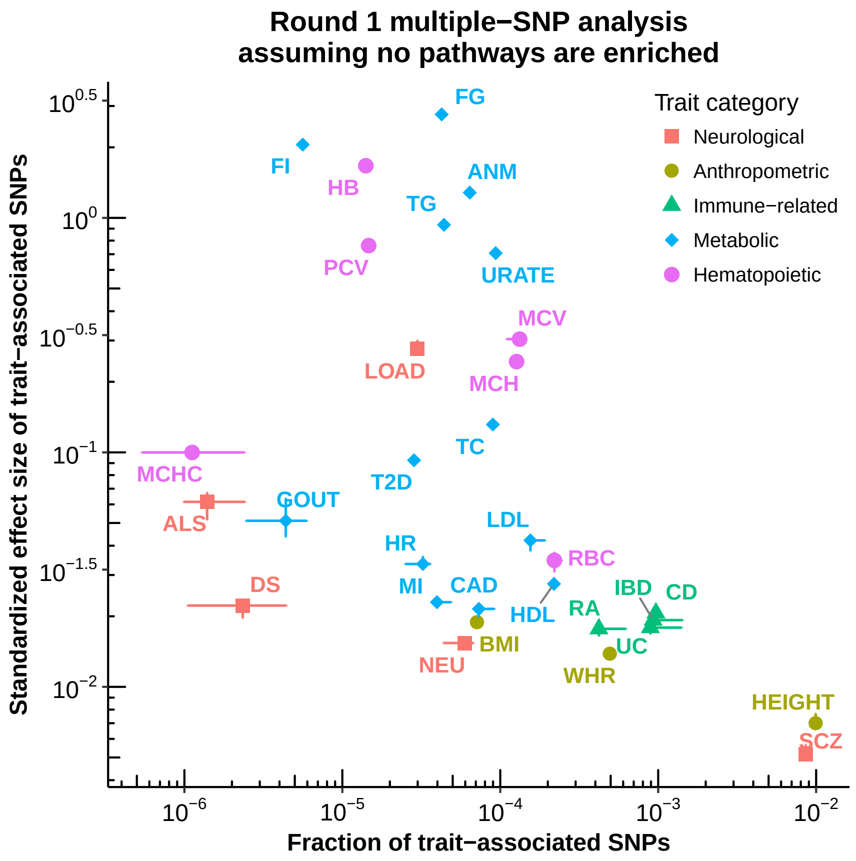
Round 1 multiple−SNP analysis
assuming no pathways are enriched
Standardized effect size of trait−associated SNPs
Fraction of trait−associated SNPs
Trait category
Neurological
Anthropometric
Immune−related
Metabolic
Hematopoietic
FG
FI
HB
ANM
TG
PCV
URATE
MCV
LOAD
MCH
MCHC
TC
T2D
ALS
GOUT
LDL
HR
RBC
DS
MI
CAD
IBD
CD
HDL
RA
BMI
UC
NEU
WHR
HEIGHT
SCZ

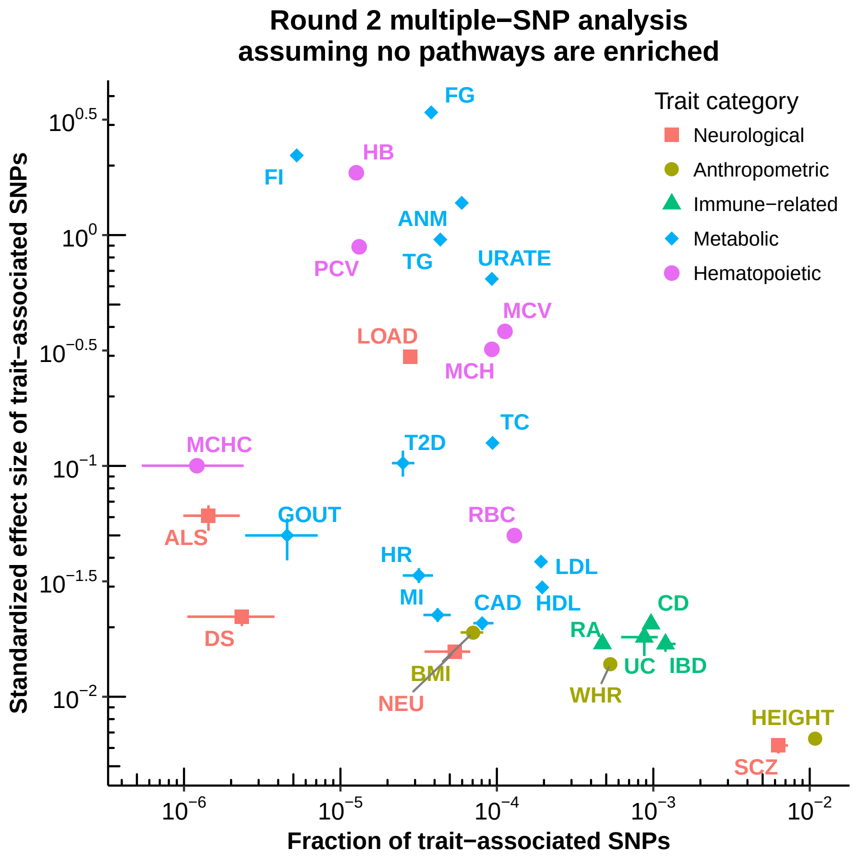
Round 2 multiple−SNP analysis
assuming no pathways are enriched
Standardized effect size of trait−associated SNPs
Fraction of trait−associated SNPs
Trait category
Neurological
Anthropometric
Immune−related
Metabolic
Hematopoietic
FG
HB
FI
ANM
PCV
TG
URATE
MCV
LOAD
MCH
TC
MCHC
T2D
GOUT
RBC
ALS
HR
LDL
MI
CAD
HDL
CD
DS
RA
UC
IBD
BMI
NEU
WHR
HEIGHT
SCZ

18. Supplementary Figure 10. Ranking similarity between genome-wide multiple-SNP and single-SNP analyses, both assuming that no pathways are enriched. We first divide the entire genome into overlapped loci of 50 SNPs (with an overlap of 25 SNPs between neighboring loci). For each trait and each locus, we then use the **same** summary data to compute i) the maximum single-SNP $|z|$-score; ii) the posterior probability that the locus contains at least one trait-associated SNP (P_1); and iii) the posterior expected number of trait-associated SNPs in the locus (ENS). The single-SNP $|z|$-scores are readily available from GWAS summary statistics, whereas the posterior statistics P_1 and ENS are obtained from our Bayesian genome-wide multiple-SNP analyses (under the baseline model $M_0 : \theta = 0$). Based on these three locus-level statistics, we obtain three ranked lists of loci for each trait, and then evaluate their similarity via i) Spearman's ρ statistic; ii) Kendall's τ statistic and iii) rank biased overlap (RBO, [63]). The Spearman's ρ and Kendall's τ statistics are computed by `R` function `cor`. The RBO is computed by the function `rbo` in the package `gespeR` [64]. Each box plot below contains 31 dots, each of which denotes a trait. The details of "Round 1" and "Round 2" analyses are provided in **Supplementary Table 6**.

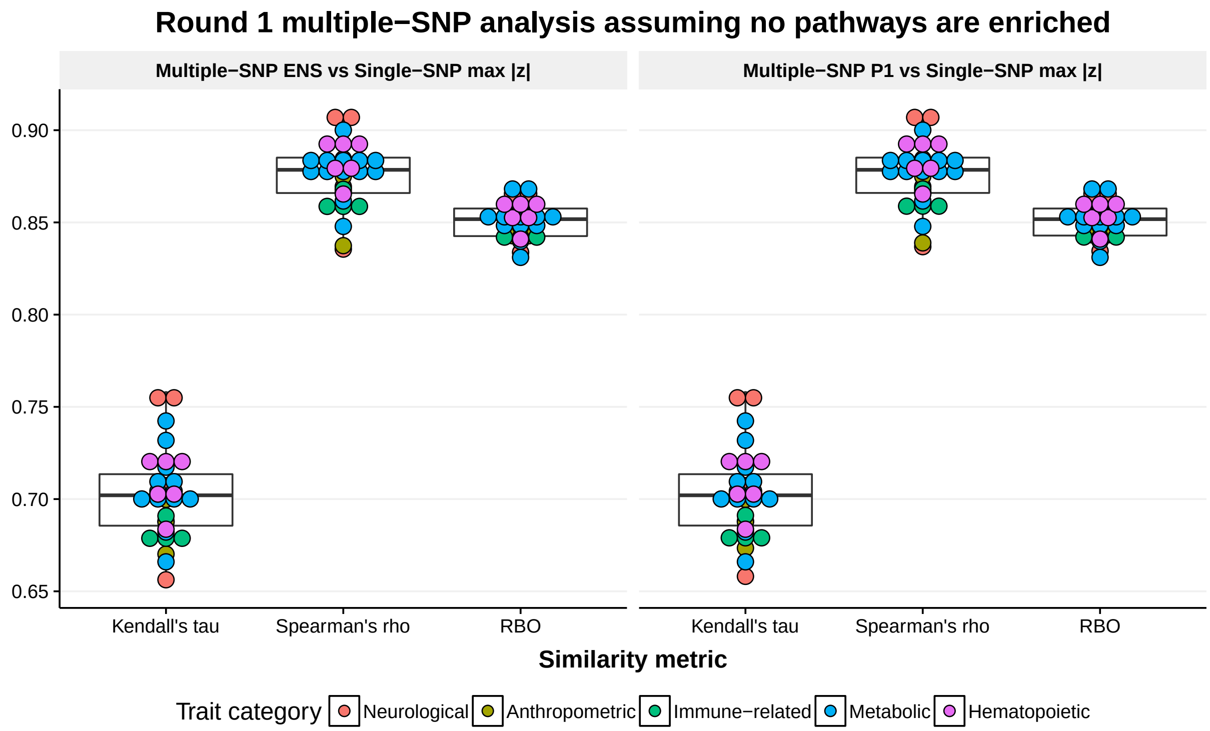
Round 1 multiple−SNP analysis assuming no pathways are enriched
Multiple−SNP ENS vs Single−SNP max |z|
Multiple−SNP P1 vs Single−SNP max |z|
0.90
0.85
0.80
0.75
0.70
0.65
Kendall's tau
Spearman's rho
RBO
Similarity metric
Trait category
Neurological
Anthropometric
Immune−related
Metabolic
Hematopoietic

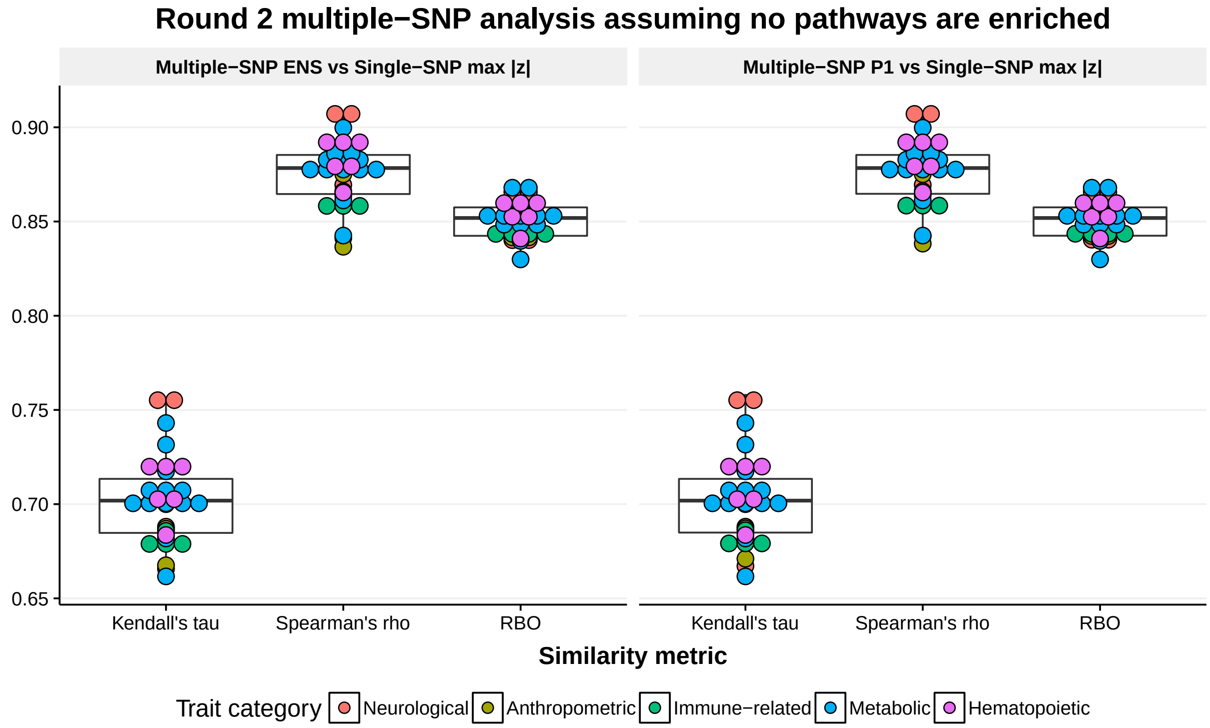
Round 2 multiple−SNP analysis assuming no pathways are enriched
Multiple−SNP ENS vs Single−SNP max |z|
Multiple−SNP P1 vs Single−SNP max |z|
0.90
0.85
0.80
0.75
0.70
0.65
Kendall's tau
Spearman's rho
RBO
Similarity metric
Trait category
Neurological
Anthropometric
Immune−related
Metabolic
Hematopoietic

19. Supplementary Figure 11. Concordance between genome-wide single-SNP and multiple-SNP analyses of 31 phenotypes, both assuming that no pathways are enriched (i.e. the baseline model $M_0 : \theta = 0$). For a given trait, the concordance between single-SNP and multiple-SNP analyses is measured by the proportion of "single-SNP hits" covered by "multiple-SNP signal loci" (y-axis). The "single-SNP hits" are SNPs reaching significance (for a given p-value threshold shown in x-axis) and separated by at least 1 Mb. The "multiple-SNP signal loci" are predefined genomic regions satisfying certain criteria (estimated $P_1 > 0.5$, $P_1 > 0.9$ or ENS > 1). For each trait, both single-SNP and multiple-SNP analyses are performed on the **same** summary-level data. See **Supplementary Figure 10** for the definition of locus and multiple-SNP posterior statistics (P_1 and ENS). See **Supplementary Table 6** for the details of "Round 1" and "Round 2" analyses.

19.1. *Heart-related traits.* The three heart-related traits are heart rate [38], coronary artery disease and myocardial infarction [18].

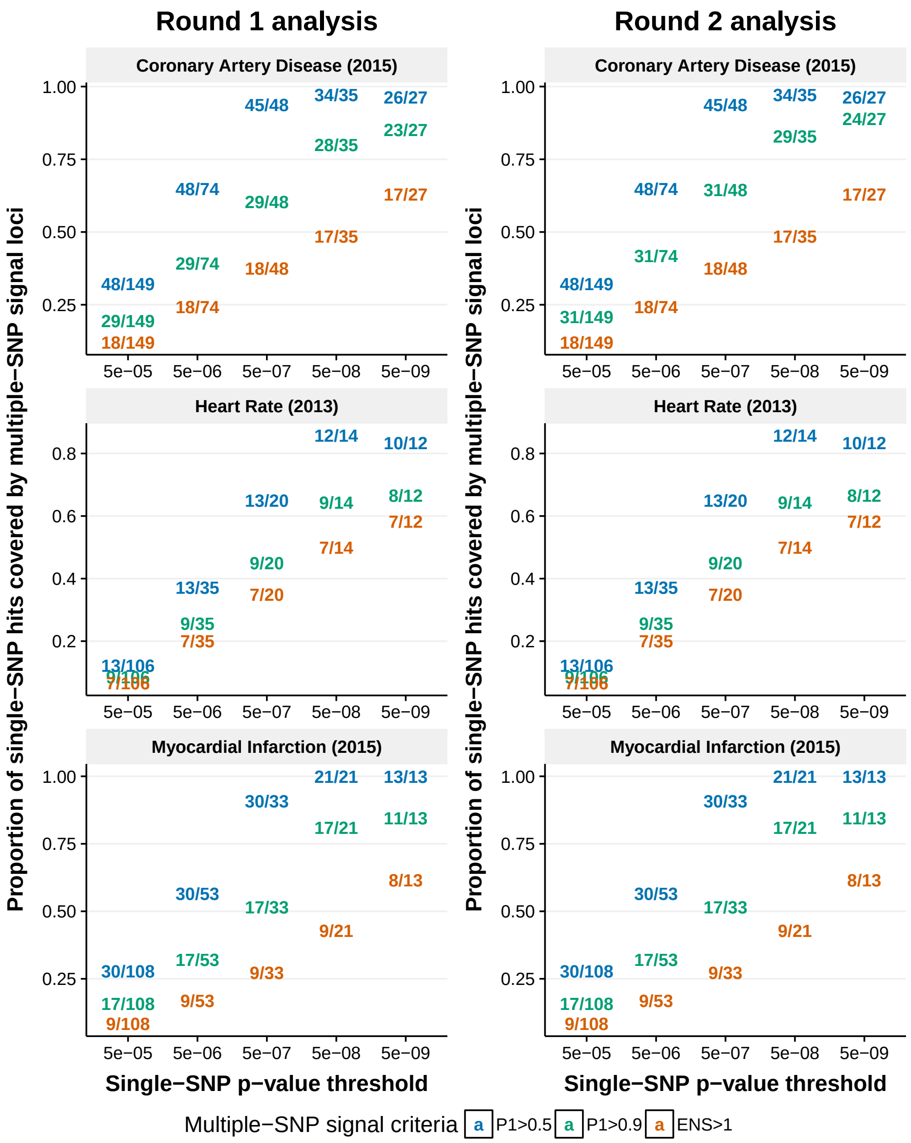

19.2. *Anthropometric traits.* The three anthropometric traits are adult height [5], body mass index [35] and waist-to-hip ratio after adjusting for body mass index [36].

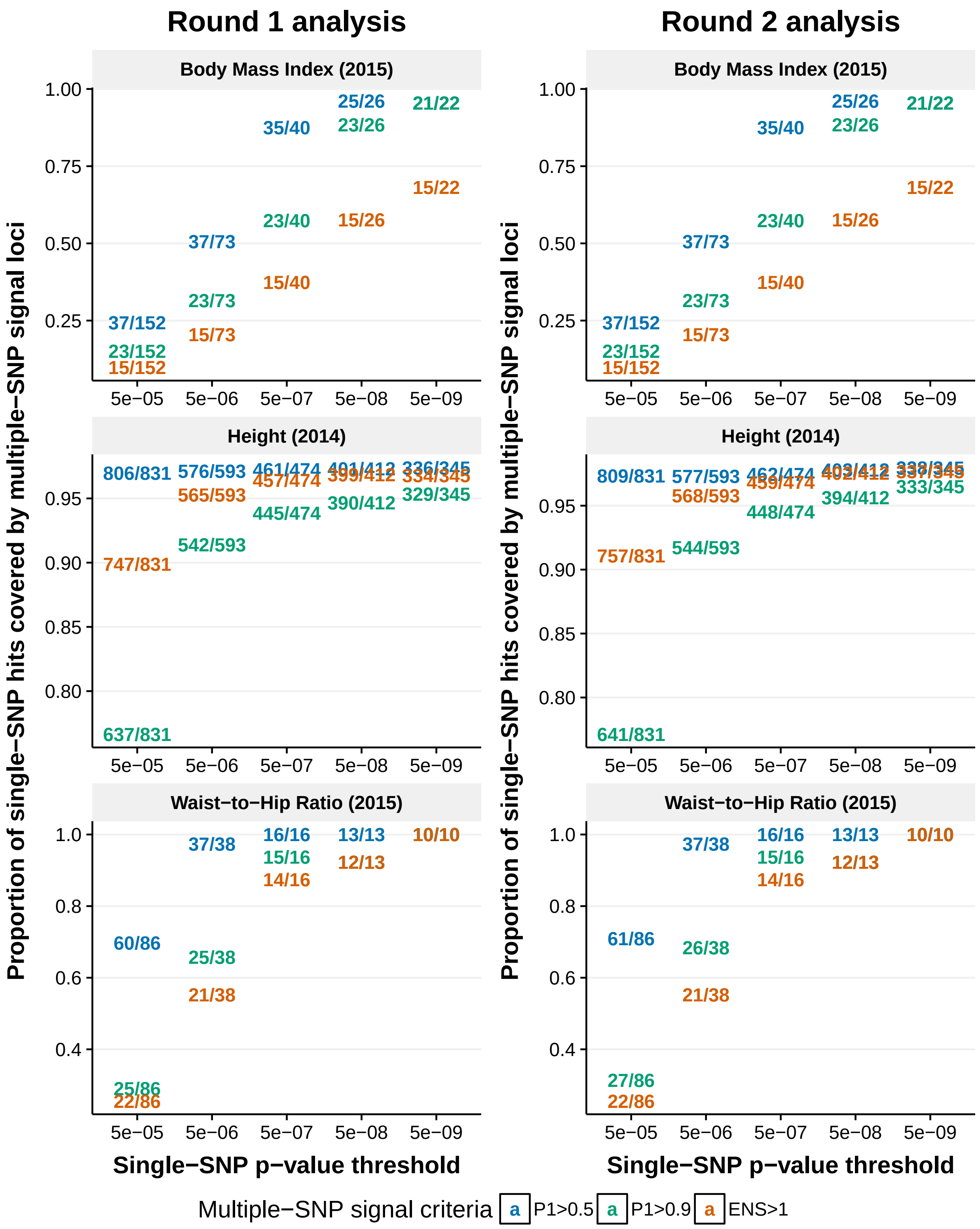

19.3. *Immune-related traits.* The four immune-related traits are rheumatoid arthritis [26], inflammatory bowel disease, Crohn's disease and ulcerative colitis [11].

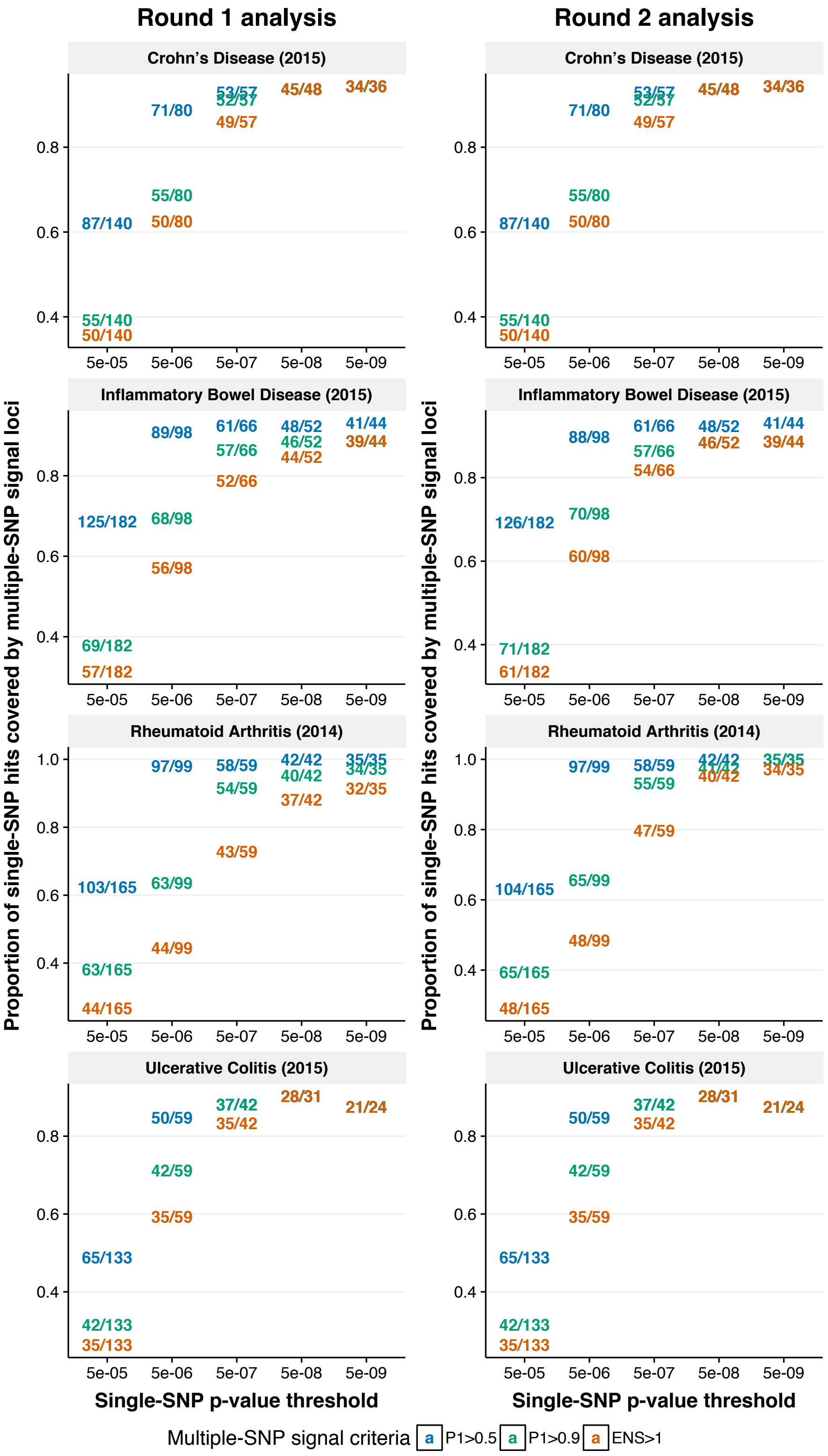

19.4. *Blood lipid traits.* The four blood lipid traits are total cholesterol, low-density lipoprotein cholesterol, high-density lipoprotein cholesterol and triglycerides [22].

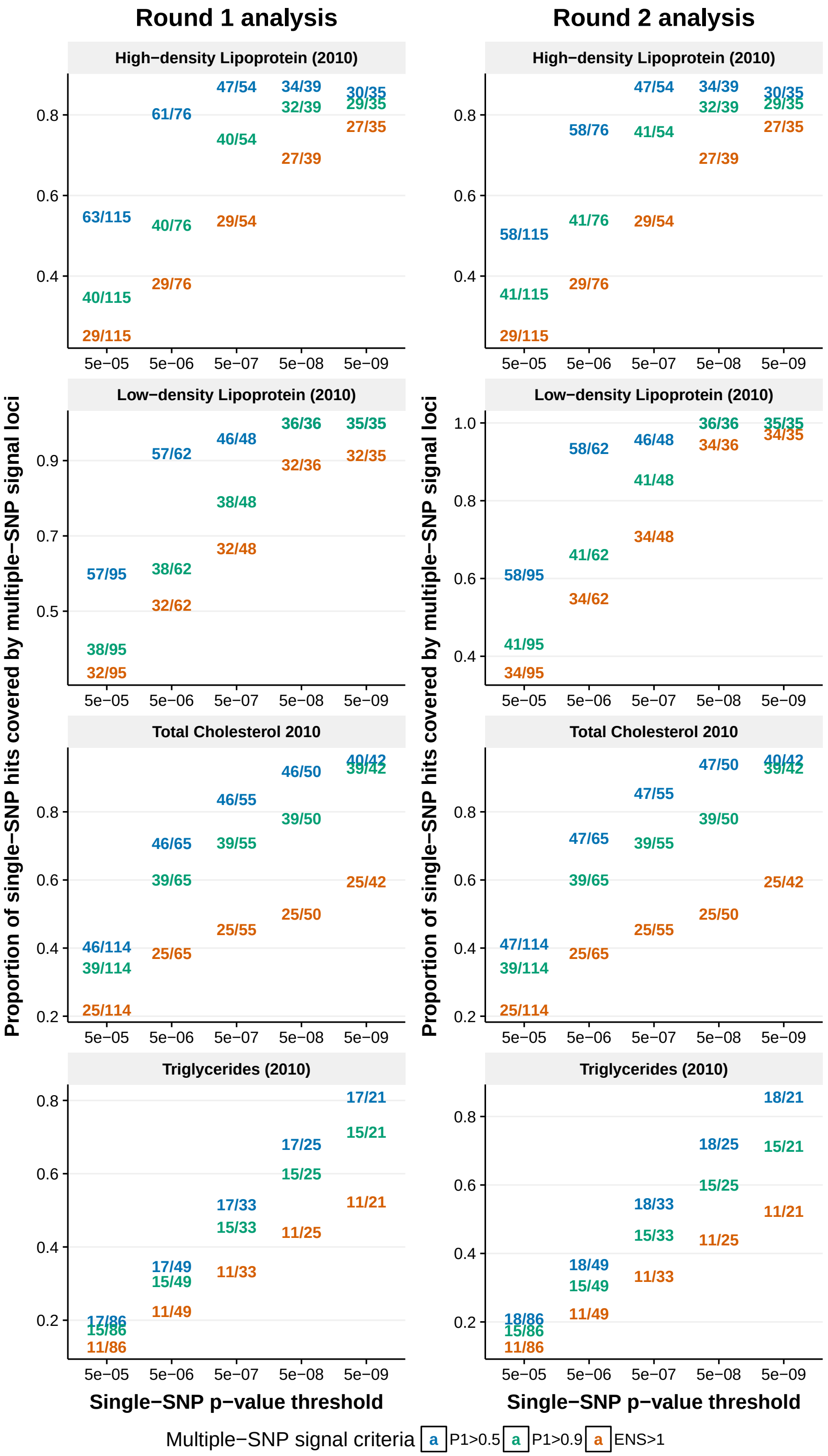

19.5. *Hematopoietic traits.* The six red blood cell traits are haemoglobin, mean cell haemoglobin, mean cell haemoglobin concentration, mean cell volume, packed cell volume and red blood cell count [46].

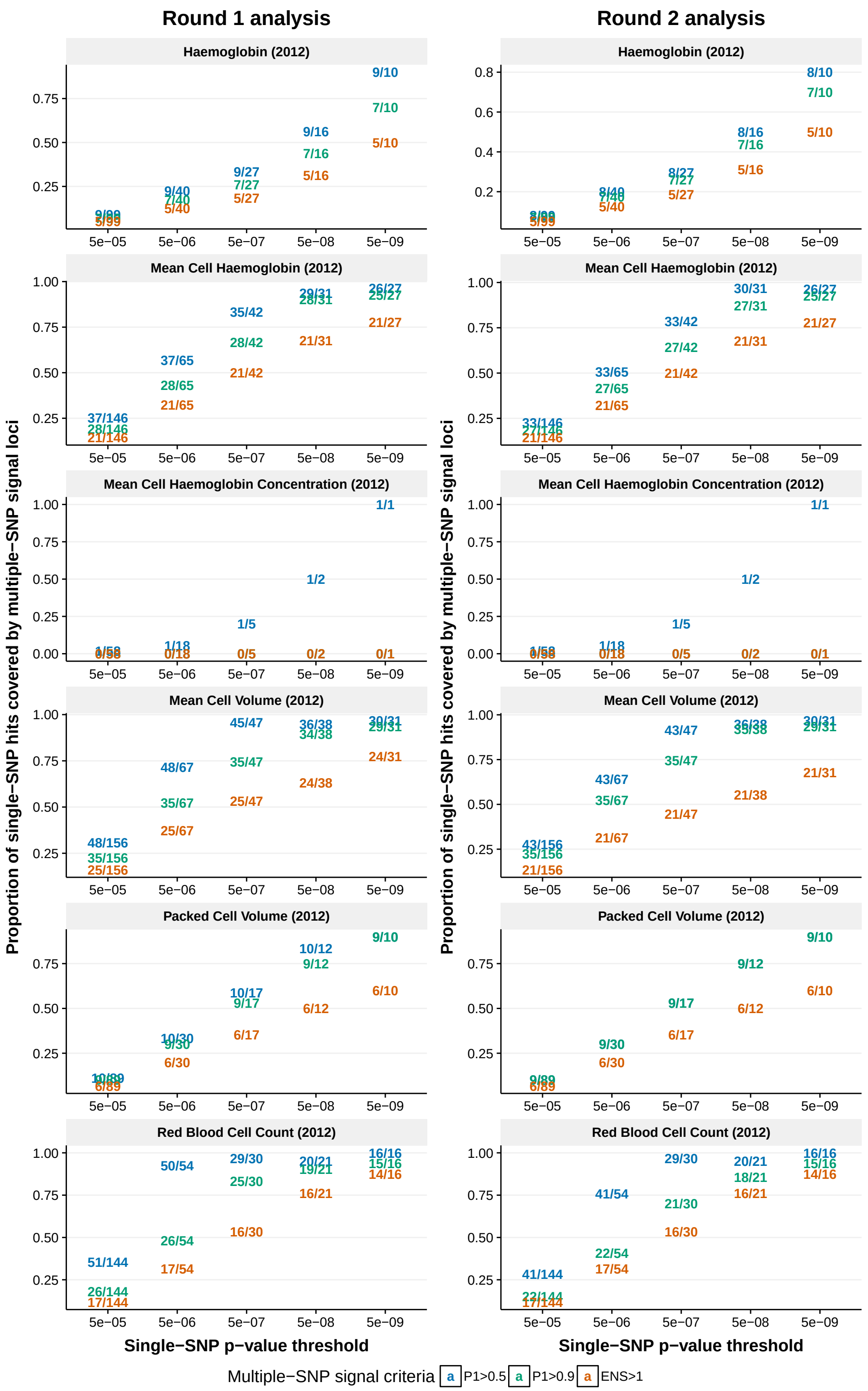

19.6. *Neurological phenotypes.* The five neurological phenotypes: Alzheimer's disease [39], schizophrenia [37], amyotrophic lateral sclerosis [45], depressive symptoms and neuroticism [40].

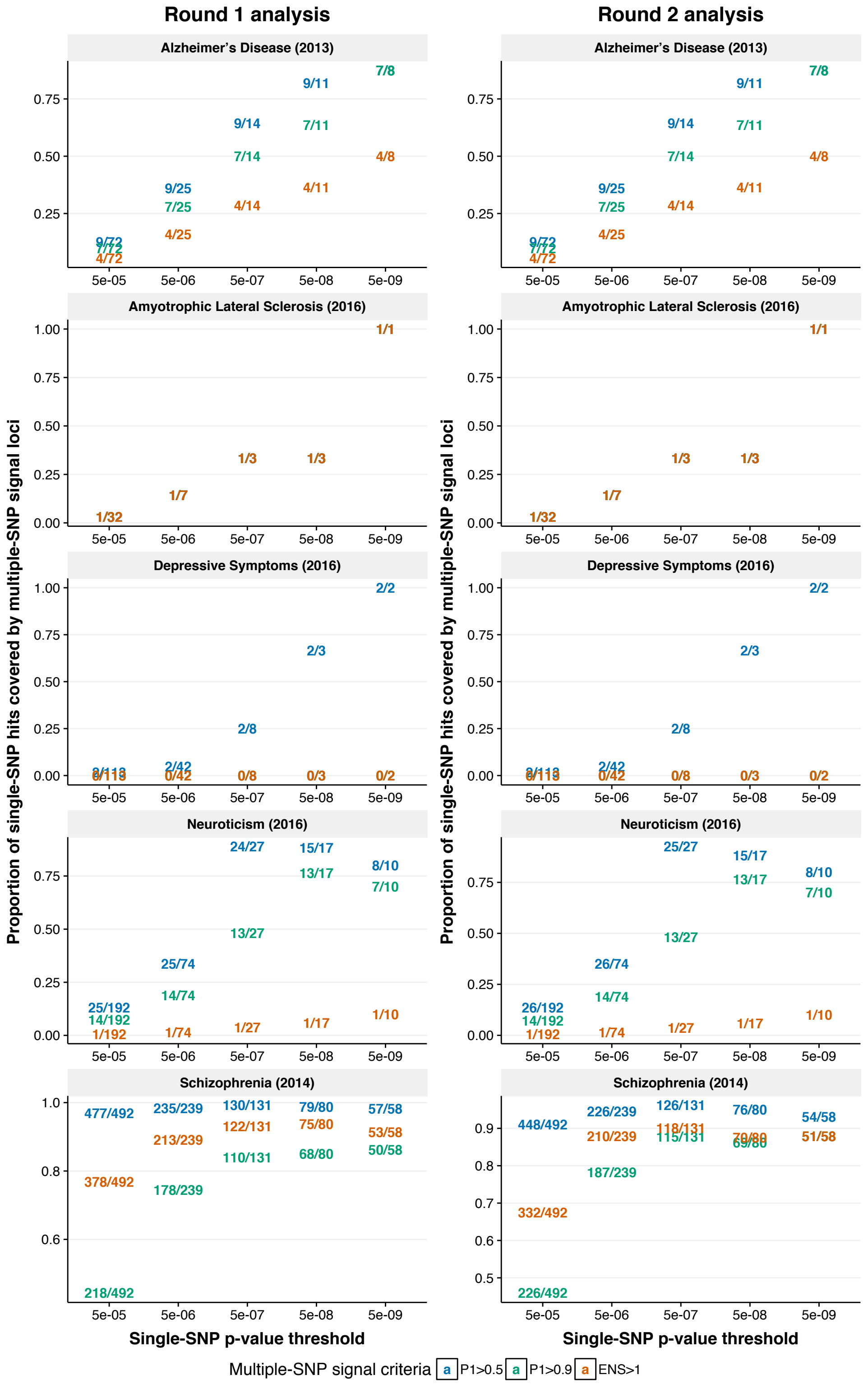

19.7. *Insulin-related traits.* The three insulin-related traits are fasting glucose, fasting insulin [44] and type 2 diabetes [41].

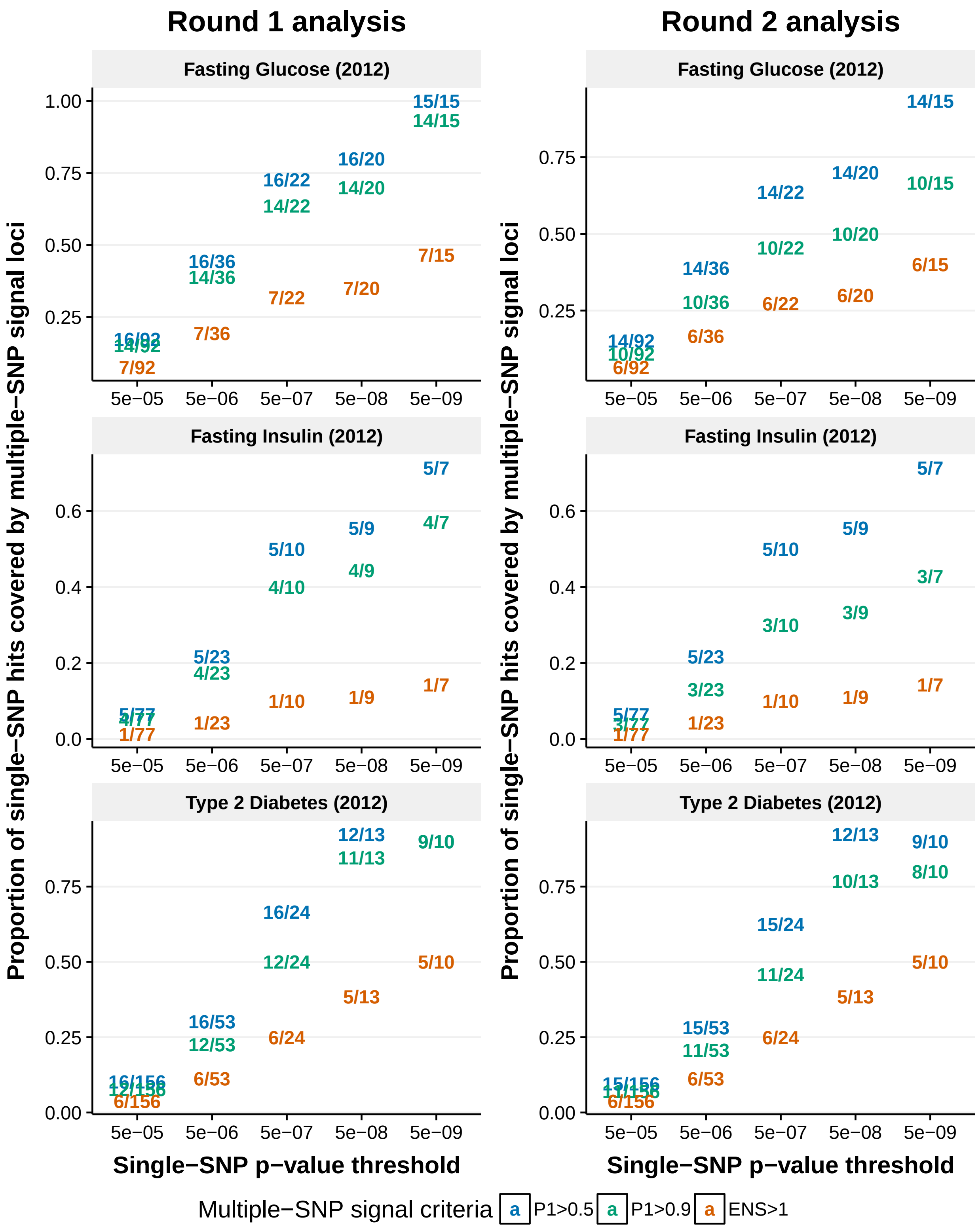

19.8. *Other traits.* The three remaining traits are serum urate, gout [43] and age at natural menopause [42].

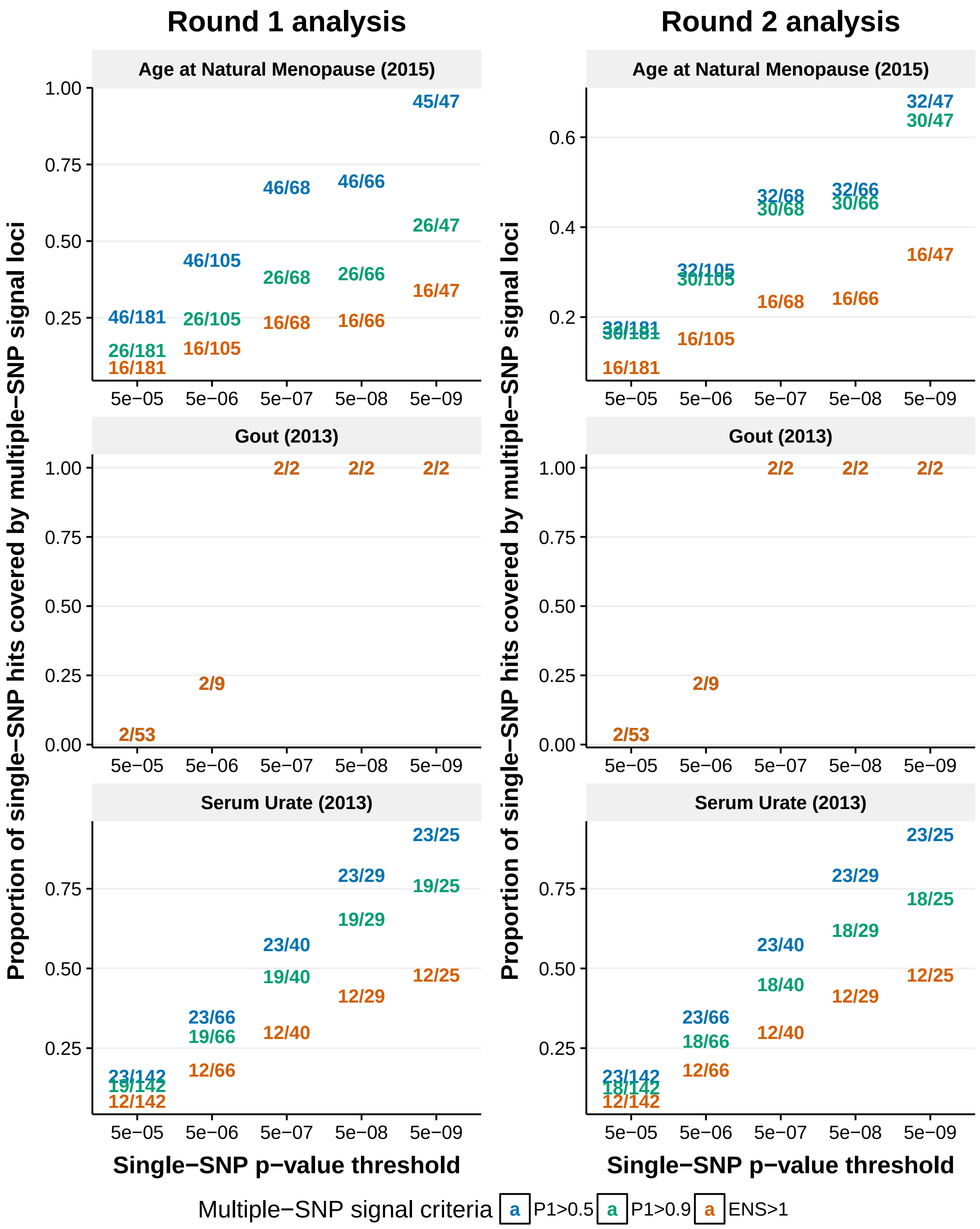

20. Supplementary Figure 12. Proportion of previously-reported genome-wide significant variants that are detected by genome-wide multiple-SNP analyses, assuming that no pathways are enriched. For each phenotype, we manually extract the genome-wide significant variants (a.k.a. GWAS hits) from corresponding publications (e.g. Tables, Supplementary Files). This is different from **Supplementary Figure 11**, where we derive GWAS hits from the raw summary statistics of the same set of SNPs that were used in our Bayesian multiple-SNP analyses. We call a GWAS hit "detected by multiple-SNP analyses" if this SNP is covered by a predefined locus satisfying certain multiple-SNP association criteria (estimated $P_1 > 0.5$, $P_1 > 0.9$ or ENS > 1). See **Supplementary Figure 10** for the definitions of locus and the multiple-SNP association posterior statistics. For each panel, phenotypes are ordered first by trait category, then the number of reported GWAS hits.

It is important to note that some published GWAS hits of certain traits are not necessarily (genome-wide) significant in the corresponding summary data file. For example, rs34856868 shows $p = 9.80 \times 10^{-9}$ for association with inflammatory bowel disease in Table 2 of [11]; however, the p value of the same SNP is 0.27 in the corresponding summary data file (`EUR.IBD.gwas.assoc.gz`). For this SNP, the result in Table 2 of [11] was indeed obtained from a combined analysis of data on both GWAS and custom arrays (Immunochip, [65]), whereas the result in the summary data file was only based on GWAS arrays. Because of this type of potential discrepancy between summary data files and corresponding publications, the concordance rates shown here are lower than those shown in **Supplementary Figure 11** for certain traits.

Note that **Supplementary Figure 12** is included to highlight the need for careful usage of GWAS summary statistics. To fairly assess the concordance between traditional single-SNP analyses and our multiple-SNP analyses, please use **Supplementary Figure 11**.

20.1. *All hits* & $P_1 > 0.5$. Proportion of all previous GWAS hits that are covered by loci with $P_1 > 0.5$.

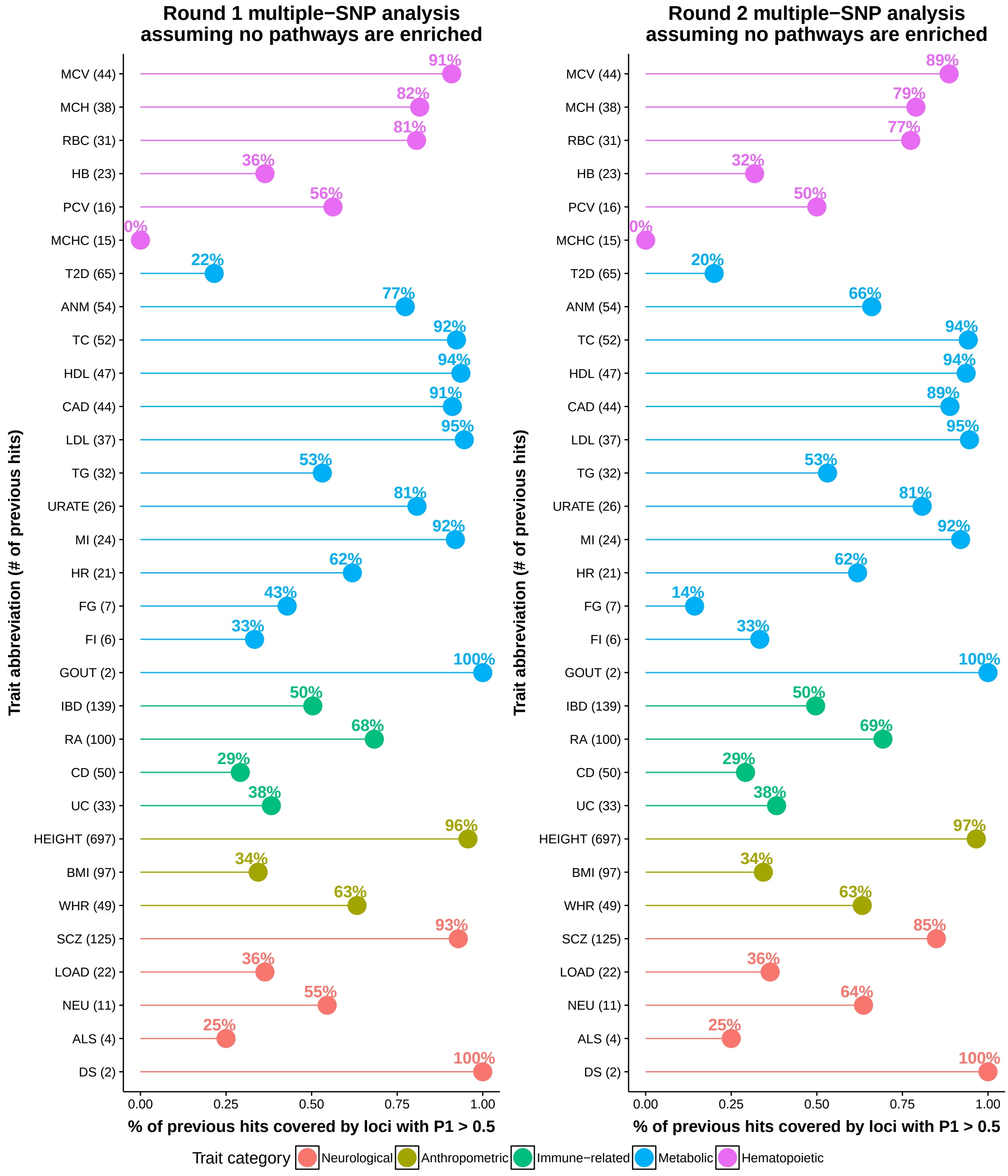

20.2. *Analyzed hits & $P_1 > 0.5$.* Proportion of previous GWAS hits that are included in genome-wide multiple-SNP analyses and are covered by loci with $P_1 > 0.5$.

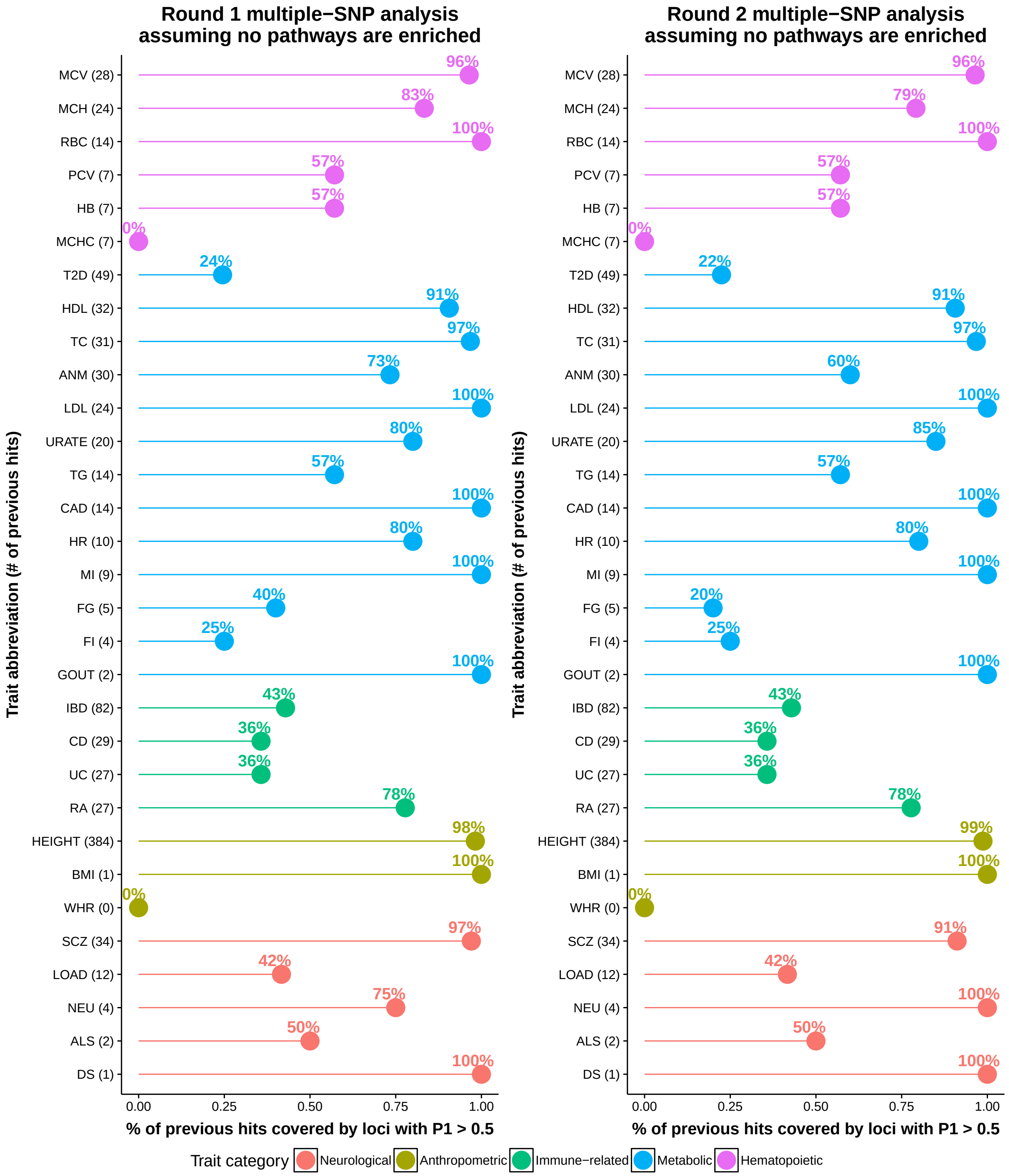

20.3. *All hits* & $P_1 > 0.9$. Proportion of all previous GWAS hits that are covered by loci with $P_1 > 0.9$.

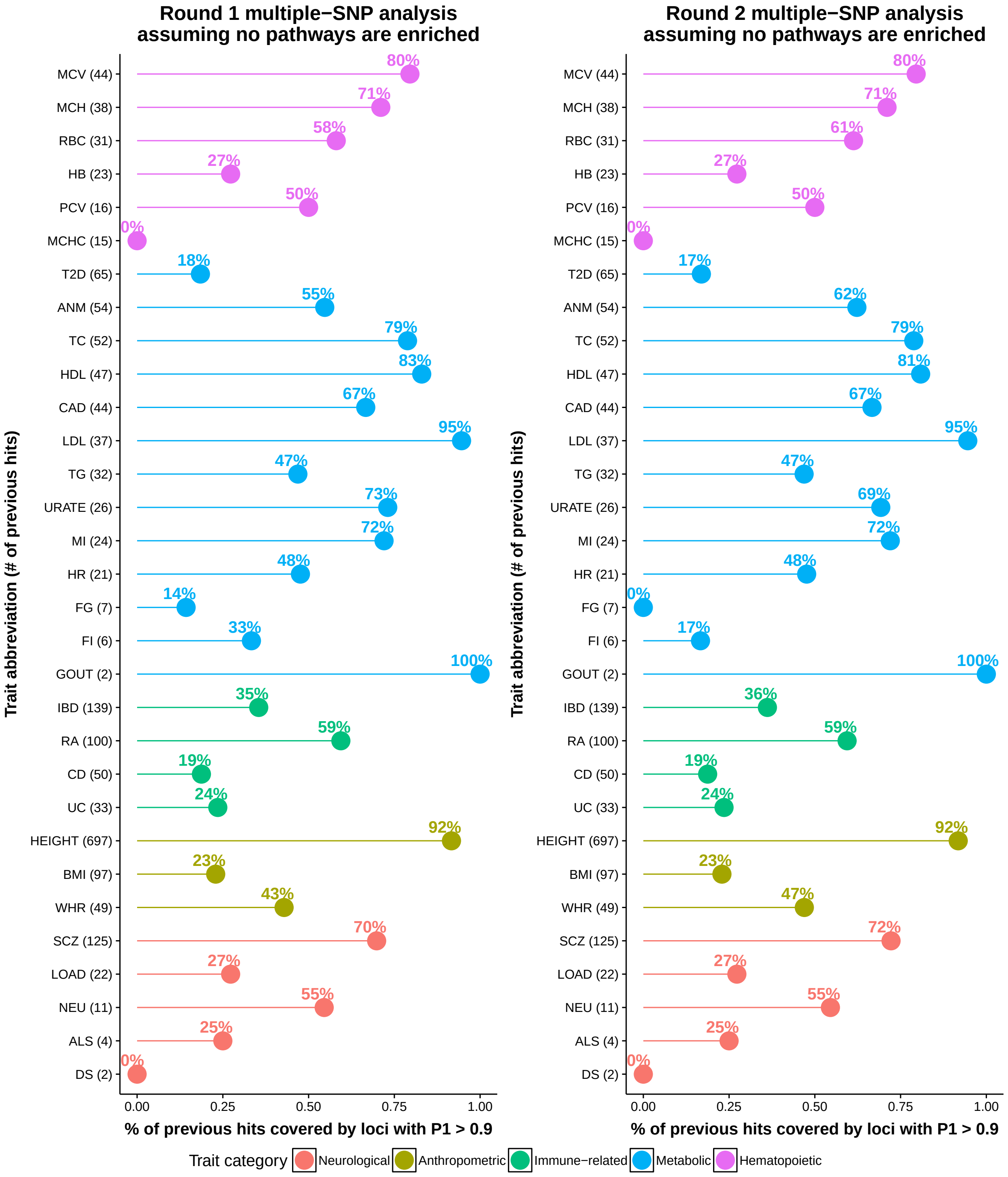

20.4. *Analyzed hits & $P_1 > 0.9$.* Proportion of all previous GWAS hits that that are included in genome-wide multiple-SNP analyses and are covered by loci with $P_1 > 0.9$.

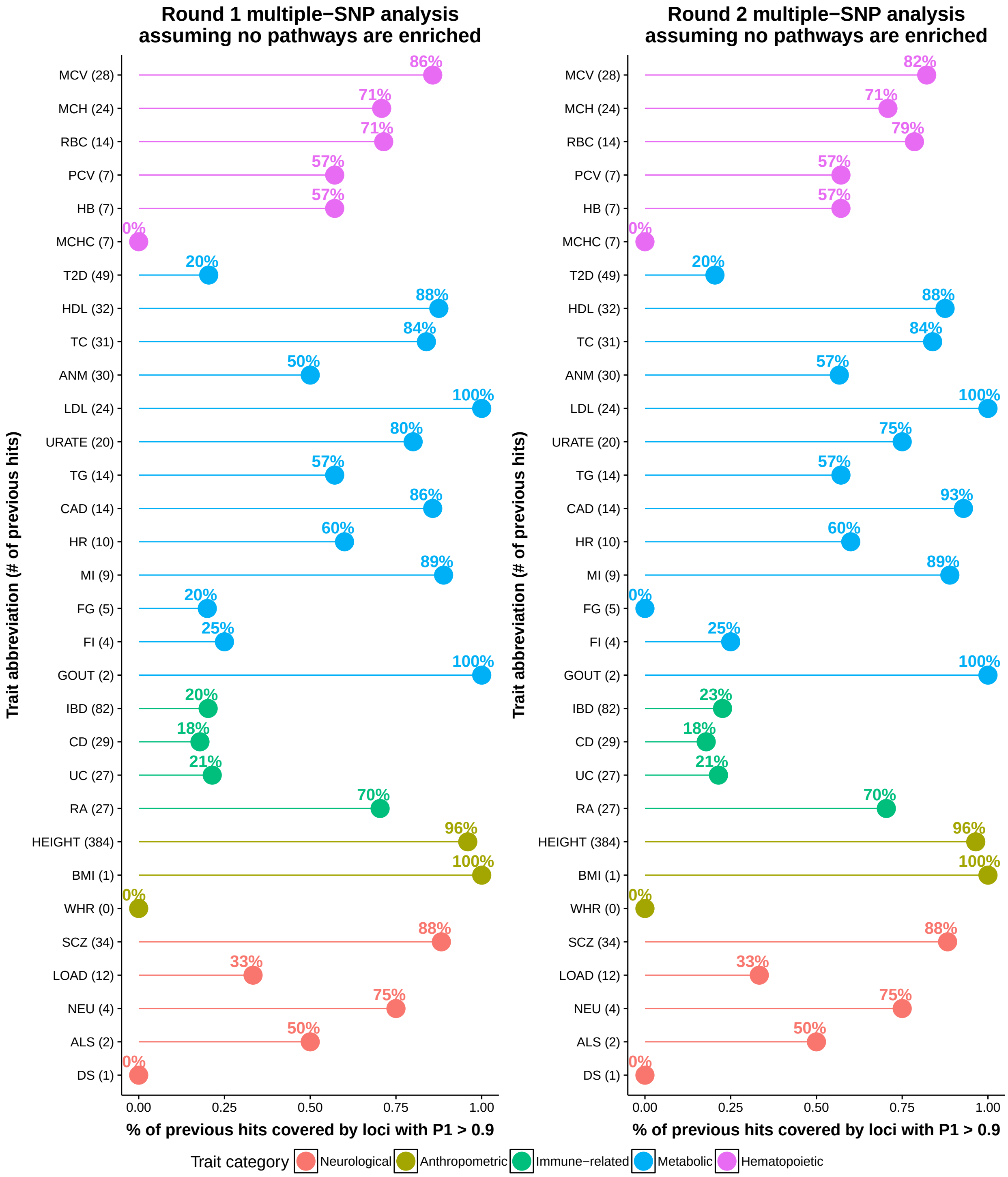

20.5. *All hits & ENS* > 1. Proportion of all previously-reported GWAS hits that are covered by loci with ENS > 1.

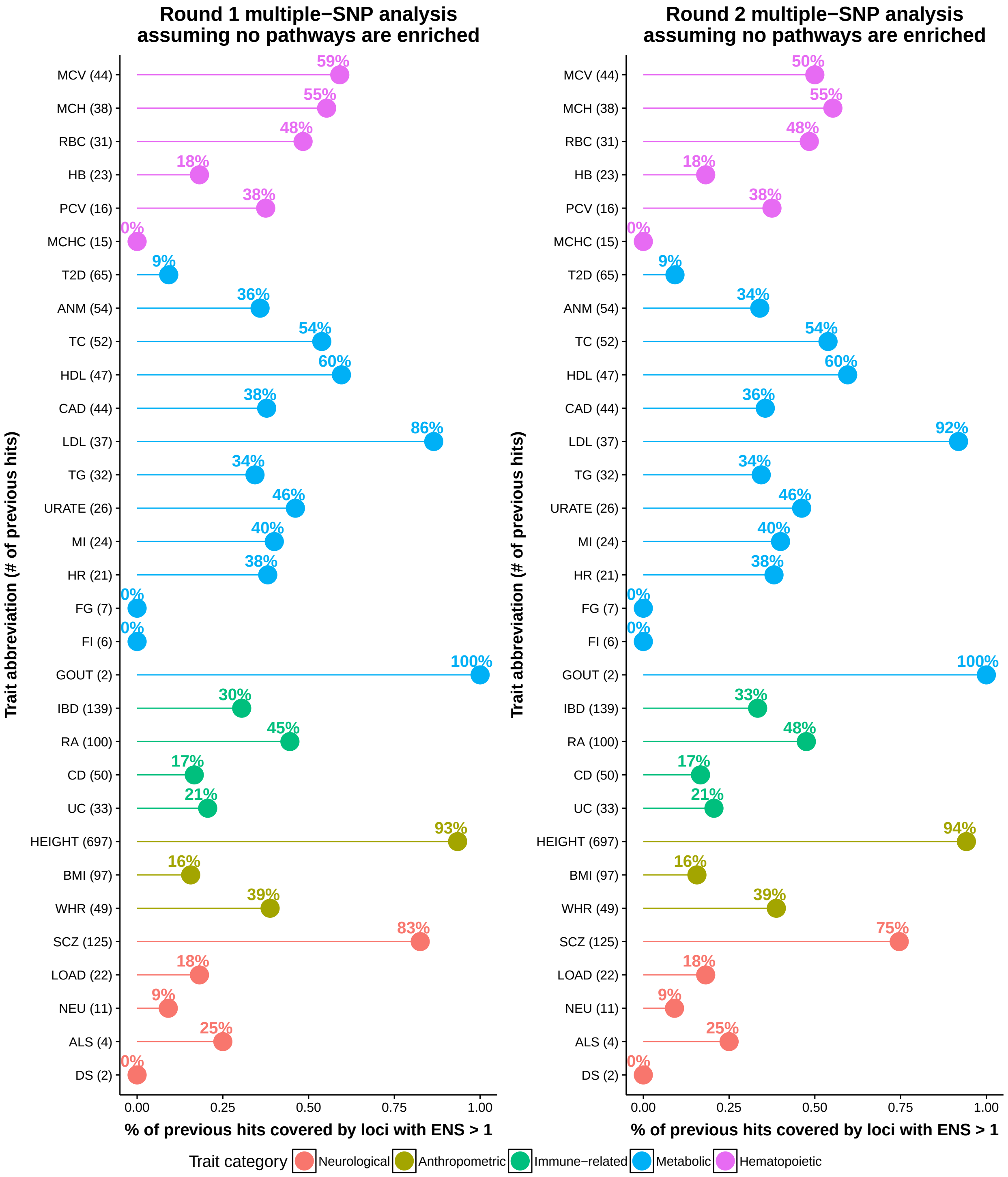

20.6. *Analyzed hits & ENS* > 1. Proportion of previously-reported GWAS hits that are included in genome-wide multiple-SNP analyses and are covered by loci with ENS > 1.

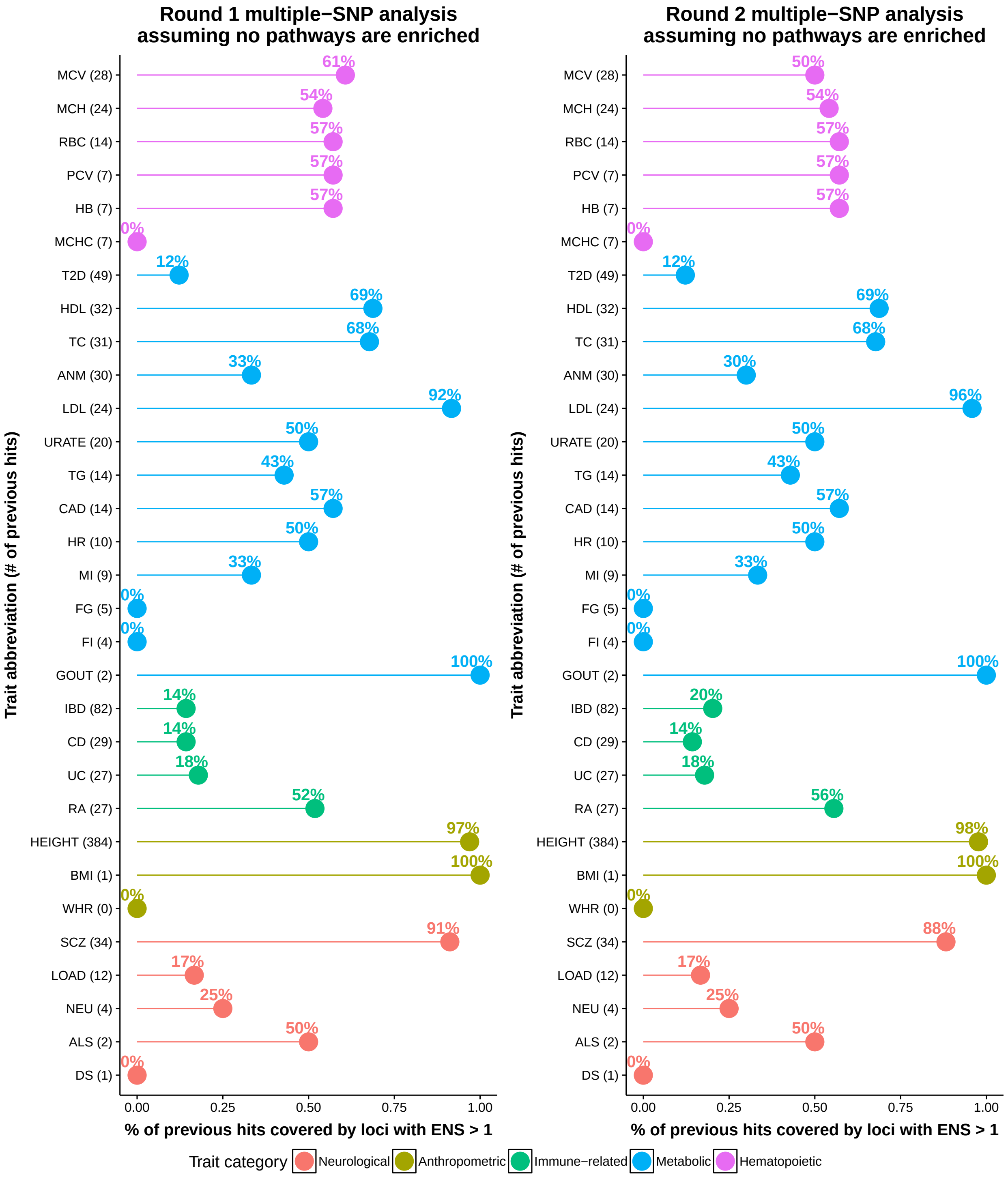

21. Supplementary Figure 13. Compare the number of signals from genome-wide multiple-SNP and single-SNP analyses, both assuming that no pathways are enriched (i.e. the baseline model $M_0 : \theta = 0$). Both axes use a logarithmic scale (base 10). Dashed lines are reference lines with intercept zero and slope one. Each point range along y-axis denotes the posterior mean and 95% credible interval of the posterior expected total number of trait-associated SNPs (ENS). See **Supplementary Notes** for the detail of computing ENS. As in **Supplementary Figure 12**, here the "hits from single-SNP analysis" are the genome-wide significant SNPs reported in corresponding publications.

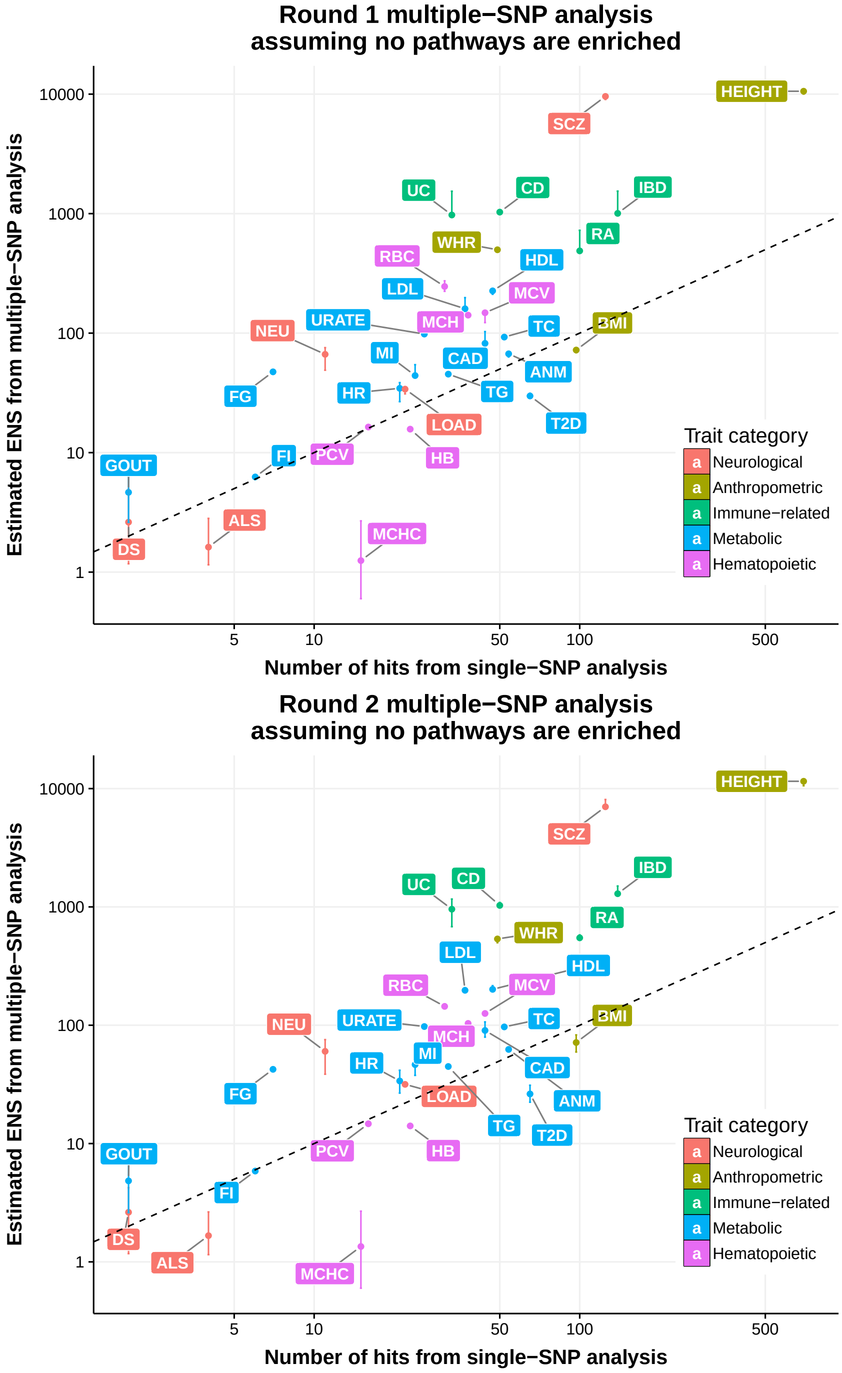

22. Supplementary Figure 14. Proportion of loci identified by genome-wide multiple-SNP analyses that are at least 1 Mb away from previously-reported GWAS hits, assuming that no pathways are enriched (i.e. the baseline model $M_0 : \theta = 0$). We call a predefined locus "detected by multiple-SNP analyses" if the locus satisfies certain multiple-SNP association criteria (estimated $P_1 > 0.5$, $P_1 > 0.9$ or ENS > 1). See **Supplementary Figure 10** for the definition of locus and the multiple-SNP association posterior statistics. See **Supplementary Figure 12** for the definition of "previously-reported GWAS hits". For each panel, phenotypes are ordered first by category, then the number of loci identified from multiple-SNP analyses.

22.1. *All hits & $P_1 > 0.5$.* Proportion of loci with $P_1 > 0.5$ that are at least 1 Mb away from all previously-reported GWAS hits.

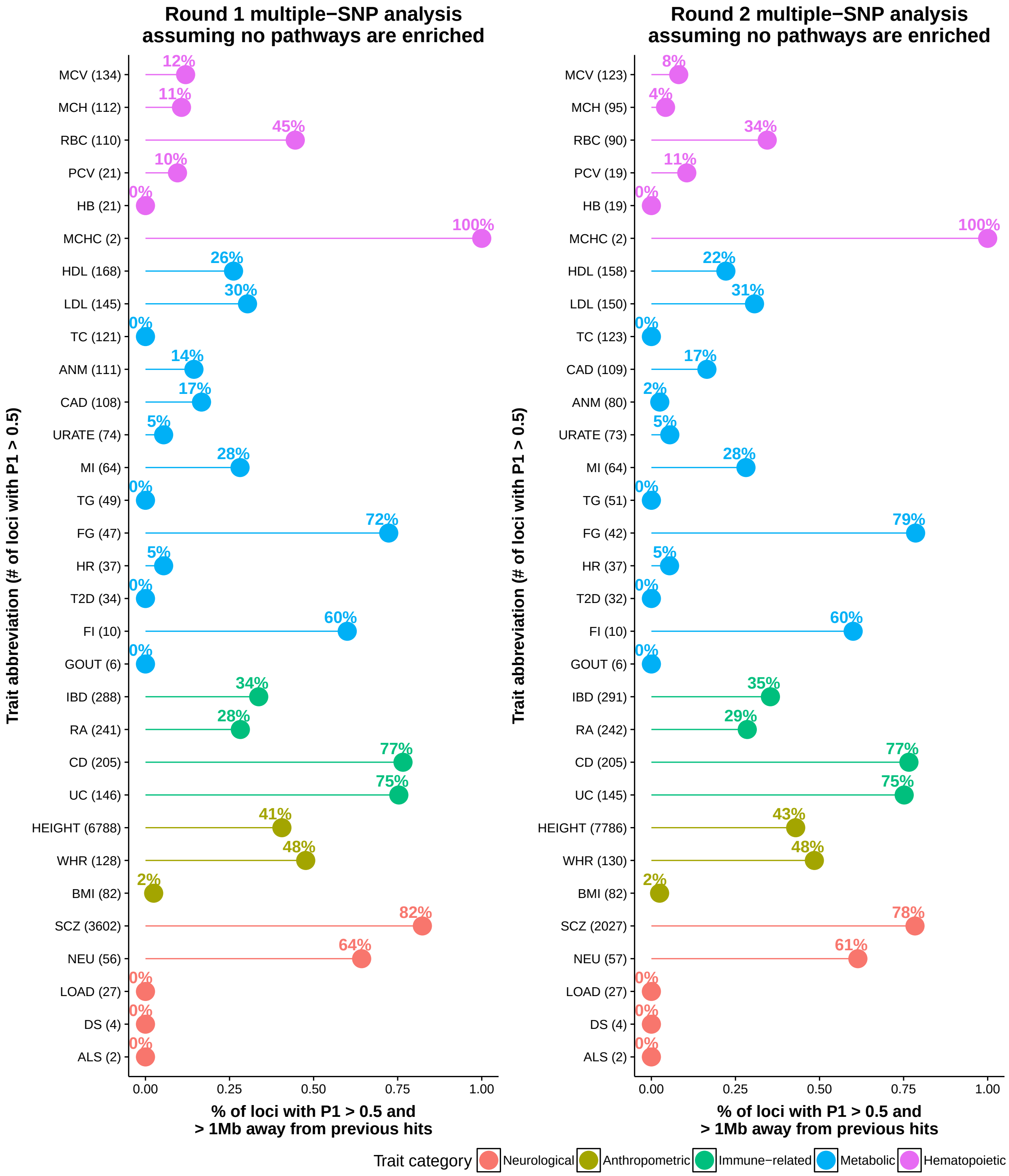

22.2. *Analyzed hits & $P_1 > 0.5$.* Proportion of loci with $P_1 > 0.5$ that are at least 1 Mb away from previously-reported GWAS hits that are included in genome-wide multiple-SNP analyses.

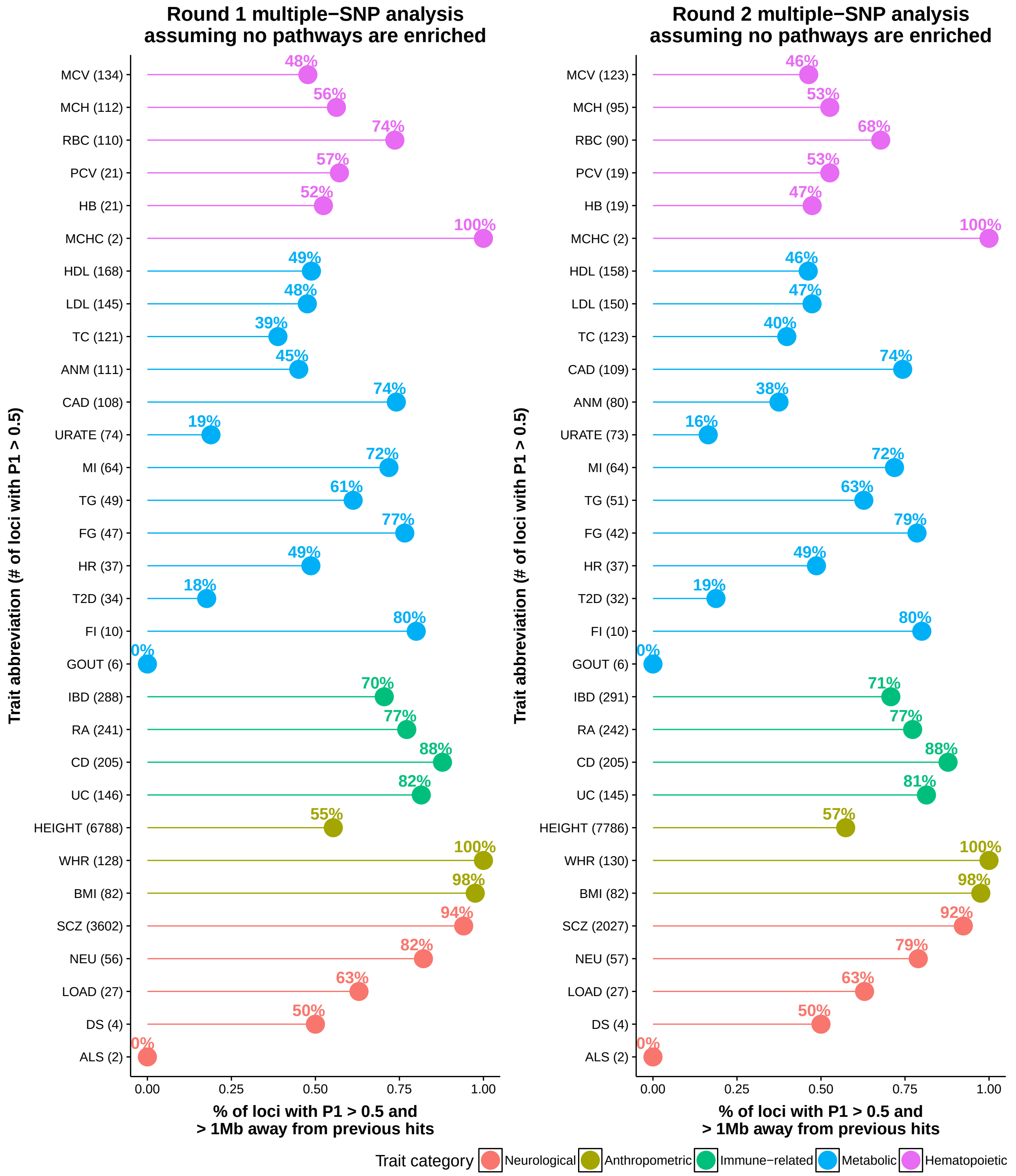

22.3. *All hits & $P_1 > 0.9$.* Proportion of loci with $P_1 > 0.9$ that are at least 1 Mb away from all previously-reported GWAS hits.

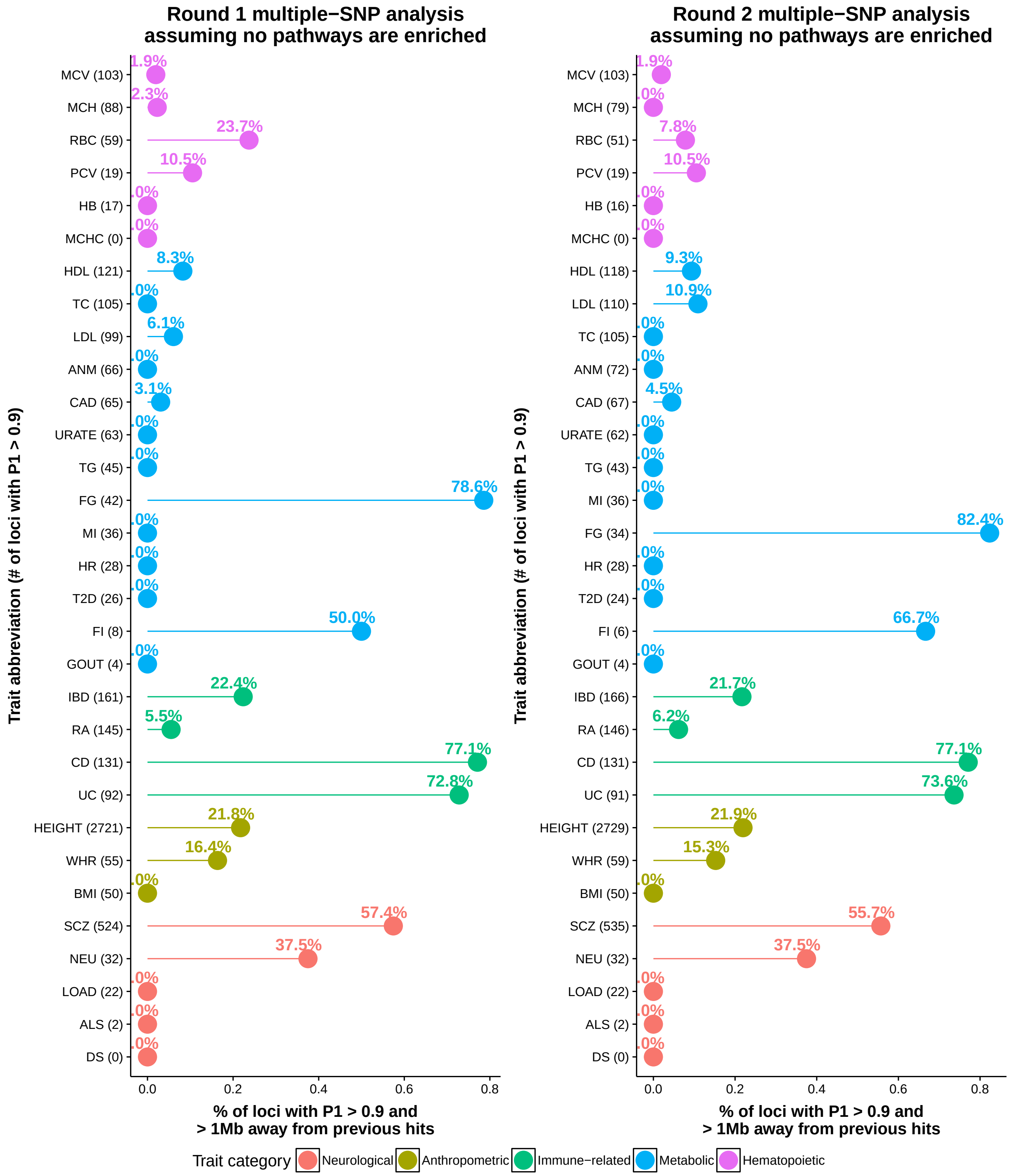

22.4. *Analyzed hits & $P_1 > 0.9$.* Proportion of loci with $P_1 > 0.9$ that are at least 1 Mb away from previously-reported GWAS hits that are included in genome-wide multiple-SNP analyses.

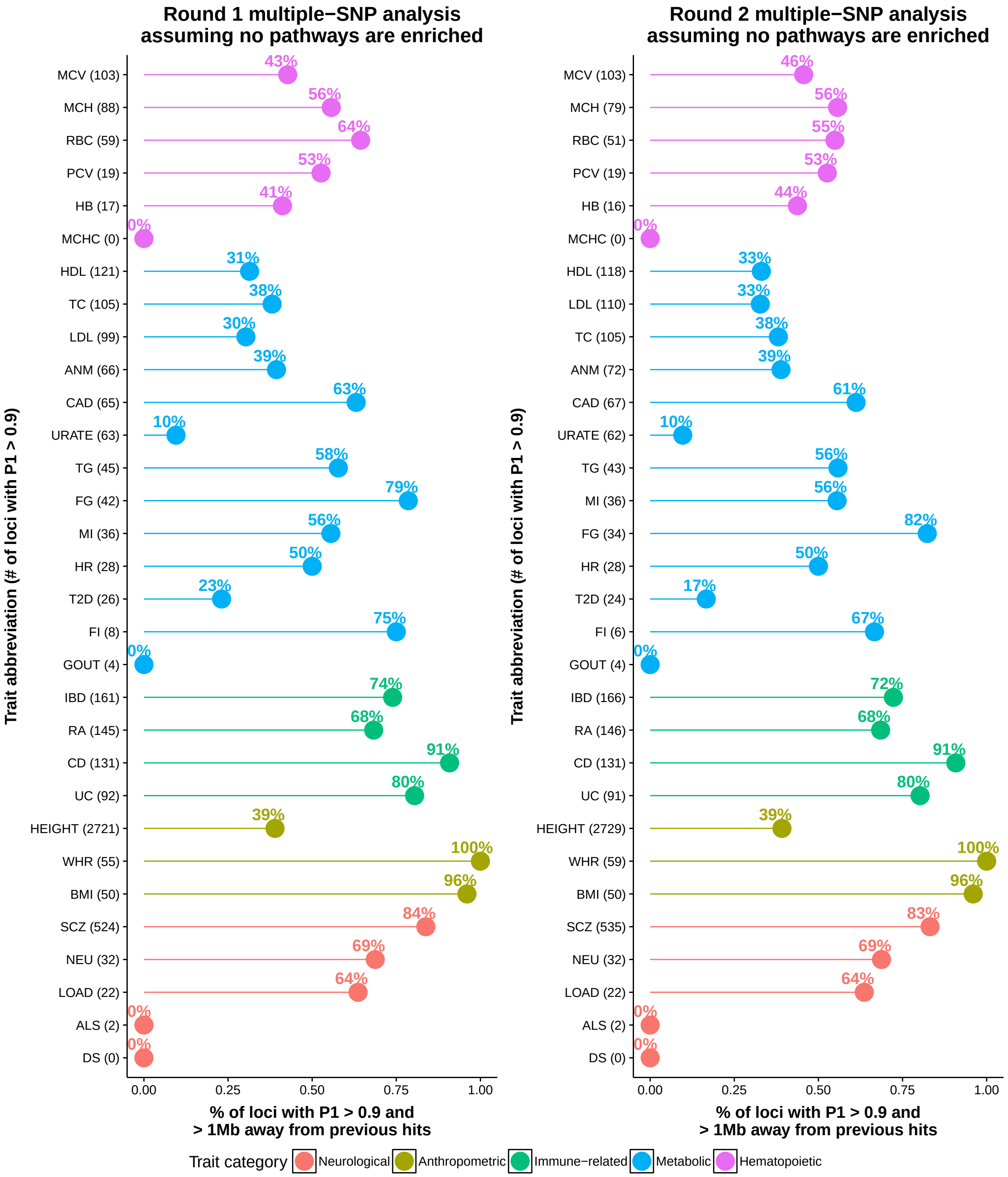

22.5. *All hits & ENS* > 1. Proportion of loci with ENS > 1 that are at least 1 Mb away from all previously-reported GWAS hits.

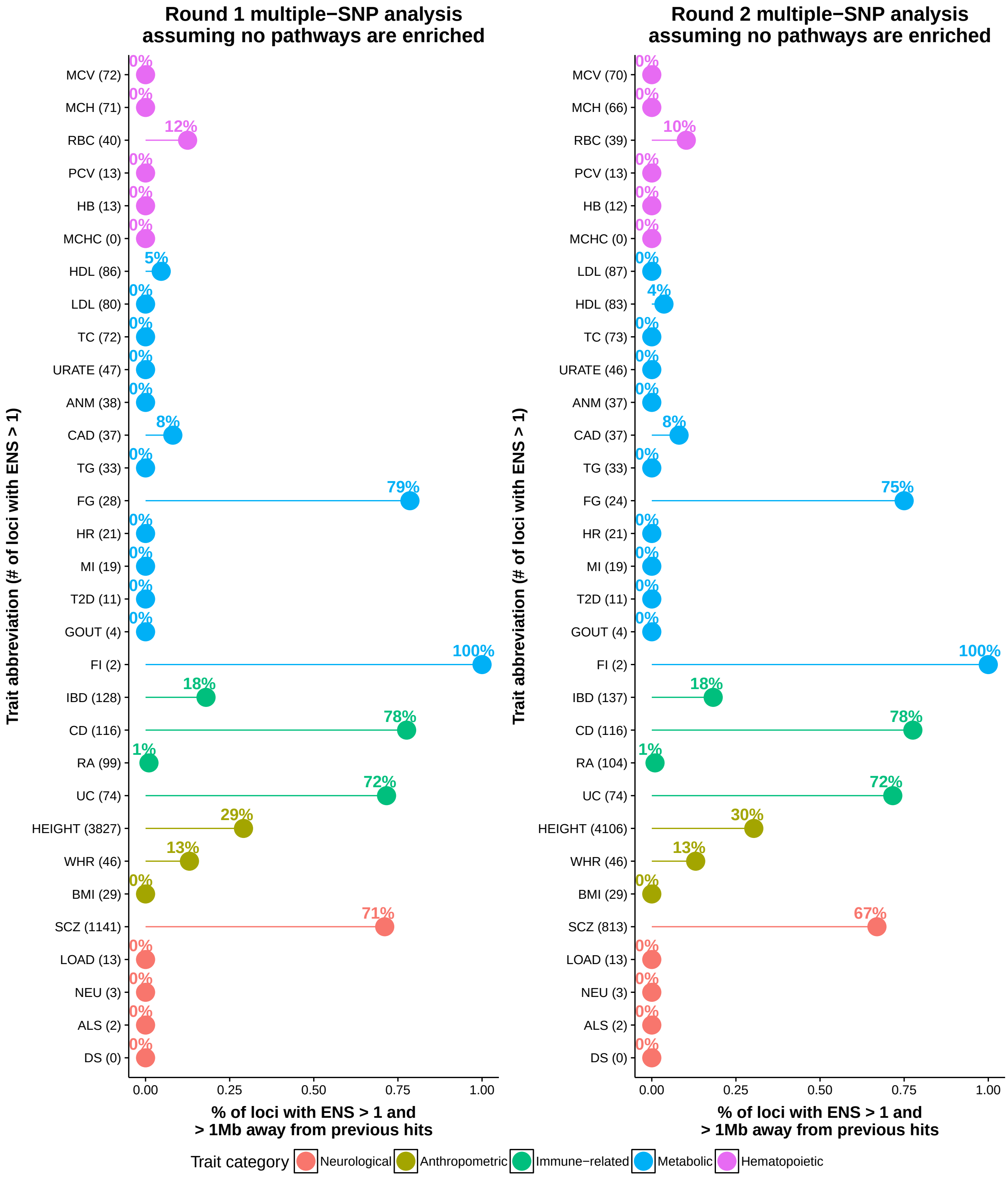

22.6. *Analyzed hits & ENS* > 1. Proportion of loci with ENS > 1 that are at least 1 Mb away from previously-reported GWAS hits that are included in genome-wide multiple-SNP analyses.

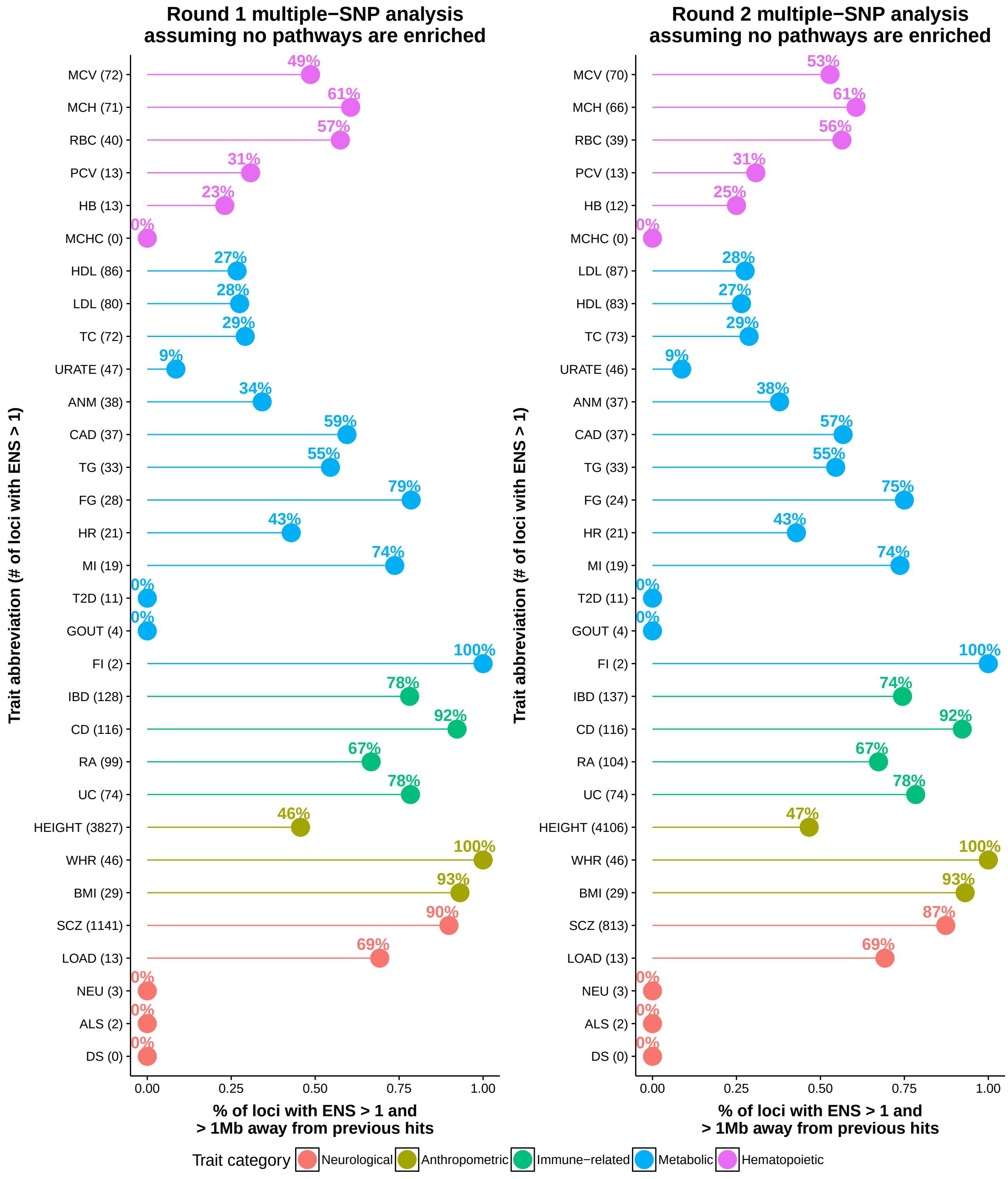

23. Supplementary Figure 15. Summary of biological pathways. Biological pathway definitions are retrieved from the Pathway Commons 2 ([66], version 7), NCBI Biosystems [67], PANTHER ([68], version 3.3) and BioCarta used in [2]. The Pathway Commons 2 database includes gene sets derived from Reactome [69], Nature Pathway Interaction Database (PID, [70]), HumanCyc [71], PANTHER, miRTarBase [72] and Kyoto Encyclopedia of Genes and Genomes (KEGG, [73]) pathways. The NCBI BioSystem database contains pathways from KEGG, BioCyc [74], PID, Reactome and WikiPathways [75]. See Supporting Information Text S1 of [2] for the rationale for choosing these pathway databases (`https://doi.org/10.1371/journal.pgen.1003770.s015`). For each panel, the bar chart on the right side (labelled as "Total") shows the total number of pathways retrieved from the corresponding databases, and the one on the left side (labelled as "Analyzed") shows the number of pathways used in the present enrichment analyses.

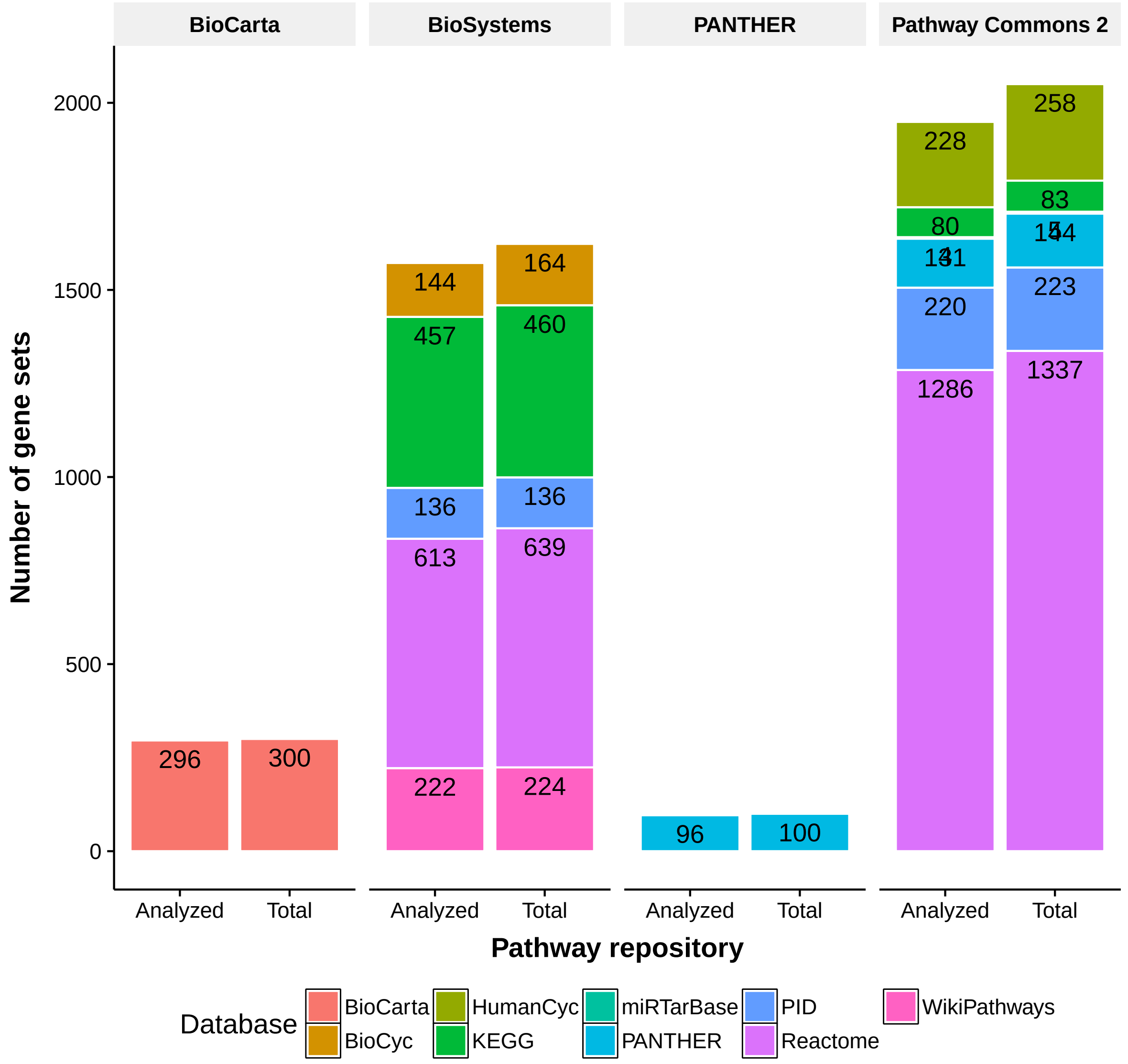

24. Supplementary Figure 16. Summary of genes. Genomic definitions for genes are derived from *Homo sapiens* reference genome GRCh37. In the present study, we consider 18,313 autosomal protein-coding genes that are mapped to the reference sequence.

(a) Distributions of gene set sizes for each pathway database-repository combination. Combinations are ordered by median numbers of genes in pathways, which are displayed in each box plot. The vertical axis uses a logarithmic scale (base: 10). PC: Pathway Commons. BS: NCBI BioSystems.

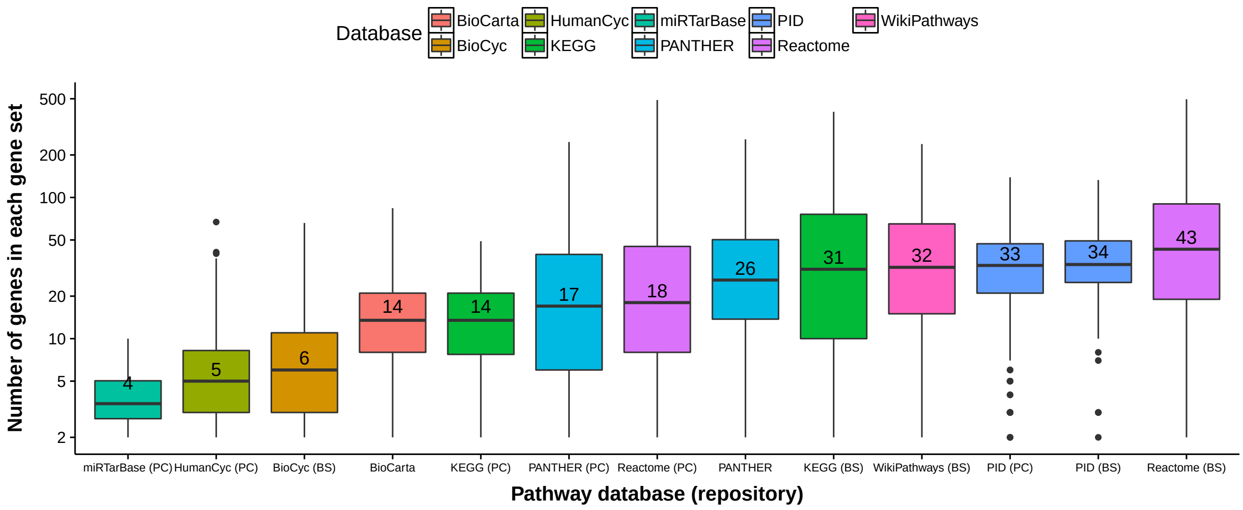

(b) Manhattan plot of the number of pathway annotations for each gene. The highlighted genes (colored in green and labelled by their HGNC symbols) belong to more than 270 of 3,913 analyzed biological pathways. The Manhattan plot is produced by the package `qqman` [76].

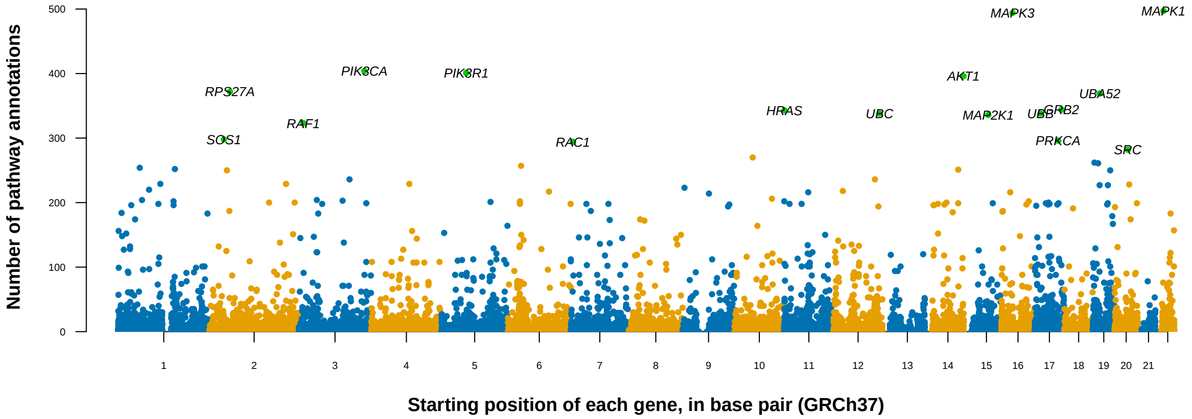

25. Supplementary Figure 17. Sanity checks of top-ranked gene set enrichments for 31 phenotypes. To quickly evaluate whether the strong enrichments identified in our model-based analyses can possibly be true, we develop two sanity checks.

The first sanity check is an "eyeball test" that visualizes the distribution of GWAS single-SNP z-scores for a target trait, stratified by SNP-level annotations of a target gene set. Specifically, we plot two estimated density curves for each pair of trait and gene set:

- a **solid red curve** estimated from z-scores of SNPs within 100 kb of the transcribed region of a gene in the gene set ("inside gene set" SNPs);
- a **dashed black curve** estimated from z-scores of remaining SNPs ("outside gene set" SNPs).

For a typical pair of trait and gene set that is deemed to pass the "eyeball test", its dashed black curve is often more "spiky" at zero, and its solid red curve is more spread out. The density curves are produced by the function `geom_density` (default setting) in the package `ggplot2` [77].

The second sanity check computes a likelihood ratio (LR) for the following two models:

- **baseline model** (a3): "inside gene set" SNPs have the same effect size distribution as "outside gene set" SNPs, which can be estimated by `a1$fitted_g` based on the whole genome data;
- **enrichment model** (a2): "inside gene set" and "outside gene set" SNPs have different effect size distributions, which should be estimated separately.

For a strongly enriched gene set, its LR value tends to be very large, since the data should favor the enrichment (a2) over the baseline model (a3). The second check based on LR computation complements the first visual check in cases where the "eyeball test" results are not clearly visible. The LR calculation is based on the package `ashr` [78]. Below are `R` codes that illustrate the LR calculation.

```
suppressPackageStartupMessages(library(ashr))

# load GWAS summary statistics and SNP-level annotations
betahat <- c(sumstat$betahat)
se <- c(sumstat$se)
snps <- c(sumstat$snps)

# analyze summary data of the whole genome
a1 <- ashr::ash(betahat=betahat, sebetahat=se,
                mixcompdist="halfuniform", method="shrink")

# analyze summary data of SNPs that are "inside gene set"
# where the prior is estimated from data
a2 <- ashr::ash(betahat=betahat[snps], sebetahat=se[snps],
                mixcompdist="halfuniform", method="shrink")

# analyze summary data of SNPs that are "inside gene set"
# where the prior is fixed as the one estimated in a1
a3 <- ashr::ash(betahat=betahat[snps], sebetahat=se[snps],
                mixcompdist="halfuniform", method="shrink", fixg=T, g=a1$fitted_g)

# compute log10 likelihood ratio statistics
log10LR <- (a2$logLR - a3$logLR) / log(10)
```

We first check the trait-pathway pairs reported in **Table 1**, and then check the trait-tissue pairs reported in **Table 3**. We also check the top 10 most enriched pathways with at least 10 member genes for each of the 31 traits in our Round 2 analyses (see **Supplementary Table 7** for details). Full information about these top enriched pathways and tissue-based gene sets is available at http://xiangzhu.github.io/rss-gsea/.

Pathway-trait pairs reported in Table 1

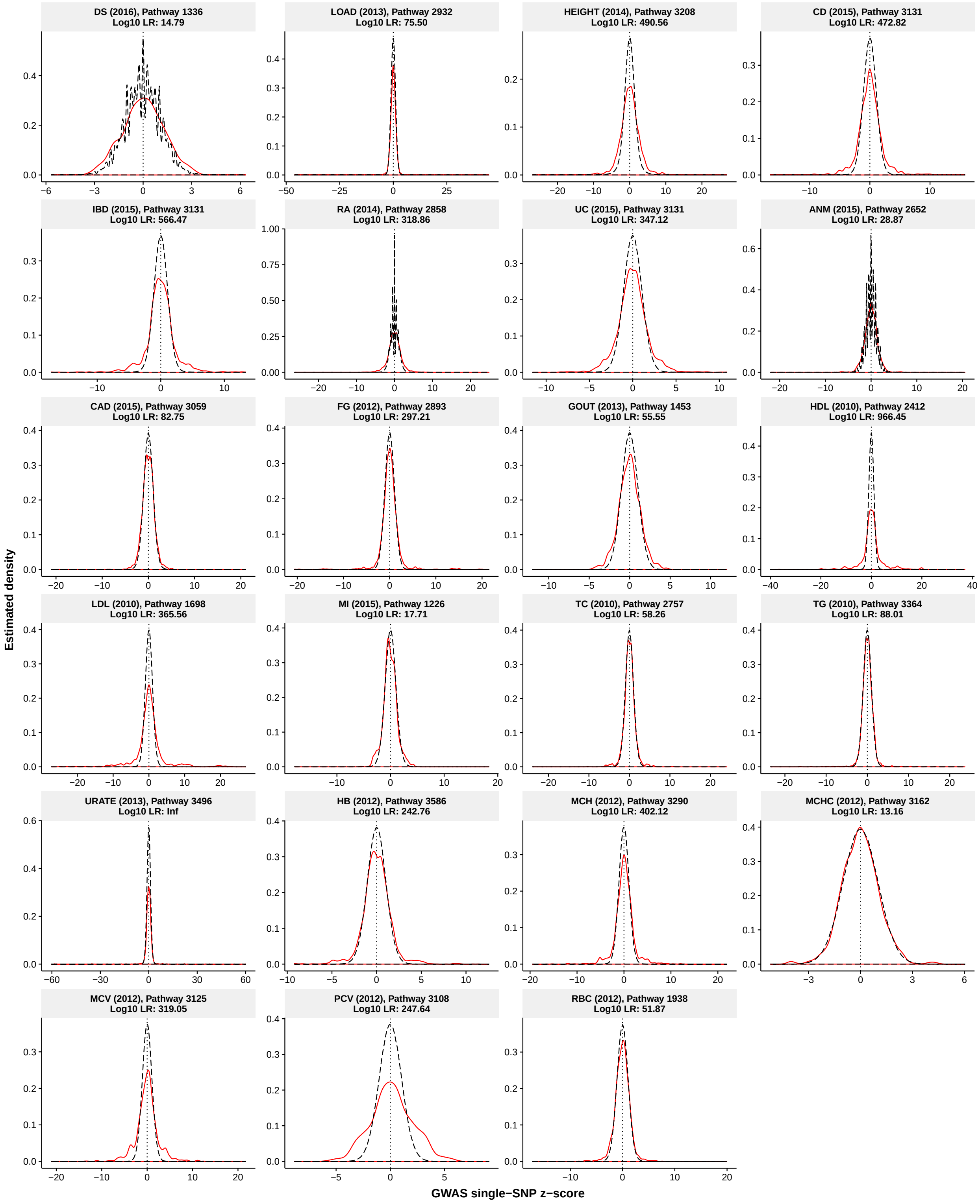

Tissue-trait pairs reported in Table 3

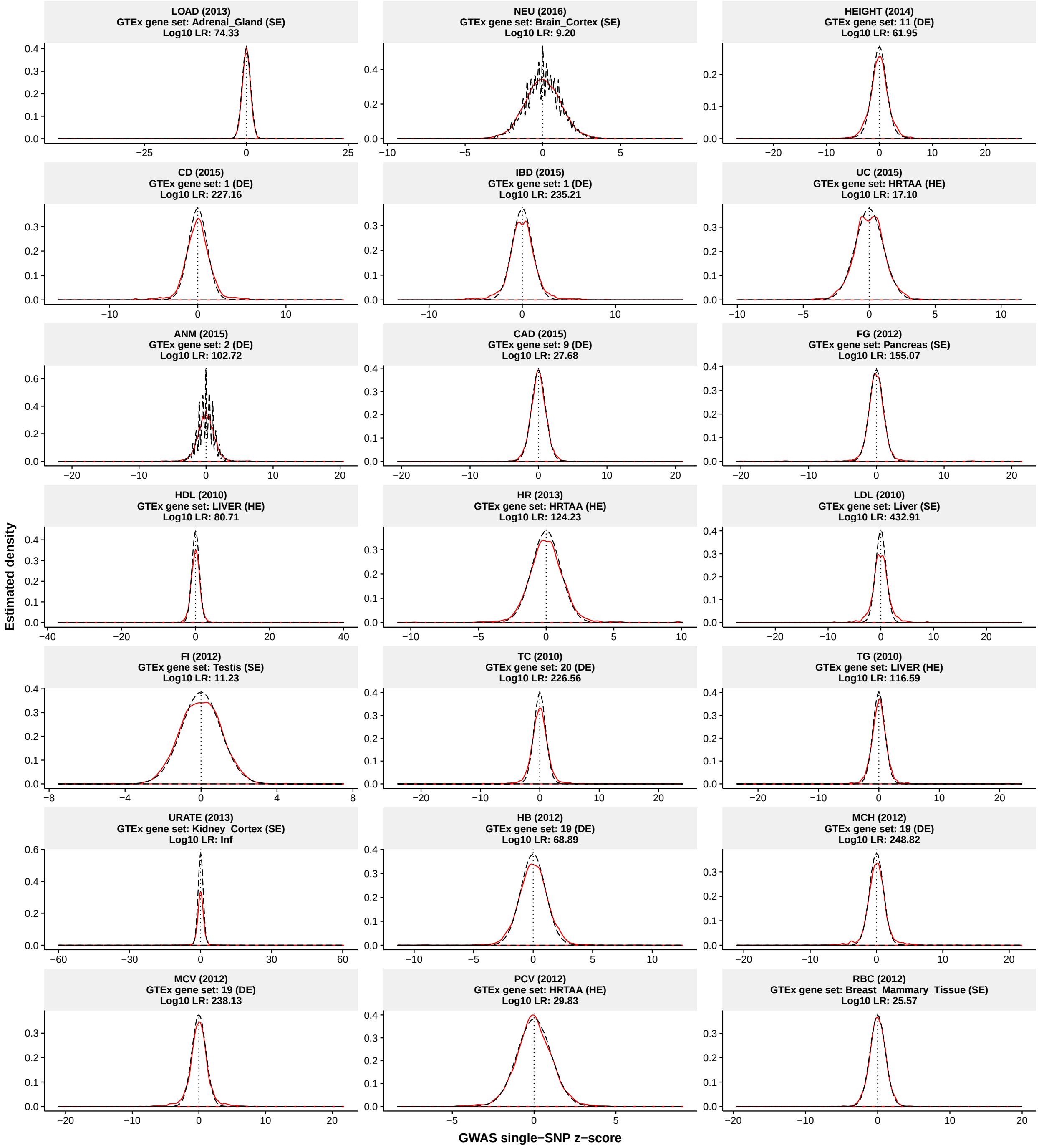

Amyotrophic lateral sclerosis [45]

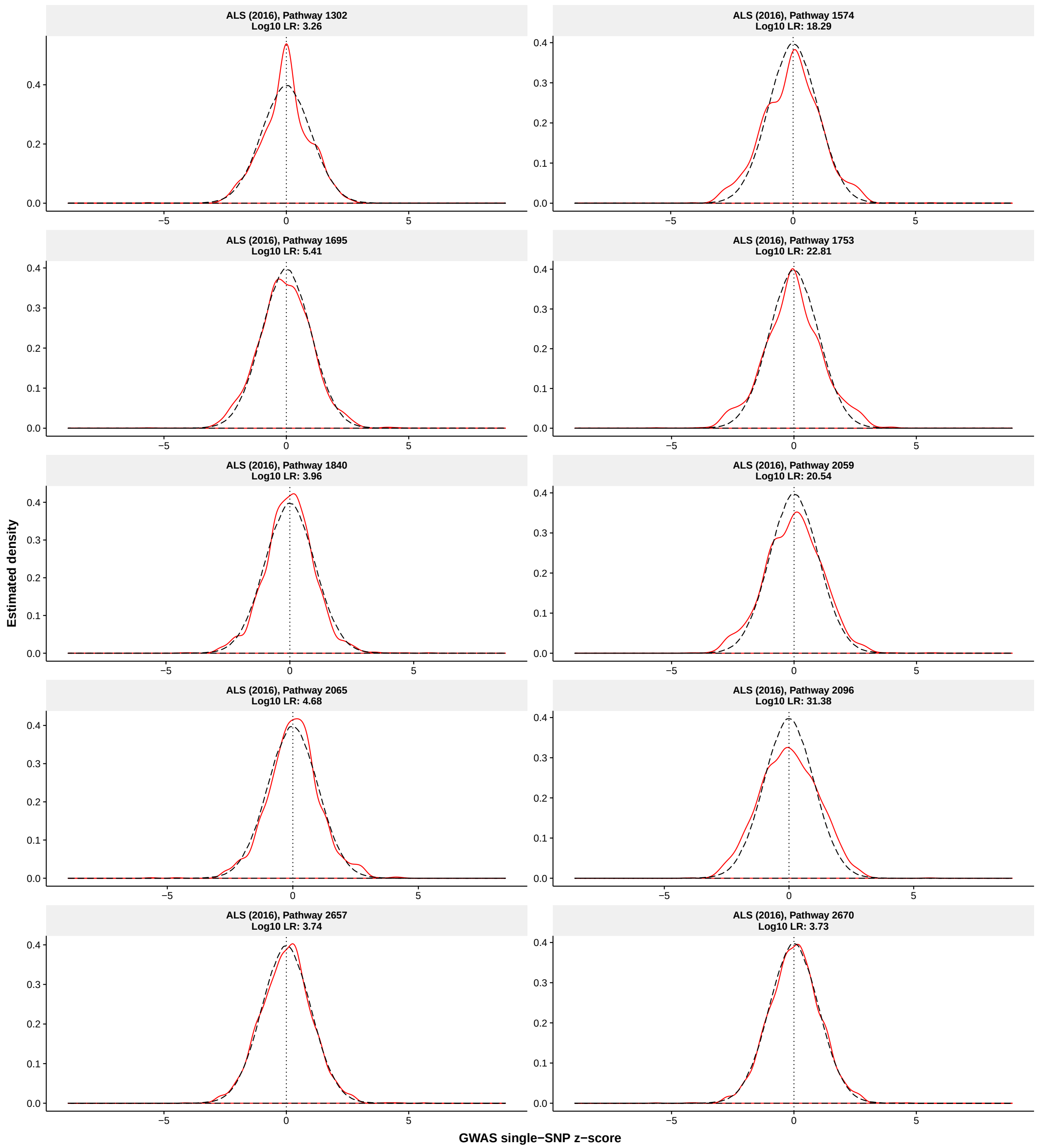

Age at natural menopause [42]

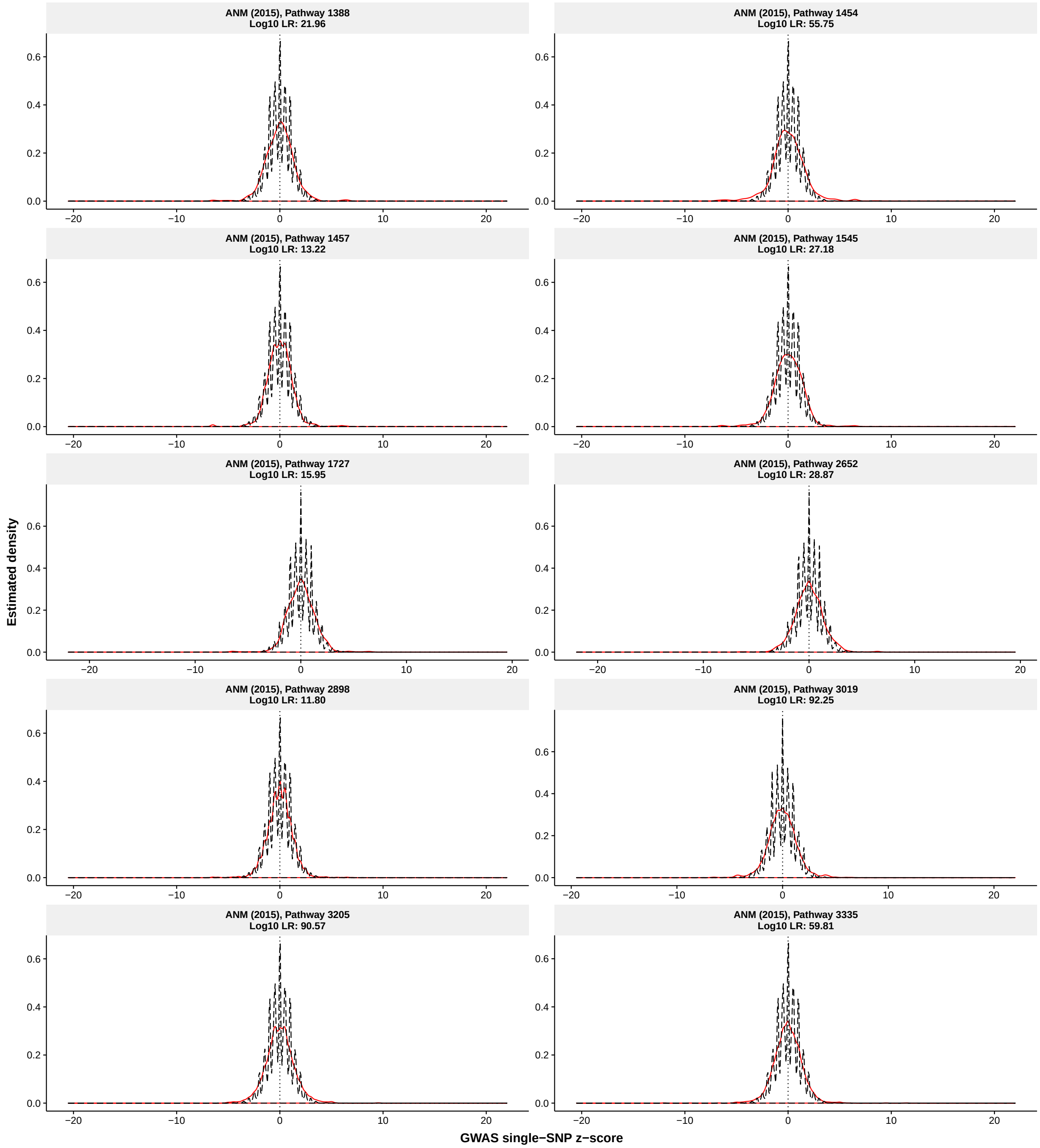

Body mass index [35]

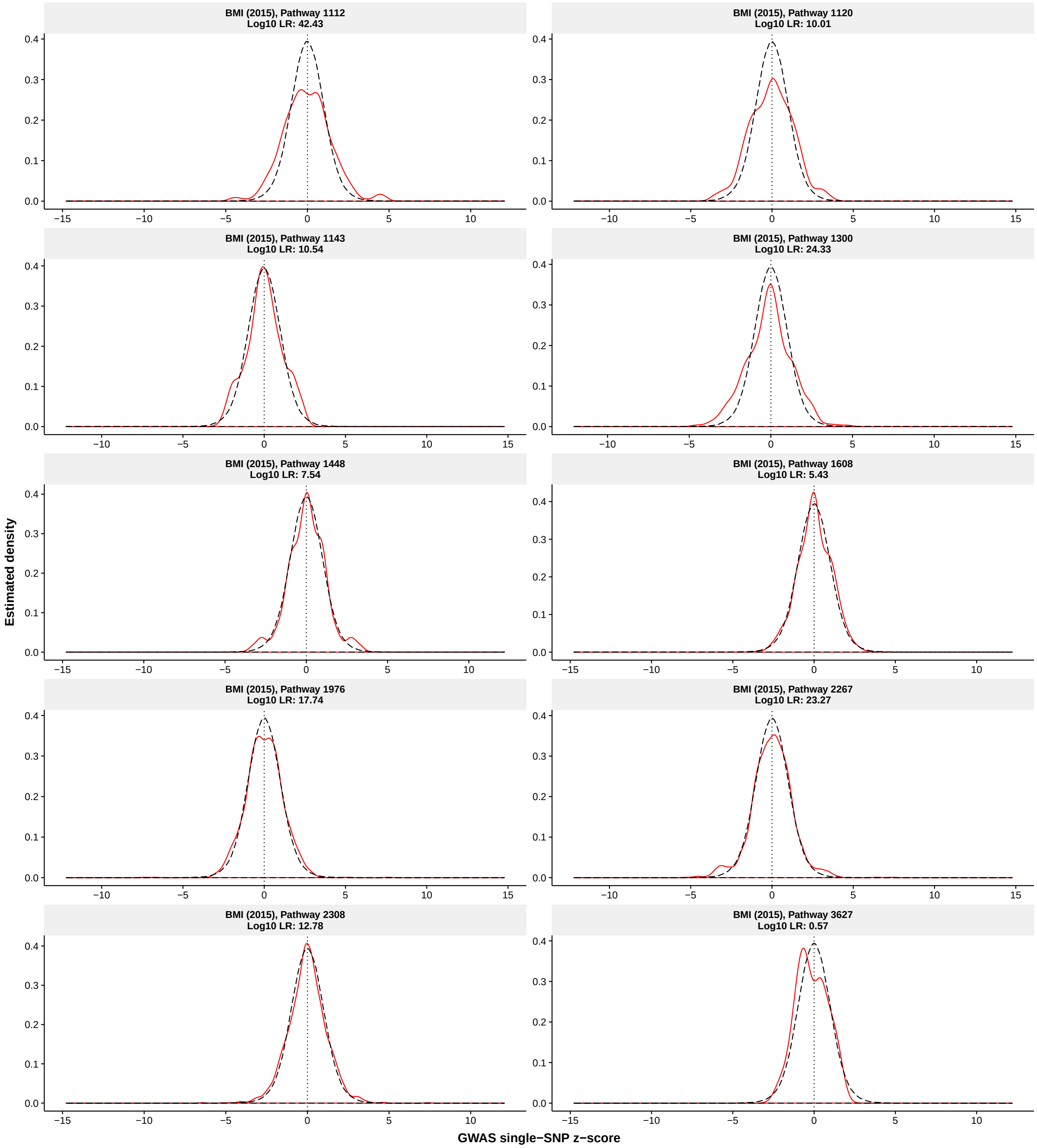

Coronary artery disease [18]

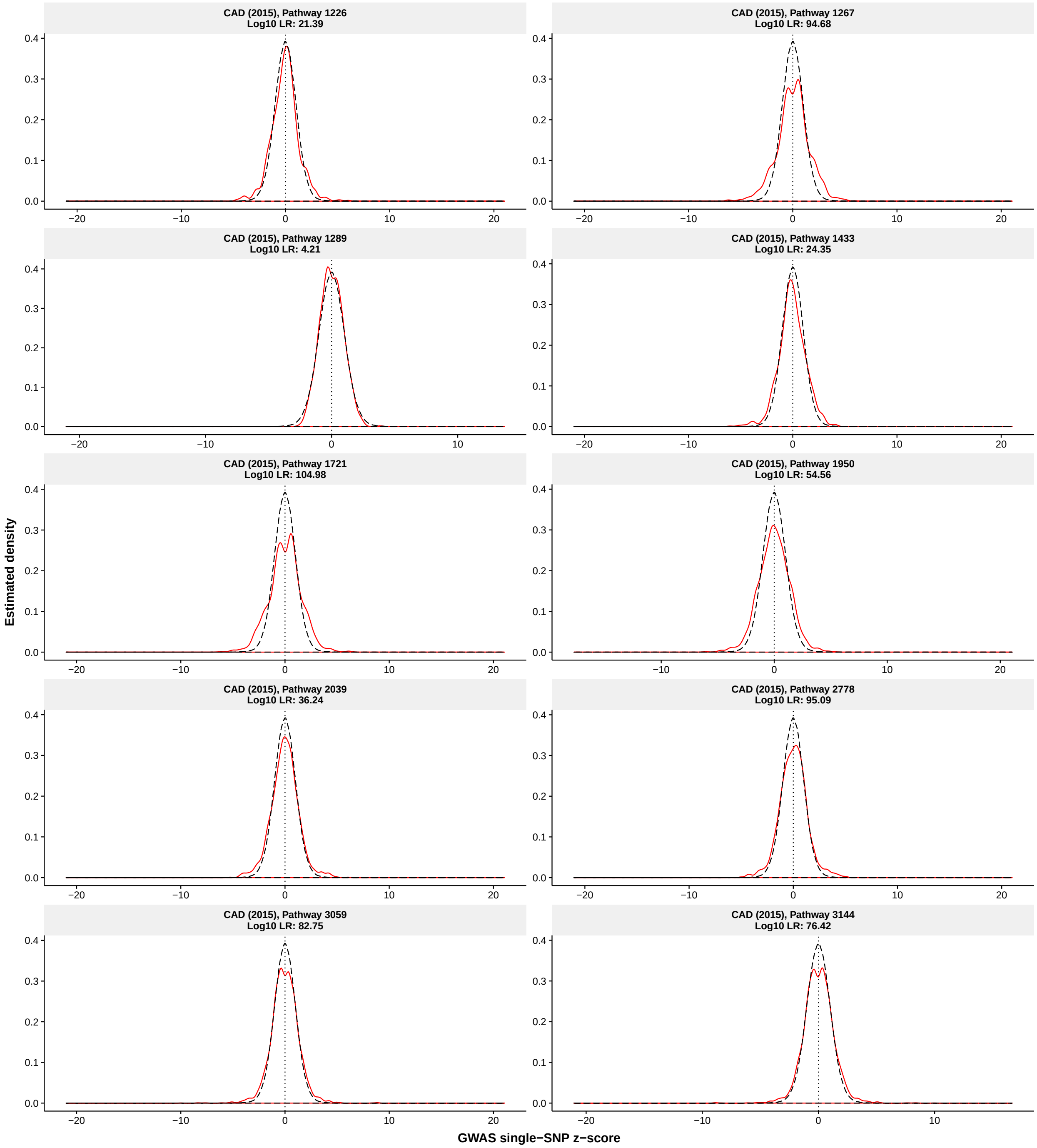

Crohn's disease [11]

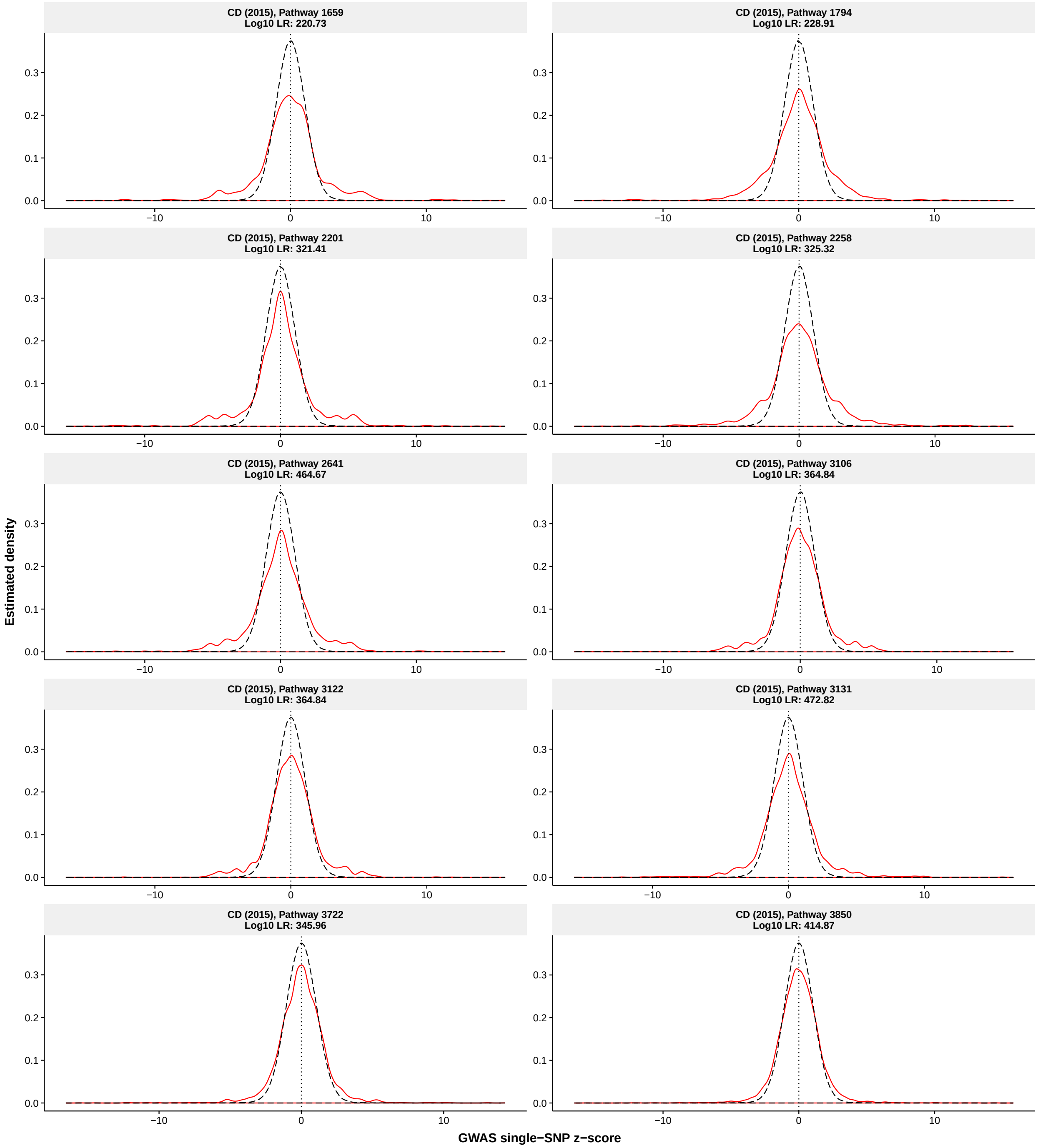

Depressive symptoms [40]

Note that there are only five pathways with at least 10 member genes in Round 2 enrichment analysis of depressive symptoms; see `https://xiangzhu.github.io/rss-gsea/ds_2016.html`.

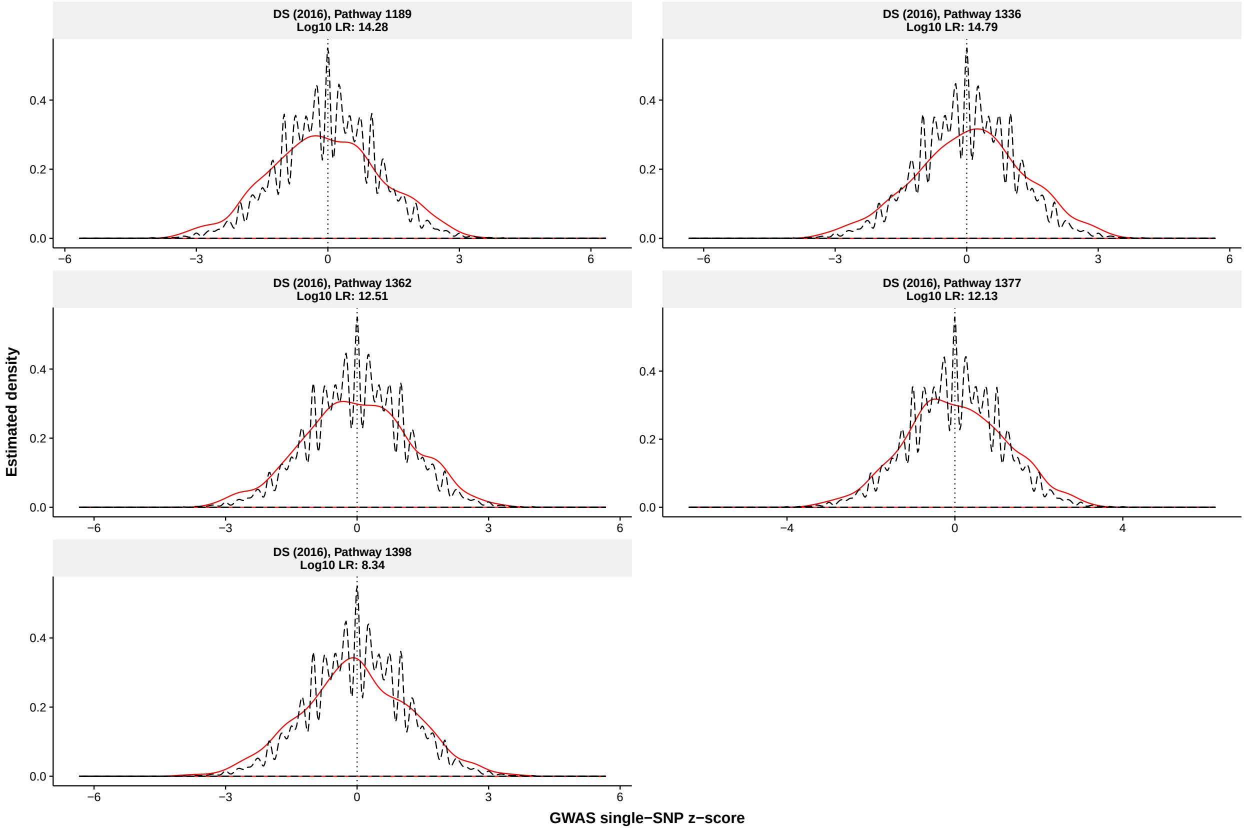

Fasting glucose levels [44]

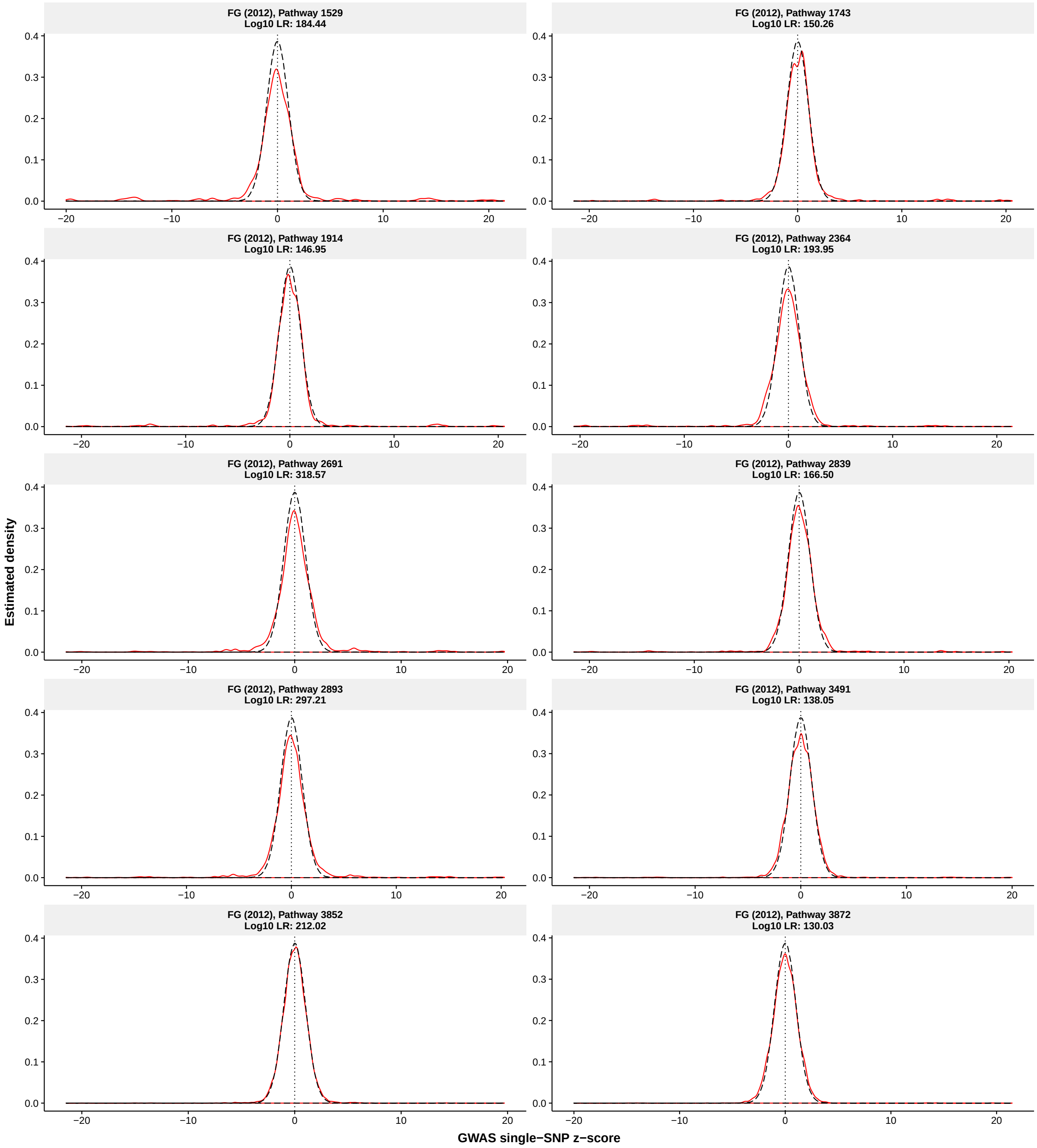

Fasting insulin levels [44]

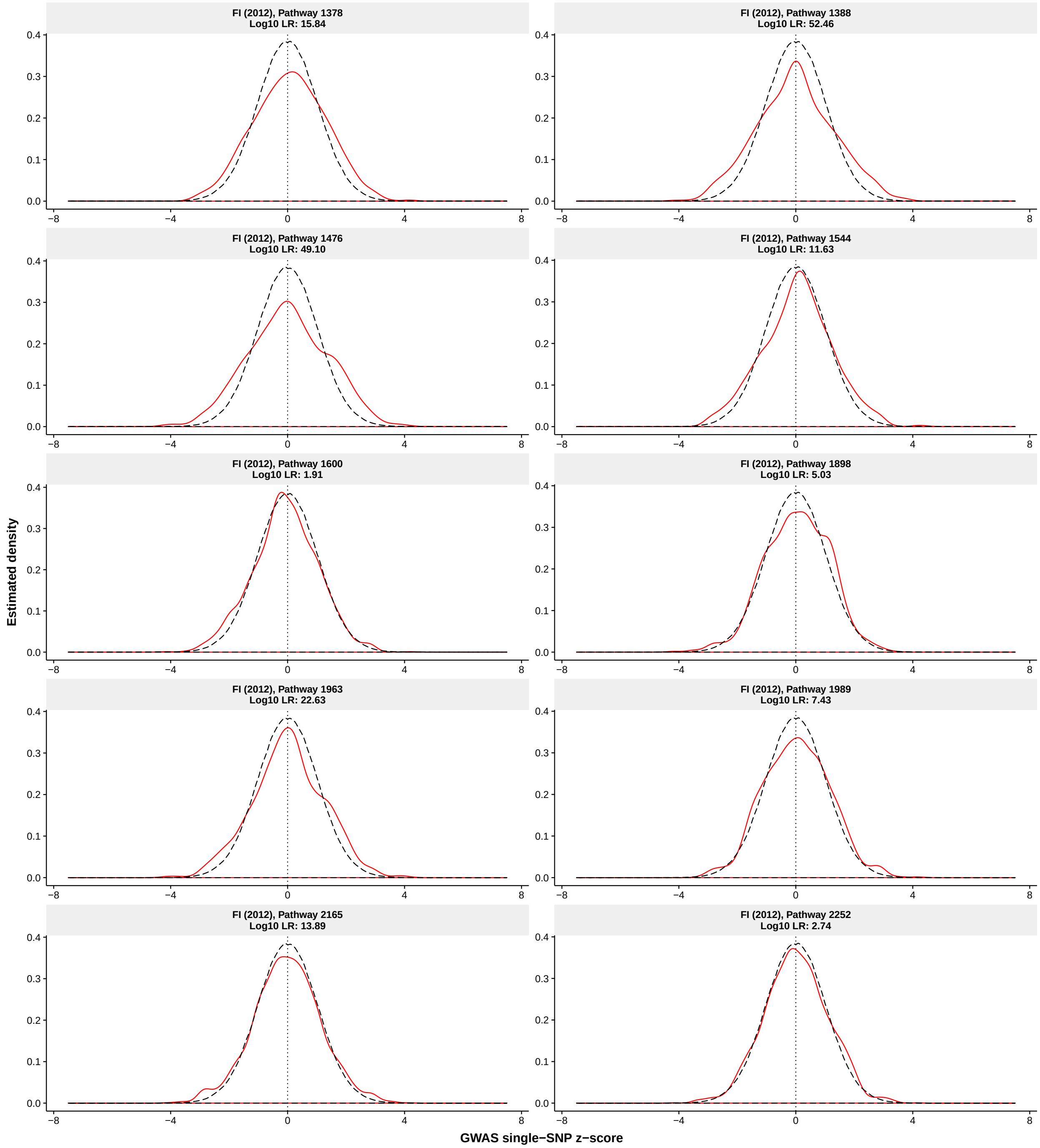

Gout [43]

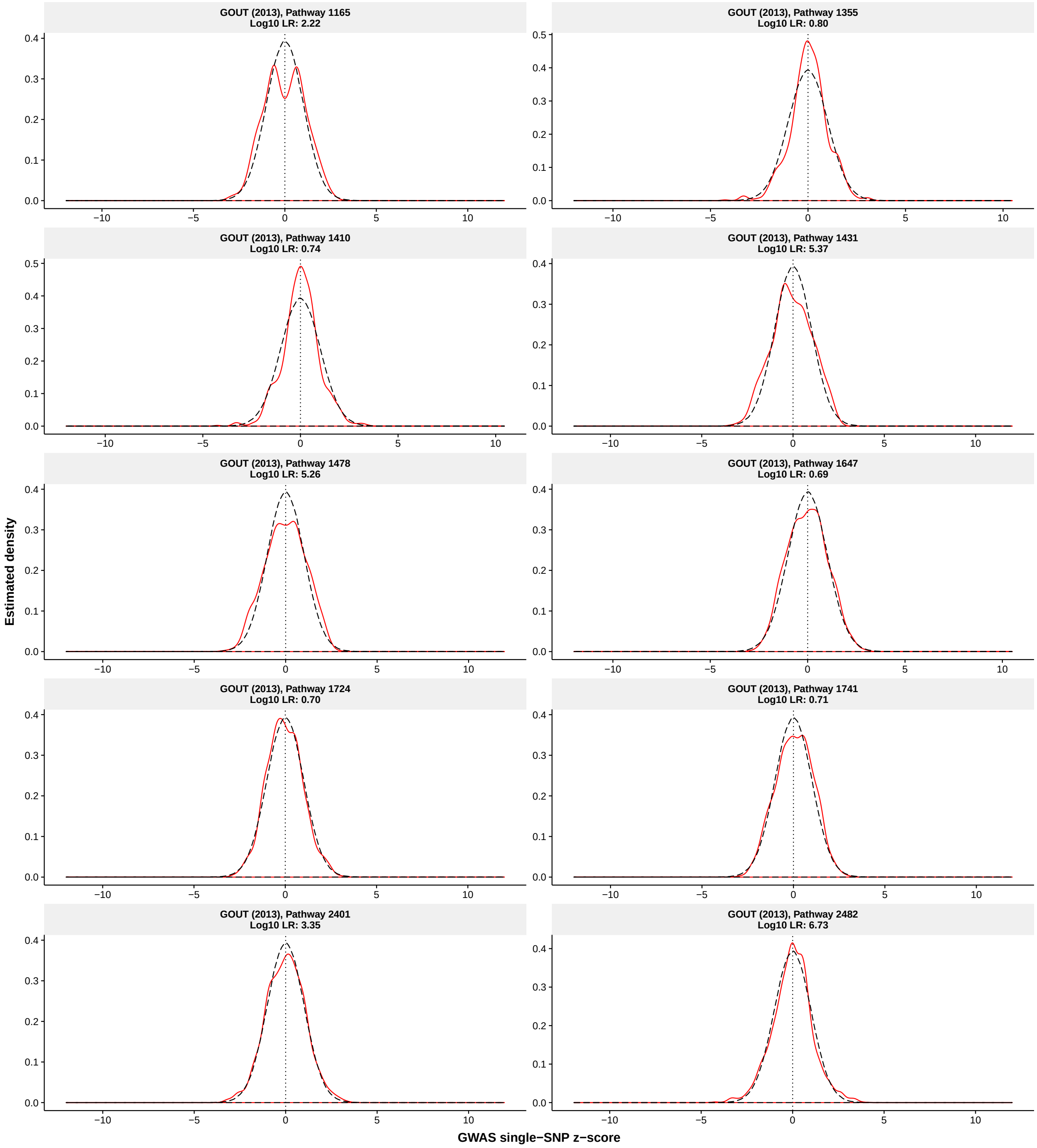
GOUT (2013), Pathway 1165
Log10 LR: 2.22
GOUT (2013), Pathway 1355
Log10 LR: 0.80
GOUT (2013), Pathway 1410
Log10 LR: 0.74
GOUT (2013), Pathway 1431
Log10 LR: 5.37
GOUT (2013), Pathway 1478
Log10 LR: 5.26
GOUT (2013), Pathway 1647
Log10 LR: 0.69
GOUT (2013), Pathway 1724
Log10 LR: 0.70
GOUT (2013), Pathway 1741
Log10 LR: 0.71
GOUT (2013), Pathway 2401
Log10 LR: 3.35
GOUT (2013), Pathway 2482
Log10 LR: 6.73
Estimated density
GWAS single−SNP z−score

Haemoglobin [46]

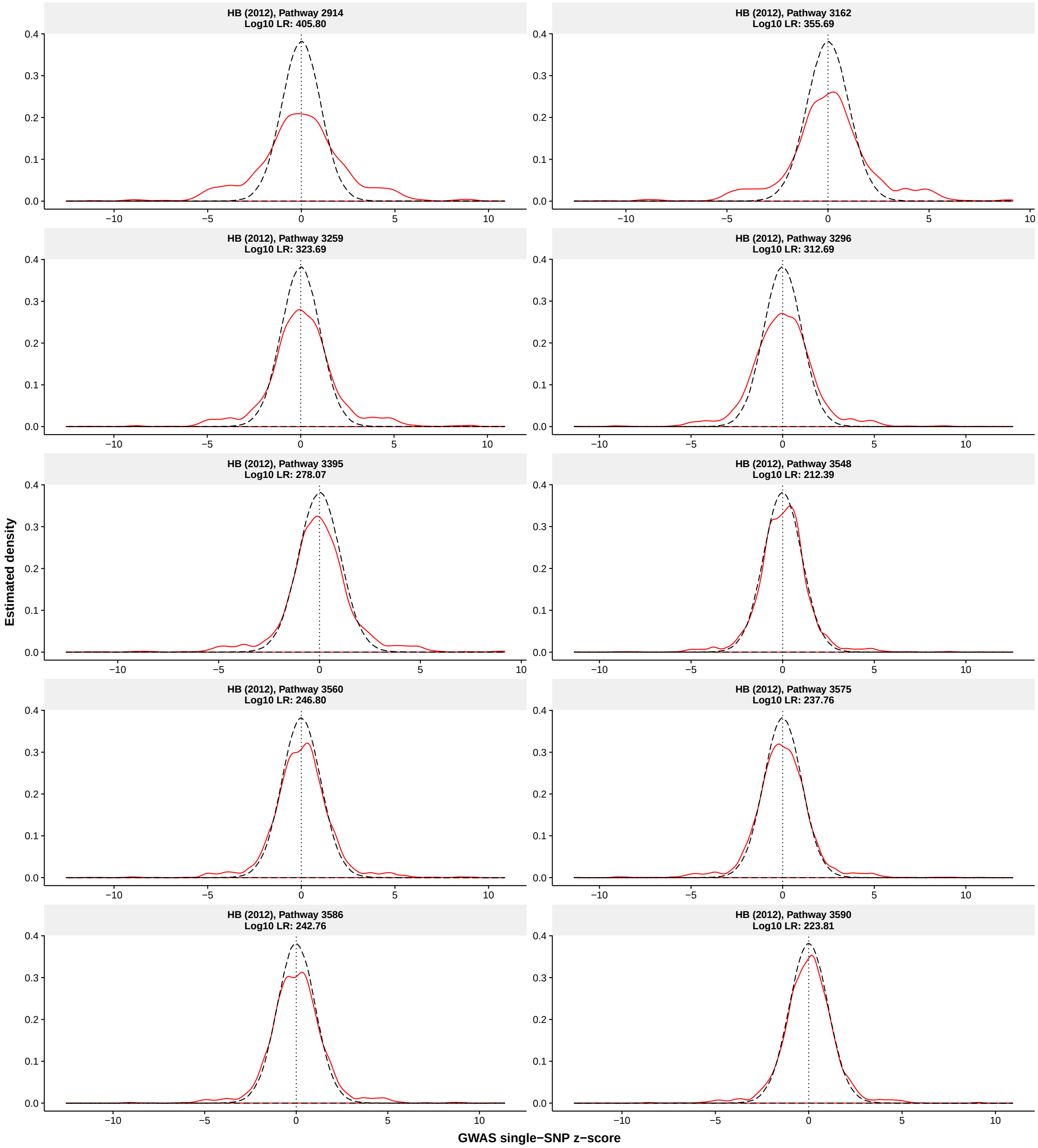

High-density lipoprotein [22]

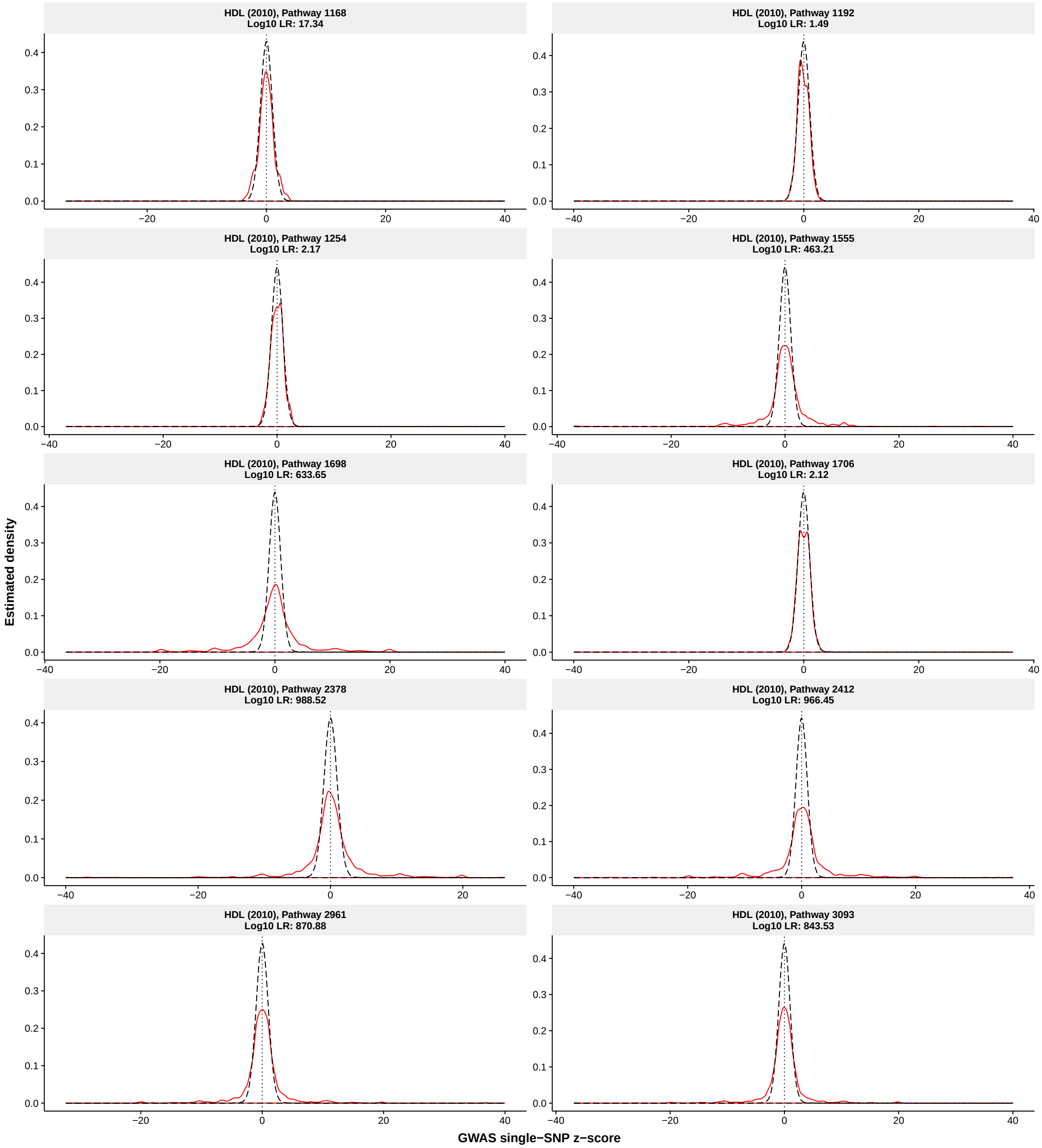

Adult height [5]

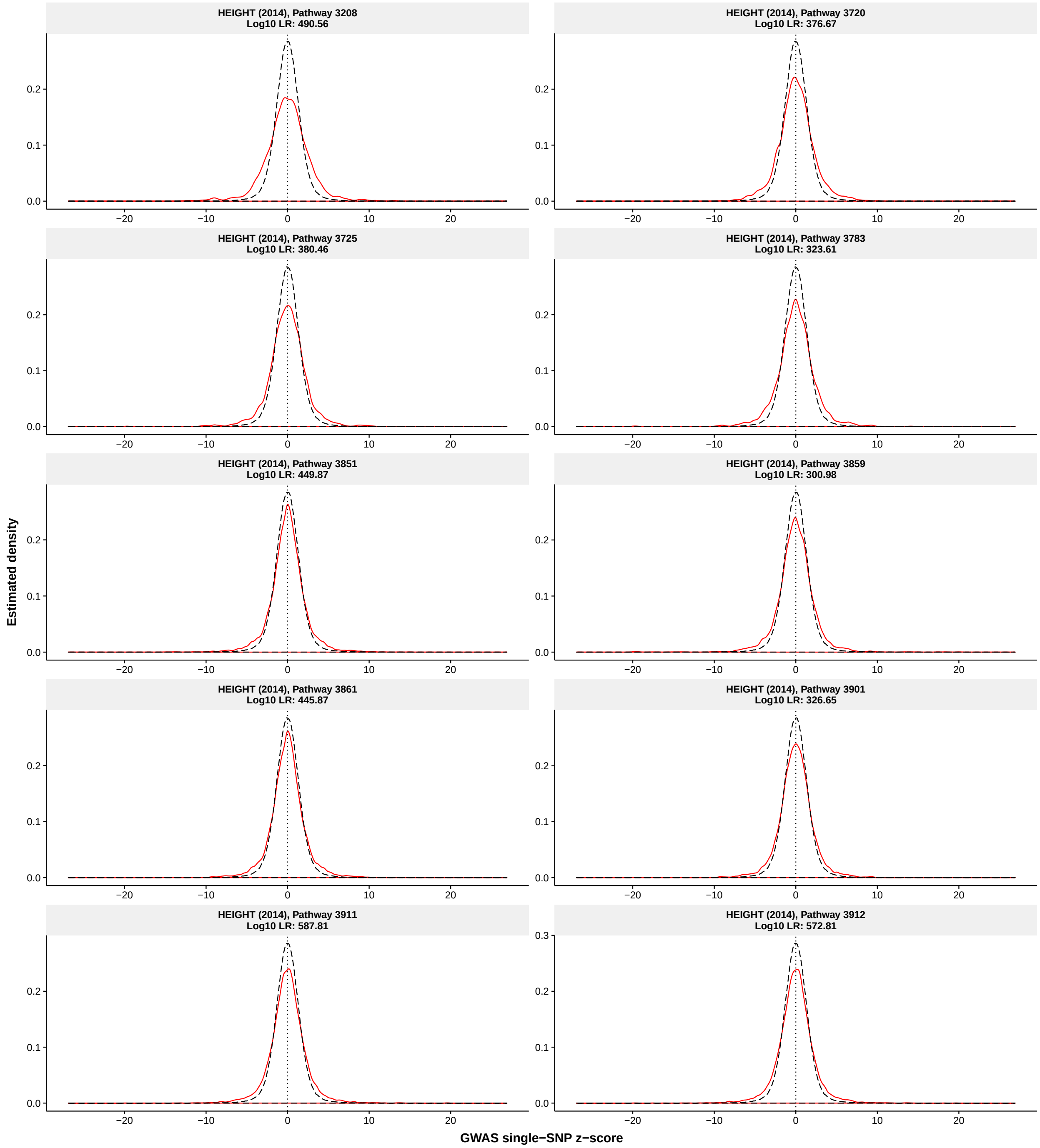

Heart rate [38]

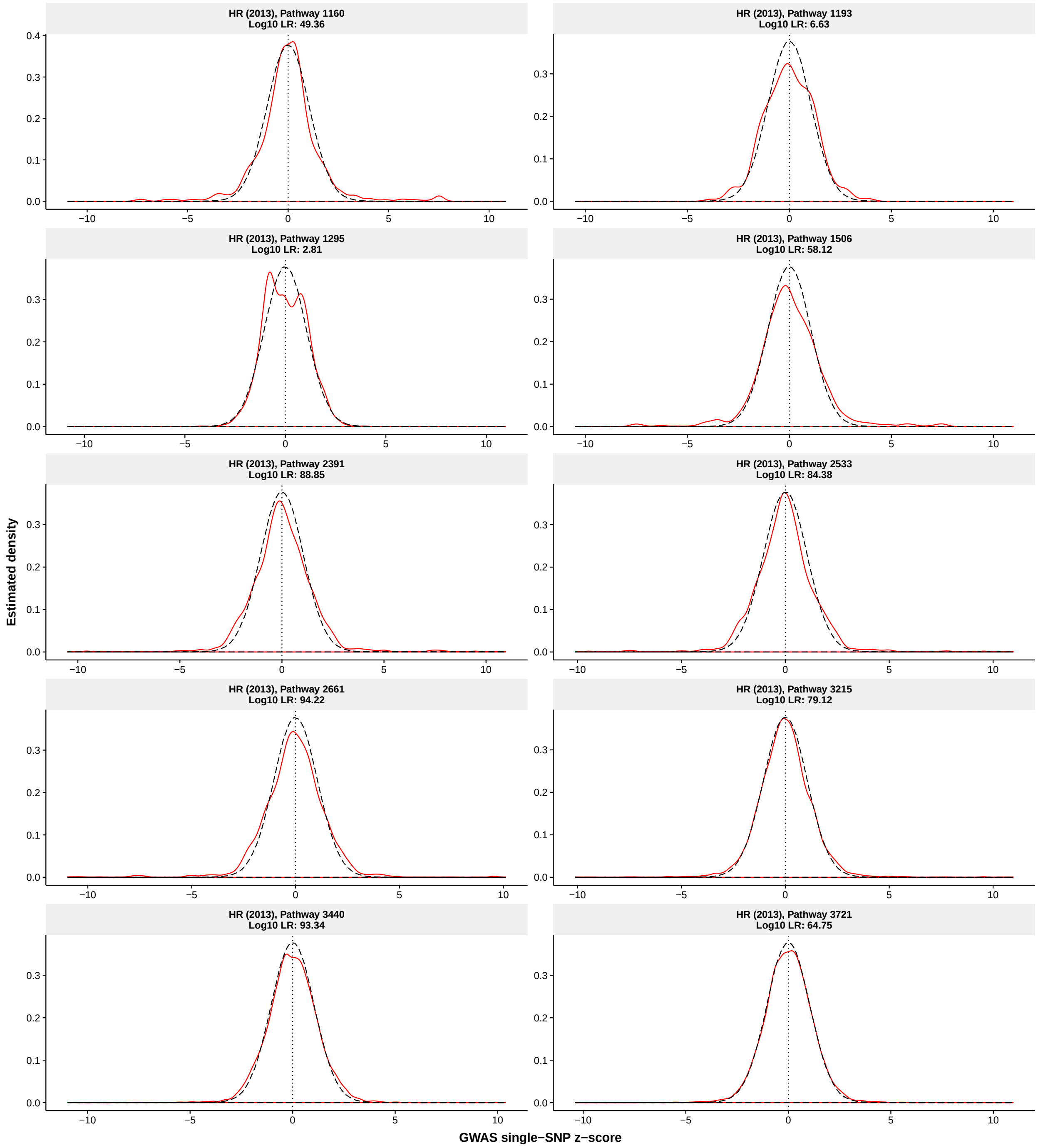

Inflammatory bowel disease [11]

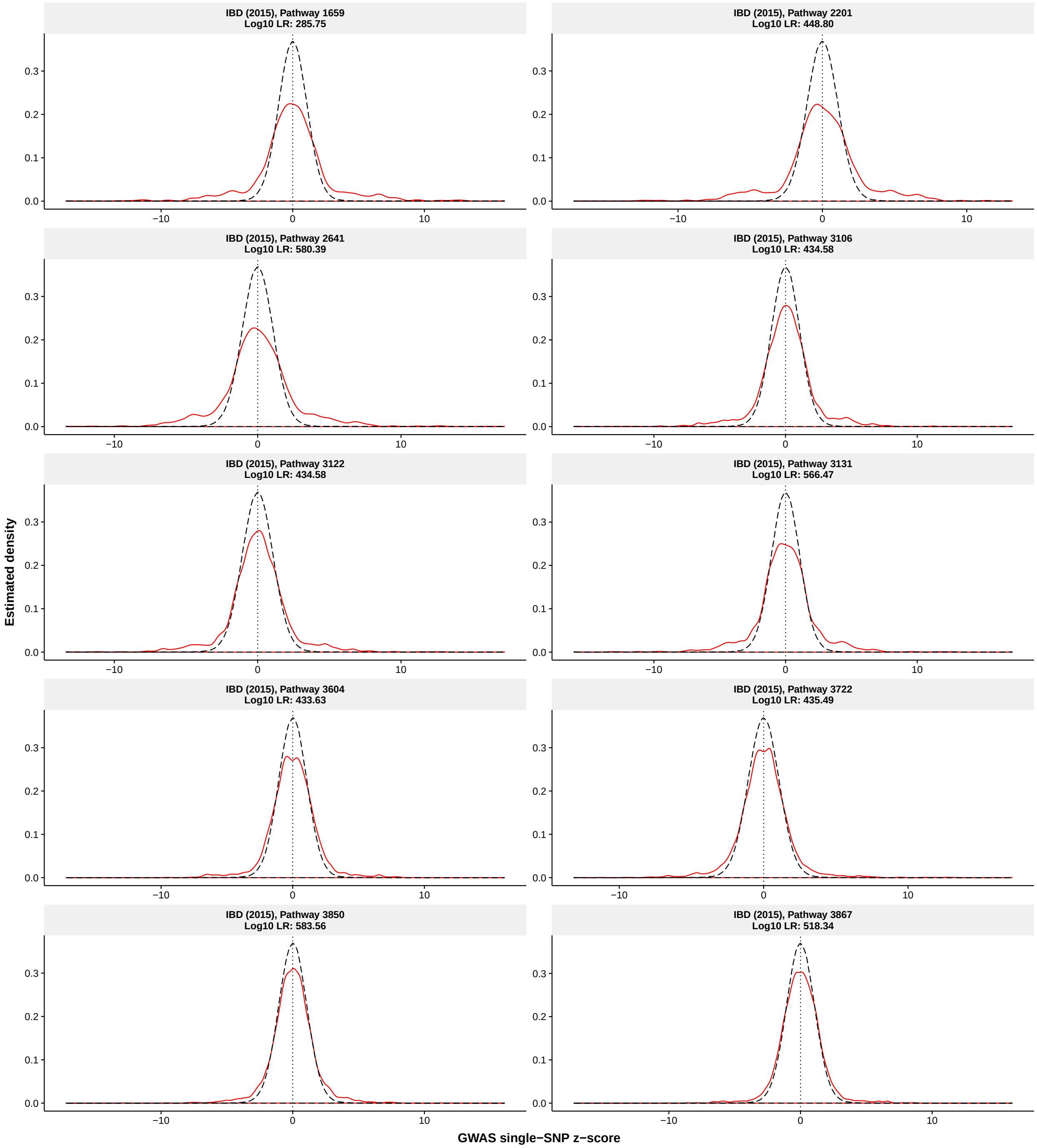

Low-density lipoprotein [22]

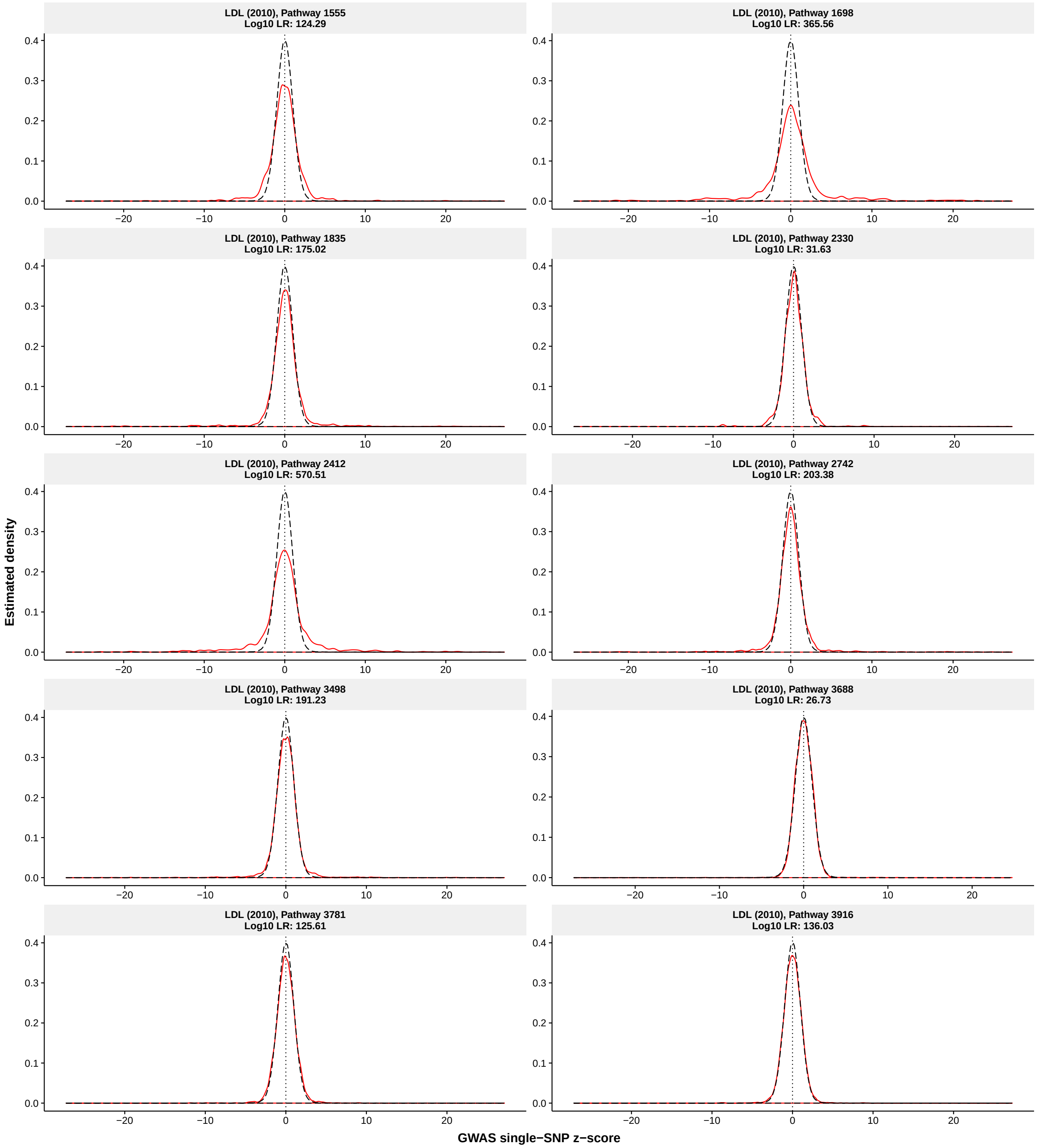

Alzheimer's disease [39]

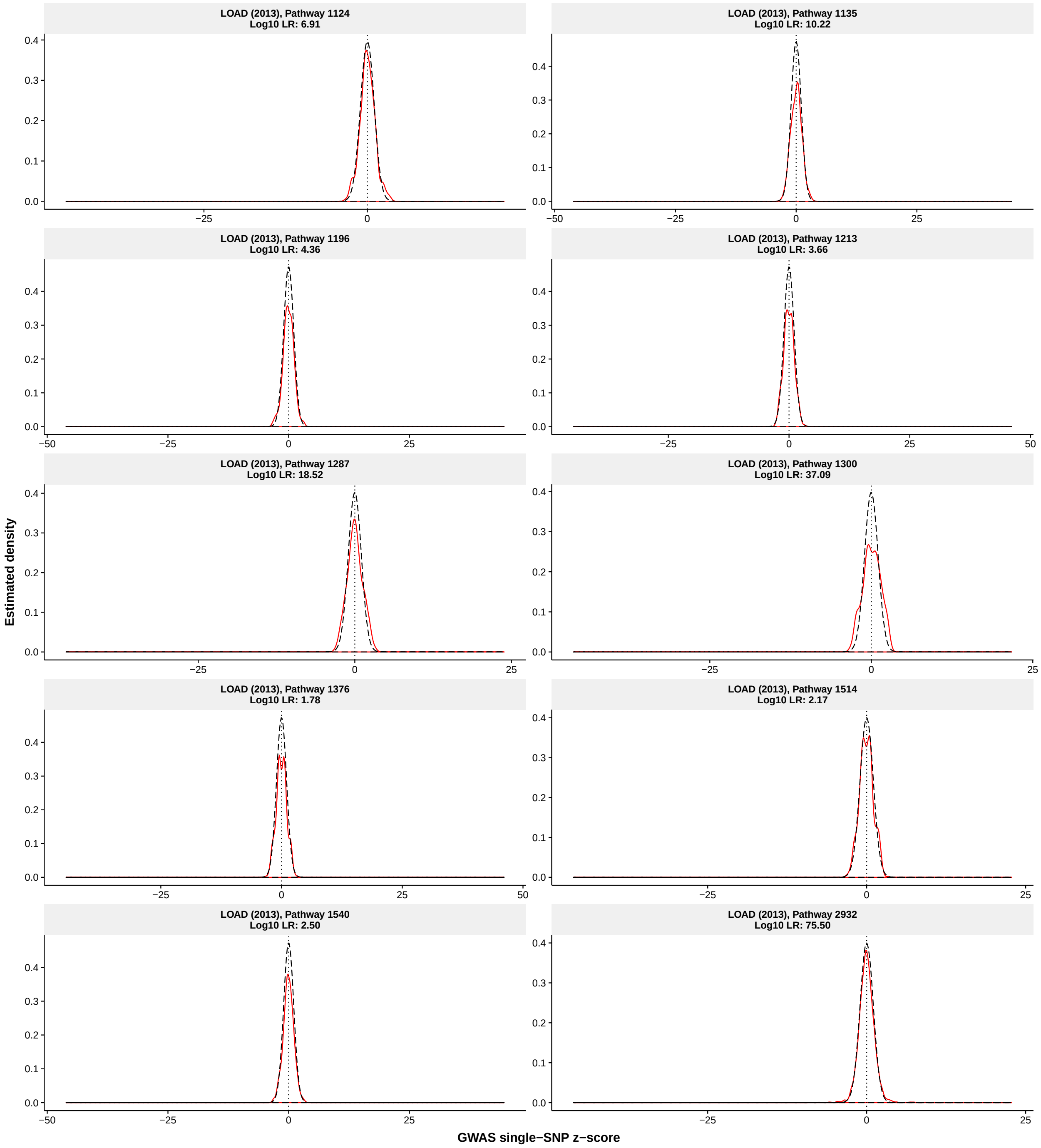

Mean cell haemoglobin [46]

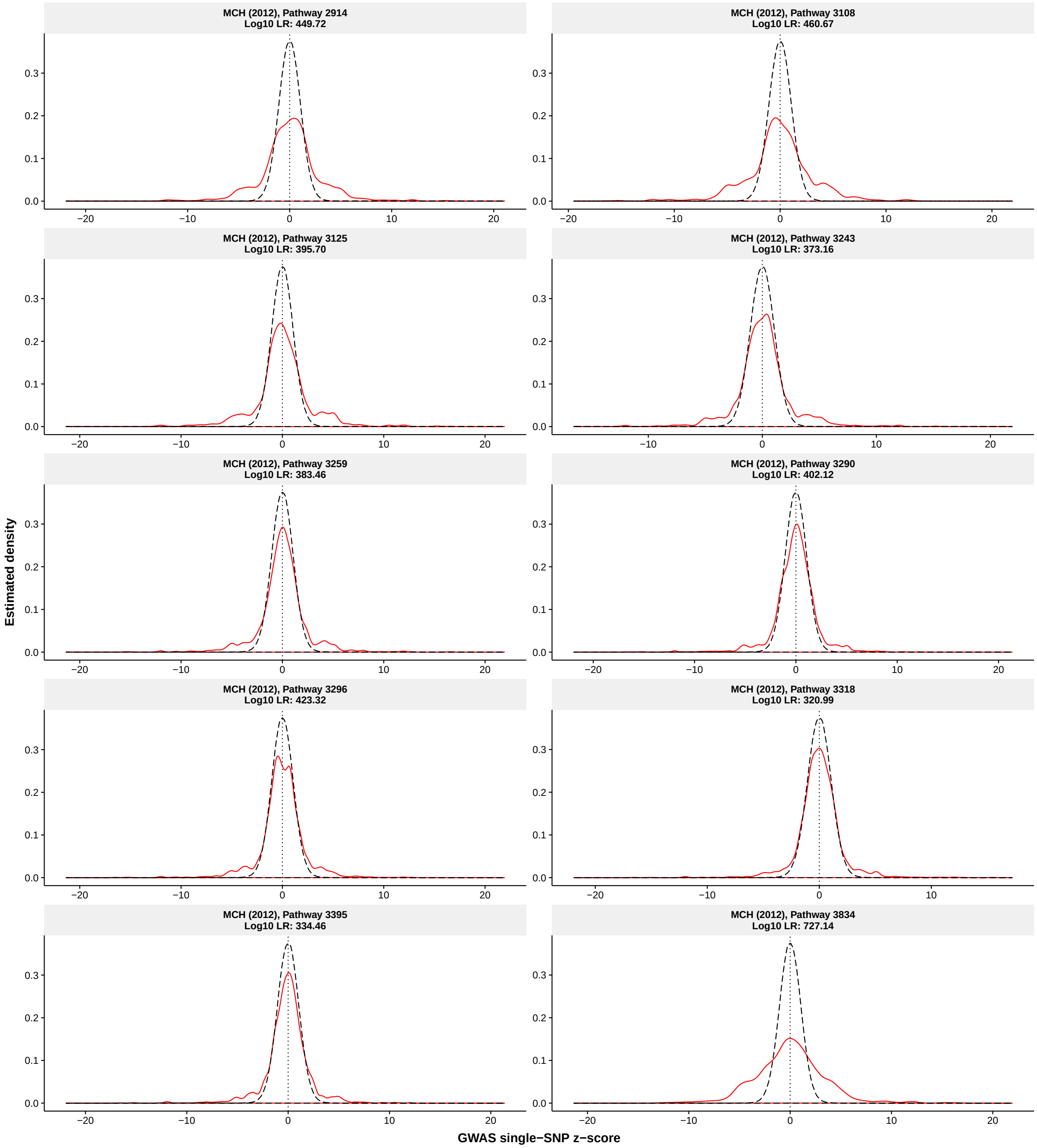

Mean cell haemoglobin concentration [46]

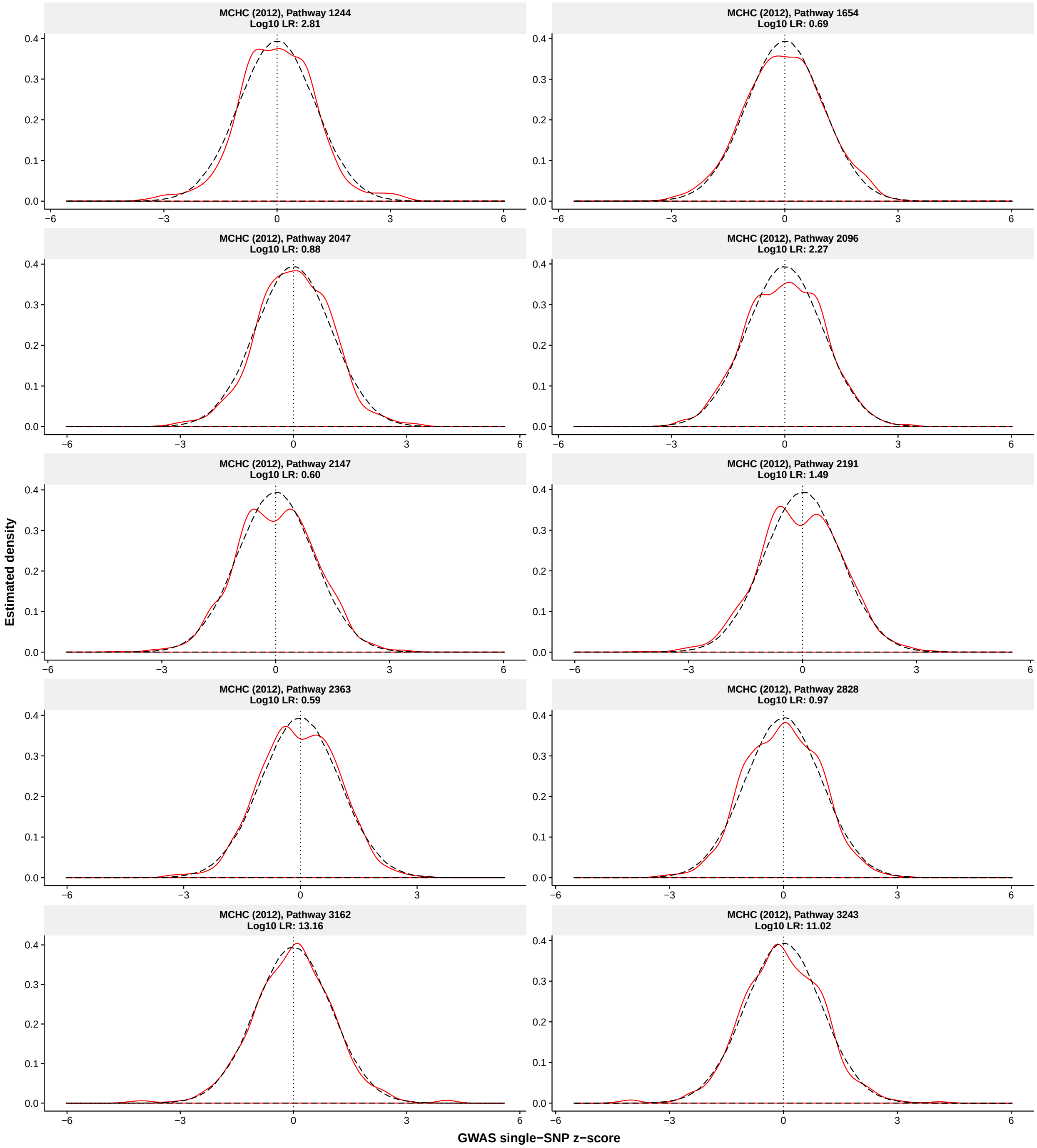

Mean cell volume [46]

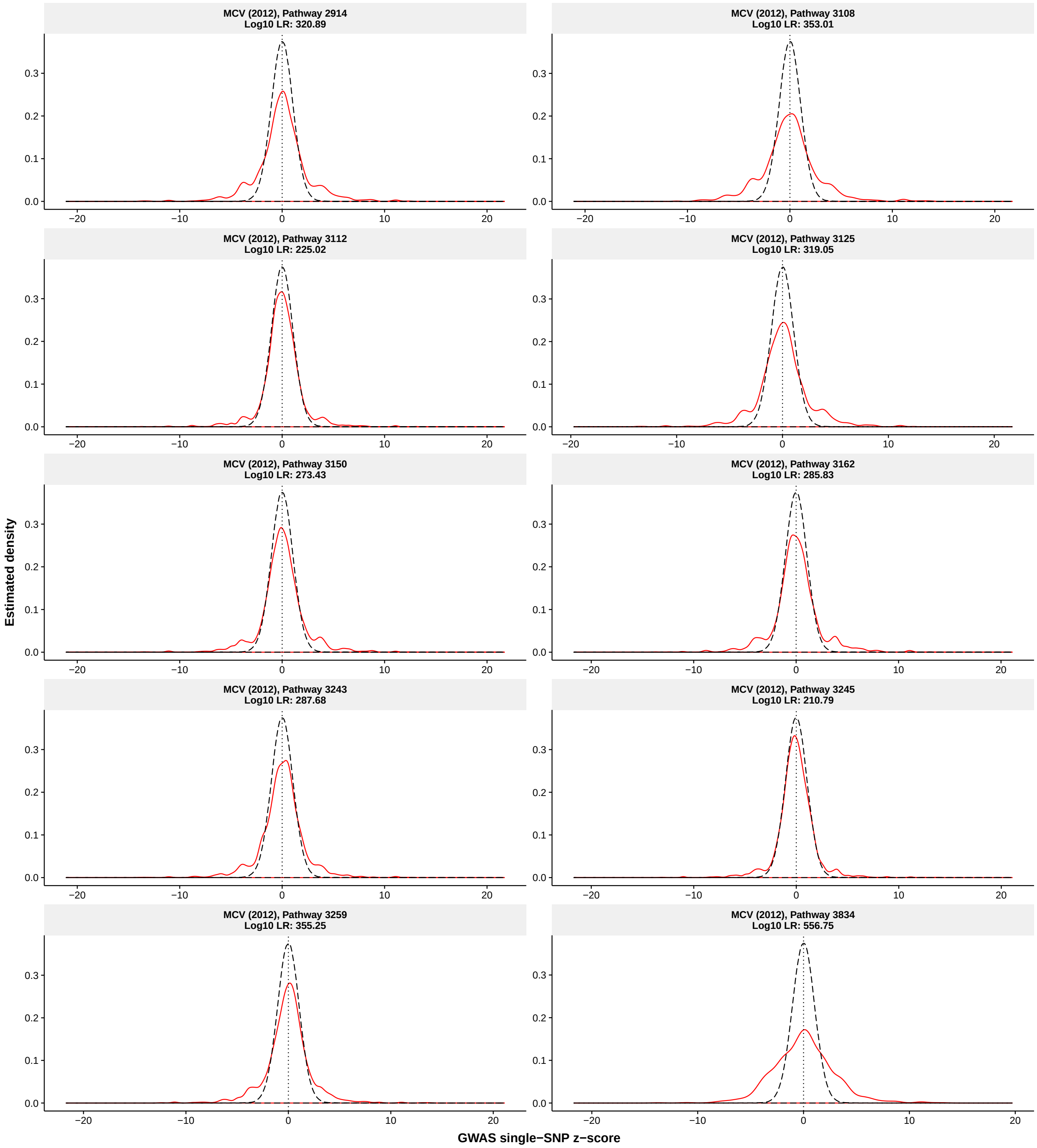

Myocardial infarction [18]

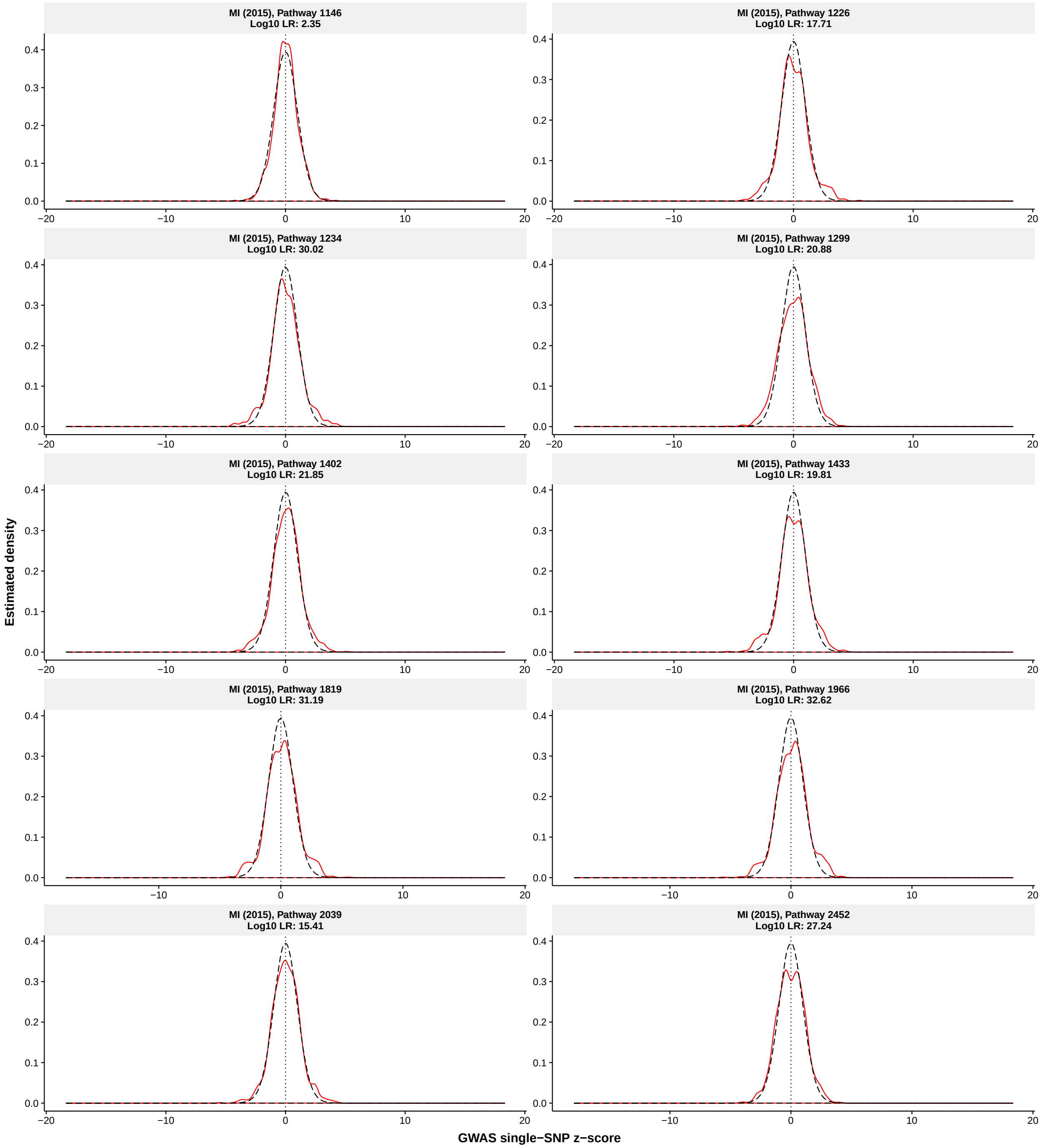

Neuroticism [40]

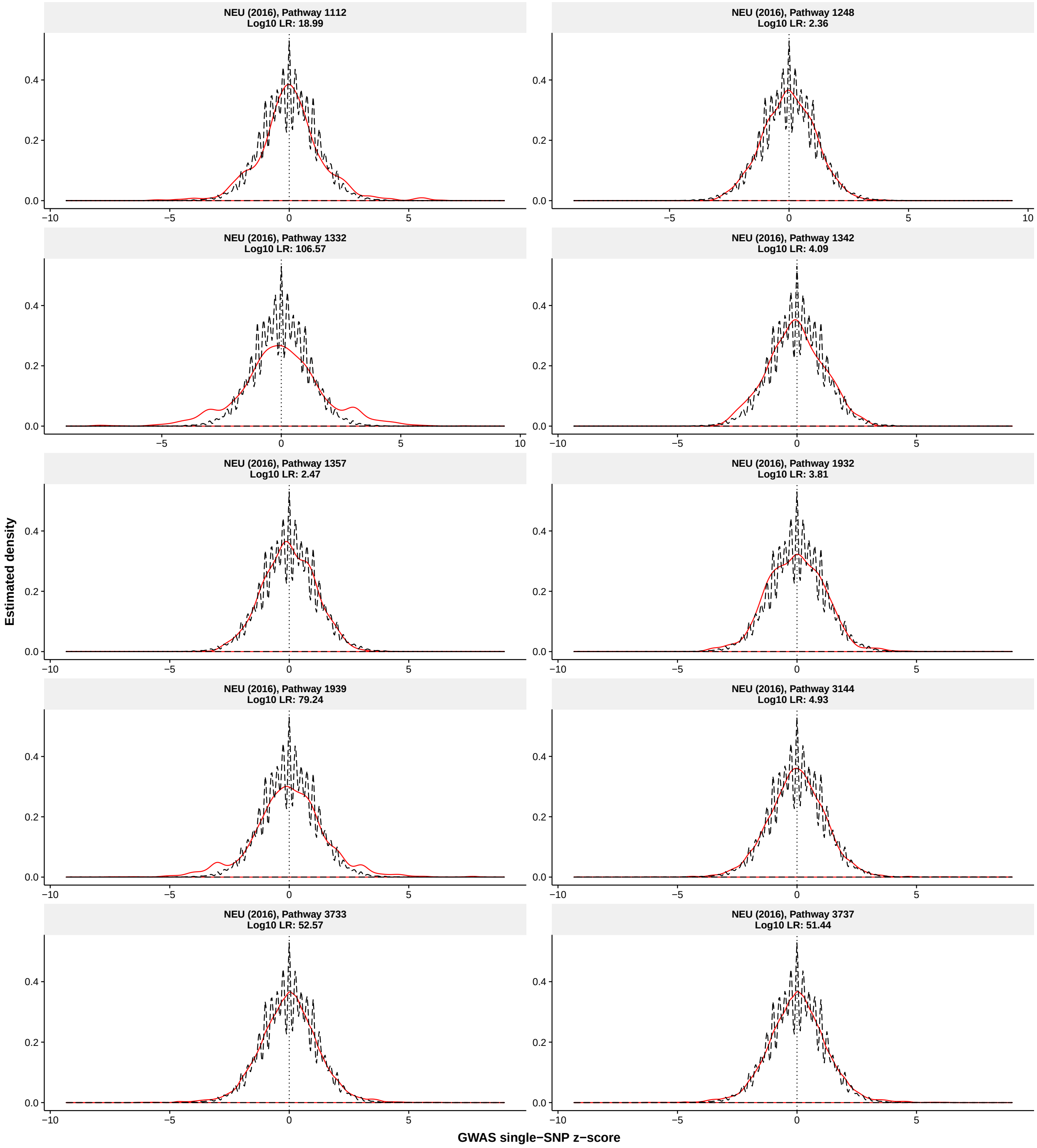

Packed cell volume [46]

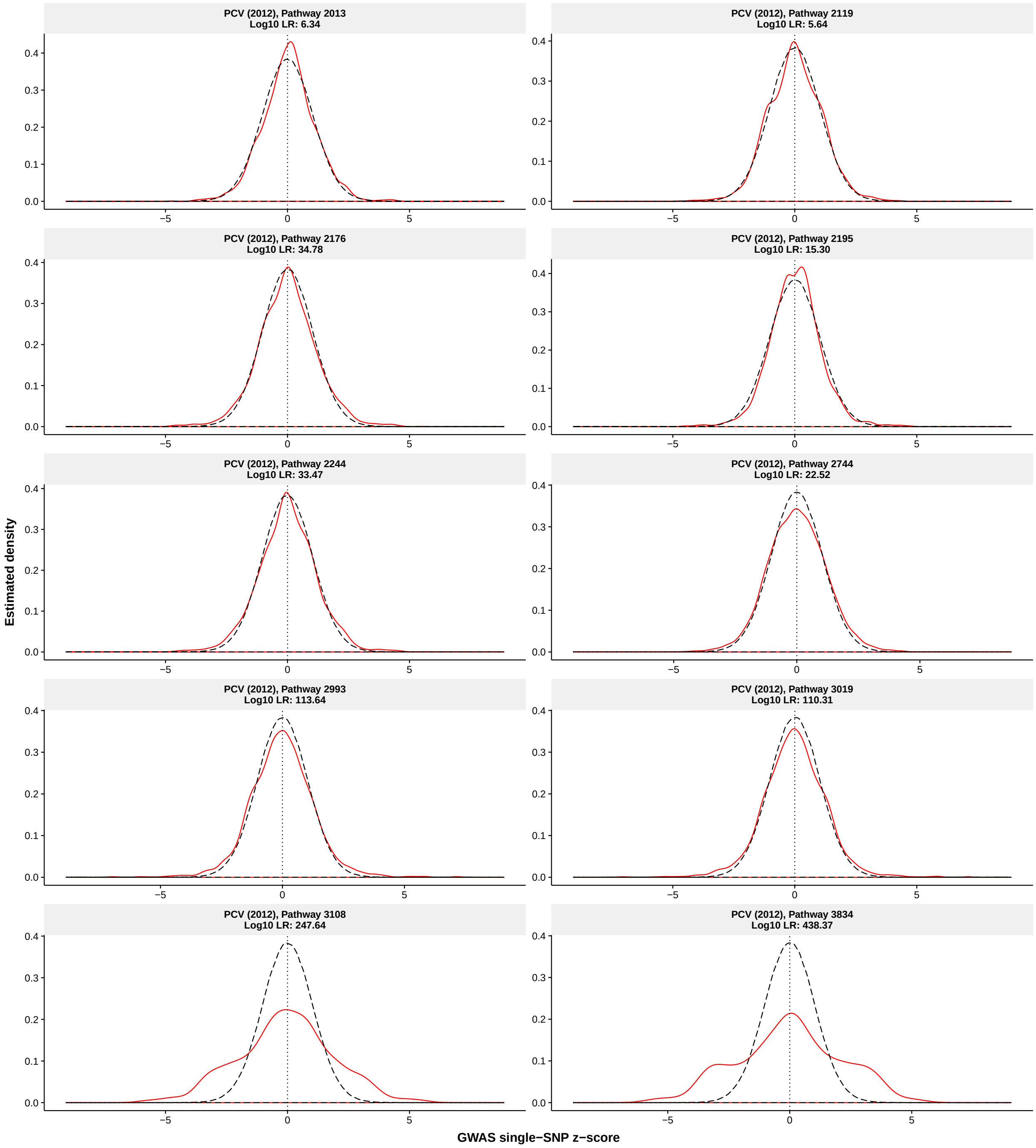

Rheumatoid arthritis [26]

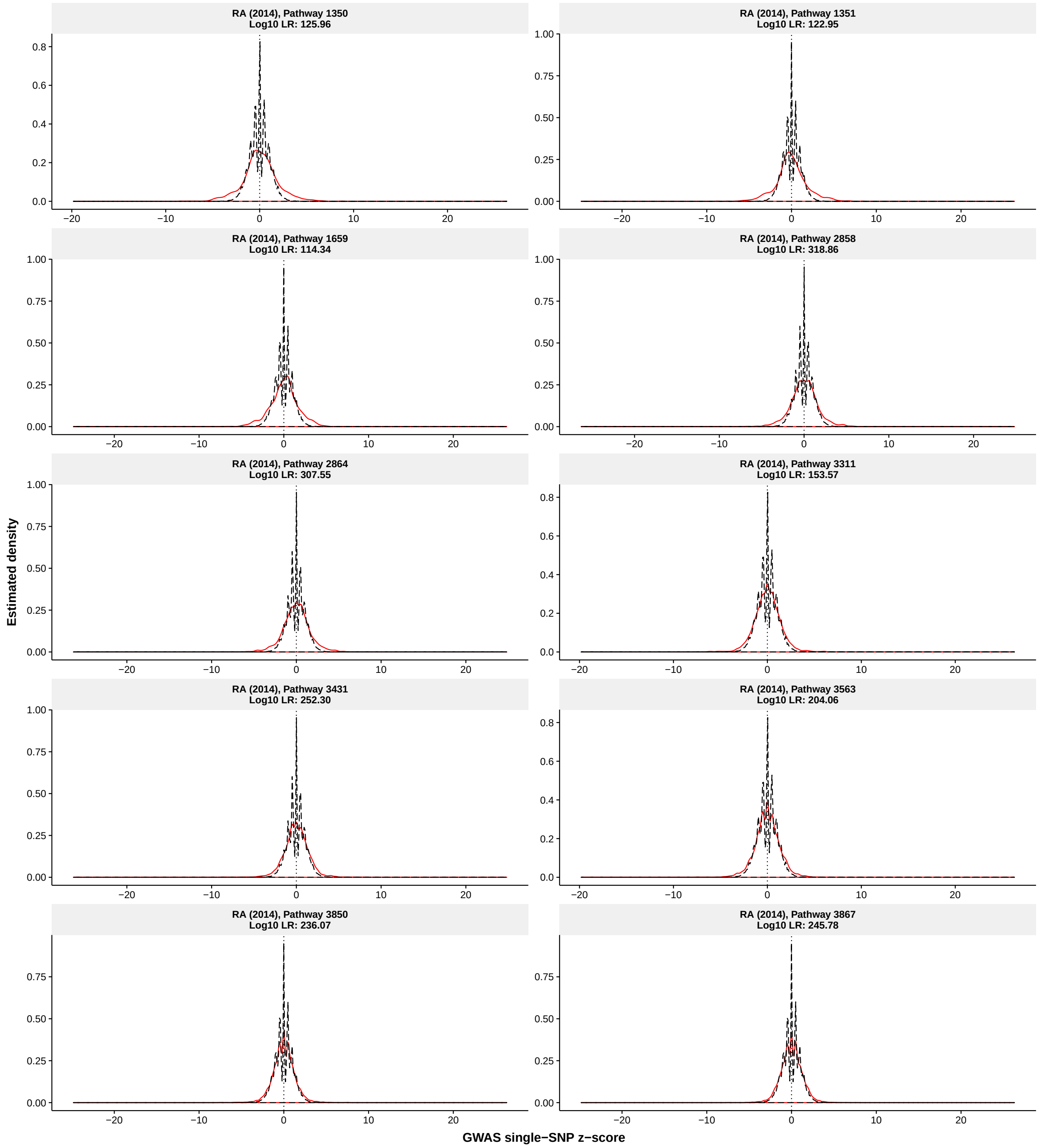

Red blood cell count [46]

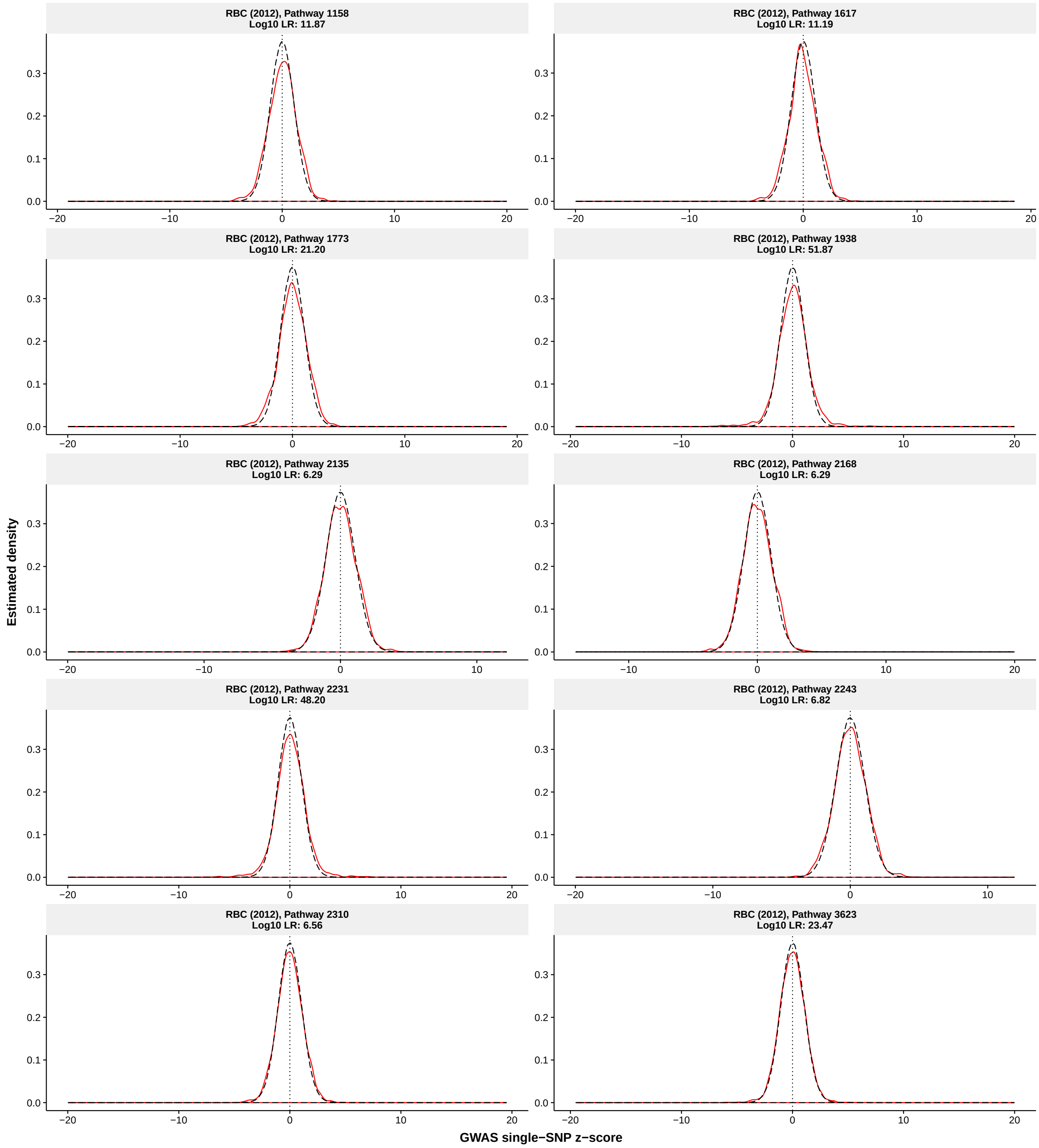

Schizophrenia [37]

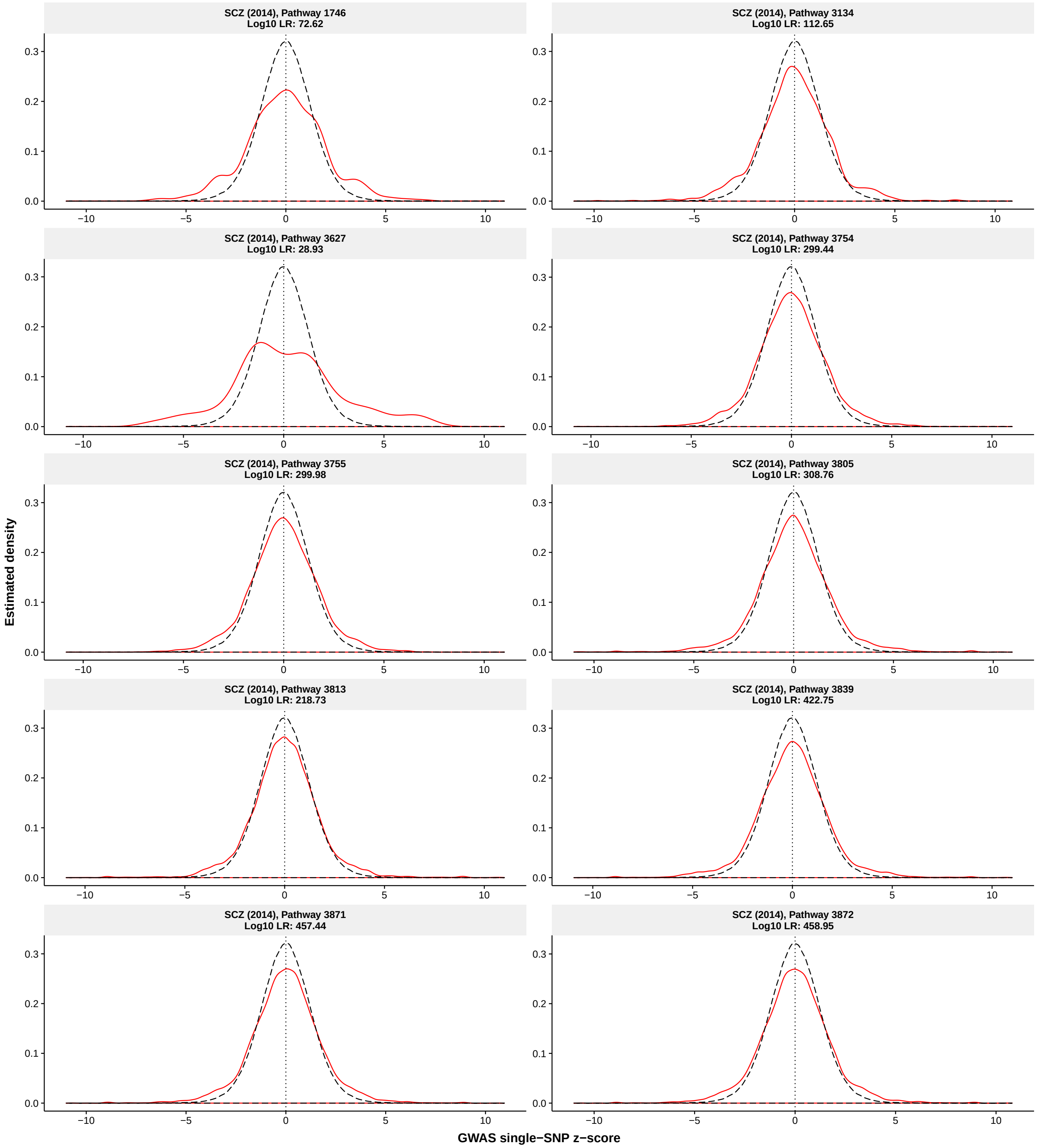

Type 2 diabetes [41]

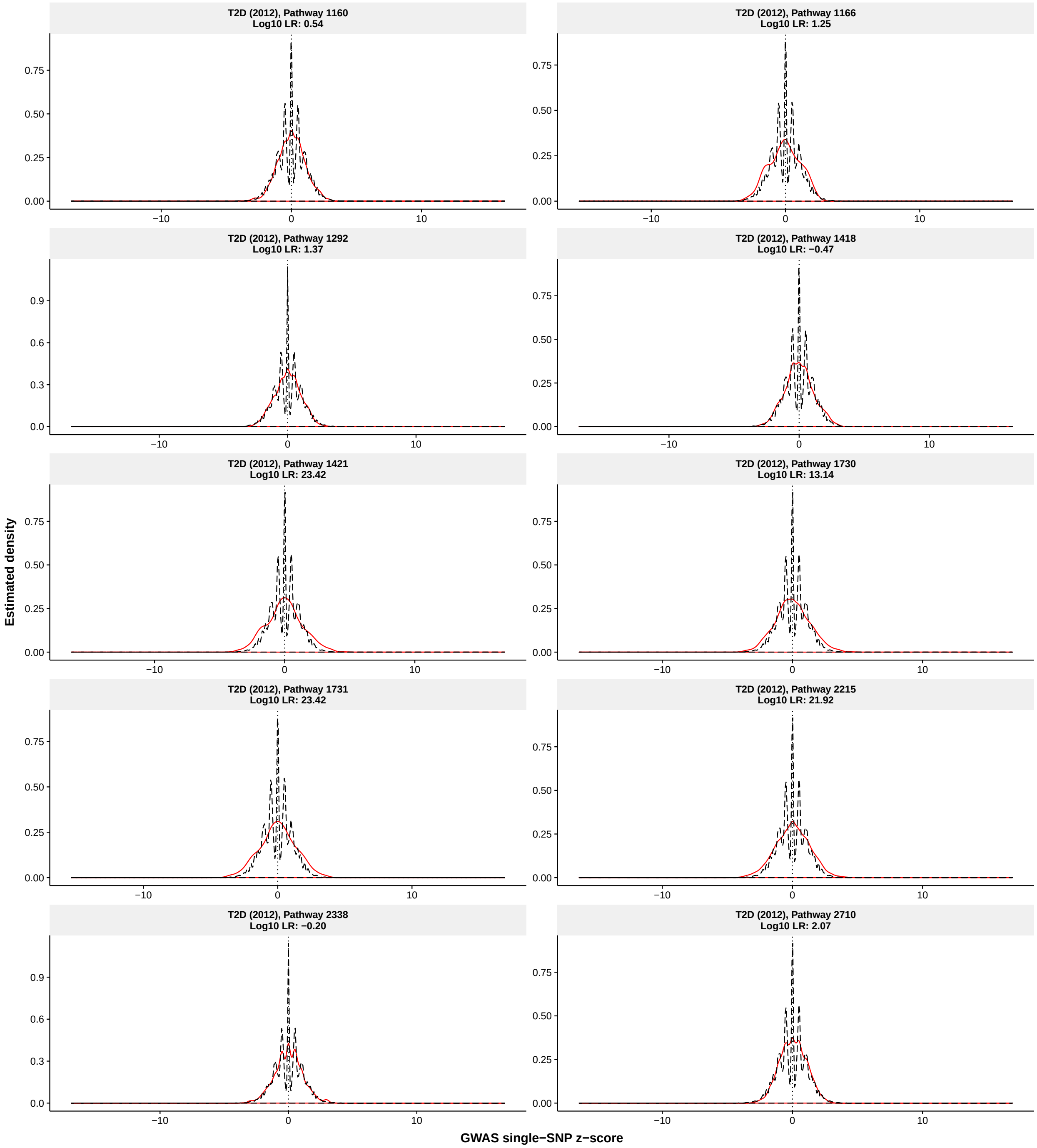

Total cholesterol [22]

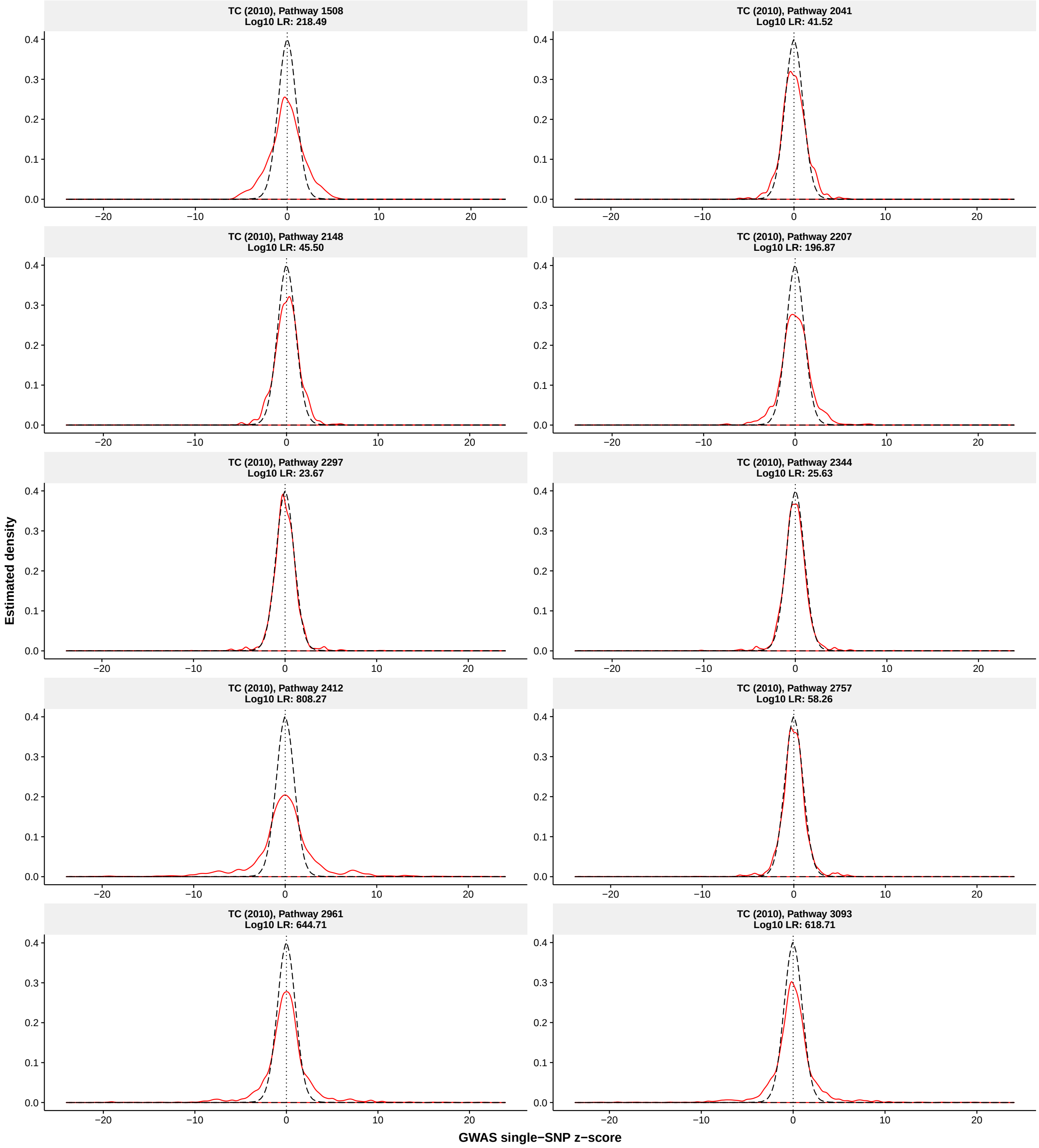

Triglycerides [22]

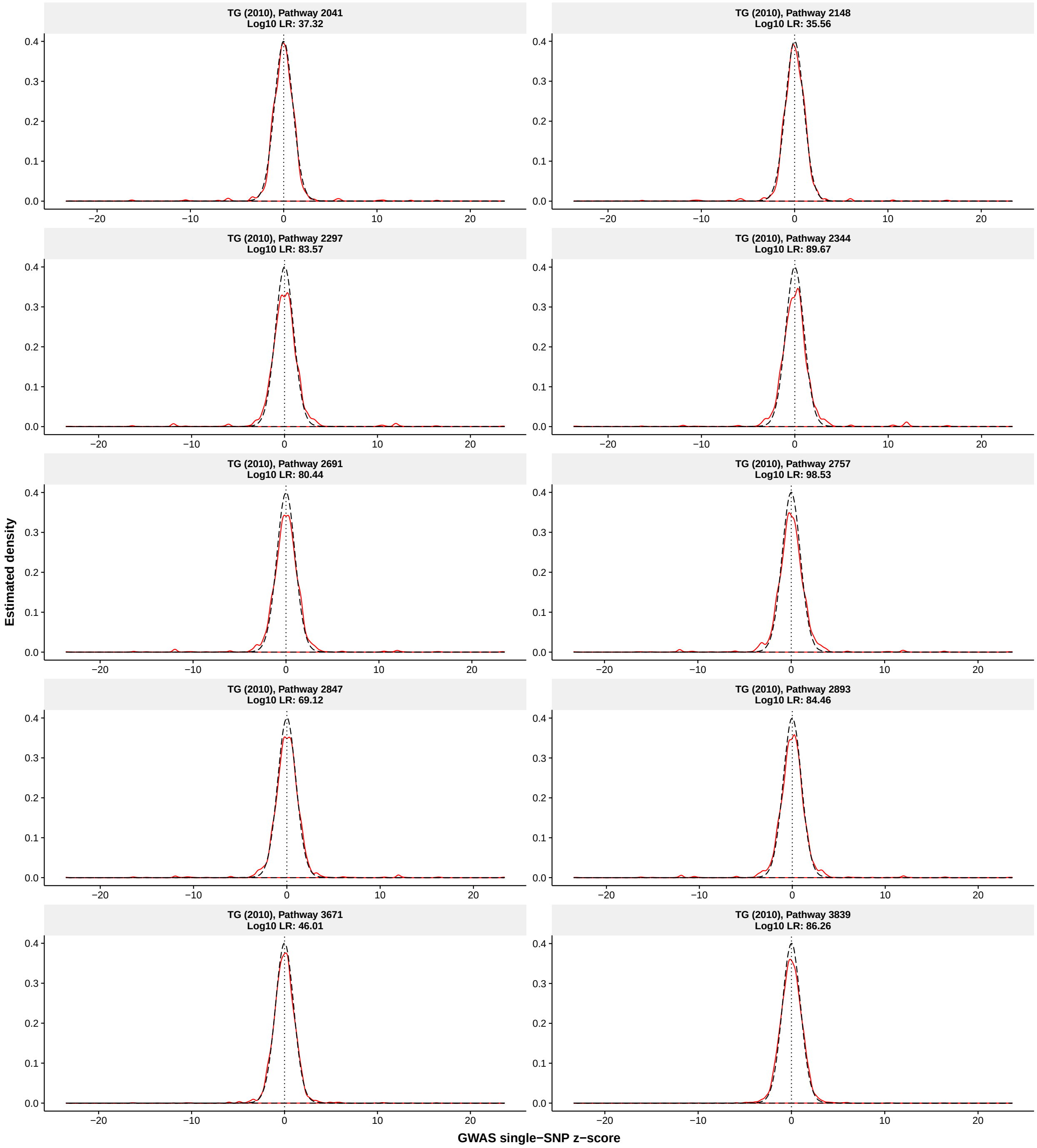

Ulcerative colitis [11]

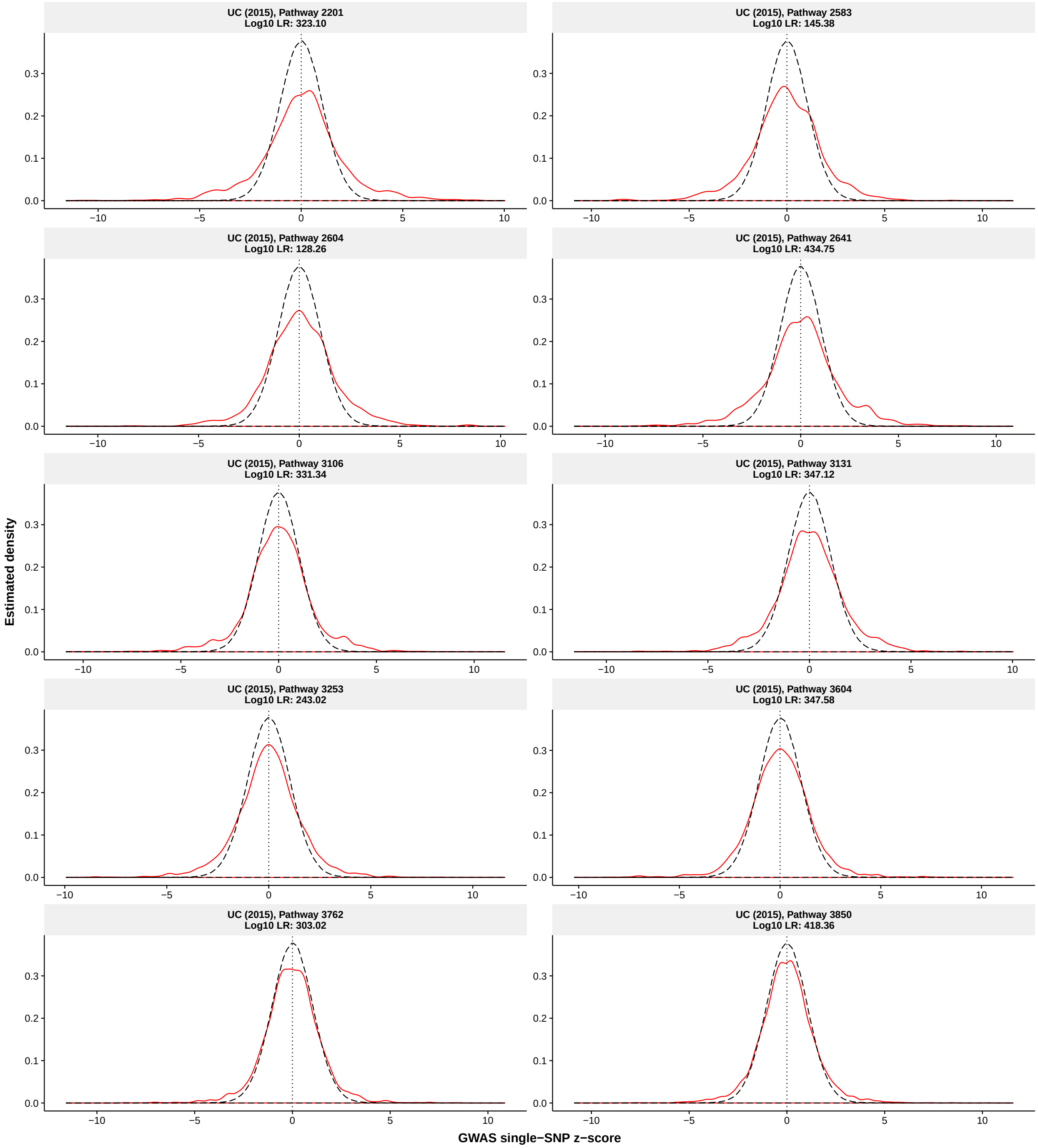

Serum urate concentrations [43]

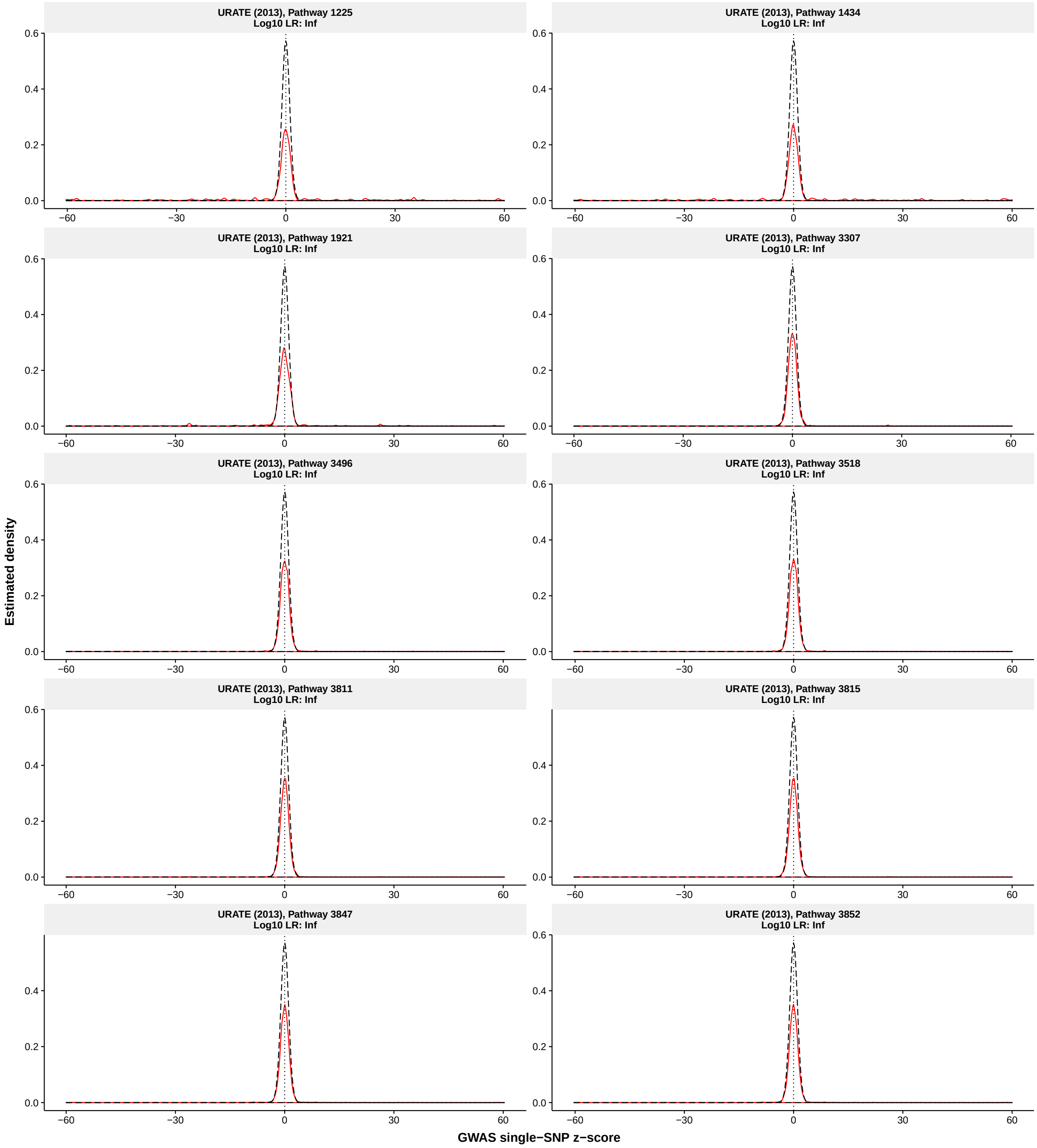

Waist-to-hip ratio adjusted for body mass index [36]

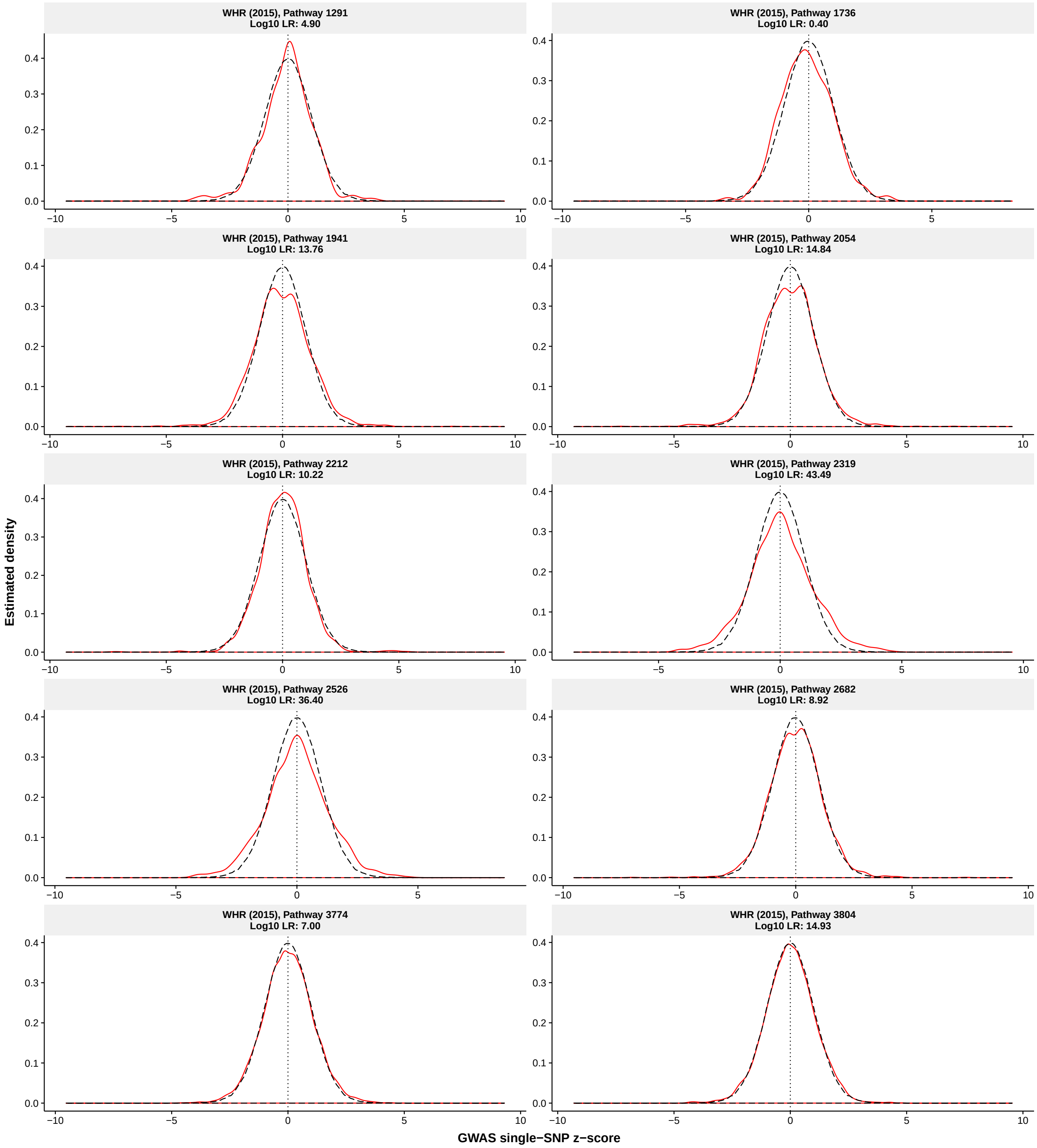

26. Supplementary Figure 18. Compare the likelihood ratio (LR) with the enrichment Bayes factor (BF). Simulation details are provided in **Supplementary Figure 1**. For each simulated dataset, we compute a sanity check LR as defined in **Supplementary Figure 17**, and use RSS-E to obtain an enrichment BF. In each panel, “competitive null” indicates datasets simulated from the baseline model (i.e. no enrichment, $M_0 : \theta = 0$), and “pathway enriched” indicates datasets simulated from the enrichment model ($M_1 : \theta > 0$). Dashed lines are reference lines with slope one and intercept zero.

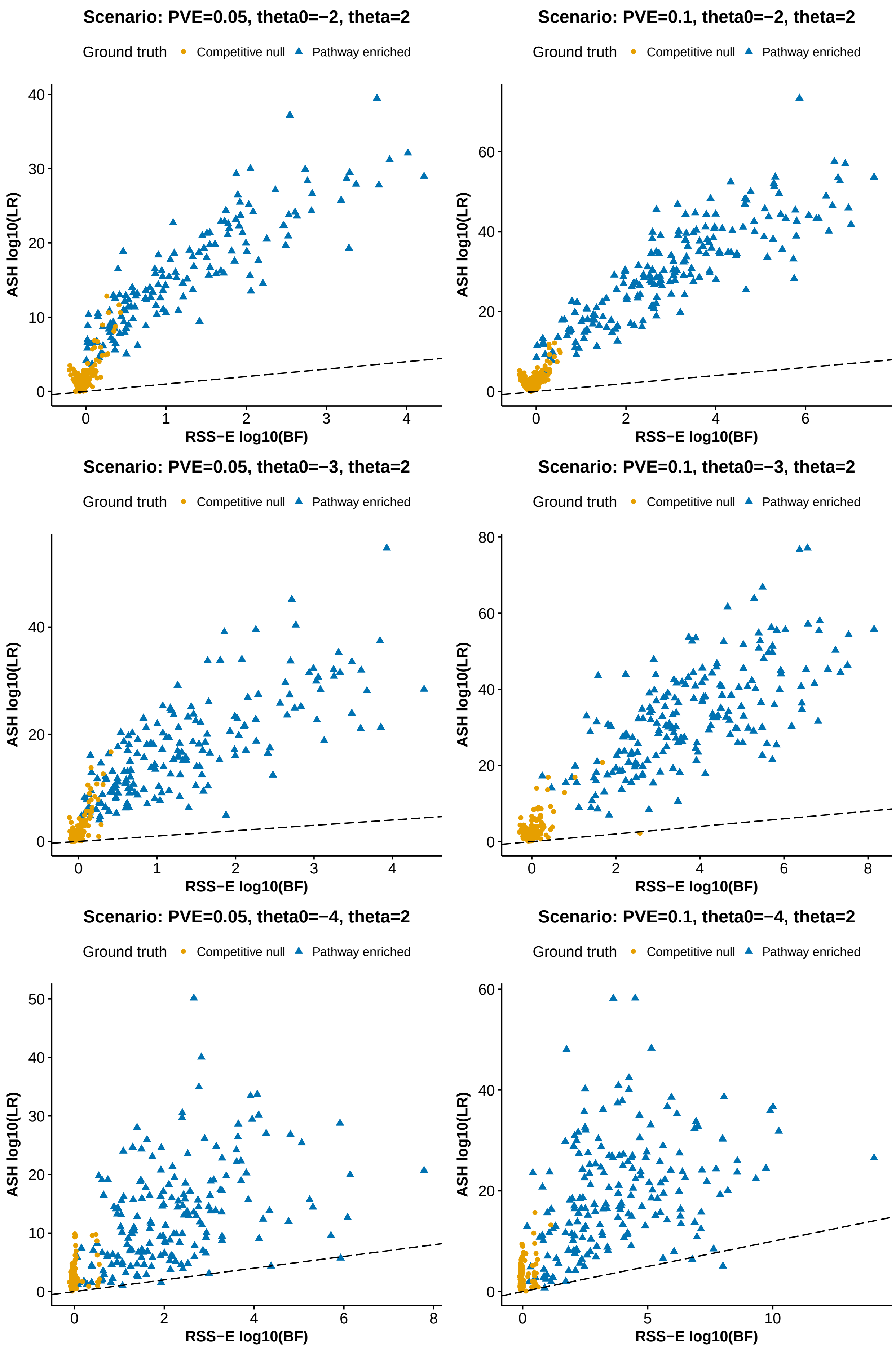
Scenario: PVE=0.05, theta0=−2, theta=2
Ground truth
Competitive null
Pathway enriched
ASH log10(LR)
RSS−E log10(BF)
Scenario: PVE=0.1, theta0=−2, theta=2
Ground truth
Competitive null
Pathway enriched
ASH log10(LR)
RSS−E log10(BF)
Scenario: PVE=0.05, theta0=−3, theta=2
Ground truth
Competitive null
Pathway enriched
ASH log10(LR)
RSS−E log10(BF)
Scenario: PVE=0.1, theta0=−3, theta=2
Ground truth
Competitive null
Pathway enriched
ASH log10(LR)
RSS−E log10(BF)
Scenario: PVE=0.05, theta0=−4, theta=2
Ground truth
Competitive null
Pathway enriched
ASH log10(LR)
RSS−E log10(BF)
Scenario: PVE=0.1, theta0=−4, theta=2
Ground truth
Competitive null
Pathway enriched
ASH log10(LR)
RSS−E log10(BF)

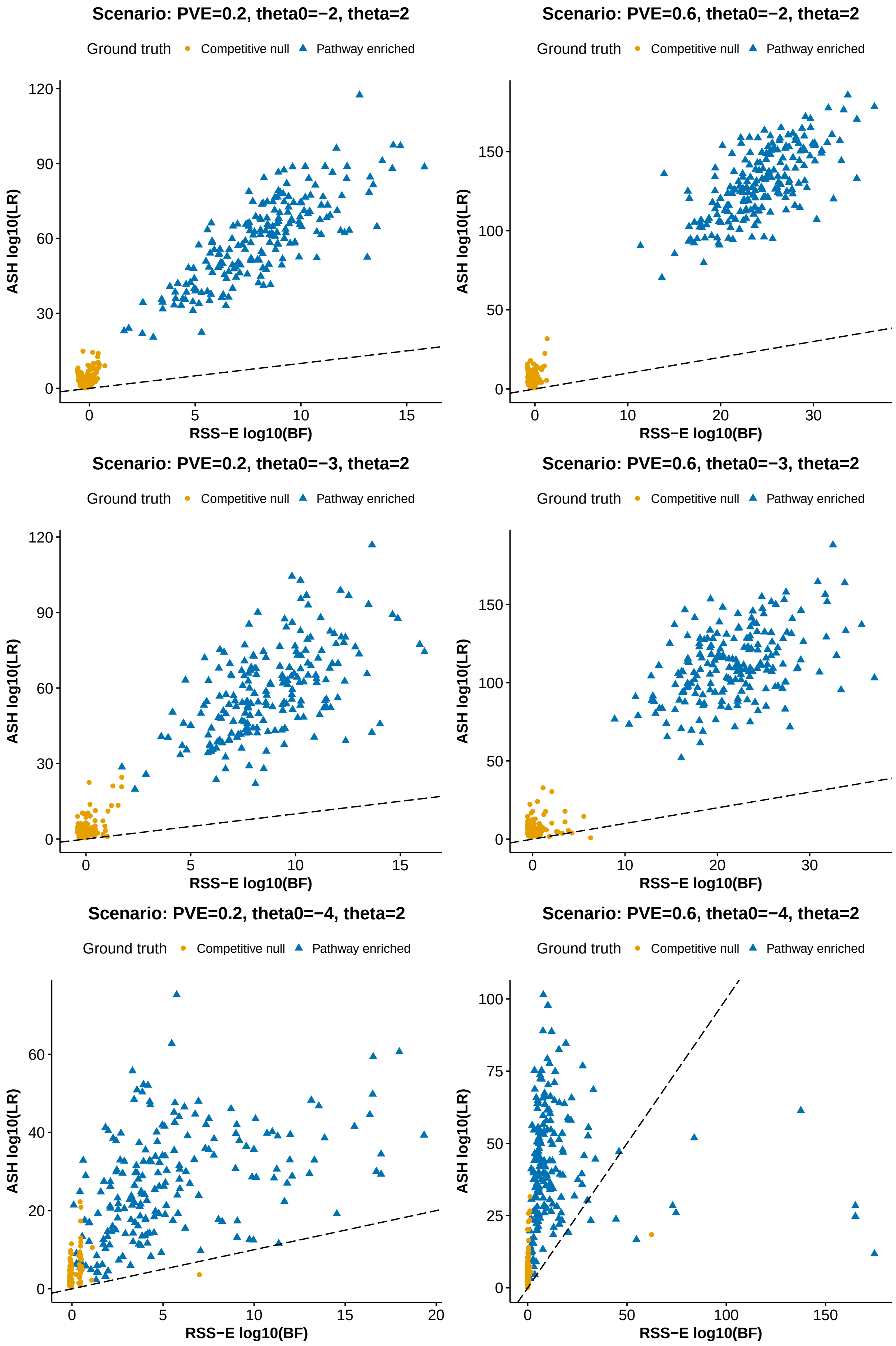

Scenario: PVE=0.2, theta0=−2, theta=2
Ground truth
Competitive null
Pathway enriched
ASH log10(LR)
RSS−E log10(BF)
0
30
60
90
120
0
5
10
15
Scenario: PVE=0.6, theta0=−2, theta=2
Ground truth
Competitive null
Pathway enriched
ASH log10(LR)
RSS−E log10(BF)
0
50
100
150
0
10
20
30
Scenario: PVE=0.2, theta0=−3, theta=2
Ground truth
Competitive null
Pathway enriched
ASH log10(LR)
RSS−E log10(BF)
0
30
60
90
120
0
5
10
15
Scenario: PVE=0.6, theta0=−3, theta=2
Ground truth
Competitive null
Pathway enriched
ASH log10(LR)
RSS−E log10(BF)
0
50
100
150
0
10
20
30
Scenario: PVE=0.2, theta0=−4, theta=2
Ground truth
Competitive null
Pathway enriched
ASH log10(LR)
RSS−E log10(BF)
0
20
40
60
0
5
10
15
20
Scenario: PVE=0.6, theta0=−4, theta=2
Ground truth
Competitive null
Pathway enriched
ASH log10(LR)
RSS−E log10(BF)
0
25
50
75
100
0
50
100
150

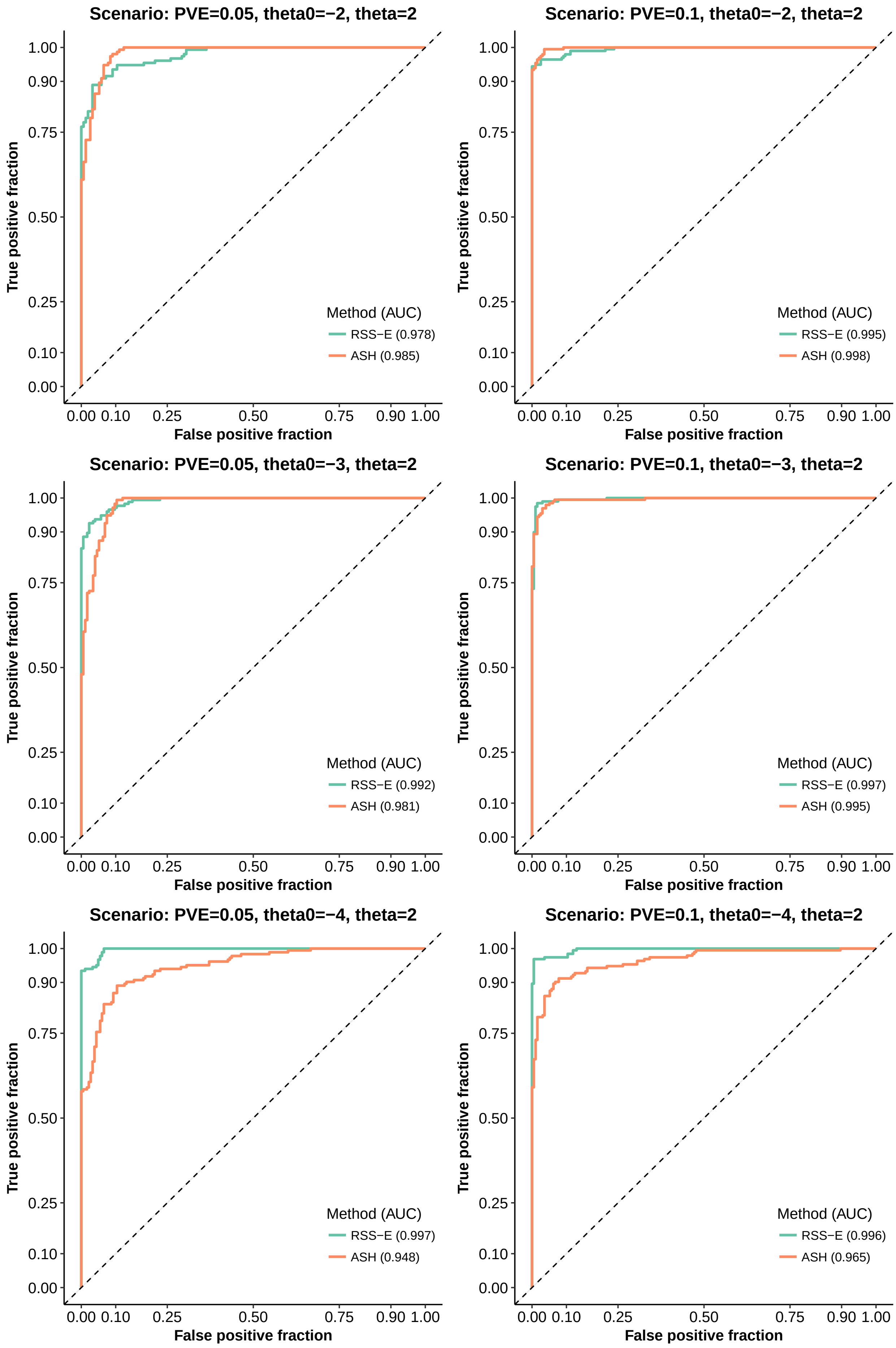

Scenario: PVE=0.05, theta0=−2, theta=2
True positive fraction
False positive fraction
Method (AUC)
RSS−E (0.978)
ASH (0.985)
Scenario: PVE=0.1, theta0=−2, theta=2
True positive fraction
False positive fraction
Method (AUC)
RSS−E (0.995)
ASH (0.998)
Scenario: PVE=0.05, theta0=−3, theta=2
True positive fraction
False positive fraction
Method (AUC)
RSS−E (0.992)
ASH (0.981)
Scenario: PVE=0.1, theta0=−3, theta=2
True positive fraction
False positive fraction
Method (AUC)
RSS−E (0.997)
ASH (0.995)
Scenario: PVE=0.05, theta0=−4, theta=2
True positive fraction
False positive fraction
Method (AUC)
RSS−E (0.997)
ASH (0.948)
Scenario: PVE=0.1, theta0=−4, theta=2
True positive fraction
False positive fraction
Method (AUC)
RSS−E (0.996)
ASH (0.965)

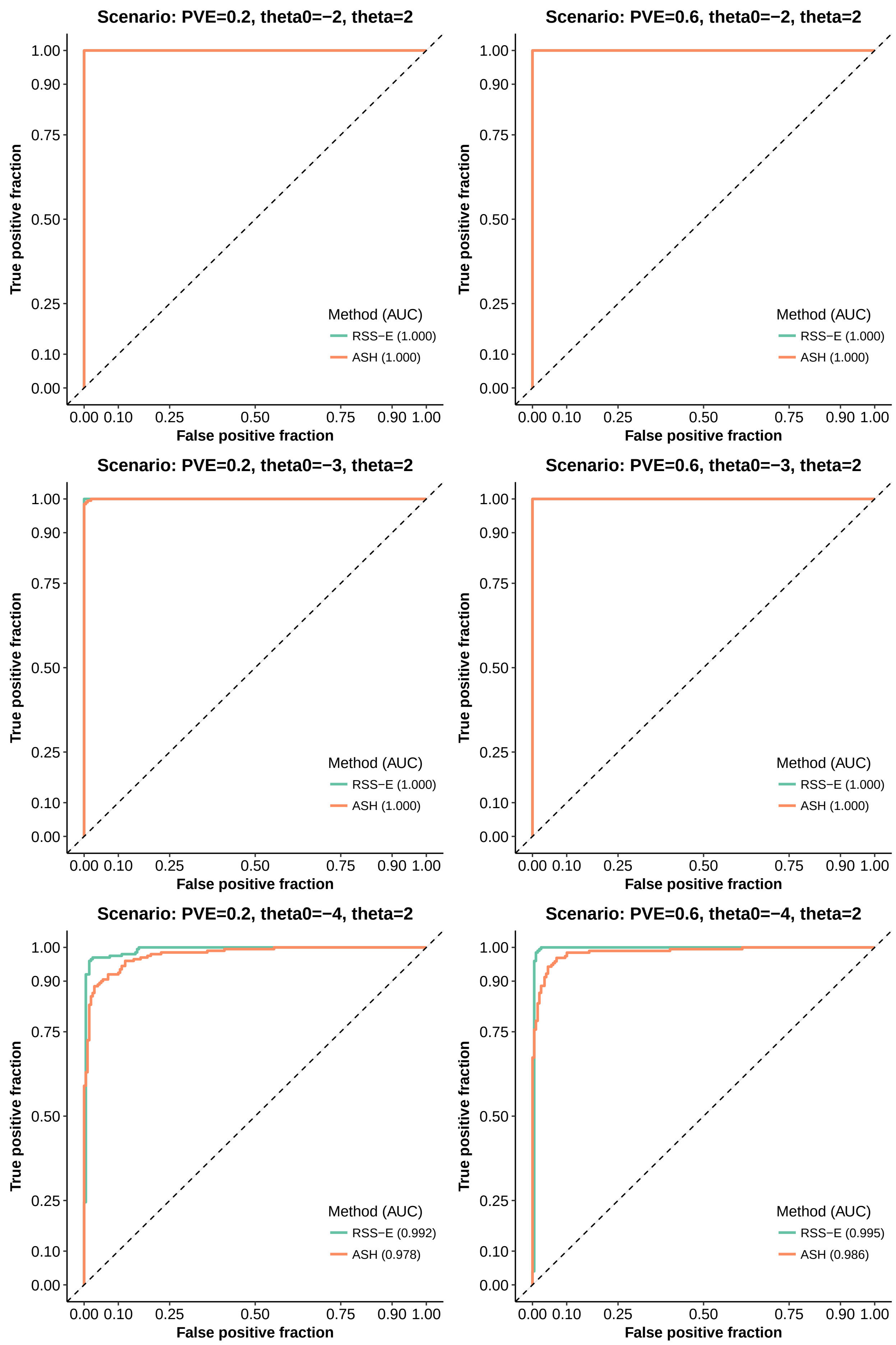
Scenario: PVE=0.2, theta0=−2, theta=2
True positive fraction
1.00
0.90
0.75
0.50
0.25
0.10
0.00
False positive fraction
0.00 0.10 0.25 0.50 0.75 0.90 1.00
Method (AUC)
RSS−E (1.000)
ASH (1.000)
Scenario: PVE=0.6, theta0=−2, theta=2
True positive fraction
False positive fraction
Method (AUC)
RSS−E (1.000)
ASH (1.000)
Scenario: PVE=0.2, theta0=−3, theta=2
True positive fraction
False positive fraction
Method (AUC)
RSS−E (1.000)
ASH (1.000)
Scenario: PVE=0.6, theta0=−3, theta=2
True positive fraction
False positive fraction
Method (AUC)
RSS−E (1.000)
ASH (1.000)
Scenario: PVE=0.2, theta0=−4, theta=2
True positive fraction
False positive fraction
Method (AUC)
RSS−E (0.992)
ASH (0.978)
Scenario: PVE=0.6, theta0=−4, theta=2
True positive fraction
False positive fraction
Method (AUC)
RSS−E (0.995)
ASH (0.986)

27. Supplementary Figure 19. Bayes factors for enrichment of genetic associations near all genes in 31 phenotypes. For each phenotype, we use RSS-E to assess the genome-wide near-gene enrichment hypothesis that SNPs within 100 kb of the transcribed region of any autosomal protein-coding gene (18,313 in total in the present study; see **Supplementary Figure 16**) are more likely to be associated with the phenotype. The x-axis shows Bayes factor (BF) for each genome-wide near-gene enrichment hypothesis. The x-axis uses a normal scale inside the range [-1.5, 1.5], and a logarithmic scale (base 10) outside (-1.5, 1.5).

For each phenotype we consider

- Model M_0: each SNP has equal chance of being associated with the phenotype;
- Model M_1: SNPs inside a target gene set are more often associated with the phenotype;
- Model M_2: near-gene SNPs are more often associated with the phenotype.

To assess the enrichment of target gene set we compute the following BF:

$$\text{BF}(M_1|M_0) = \frac{\Pr(\text{Data}|M_1)}{\Pr(\text{Data}|M_0)}.$$

Here we compute another BF to assess the genome-wide near-gene enrichment:

$$\text{BF}(M_2|M_0) = \frac{\Pr(\text{Data}|M_2)}{\Pr(\text{Data}|M_0)}.$$

For a given phenotype, if $\text{BF}(M_1|M_0) > \text{BF}(M_2|M_0)$, then the observed GWAS summary statistics show more support for the target gene set enrichment than near-gene enrichment, and vice versa. See [2] for more discussions of interpreting enrichment BFs.

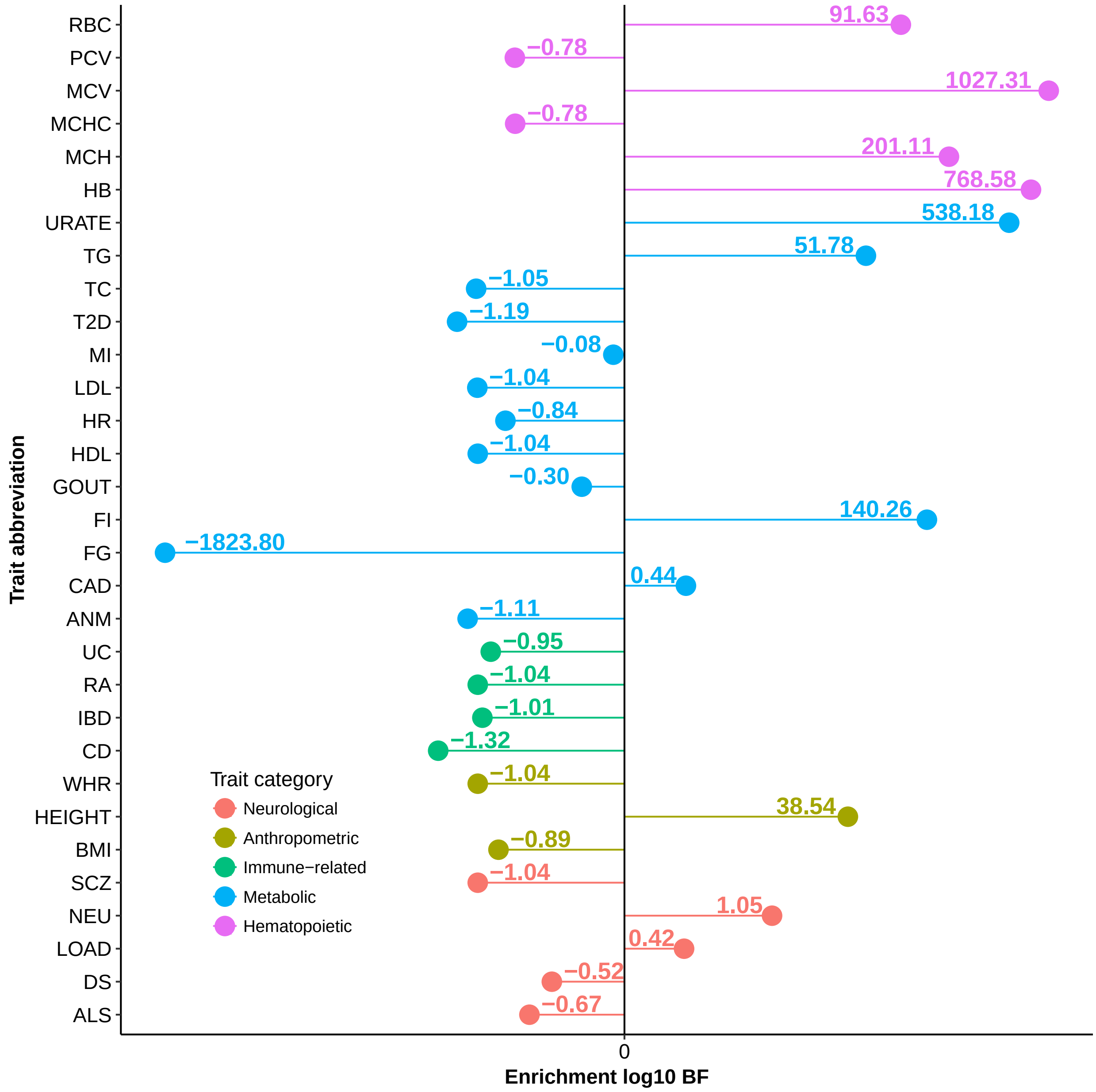

Trait abbreviation
RBC
PCV
MCV
MCHC
MCH
HB
URATE
TG
TC
T2D
MI
LDL
HR
HDL
GOUT
FI
FG
CAD
ANM
UC
RA
IBD
CD
WHR
HEIGHT
BMI
SCZ
NEU
LOAD
DS
ALS
91.63
−0.78
1027.31
−0.78
201.11
768.58
538.18
51.78
−1.05
−1.19
−0.08
−1.04
−0.84
−1.04
−0.30
140.26
−1823.80
0.44
−1.11
−0.95
−1.04
−1.01
−1.32
−1.04
38.54
−0.89
−1.04
1.05
0.42
−0.52
−0.67
Trait category
Neurological
Anthropometric
Immune−related
Metabolic
Hematopoietic
0
Enrichment log10 BF

28. Supplementary Figure 20. Enrichment analyses of randomly selected near-gene SNPs based on real GWAS summary statistics. For a given dataset of GWAS summary statistics, we select a target gene set from the top enriched biological pathways or tissue-based gene sets, and then create "null" sets by randomly drawing near-gene SNPs (defined in **Supplementary Figure 2**), the sizes of which roughly match the target gene set. We perform enrichment analysis of these random sets of near-gene SNPs on the same GWAS summary data, and compare their enrichment Bayes factors (BFs) with BFs of the actual gene sets. We also use these simulated BFs to estimate false discovery rate (FDR) at a given cutoff of BF (see the `R` codes below).

```
# a naive FDR estimate function
# "observed": the real gene sets
# "simulated": the random sets of near-gene SNPs

estimate_fdr <- function(log10.bf, case, cutoff) {
  R <- sum(log10.bf[case=="observed"] >= cutoff) / sum(case=="observed")
  V <- sum(log10.bf[case=="simulated"] >= cutoff) / sum(case=="simulated")
  FDR <- V/R
  return(FDR)
}
```

We perform this type of simulation on the following two real-data examples. The blue dots indicate the real gene sets. The yellow triangles indicate the random sets of near-gene SNPs. The red horizontal lines indicate $\log_{10}\text{BF} = 8$.

28.1. *Example 1.*

- GWAS summary statistics: low-density lipoprotein [22].
- Target gene set: *Chylomicron-mediated lipid transport* (Reactome, Pathway Commons 2), 1,139 SNPs in this pathway, $\log_{10}\text{BF} = 62.8$.
- Random sets of near-gene SNPs: 2,100 sets, set size ~ Uniform(1,000, 2,000).

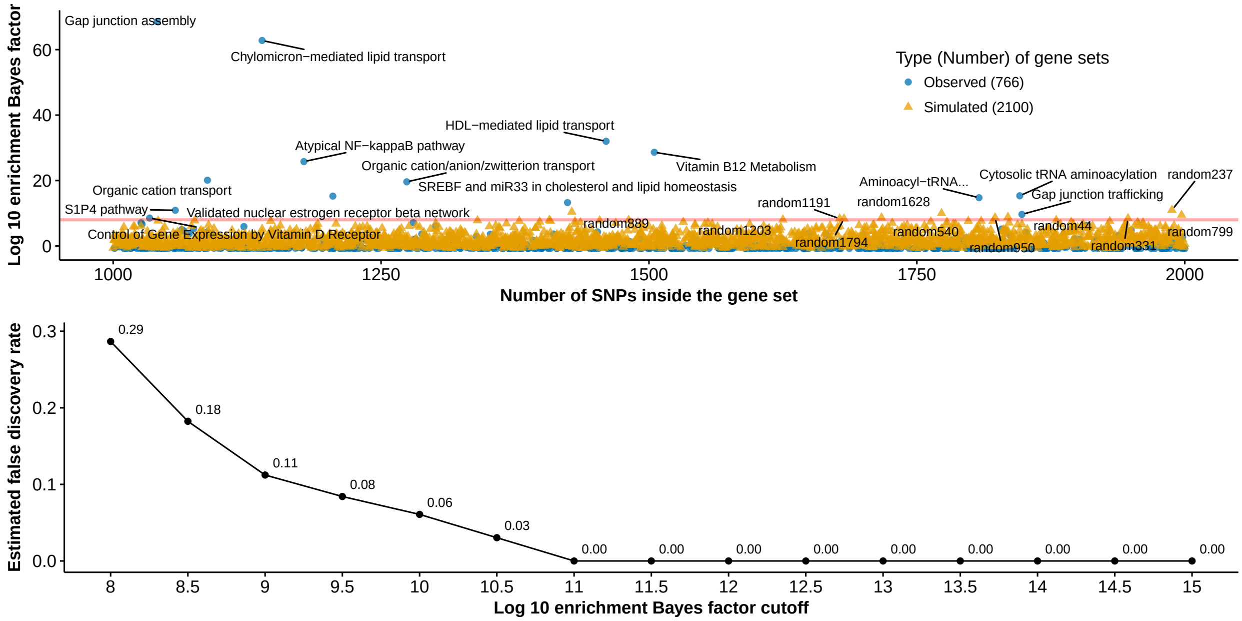

28.2. *Example 2.*

- GWAS summary statistics: late-onset Alzheimer's disease [39].
- Target gene set: *Liver distinctive cluster* [79], 9,013 SNPs in this gene set, $\log_{10} \mathrm{BF} = 35.7$.
- Random sets of near-gene SNPs: 1,000 sets, set size ~ Uniform(7,000, 13,000).

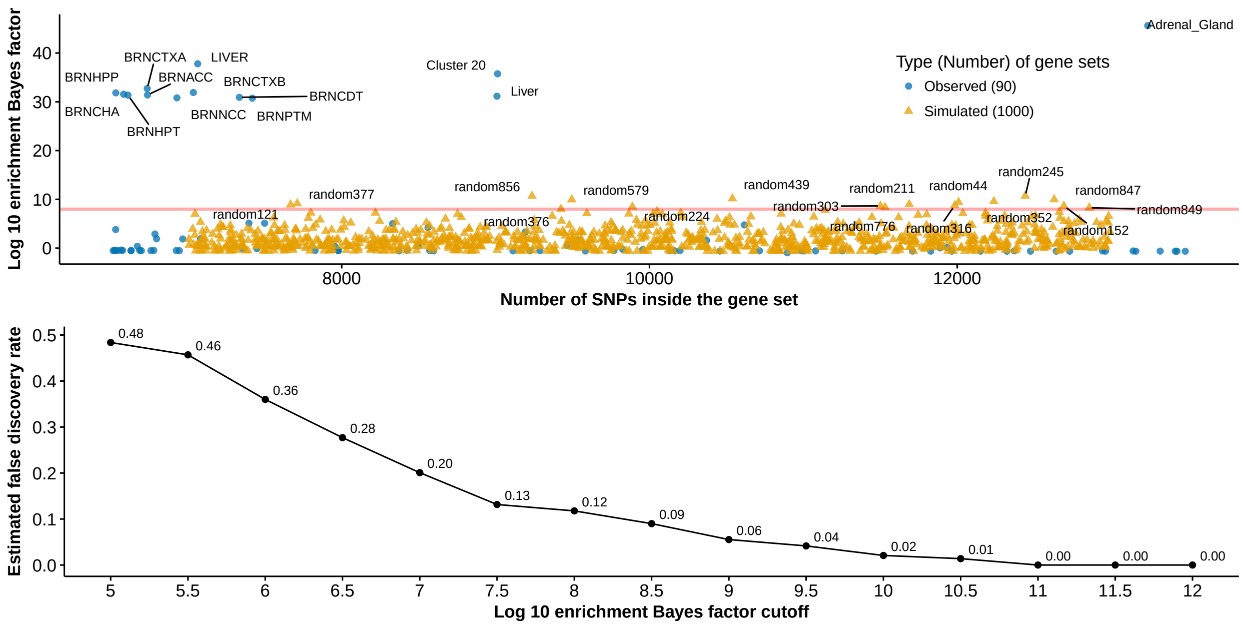

29. Supplementary Figure 21. Correlation between gene set enrichment Bayes factors (BFs) and 52 functional categories in 31 phenotypes. For each pair of phenotype and functional category, we plot the log 10 enrichment BF versus the proportion of functional SNPs for each of 4,026 gene sets (3,913 biological pathways and 113 tissue-based gene sets), and compute their Pearson correlation. In each plot, the black dashed line indicates the proportion of genome-wide SNPs falling into the given functional category; the red solid line indicates the proportion of near-gene SNPs (defined in **Supplementary Figure 2**) falling into the given functional category.

Below are the 52 functional categories used in [55].

```
##  [1] "Coding_UCSC"                        "Coding_UCSC.extend.500"
##  [3] "Conserved_LindbladToh"              "Conserved_LindbladToh.extend.500"
##  [5] "CTCF_Hoffman"                       "CTCF_Hoffman.extend.500"
##  [7] "DGF_ENCODE"                         "DGF_ENCODE.extend.500"
##  [9] "DHS_peaks_Trynka"                   "DHS_Trynka"
## [11] "DHS_Trynka.extend.500"              "Enhancer_Andersson"
## [13] "Enhancer_Andersson.extend.500"      "Enhancer_Hoffman"
## [15] "Enhancer_Hoffman.extend.500"        "FetalDHS_Trynka"
## [17] "FetalDHS_Trynka.extend.500"         "H3K27ac_Hnisz"
## [19] "H3K27ac_Hnisz.extend.500"           "H3K27ac_PGC2"
## [21] "H3K27ac_PGC2.extend.500"            "H3K4me1_peaks_Trynka"
## [23] "H3K4me1_Trynka"                     "H3K4me1_Trynka.extend.500"
## [25] "H3K4me3_peaks_Trynka"               "H3K4me3_Trynka"
## [27] "H3K4me3_Trynka.extend.500"          "H3K9ac_peaks_Trynka"
## [29] "H3K9ac_Trynka"                      "H3K9ac_Trynka.extend.500"
## [31] "Intron_UCSC"                        "Intron_UCSC.extend.500"
## [33] "PromoterFlanking_Hoffman"           "PromoterFlanking_Hoffman.extend.500"
## [35] "Promoter_UCSC"                      "Promoter_UCSC.extend.500"
## [37] "Repressed_Hoffman"                  "Repressed_Hoffman.extend.500"
## [39] "SuperEnhancer_Hnisz"                "SuperEnhancer_Hnisz.extend.500"
## [41] "TFBS_ENCODE"                        "TFBS_ENCODE.extend.500"
## [43] "Transcribed_Hoffman"                "Transcribed_Hoffman.extend.500"
## [45] "TSS_Hoffman"                        "TSS_Hoffman.extend.500"
## [47] "UTR_3_UCSC"                         "UTR_3_UCSC.extend.500"
## [49] "UTR_5_UCSC"                         "UTR_5_UCSC.extend.500"
## [51] "WeakEnhancer_Hoffman"               "WeakEnhancer_Hoffman.extend.500"
```

Functional category: Coding_UCSC

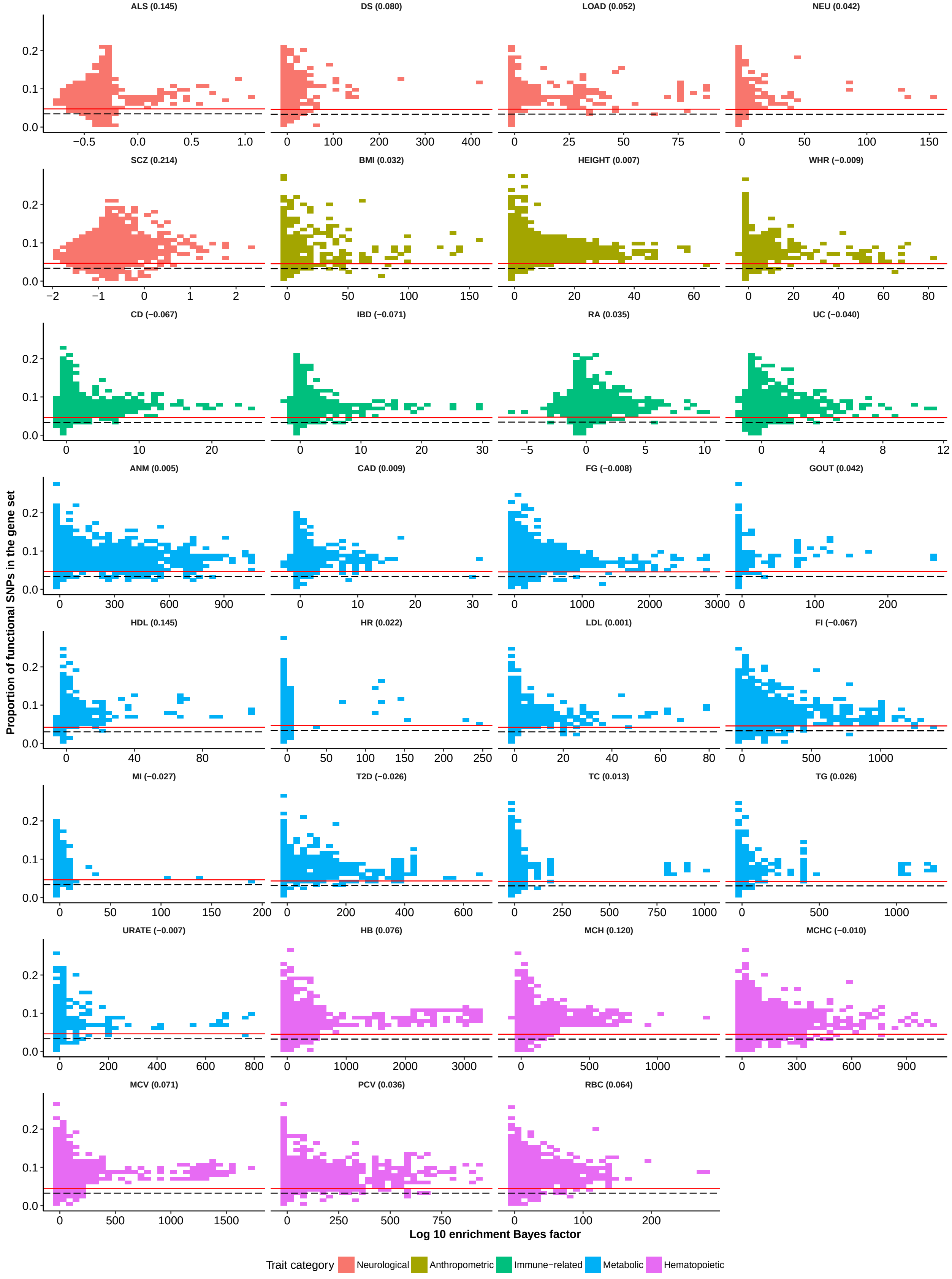

Functional category: Coding_UCSC.extend.500

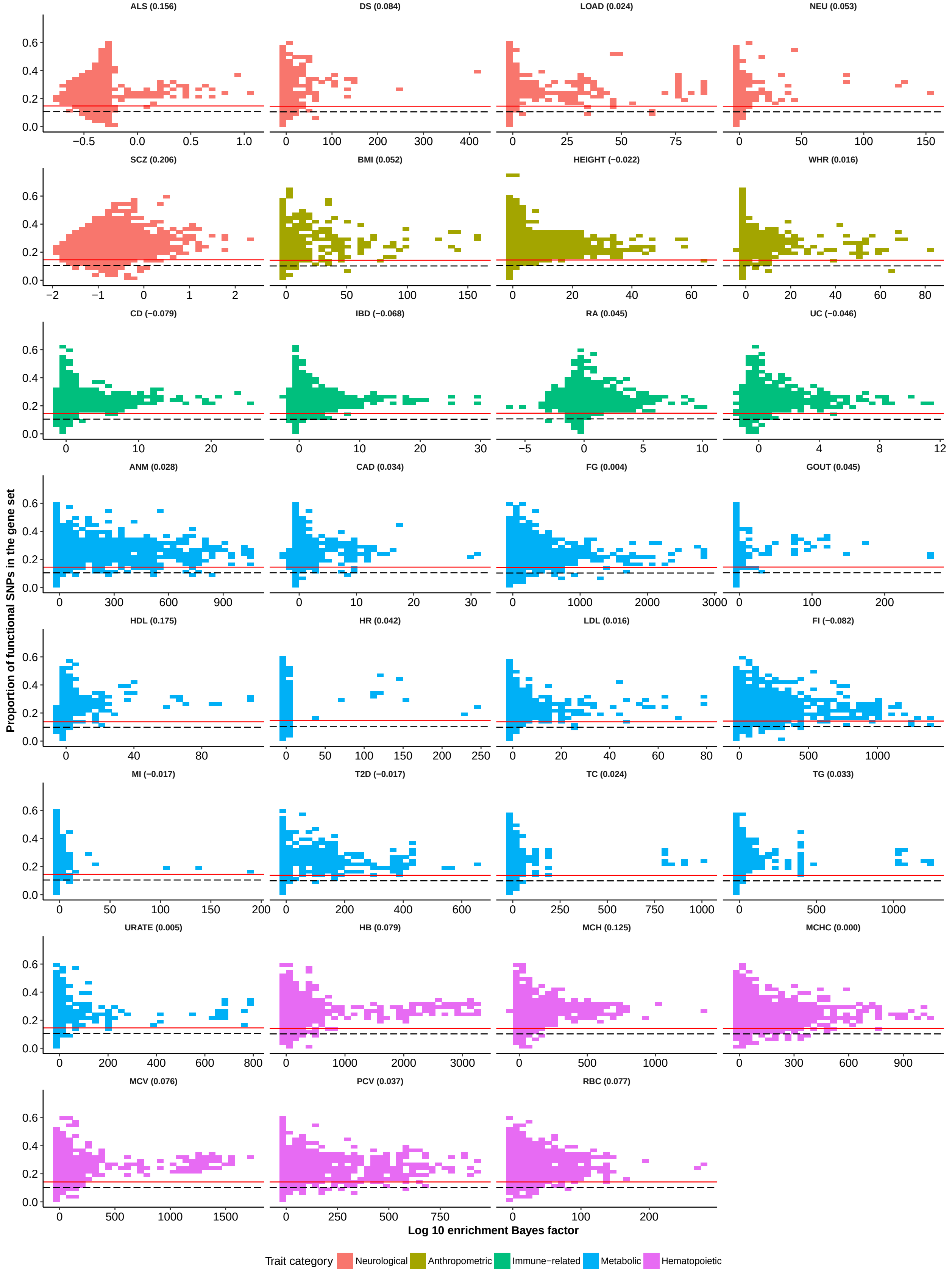

Functional category: Conserved_LindbladToh

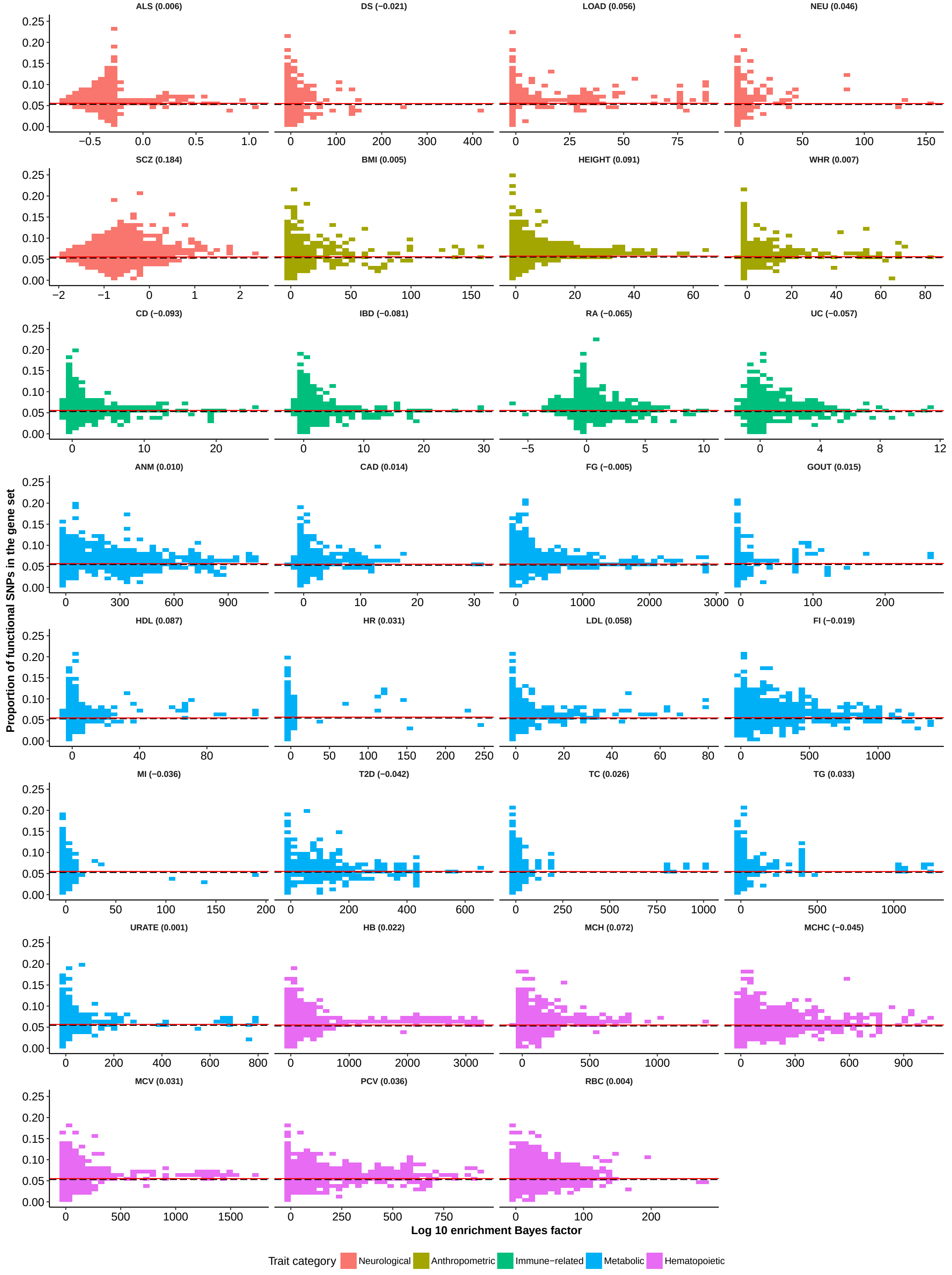

Functional category: Conserved_LindbladToh.extend.500

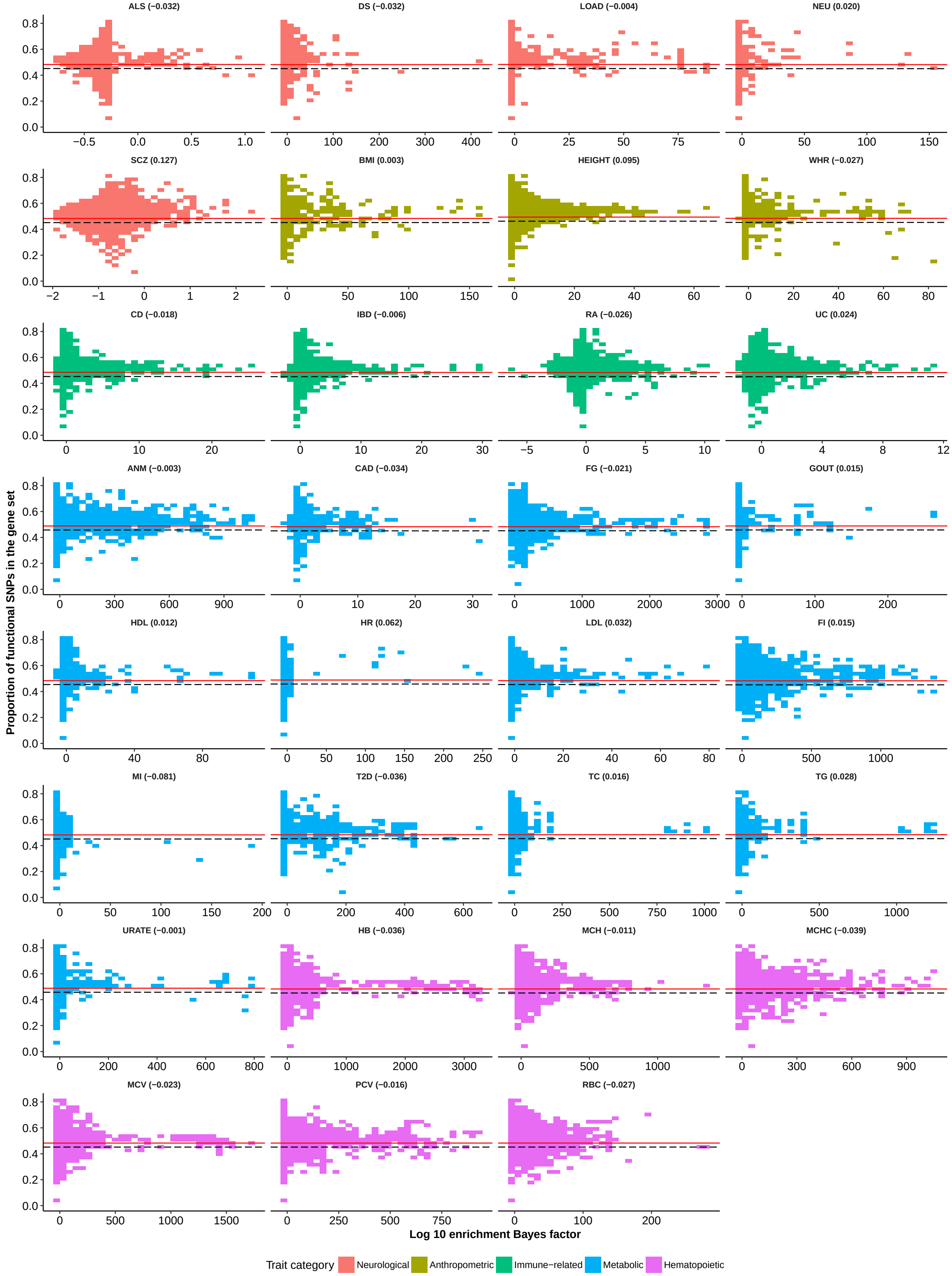

Functional category: CTCF_Hoffman

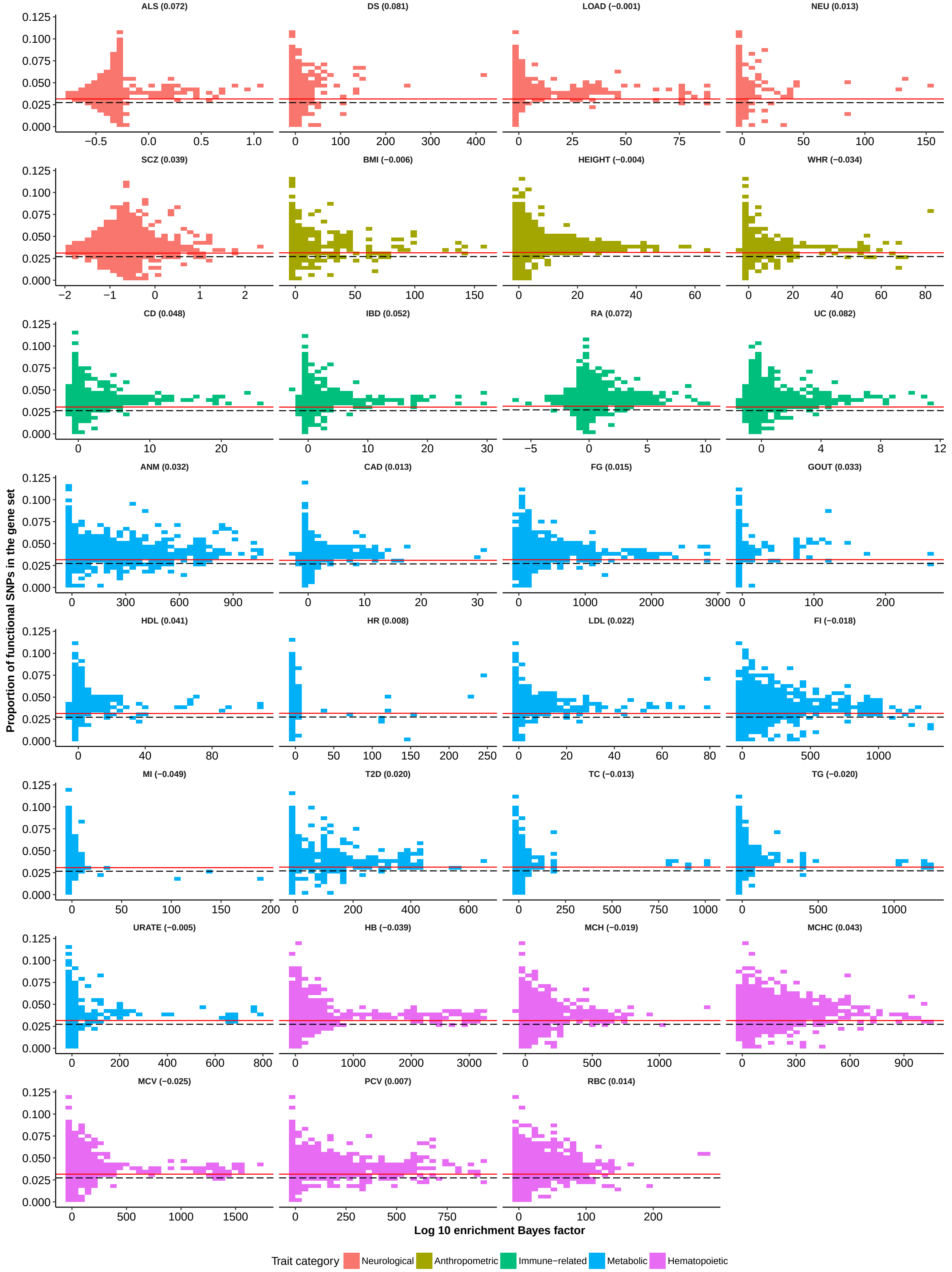

Functional category: CTCF_Hoffman.extend.500

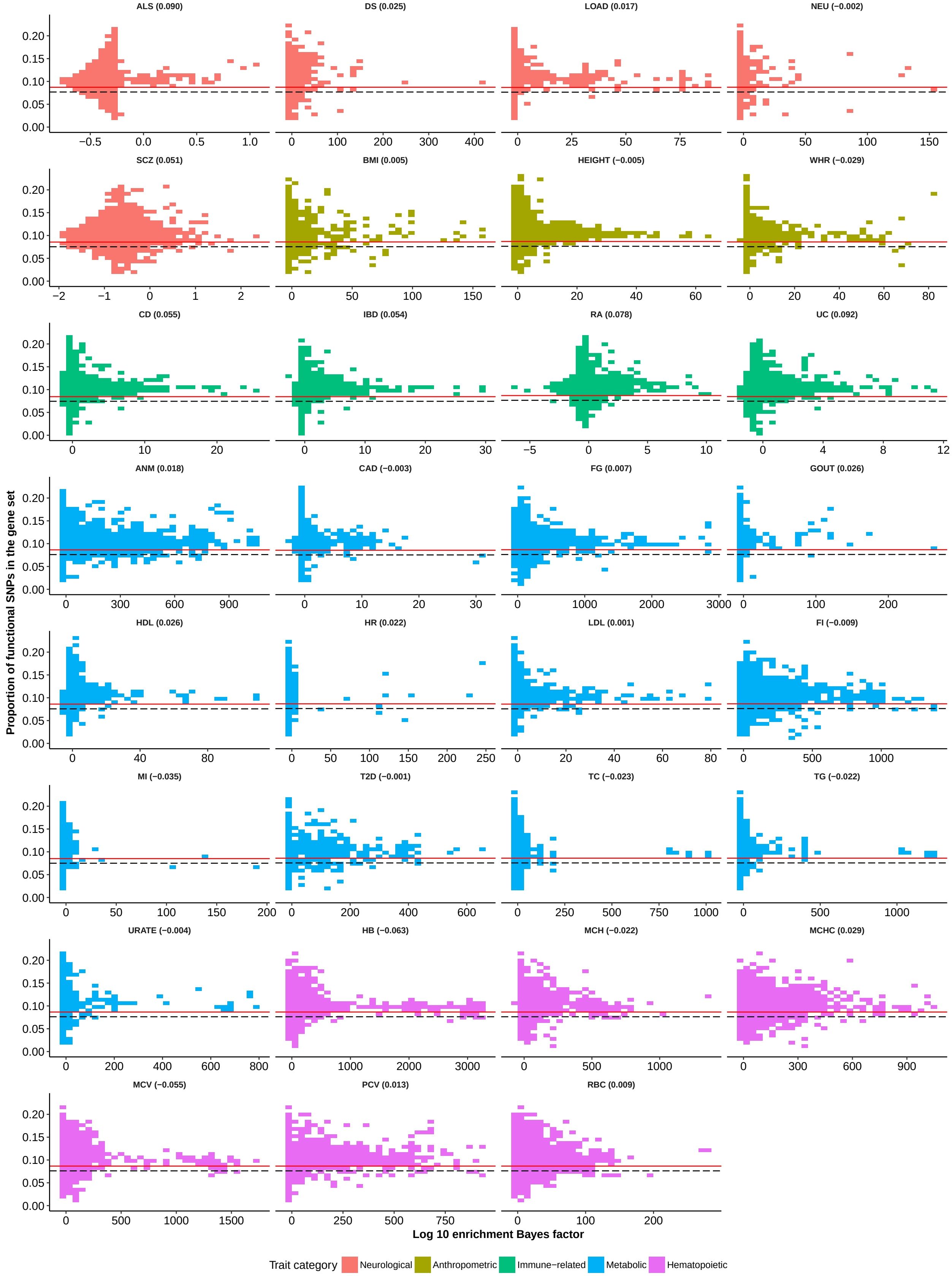

Functional category: DGF_ENCODE

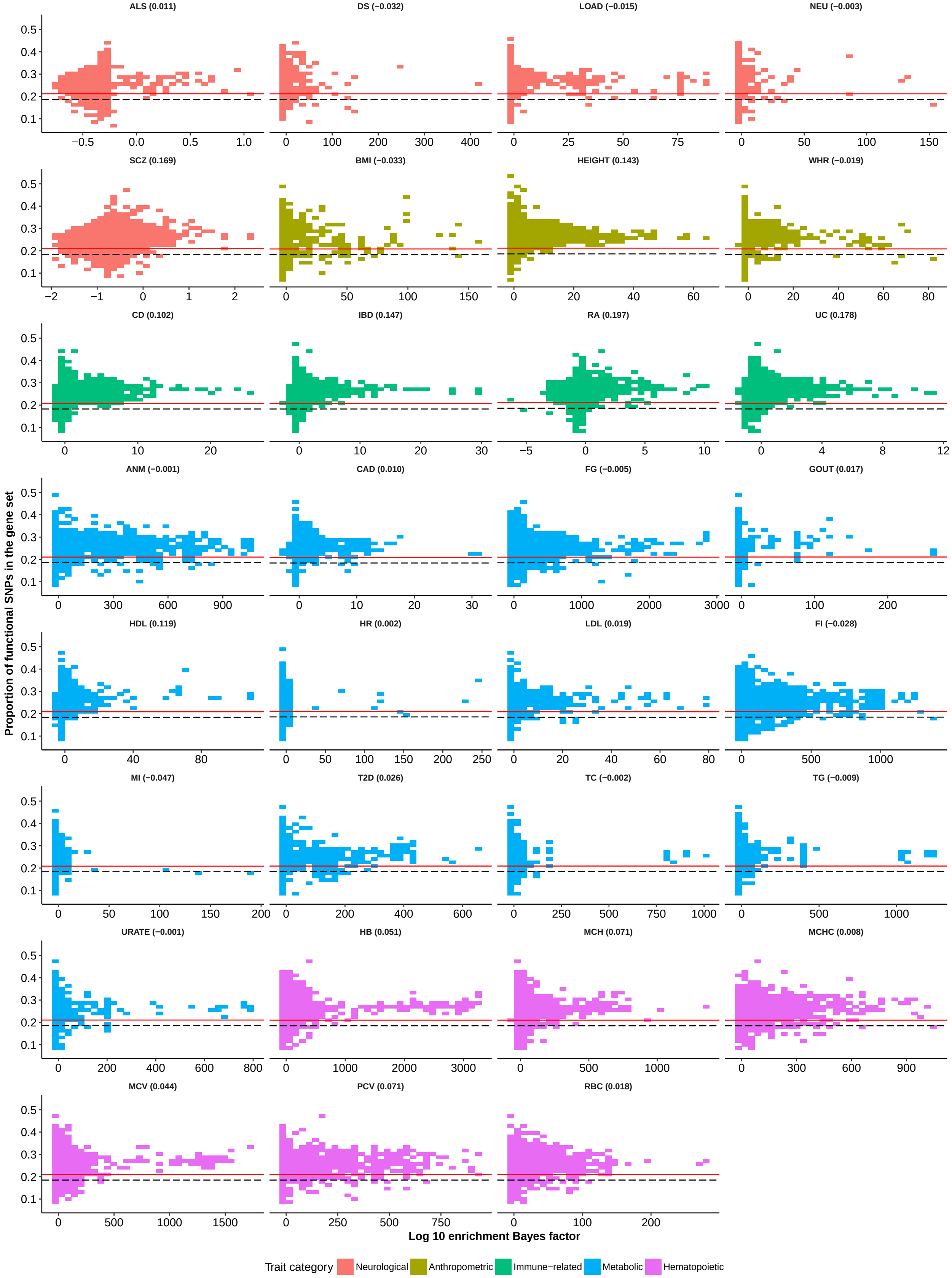

Functional category: DGF_ENCODE.extend.500

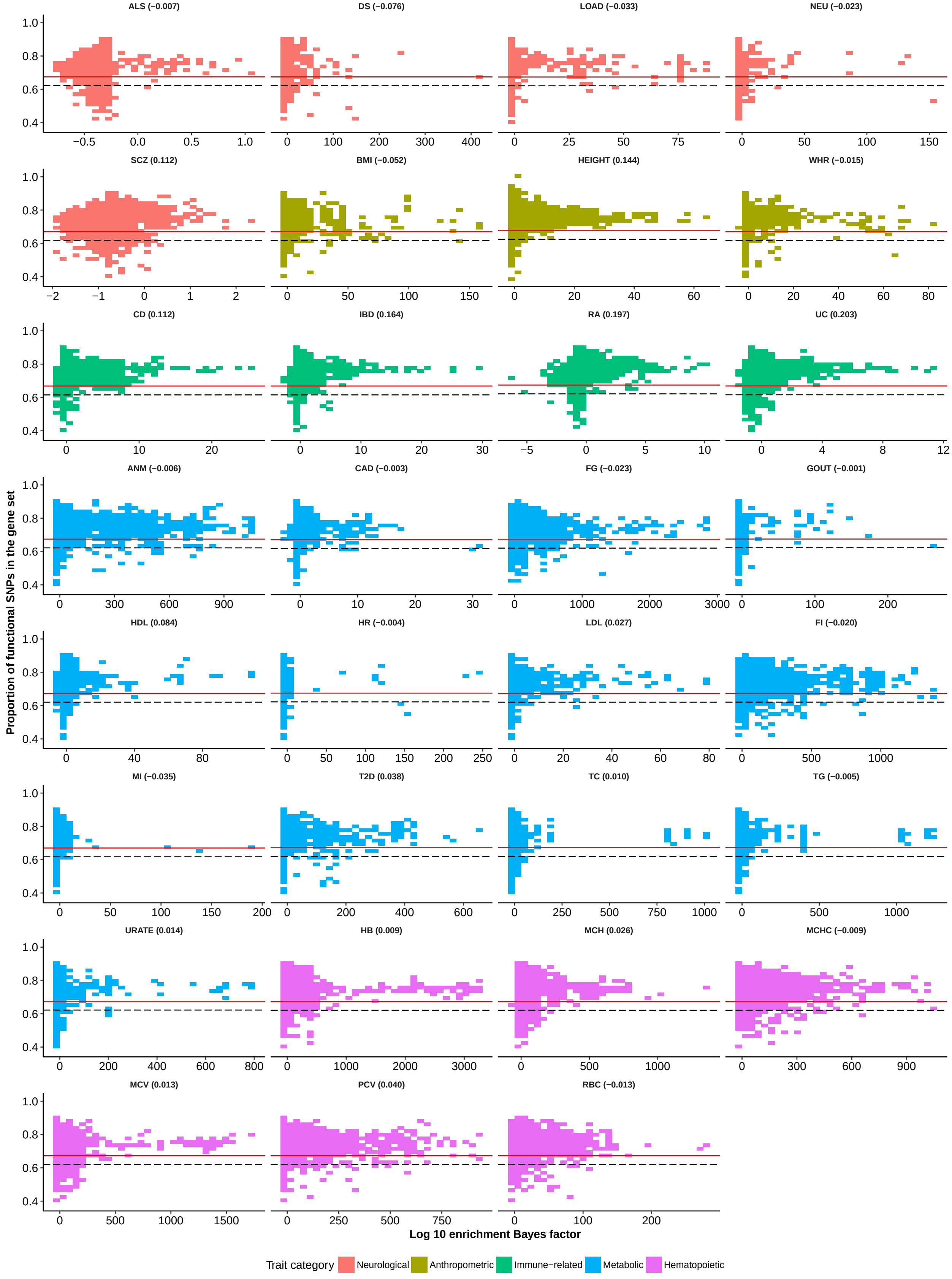

Functional category: DHS_peaks_Trynka

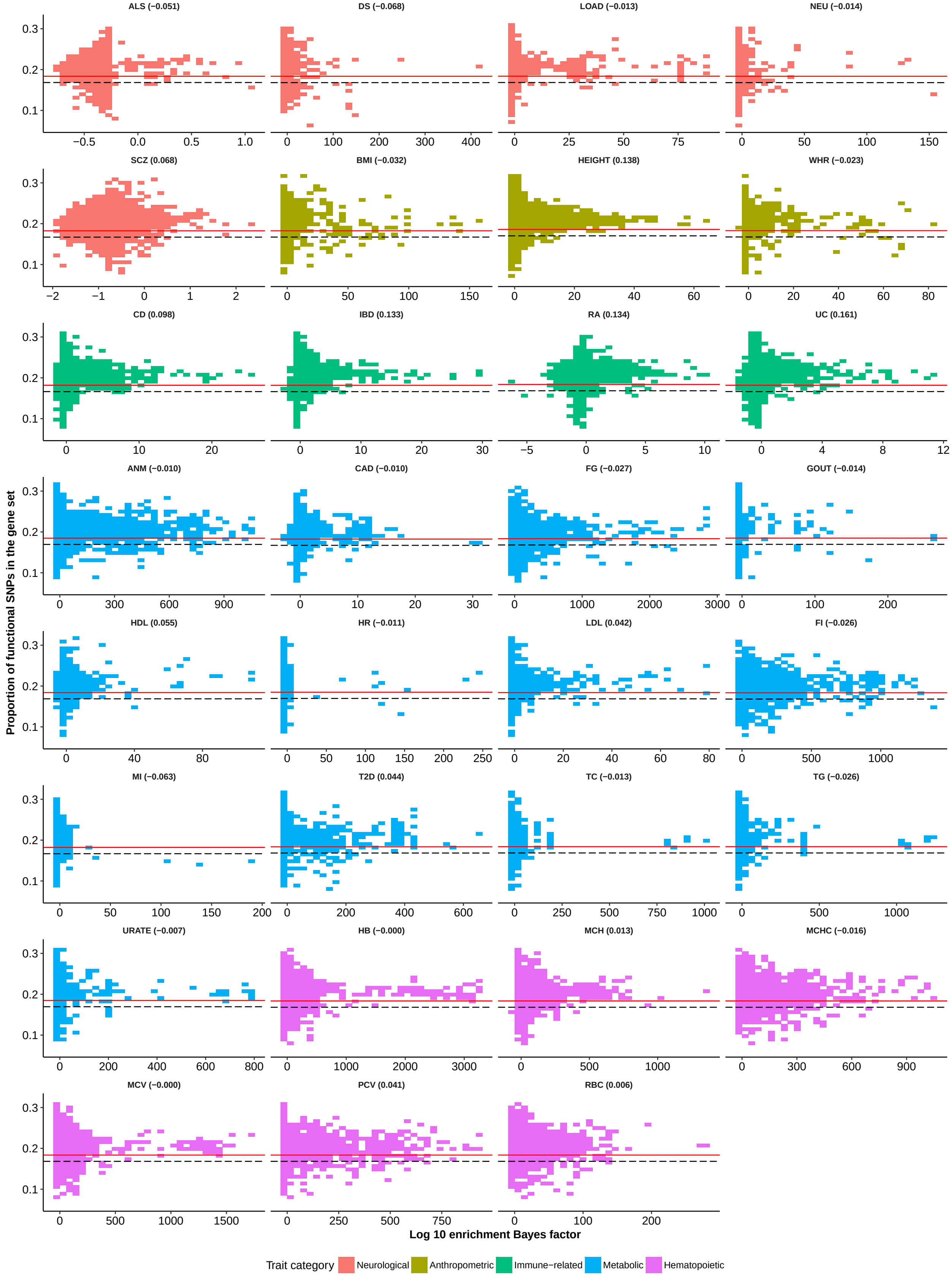

Functional category: DHS_Trynka

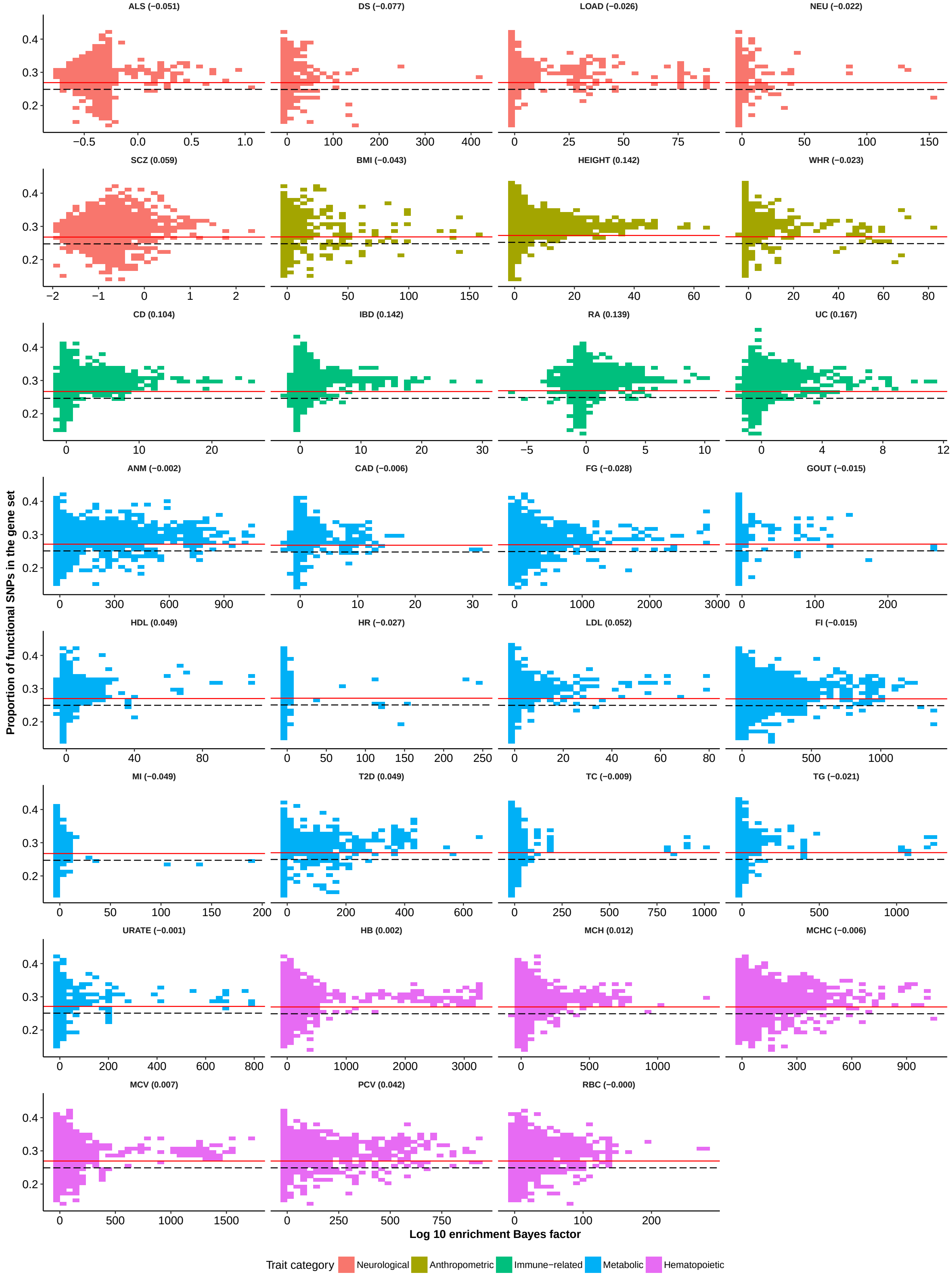

Functional category: DHS_Trynka.extend.500

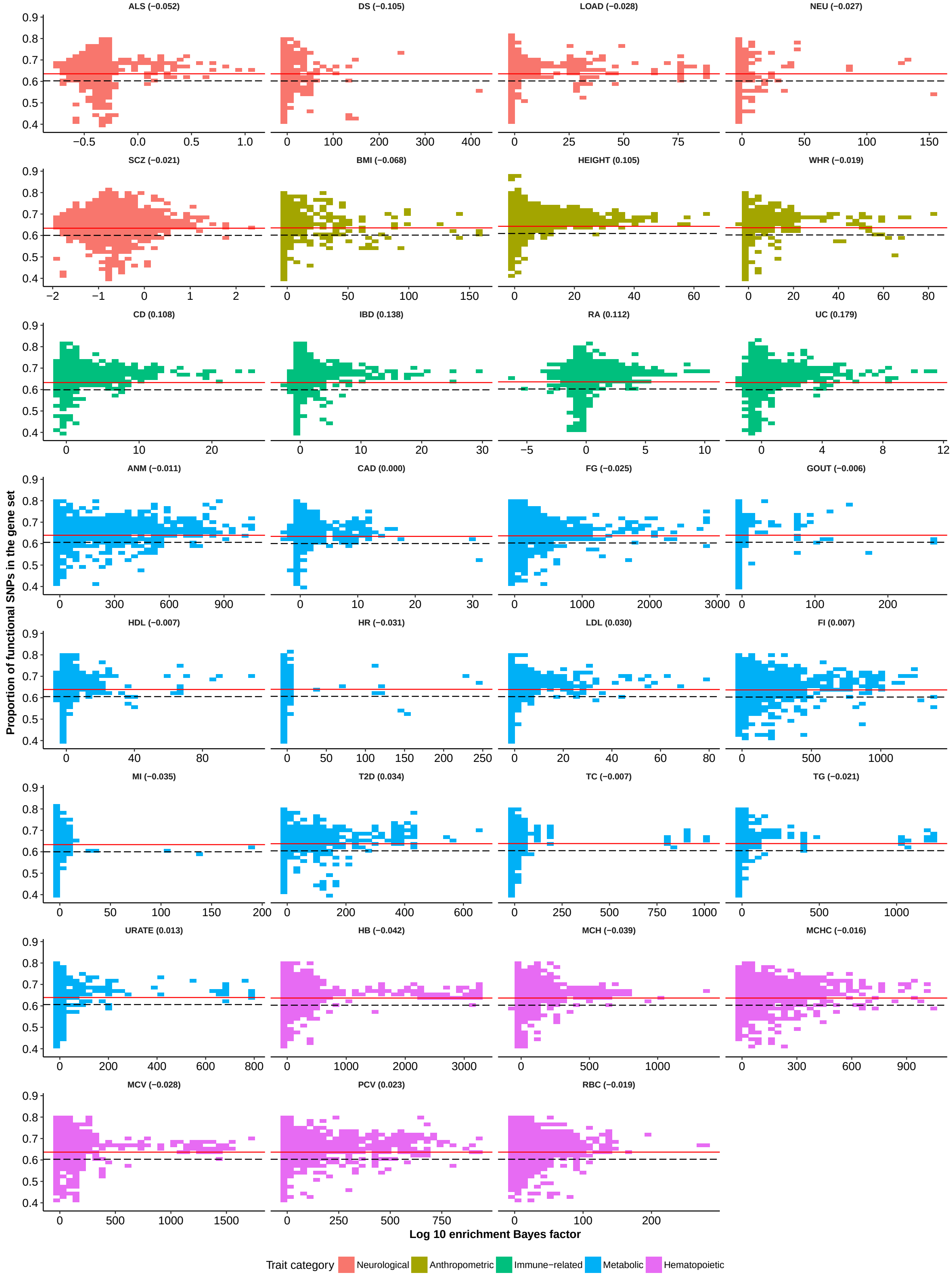

Functional category: Enhancer_Andersson

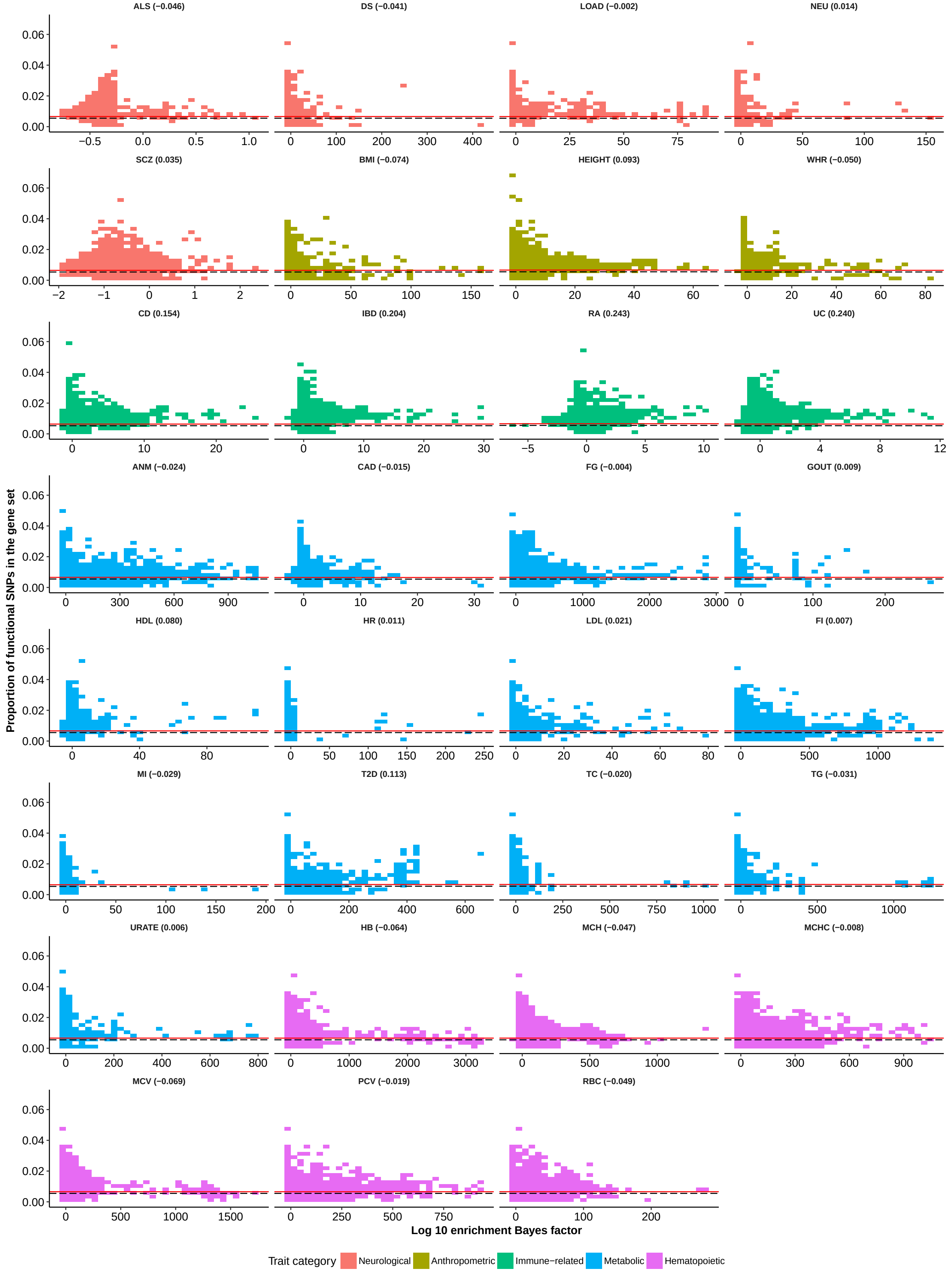

Functional category: Enhancer_Andersson.extend.500

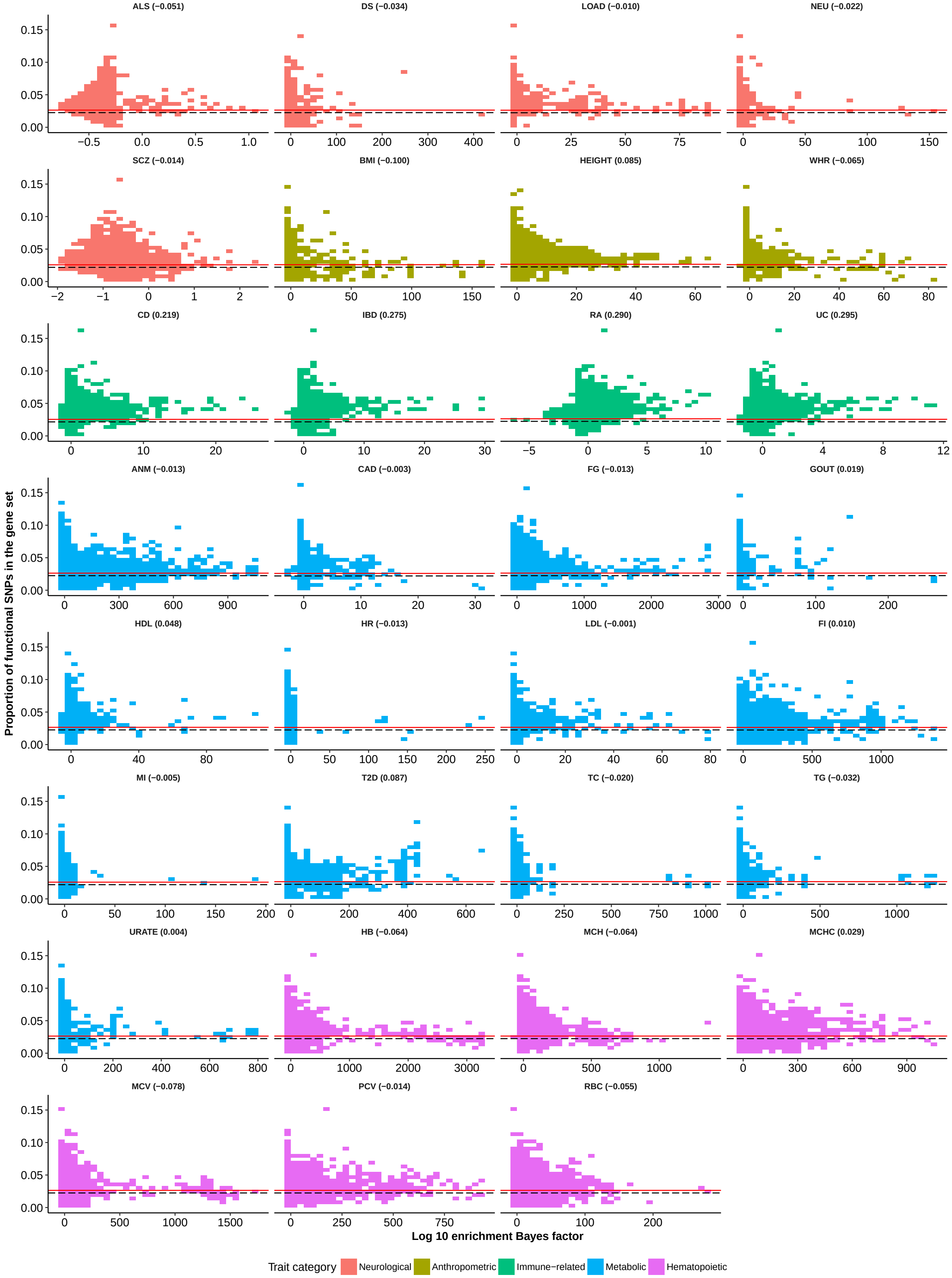

Functional category: Enhancer_Hoffman

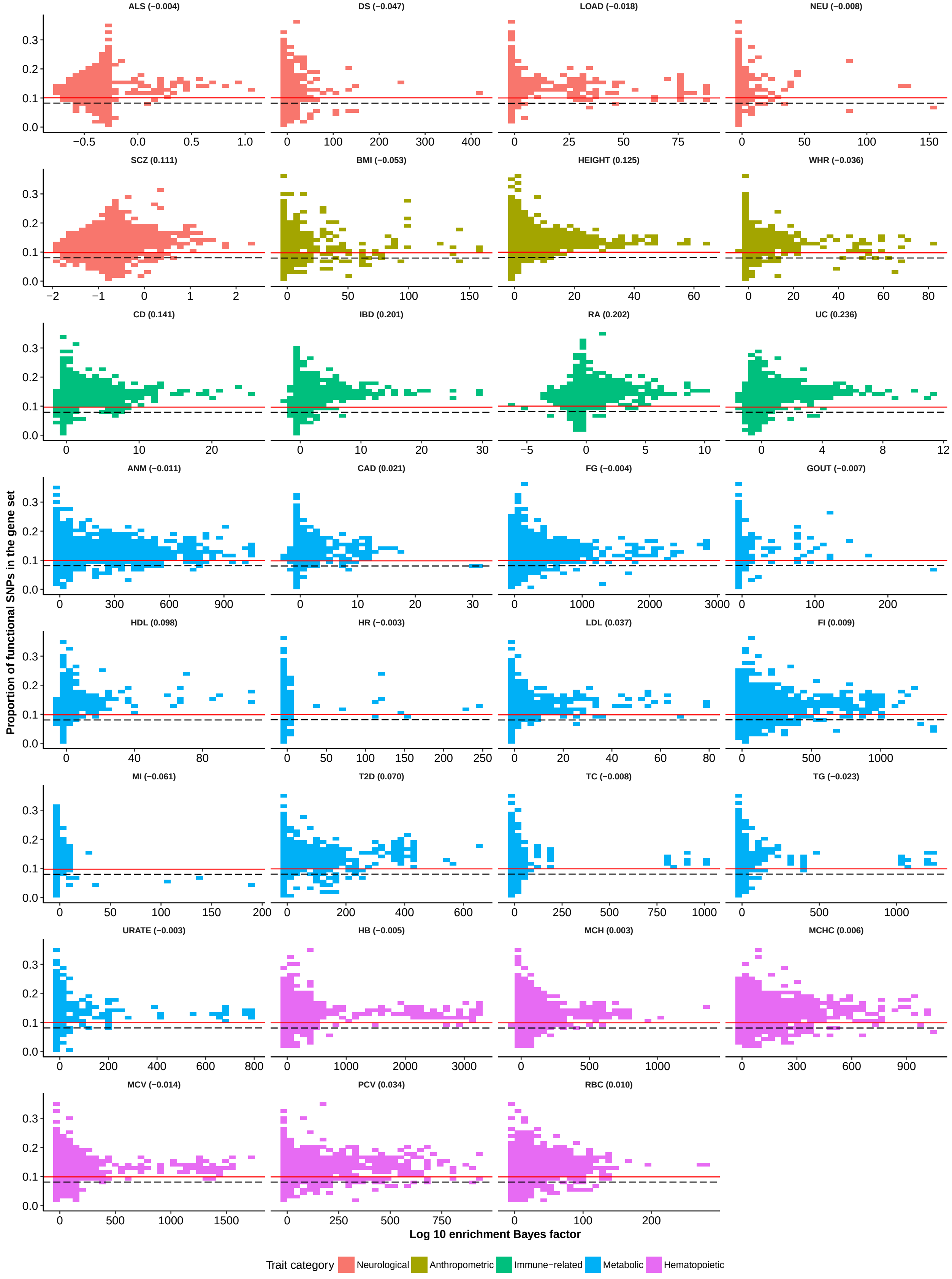

Functional category: Enhancer_Hoffman.extend.500

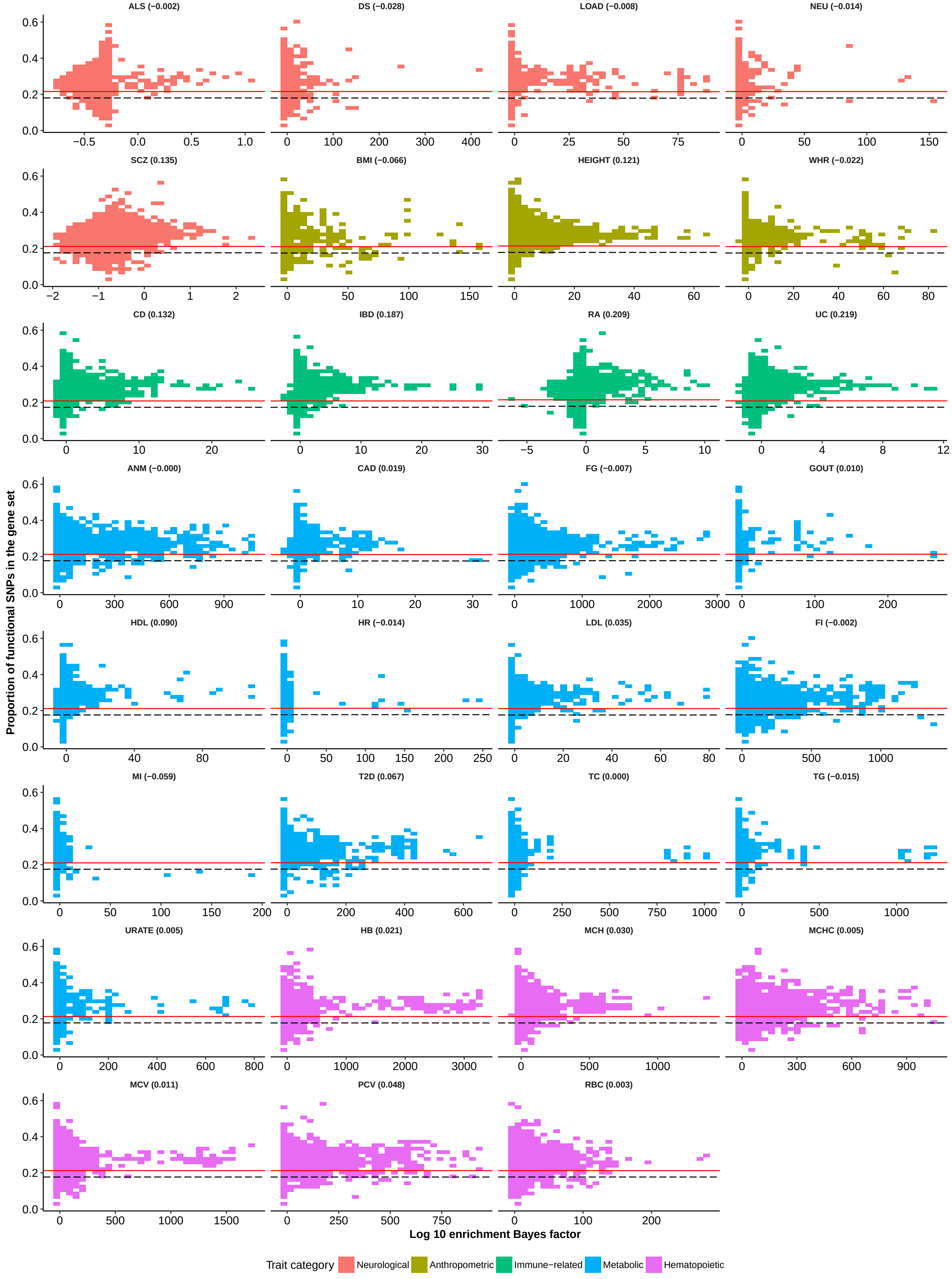

Functional category: FetalDHS_Trynka

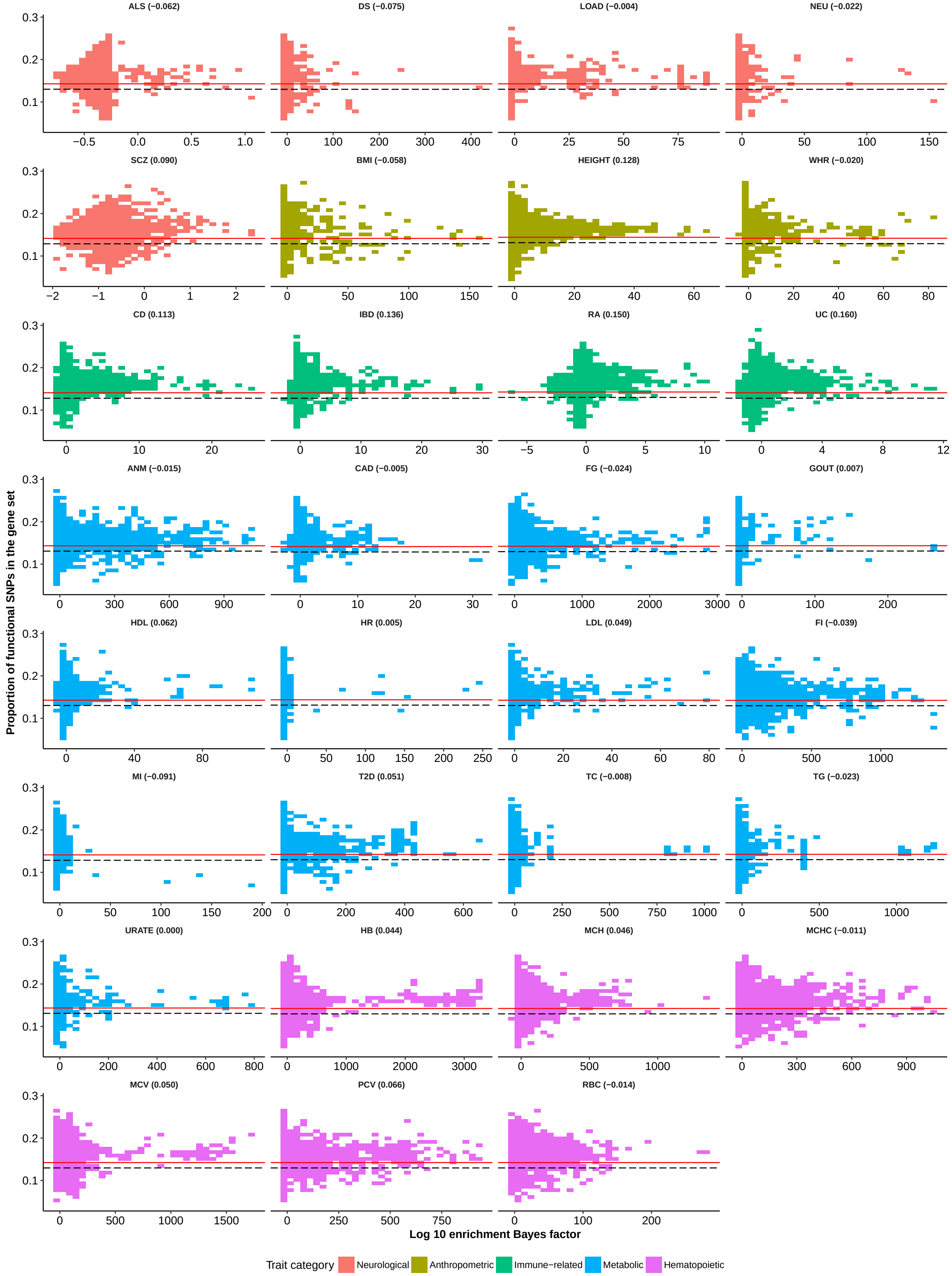

Functional category: FetalDHS_Trynka.extend.500

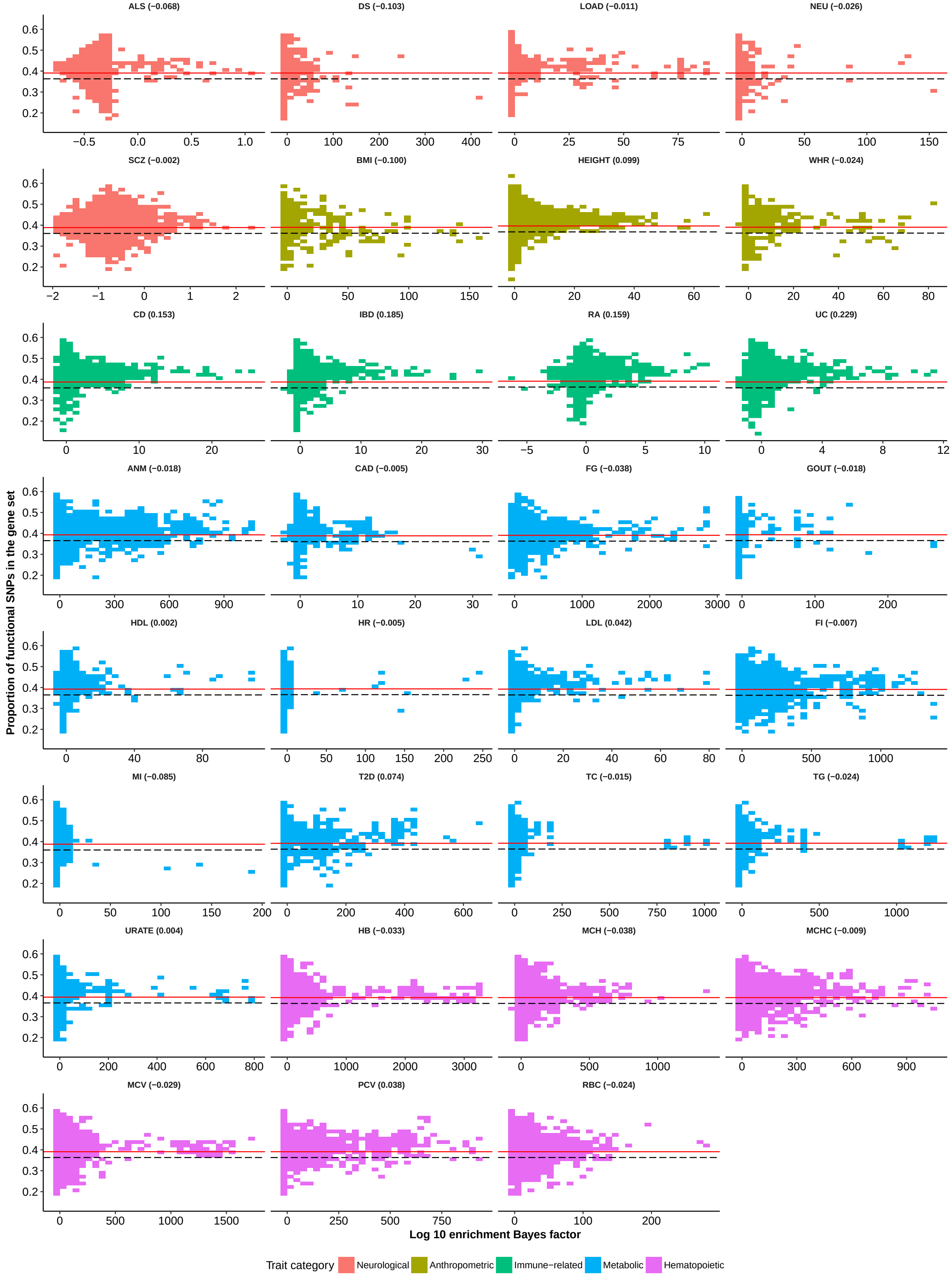

Functional category: H3K27ac_Hnisz

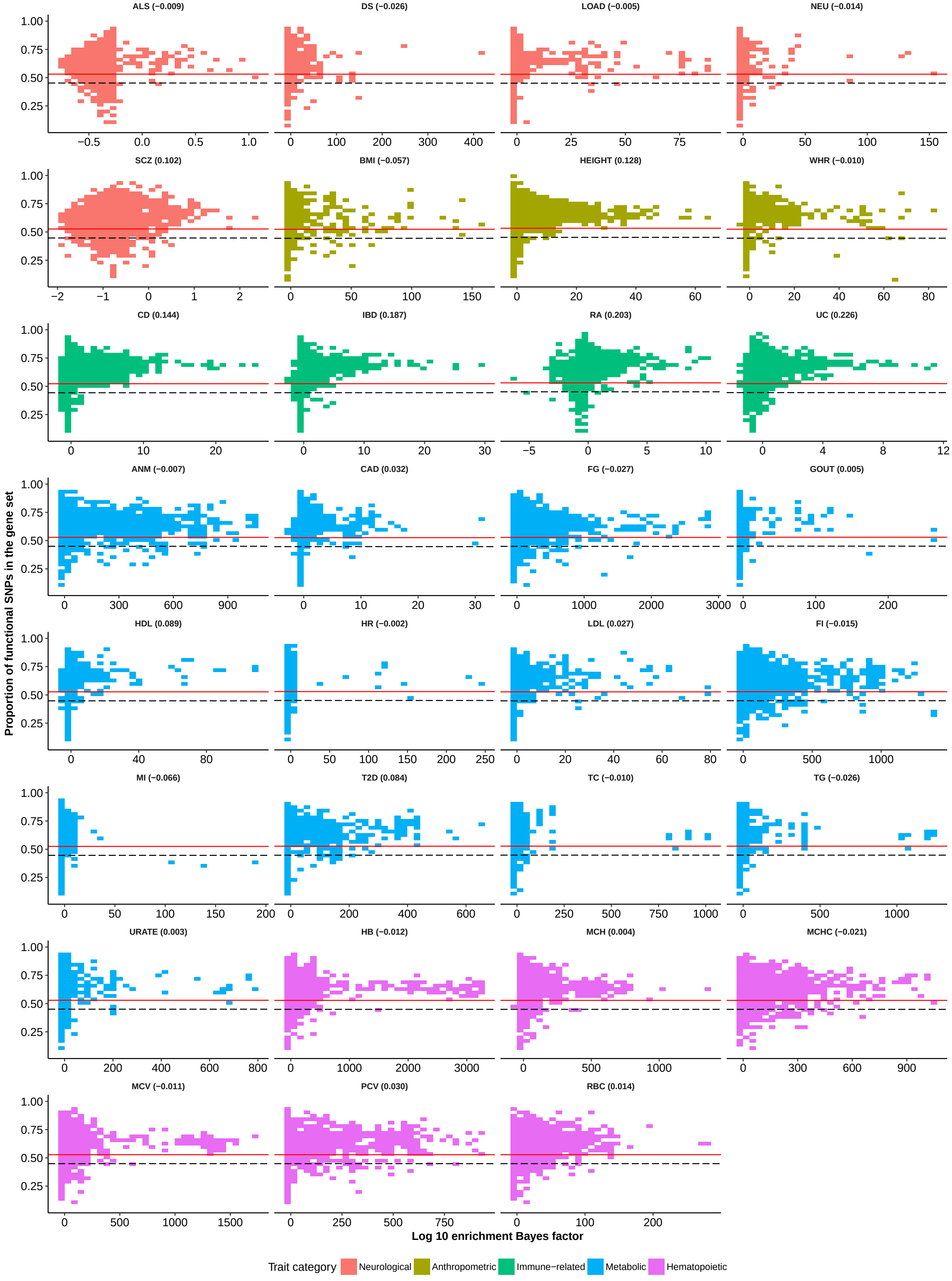

Functional category: H3K27ac_Hnisz.extend.500

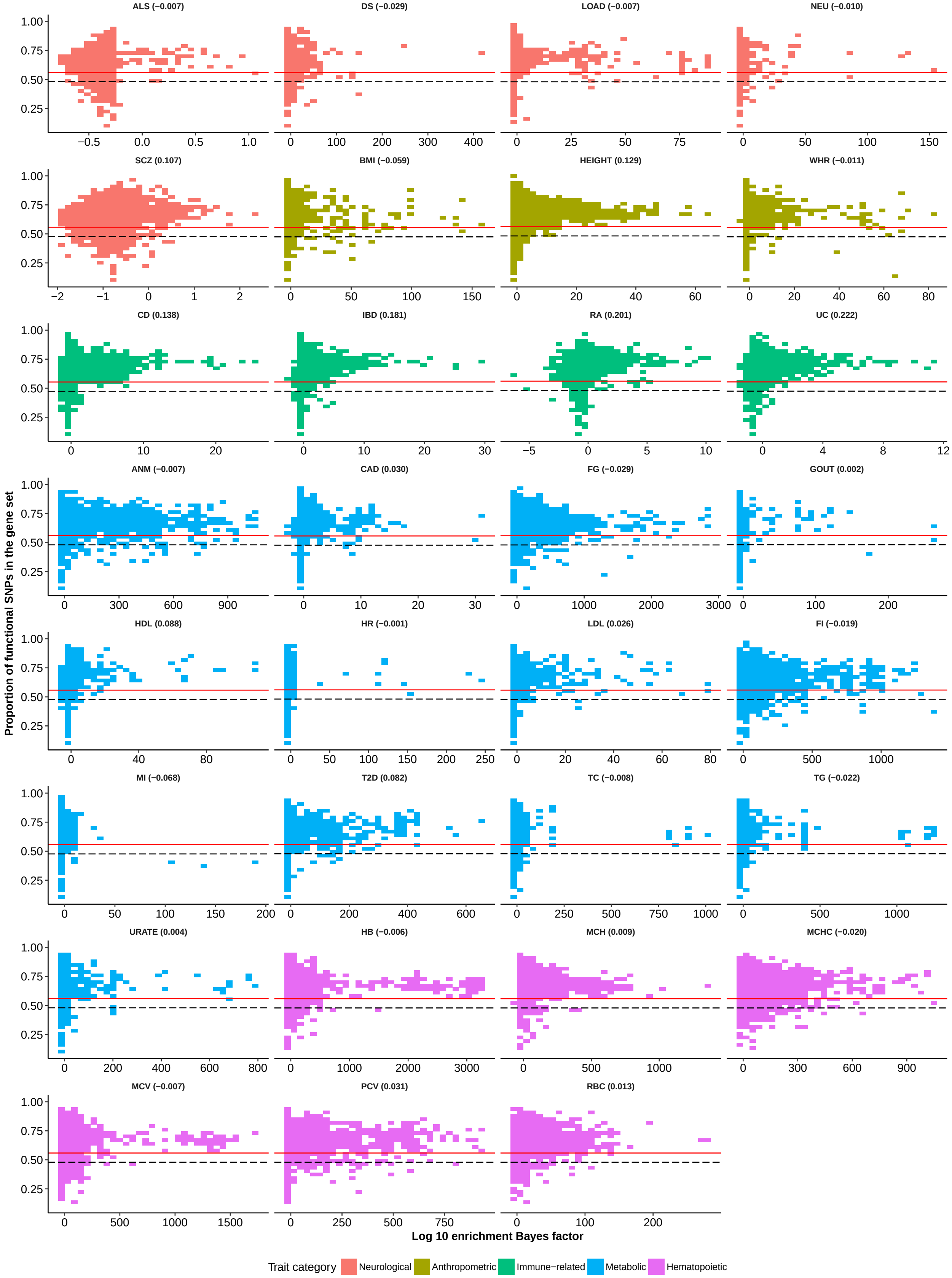

Functional category: H3K27ac_PGC2

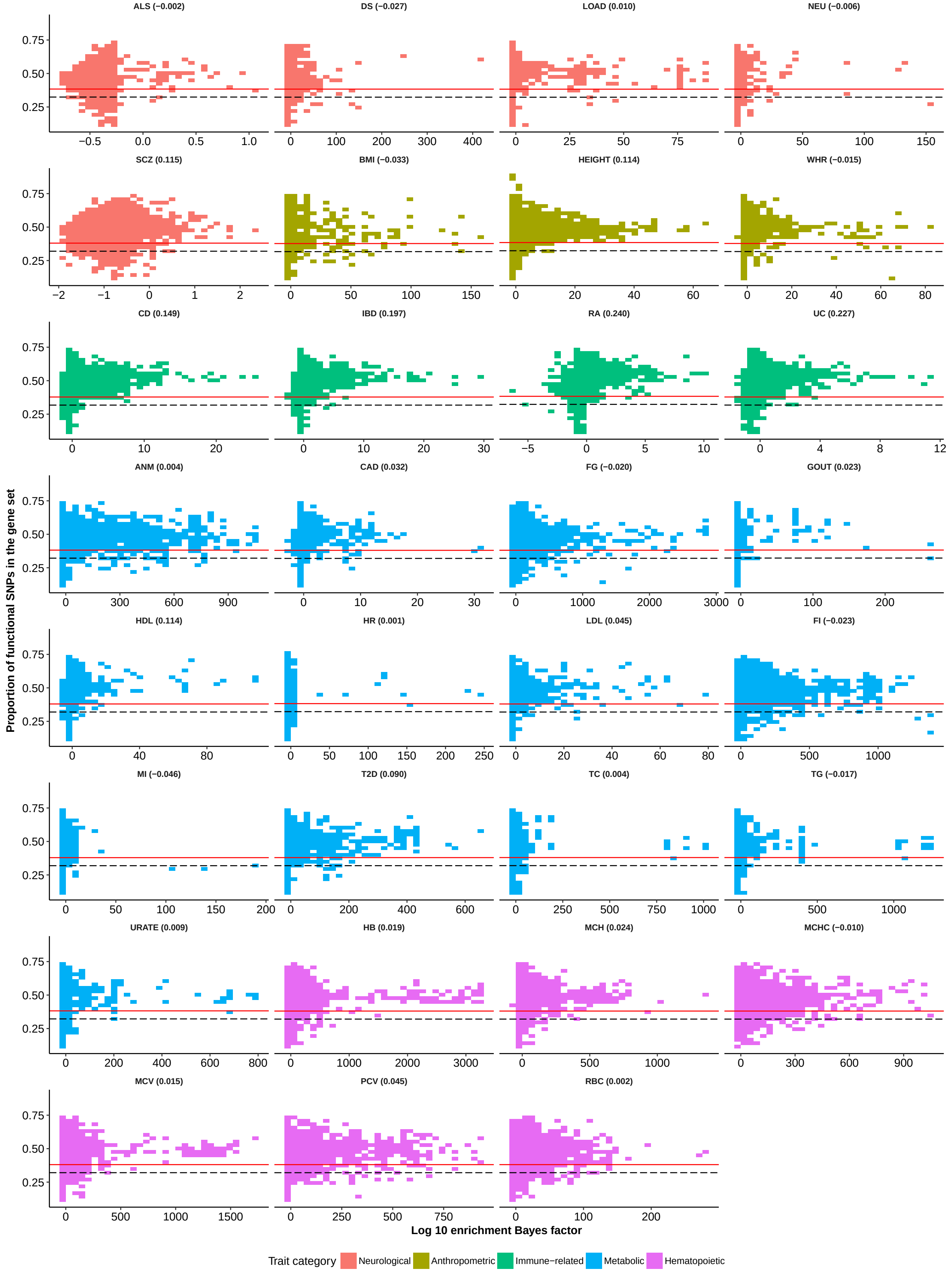

Functional category: H3K27ac_PGC2.extend.500

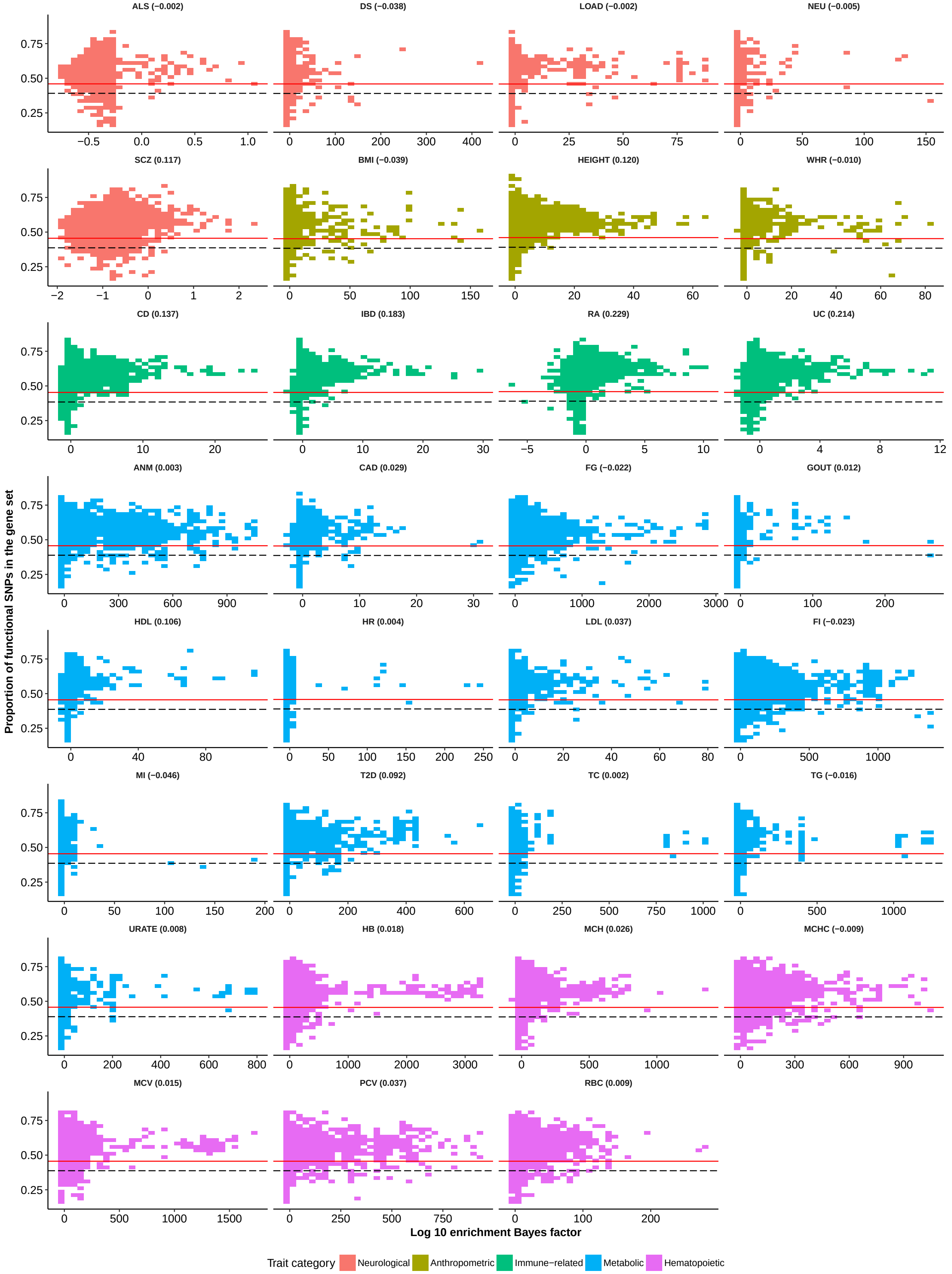

Functional category: H3K4me1_peaks_Trynka

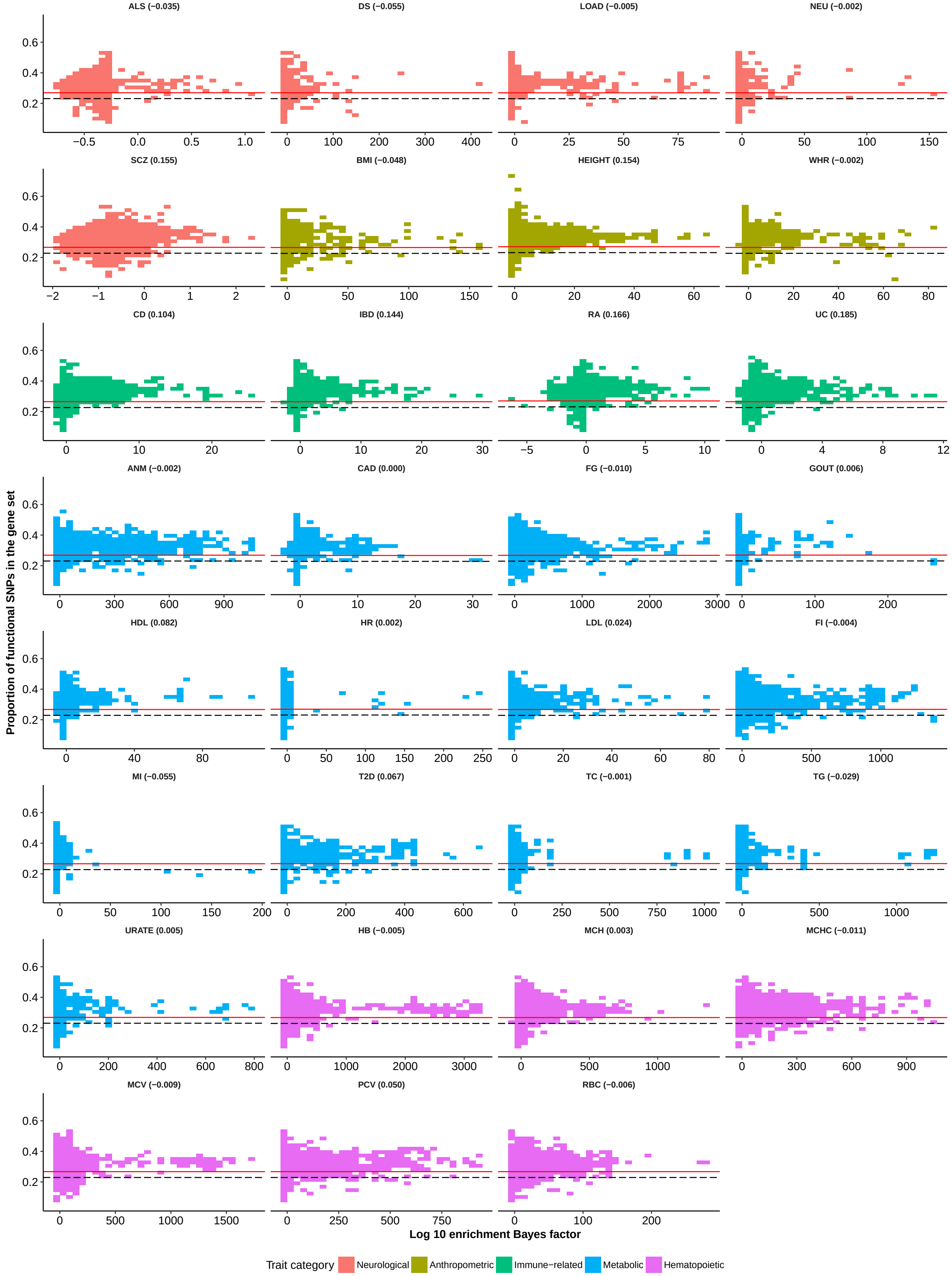

Functional category: H3K4me1_Trynka

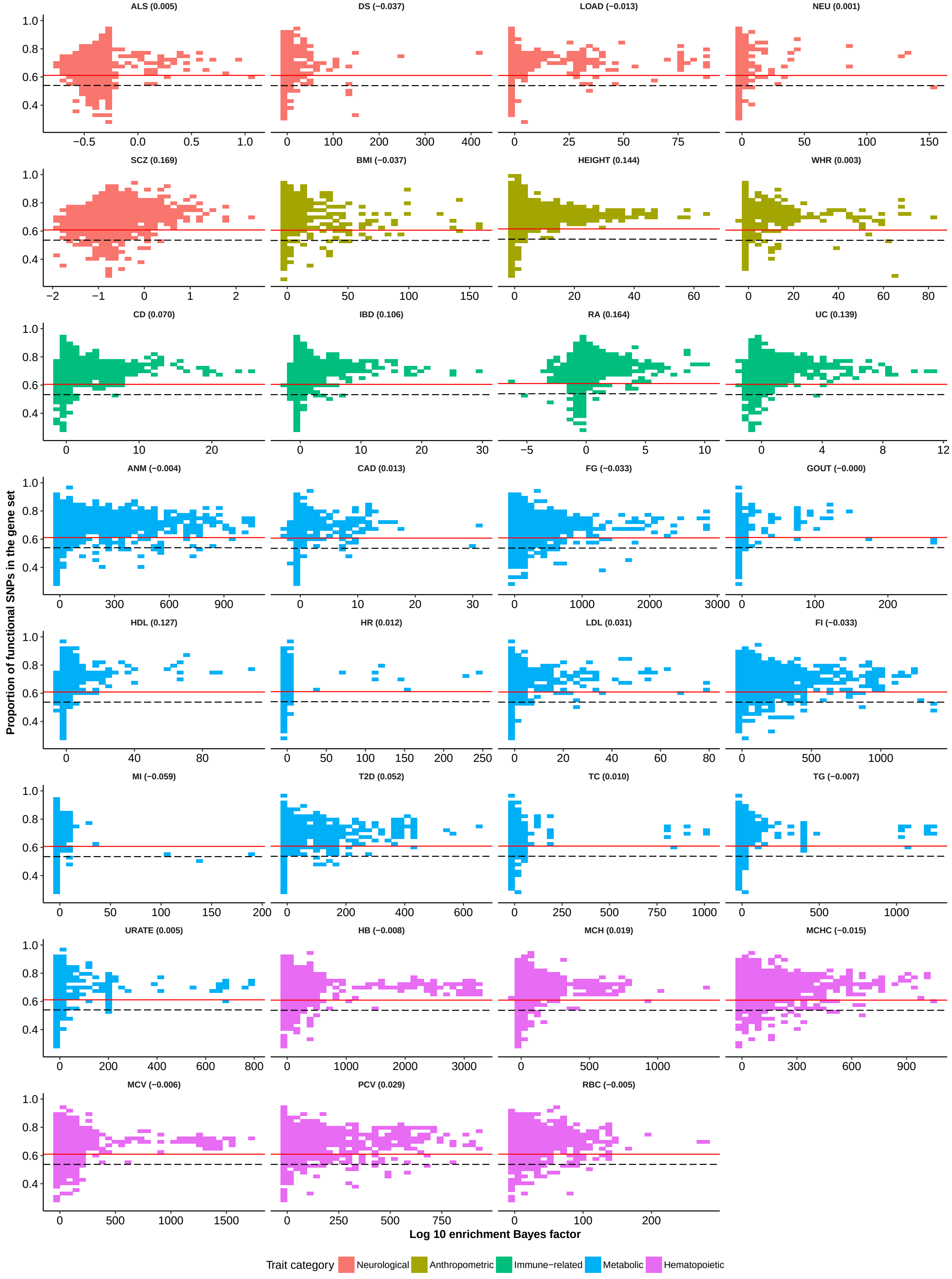

Functional category: H3K4me1_Trynka.extend.500

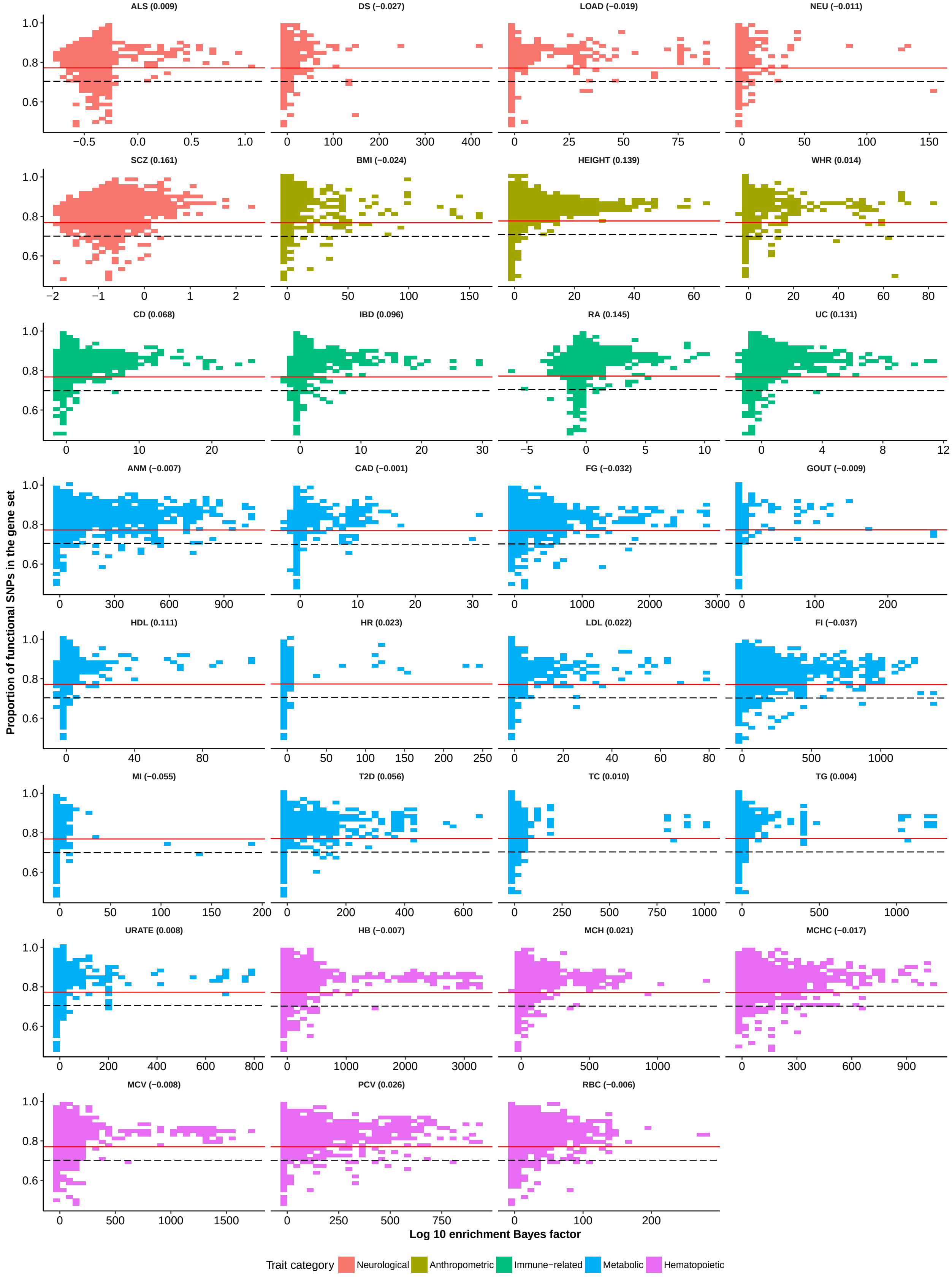

Functional category: H3K4me3_peaks_Trynka

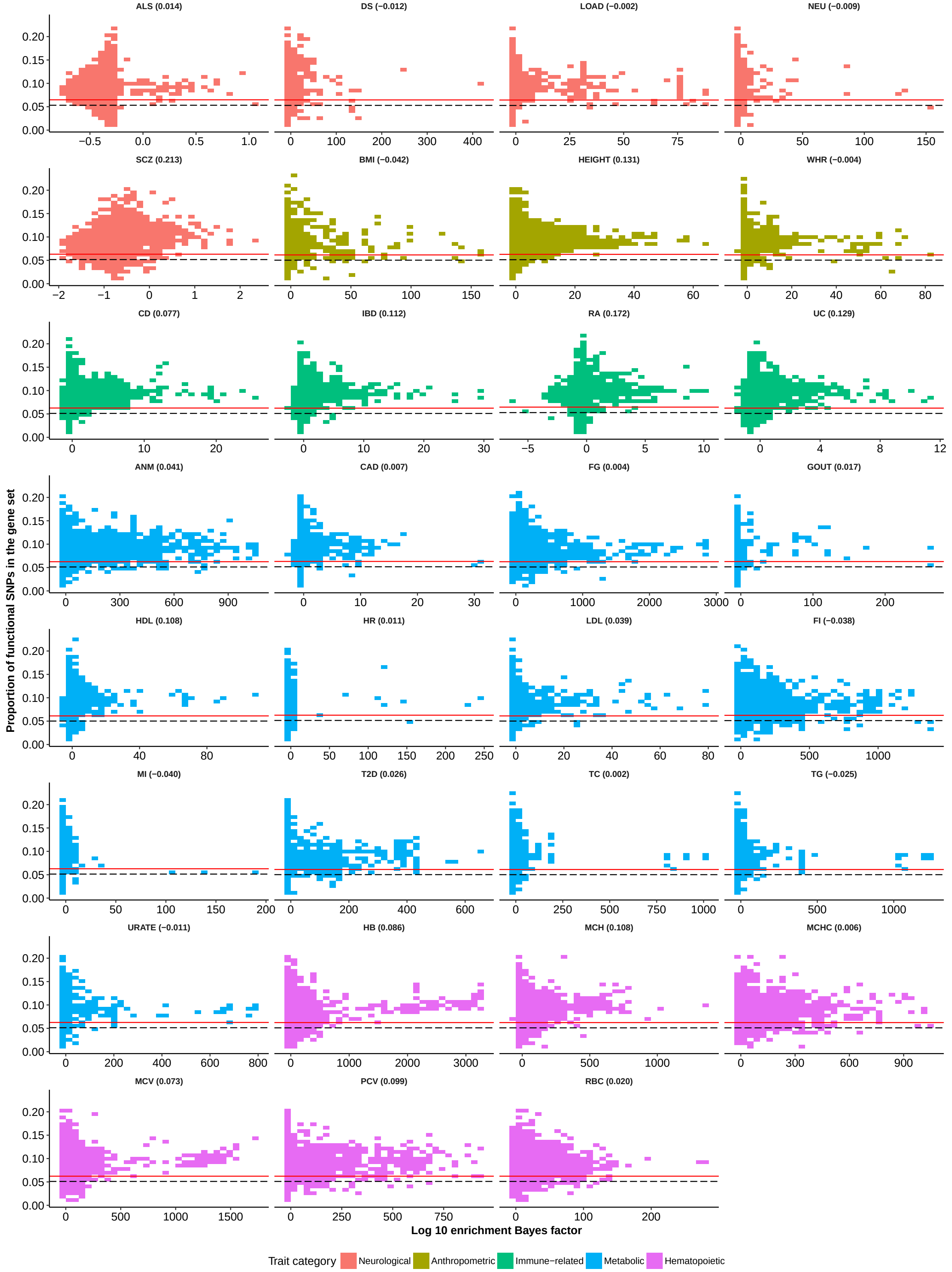

Functional category: H3K4me3_Trynka

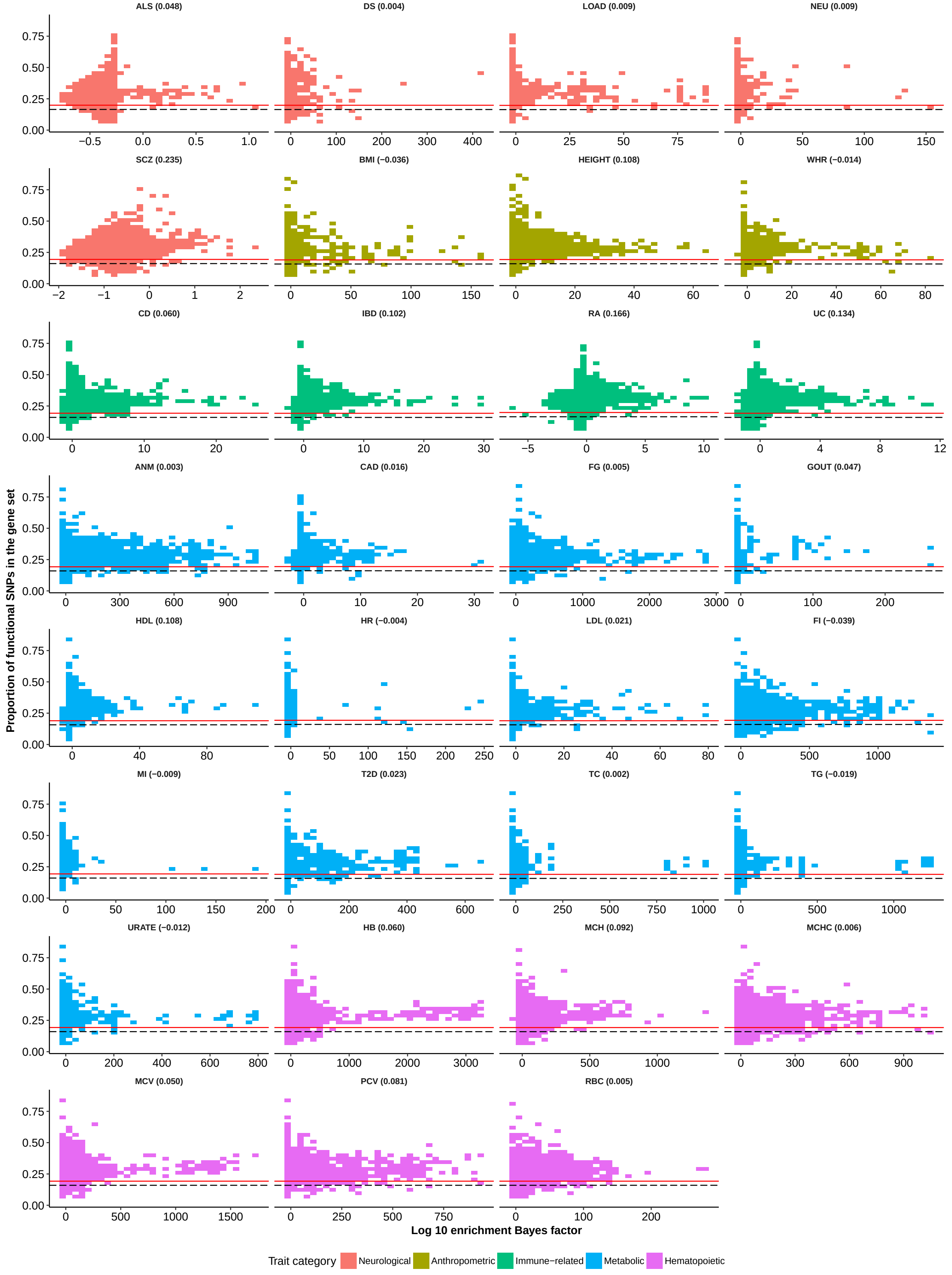

Functional category: H3K4me3_Trynka.extend.500

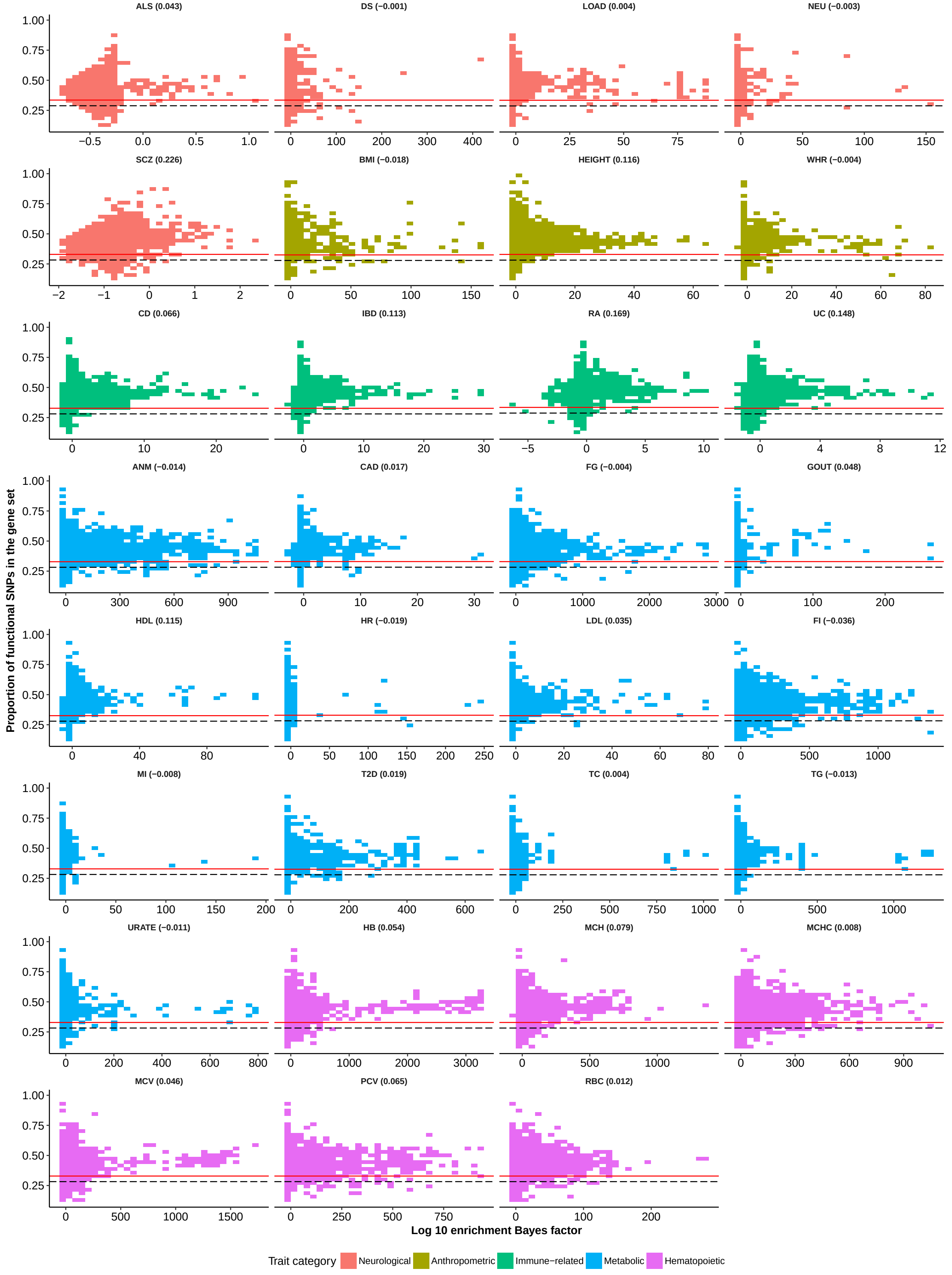

Functional category: H3K9ac_peaks_Trynka

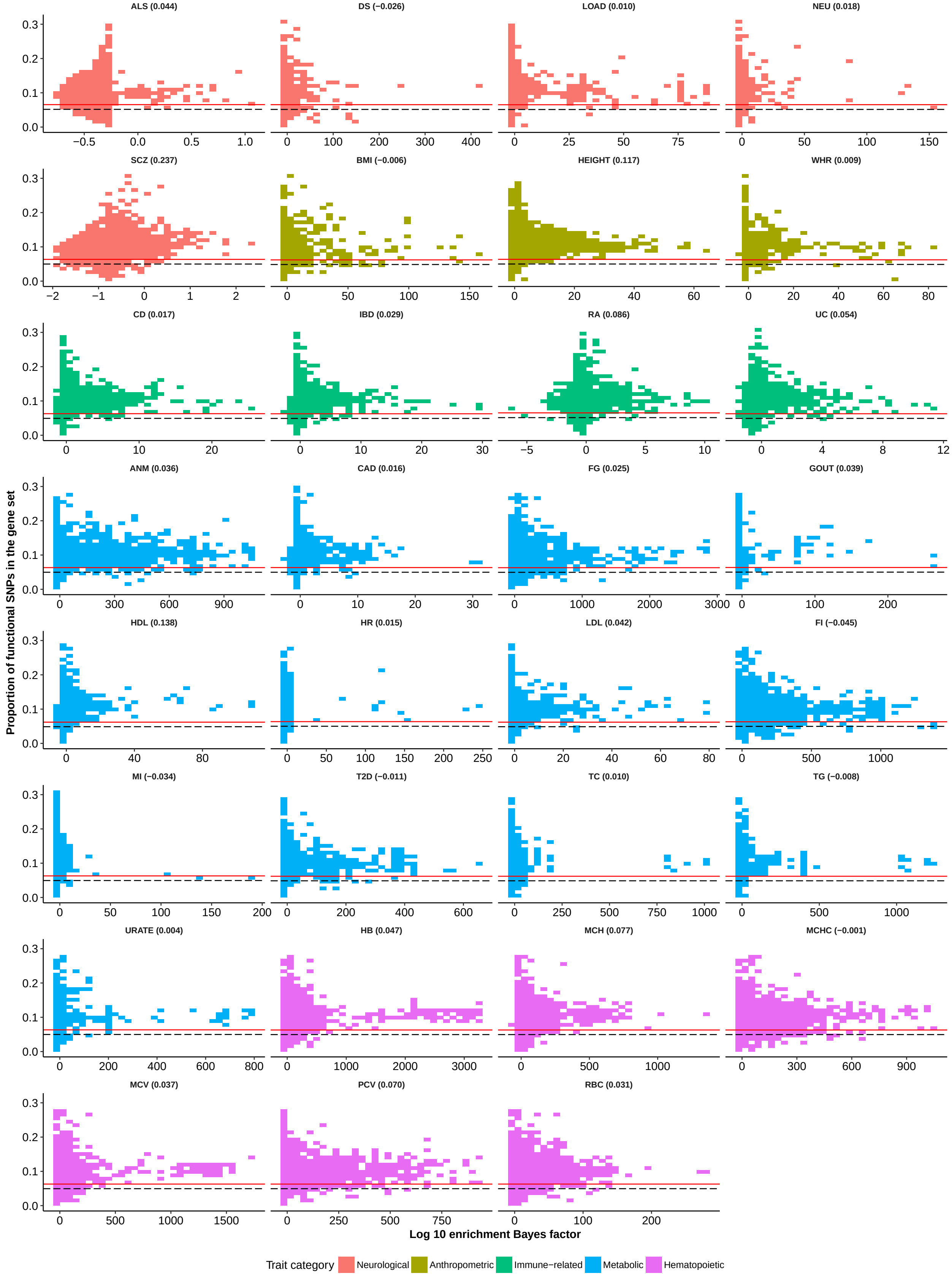

Functional category: H3K9ac_Trynka

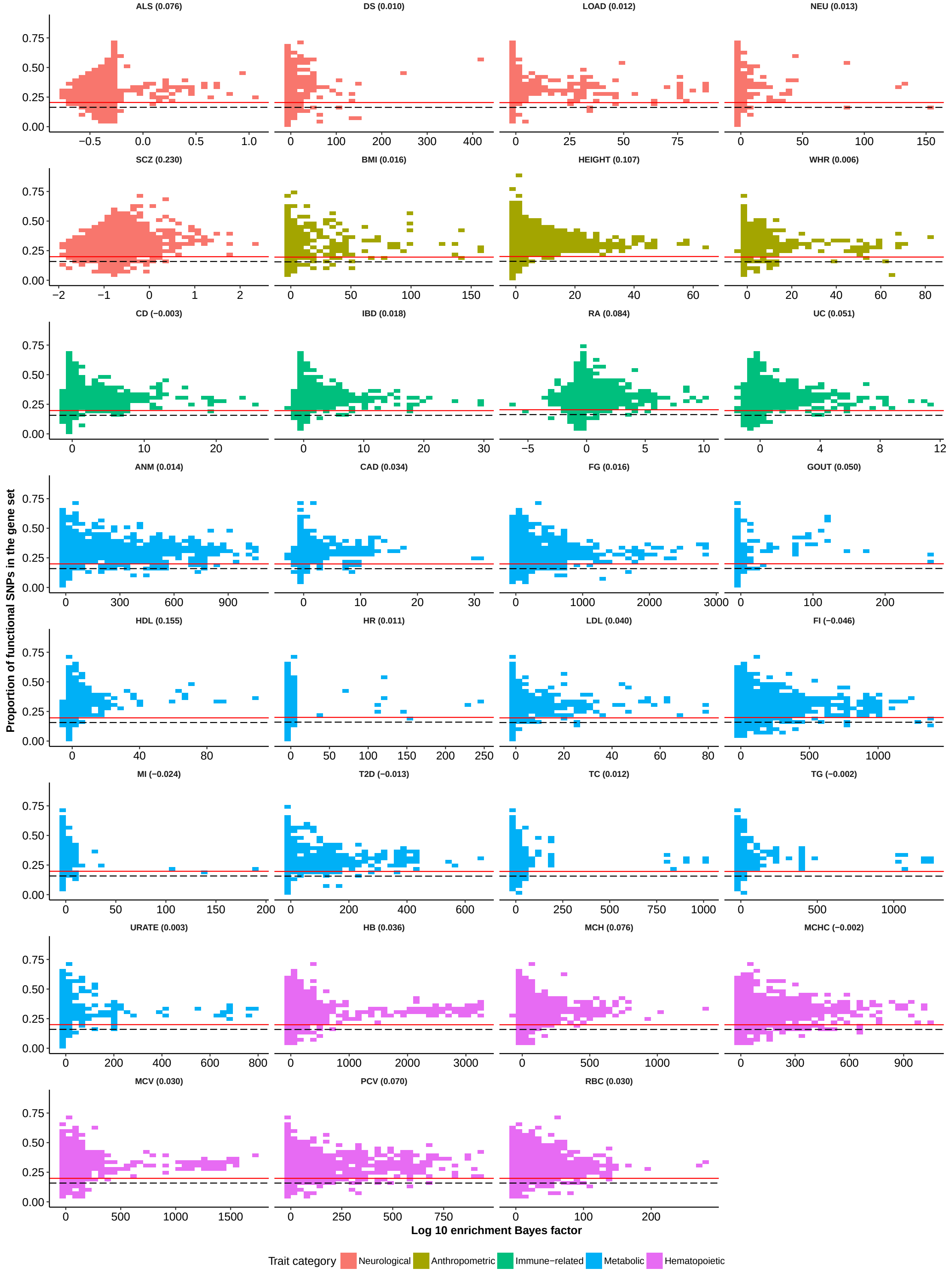

Functional category: H3K9ac_Trynka.extend.500

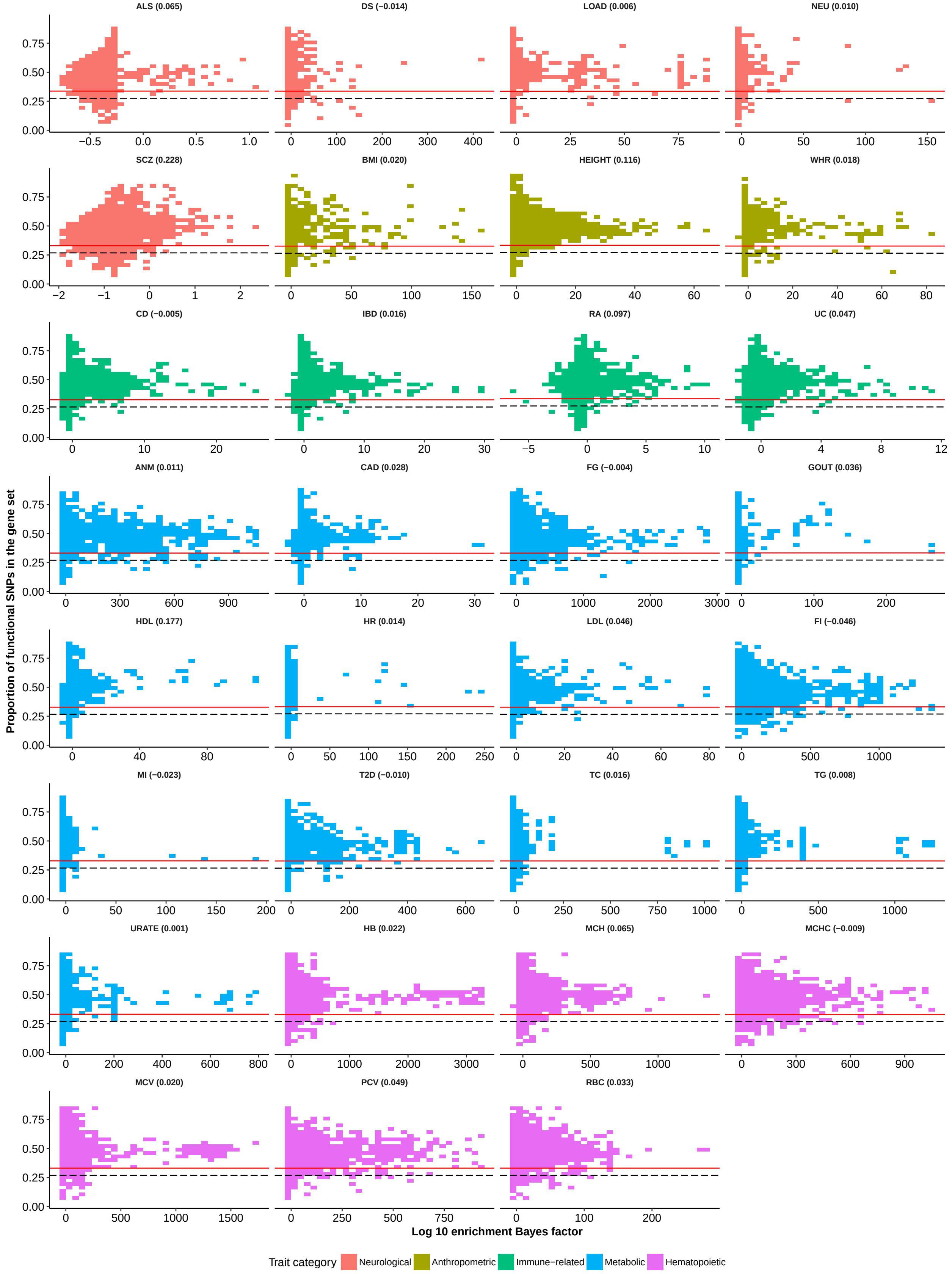

Functional category: Intron_UCSC

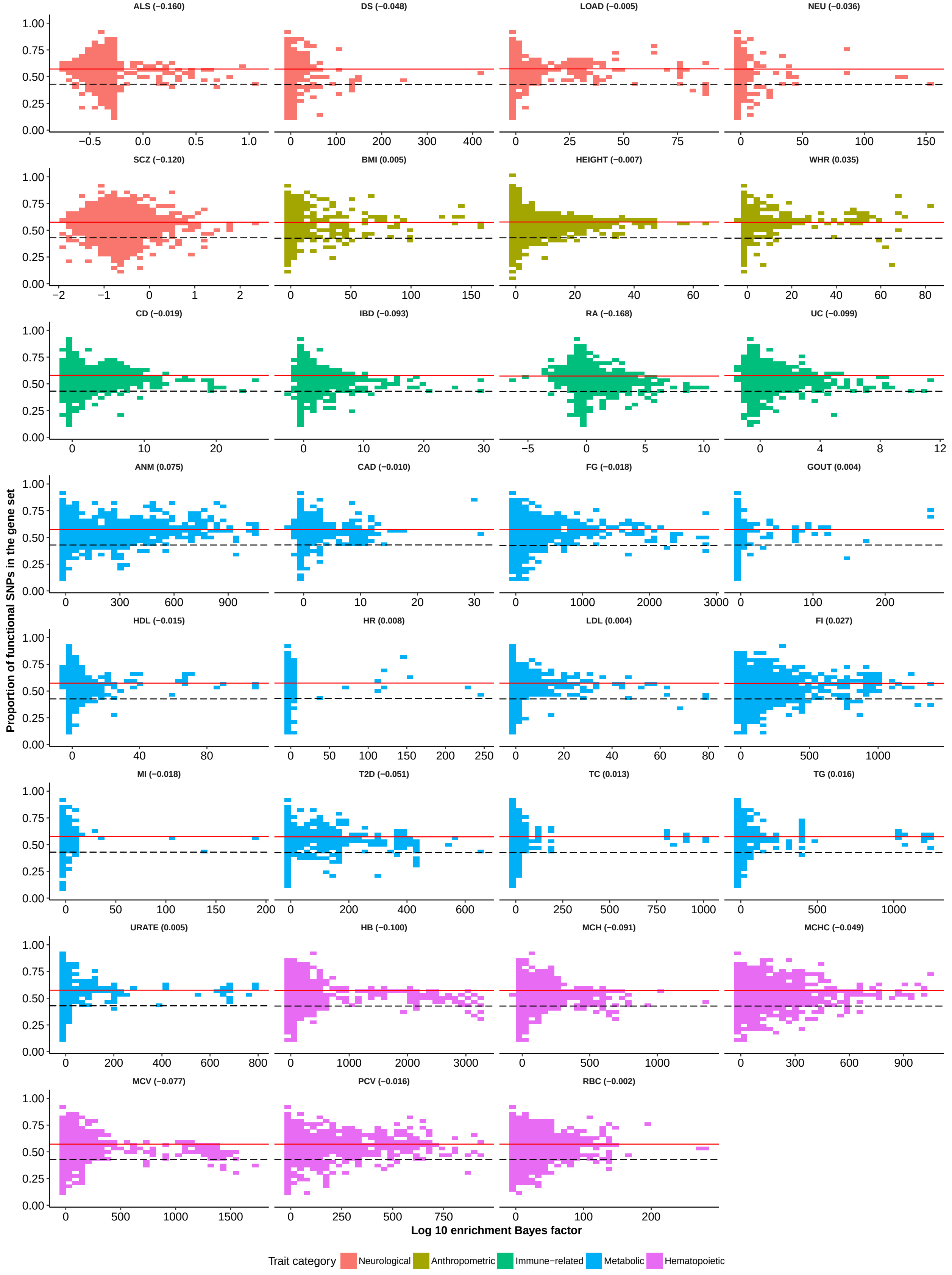

Functional category: Intron_UCSC.extend.500

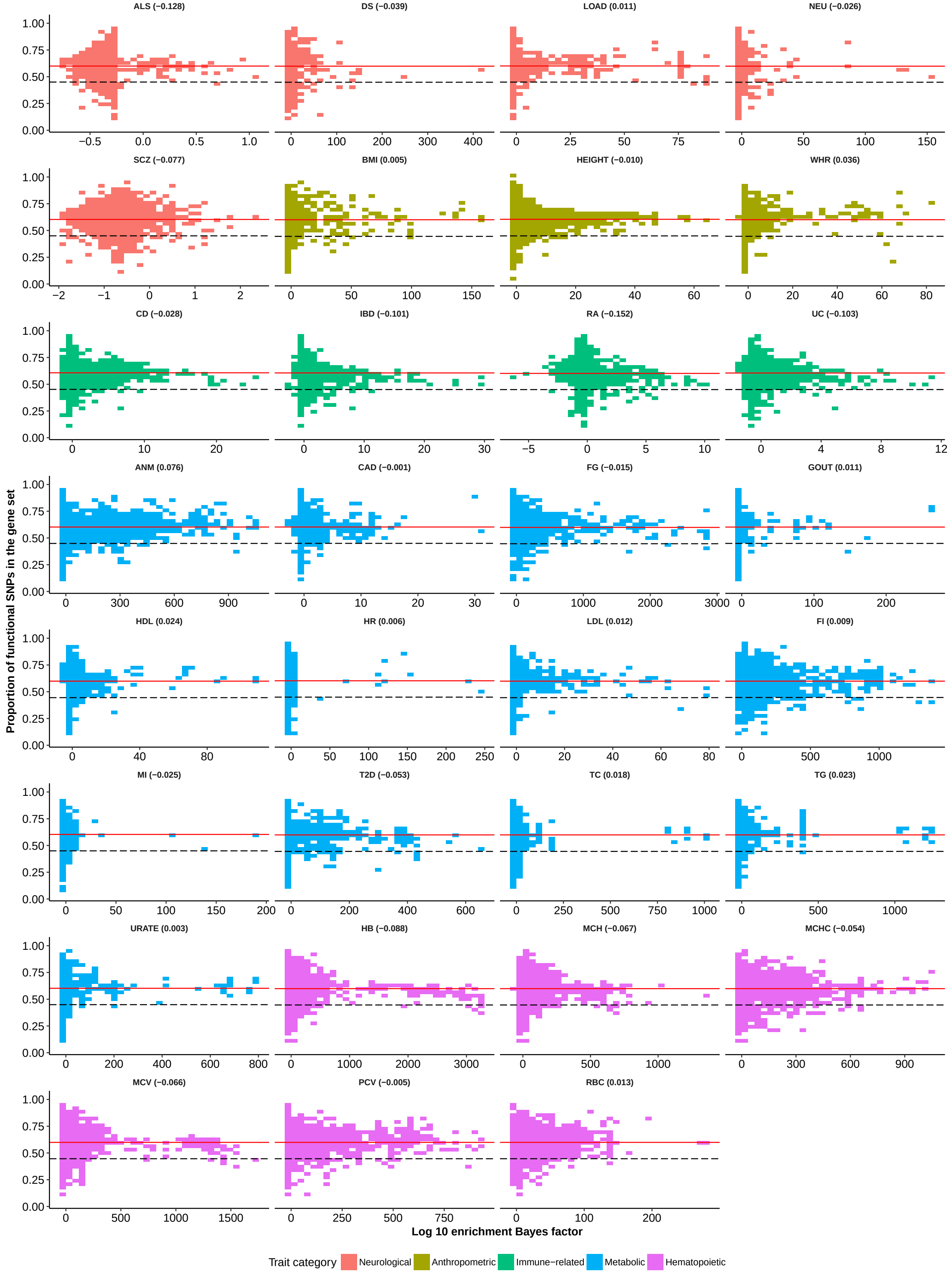

Functional category: PromoterFlanking_Hoffman

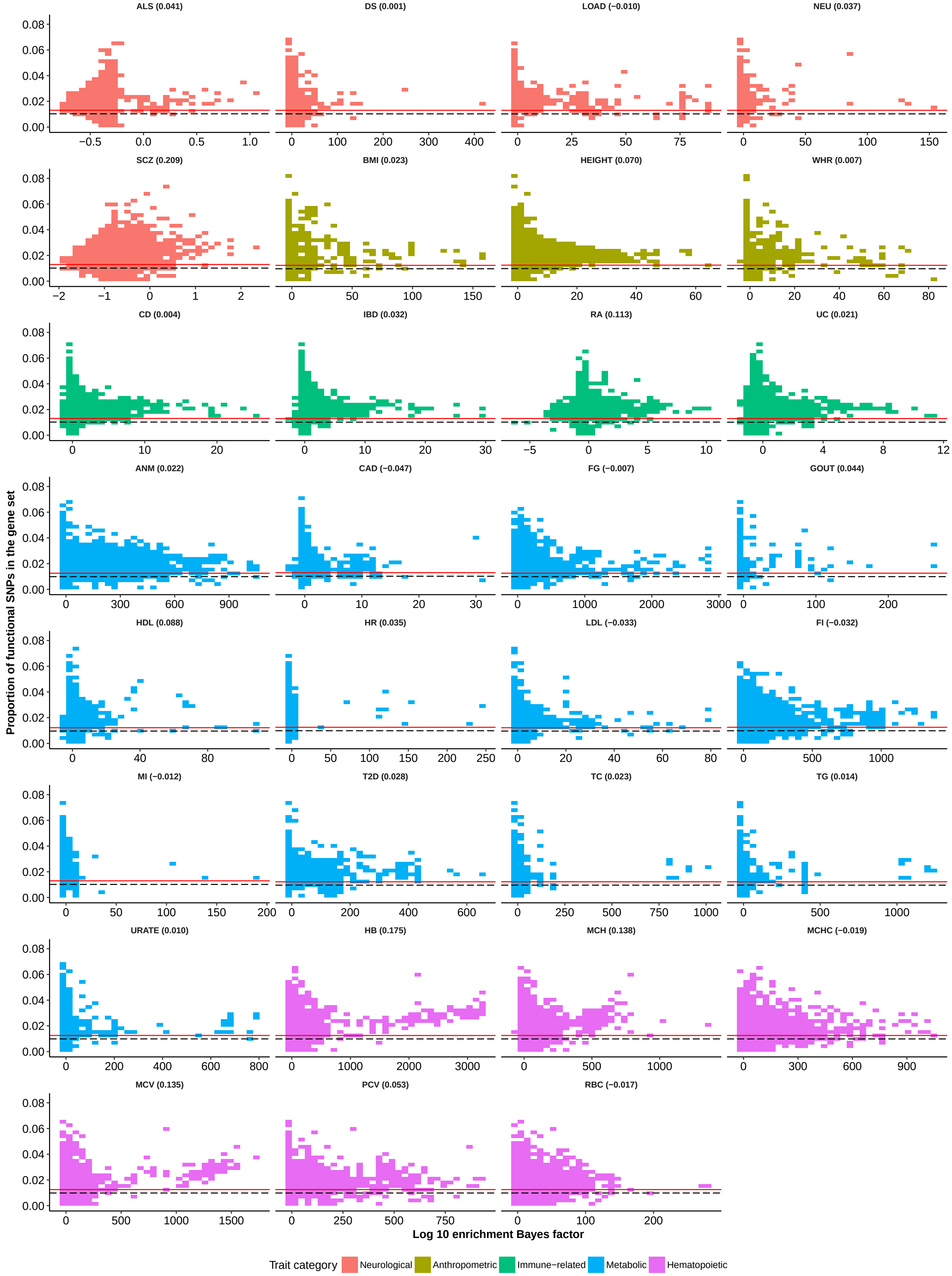

Functional category: PromoterFlanking_Hoffman.extend.500

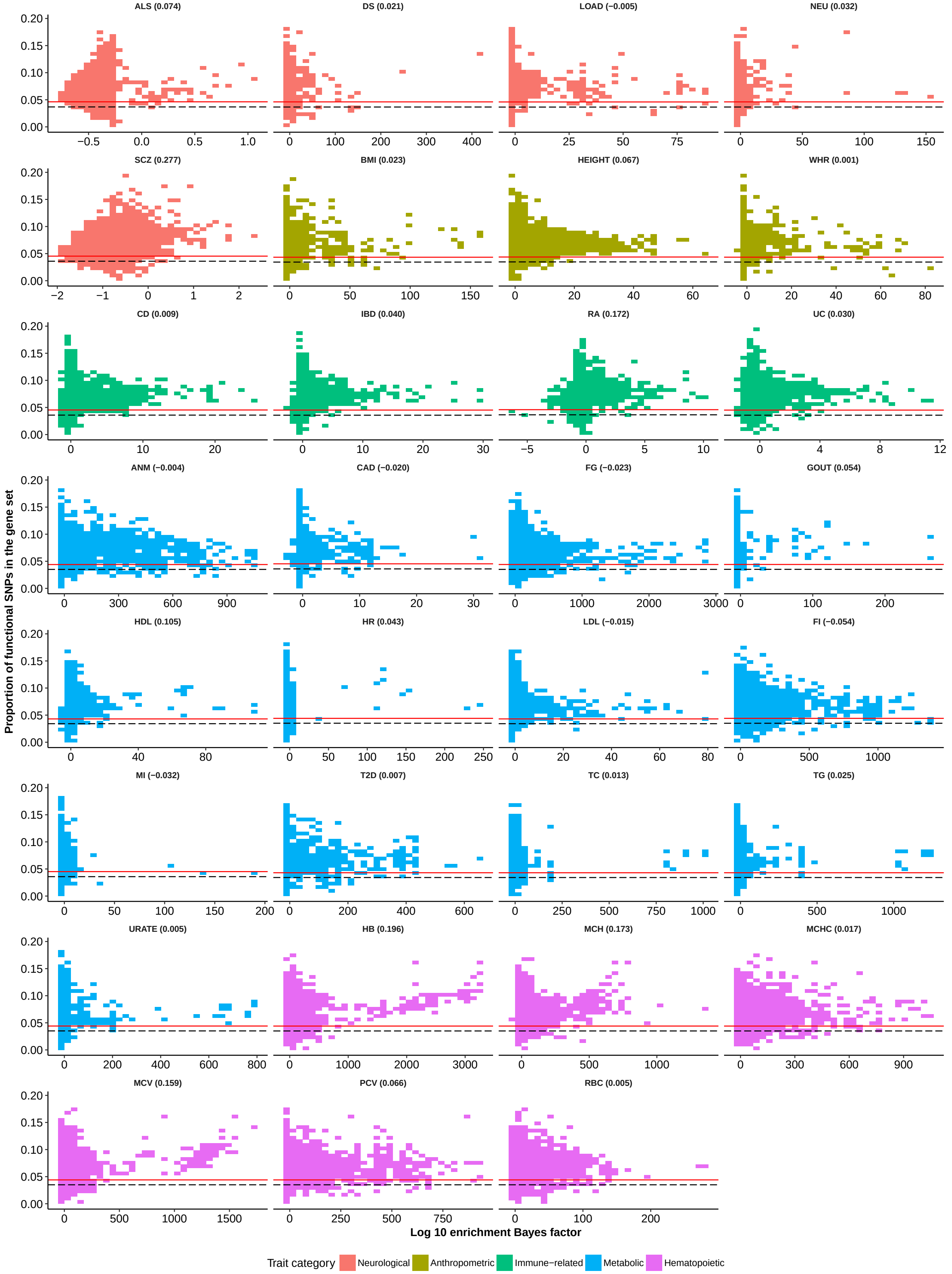

Functional category: Promoter_UCSC

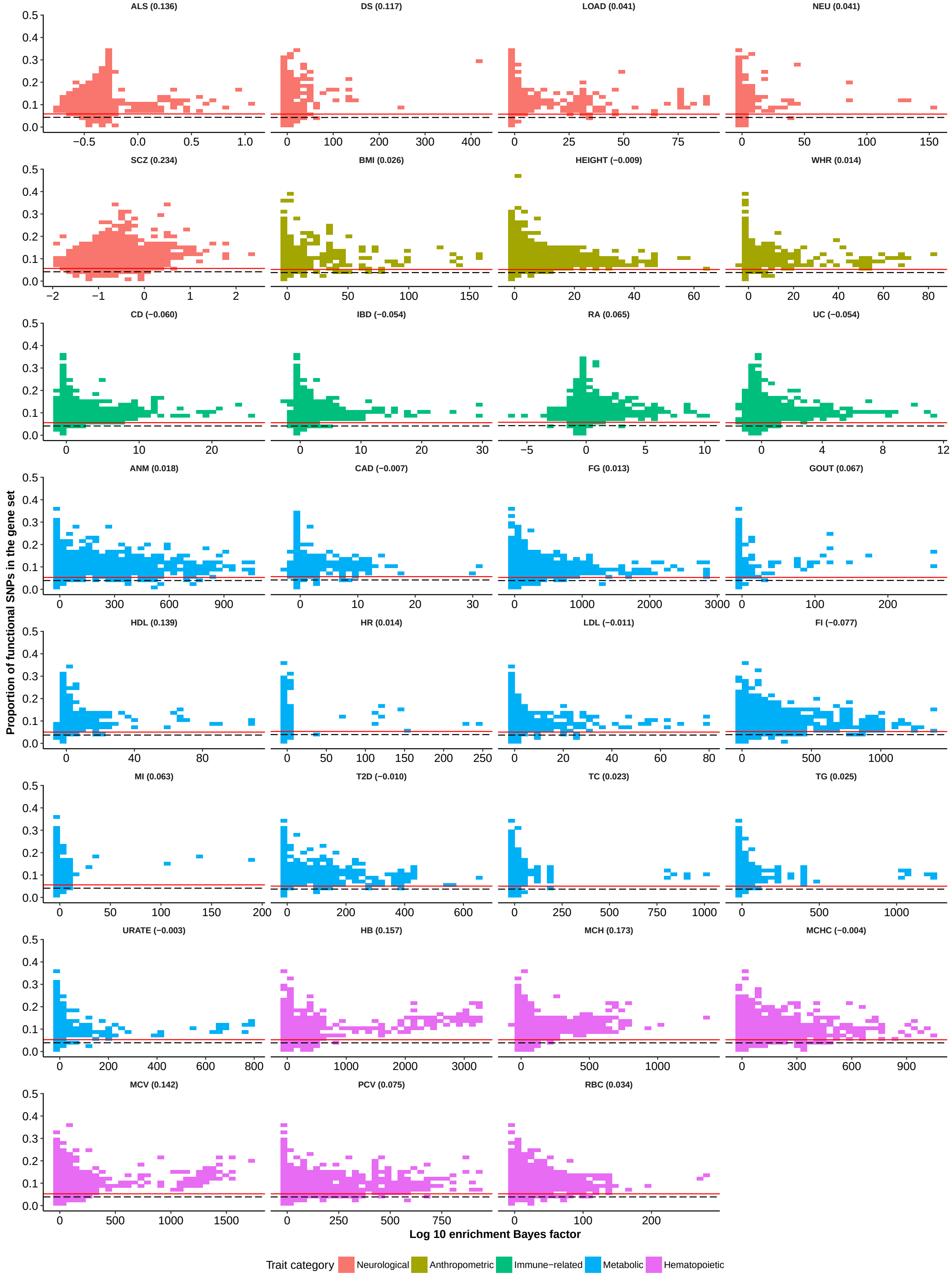

Functional category: Promoter_UCSC.extend.500

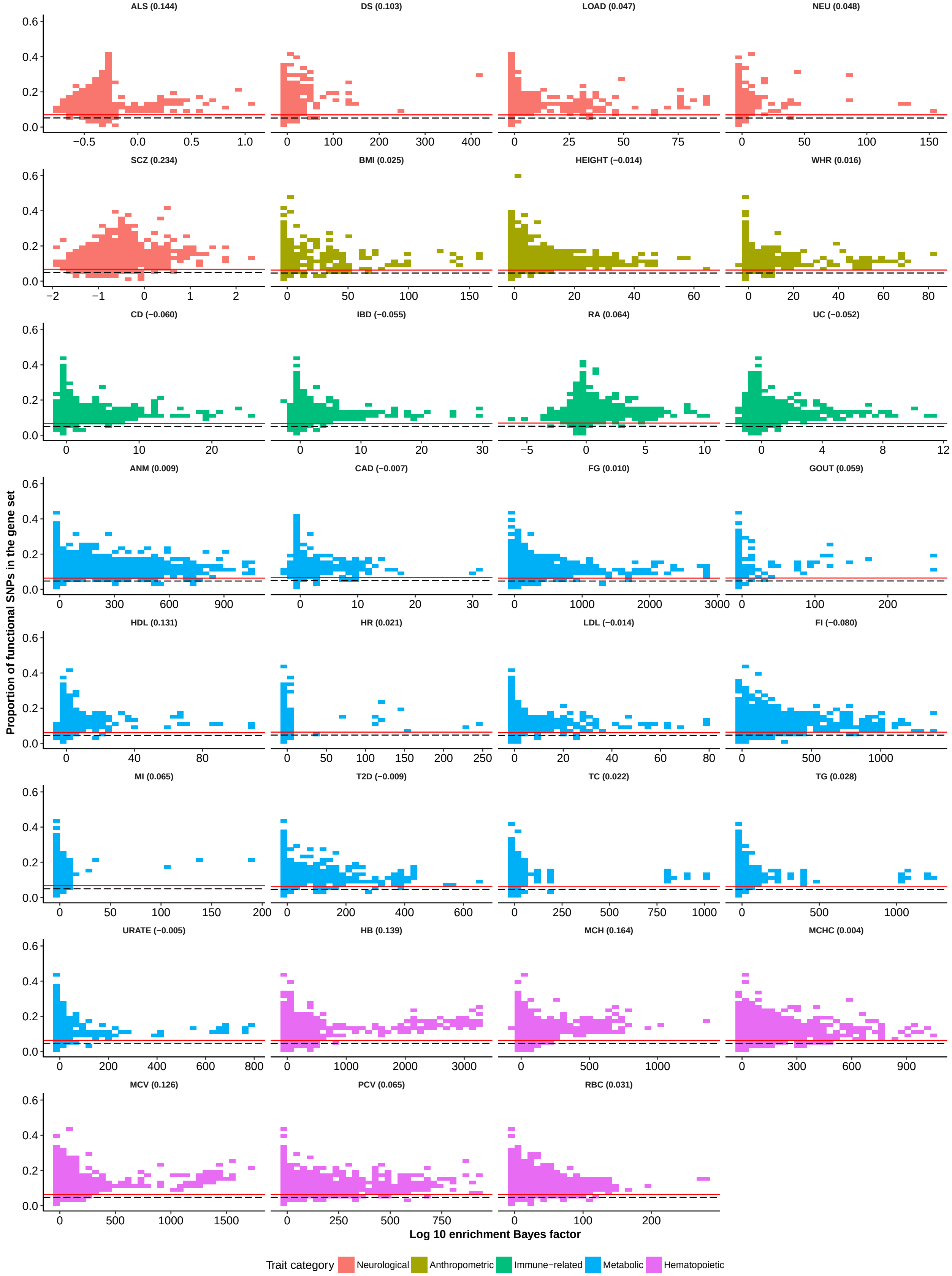

Functional category: Repressed_Hoffman

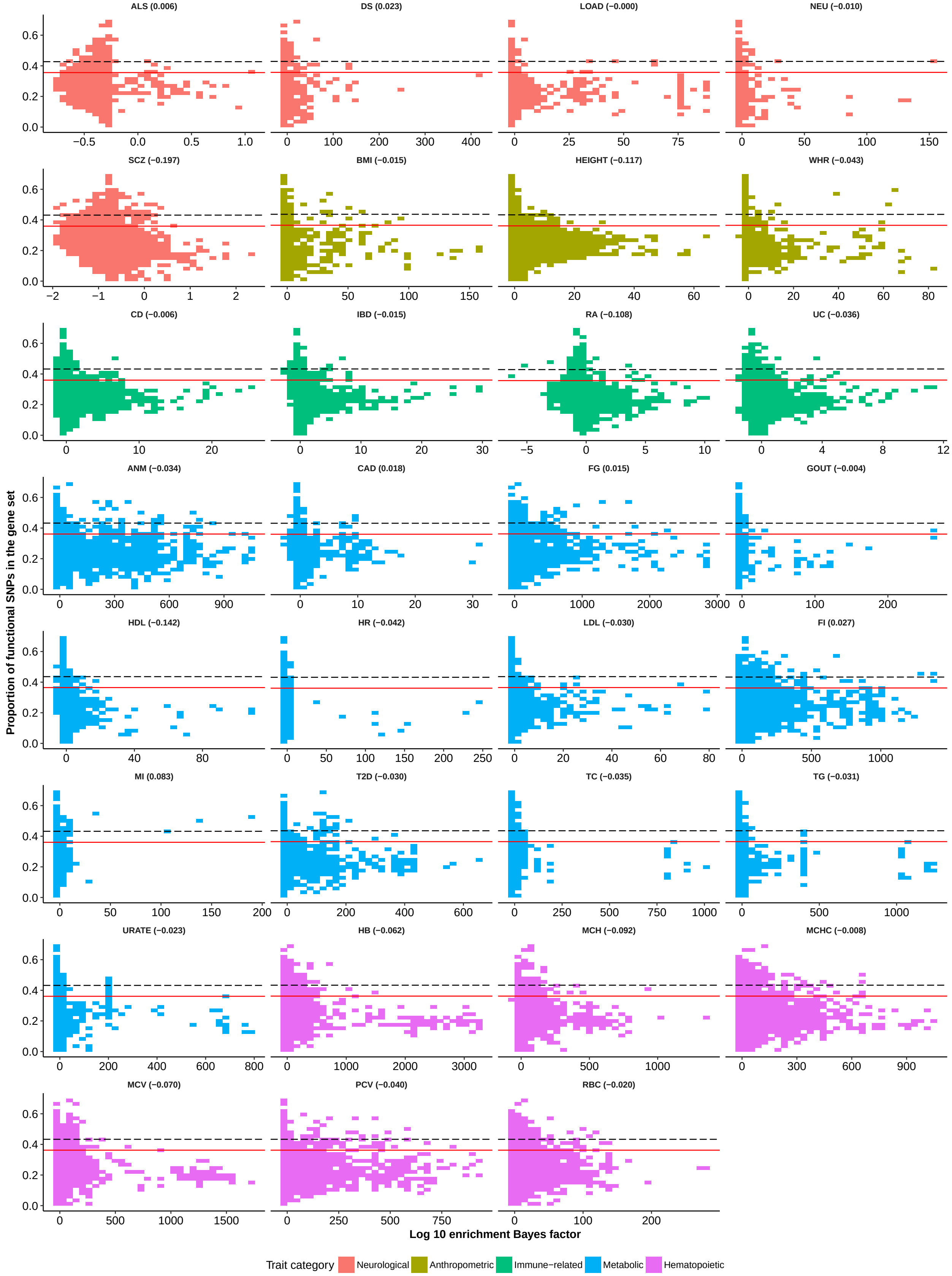

Functional category: Repressed_Hoffman.extend.500

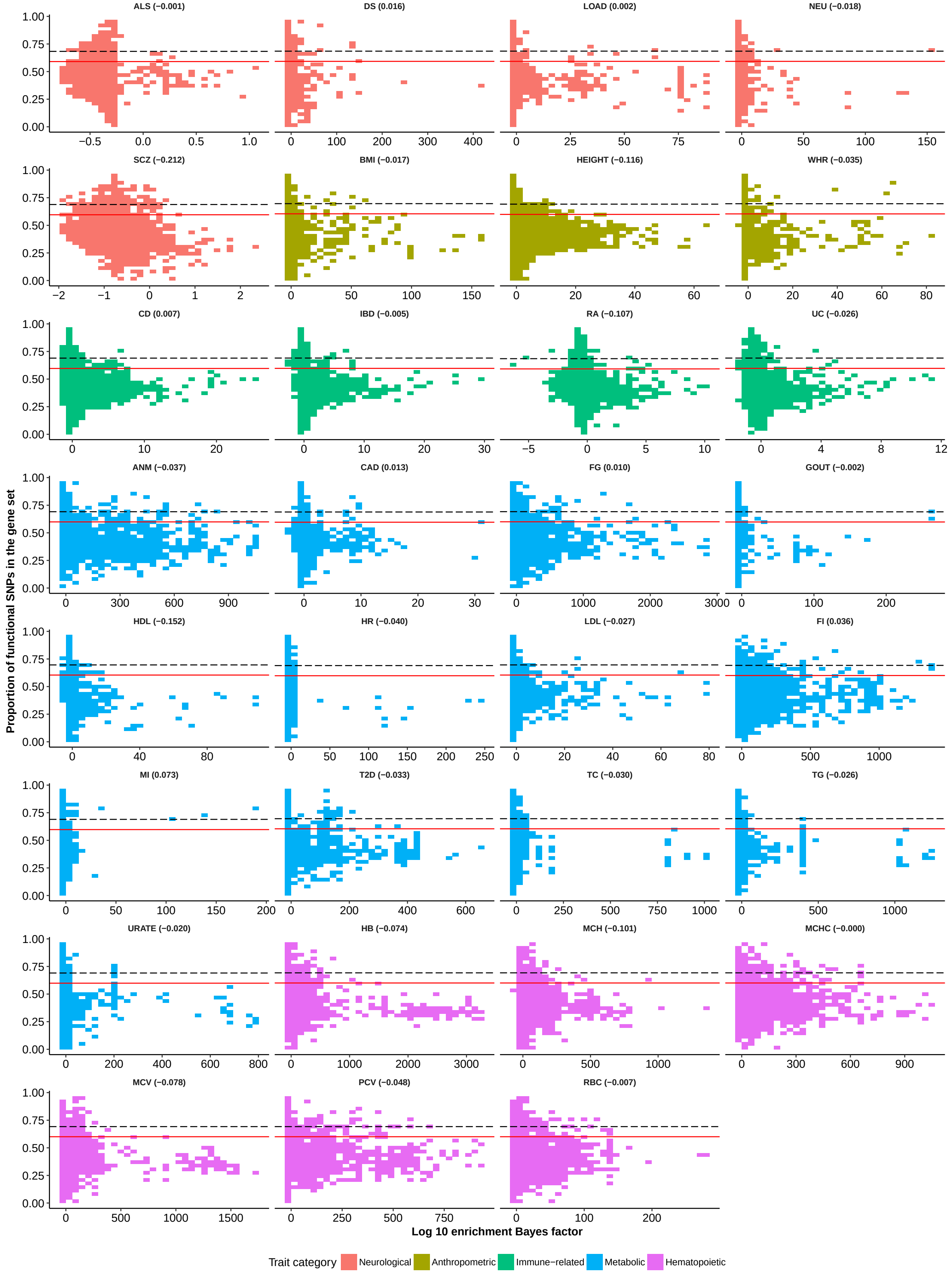

Functional category: SuperEnhancer_Hnisz

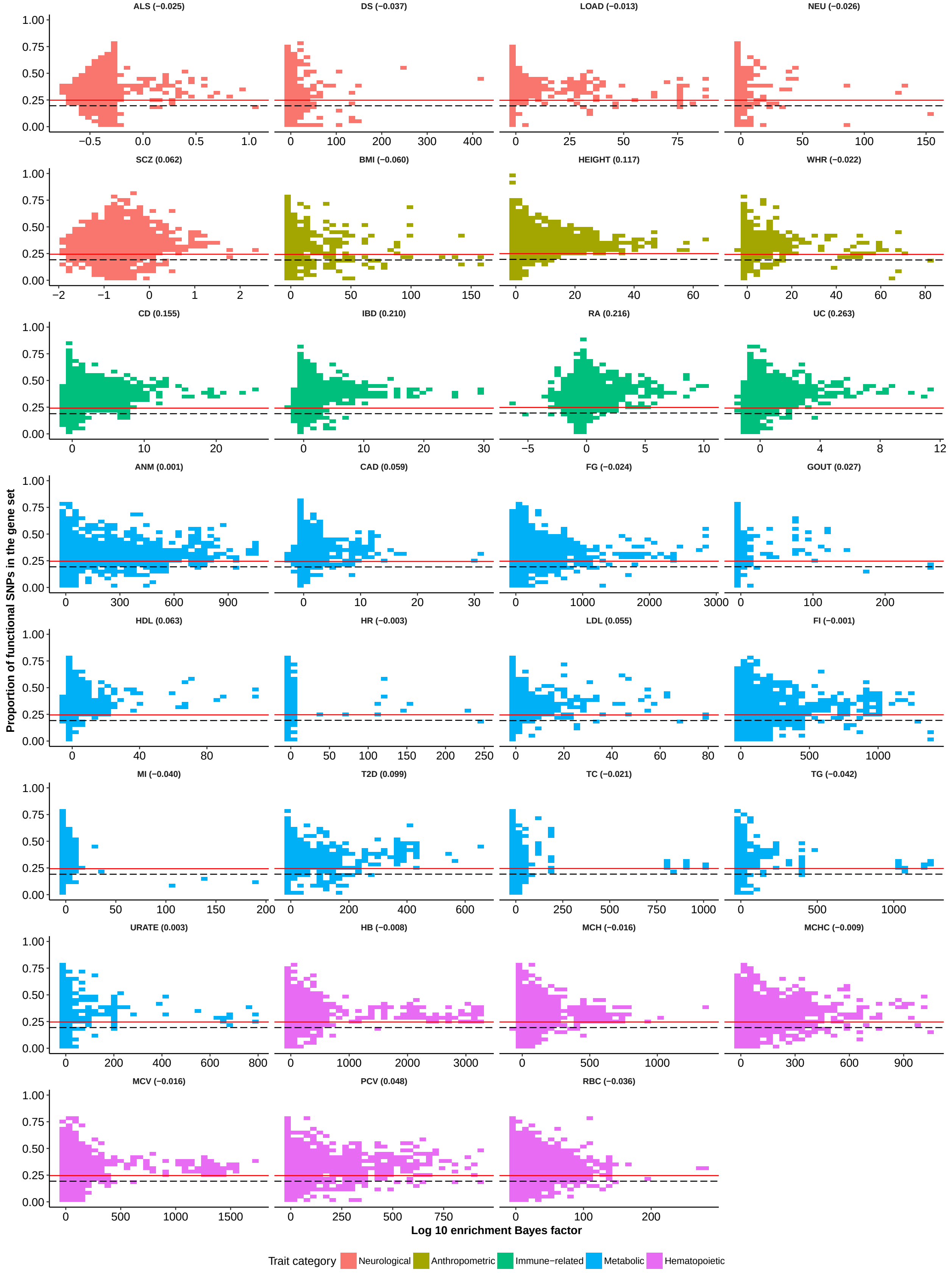

Functional category: SuperEnhancer_Hnisz.extend.500

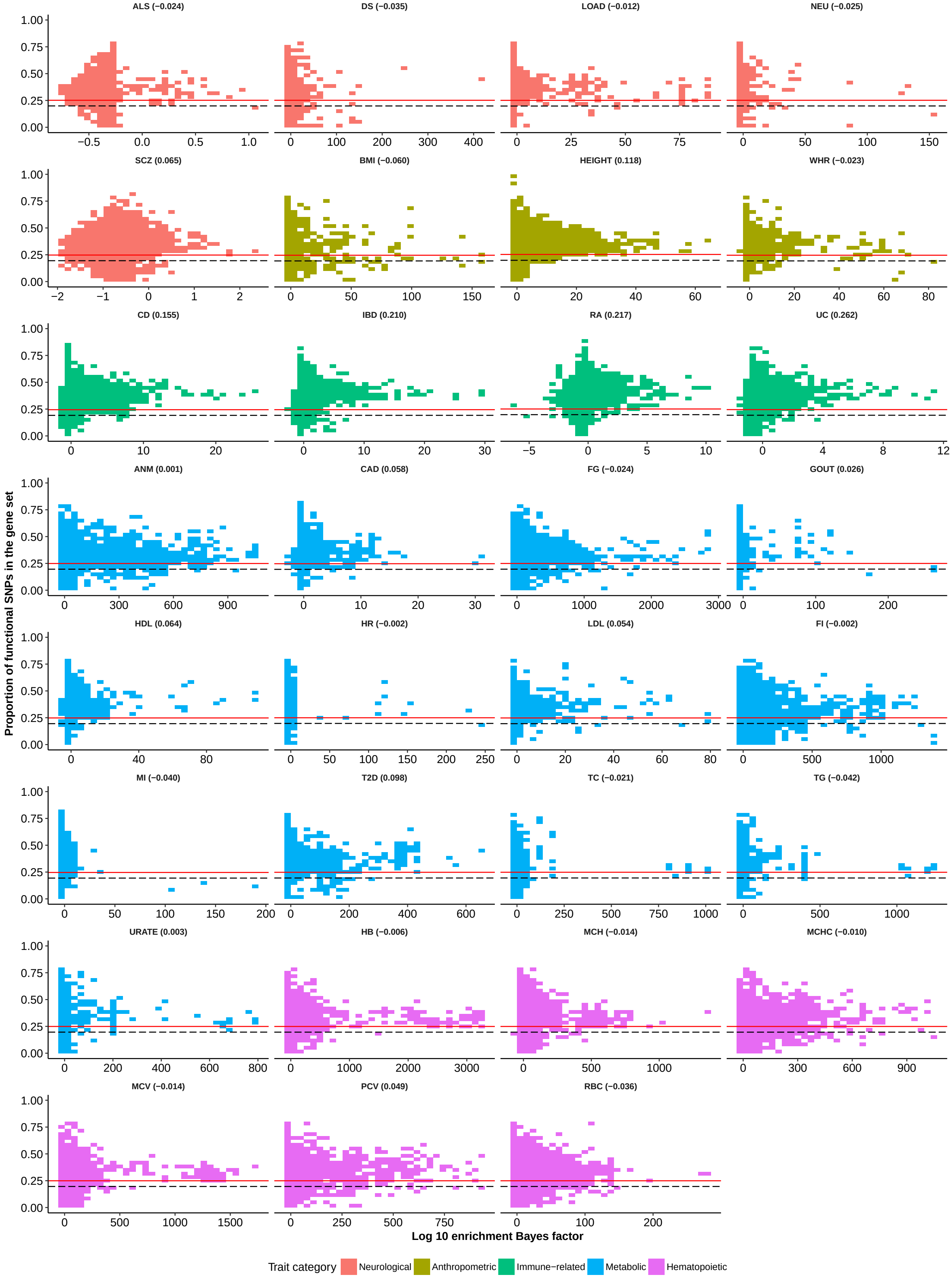

Functional category: TFBS_ENCODE

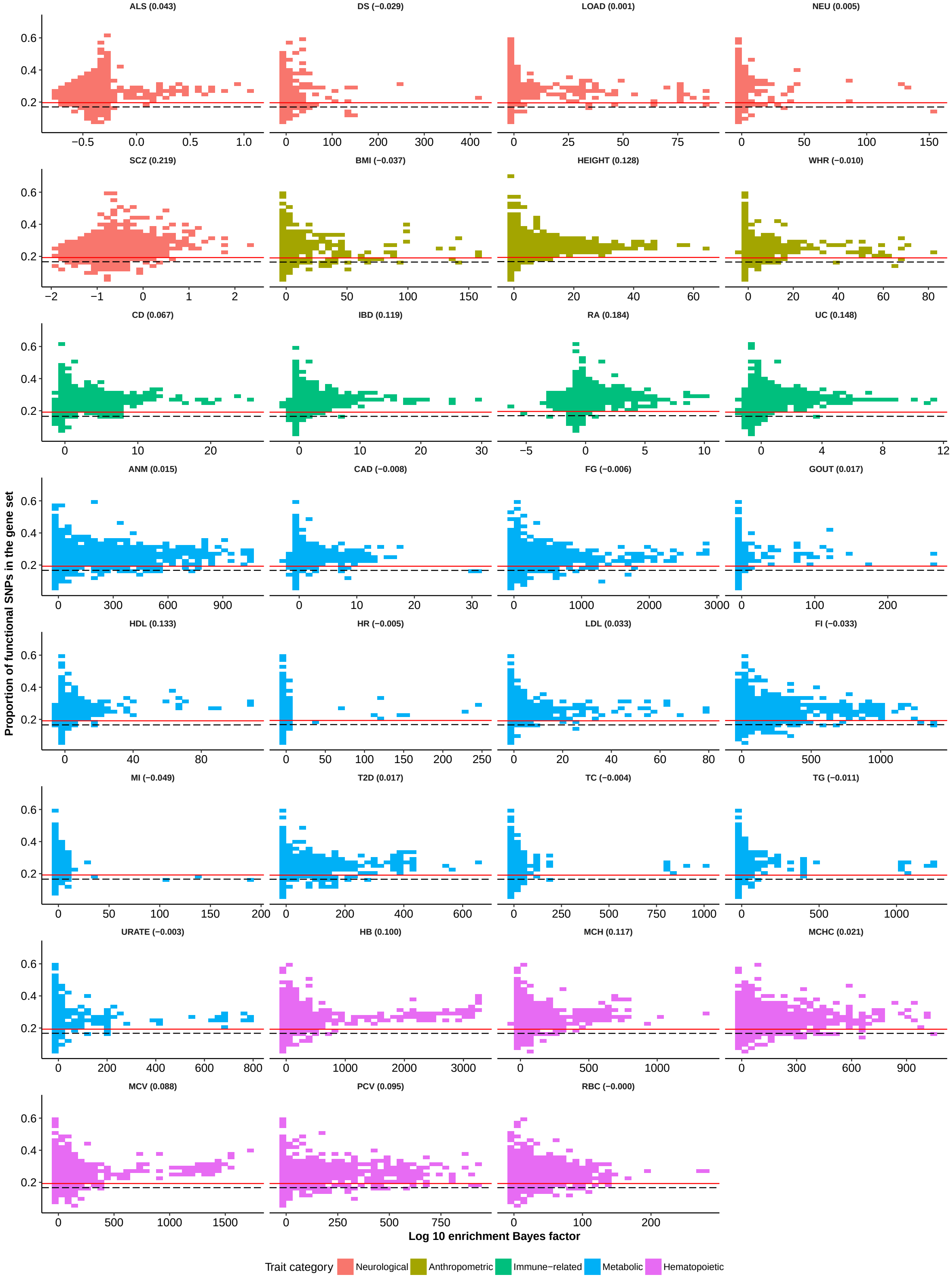

Functional category: TFBS_ENCODE.extend.500

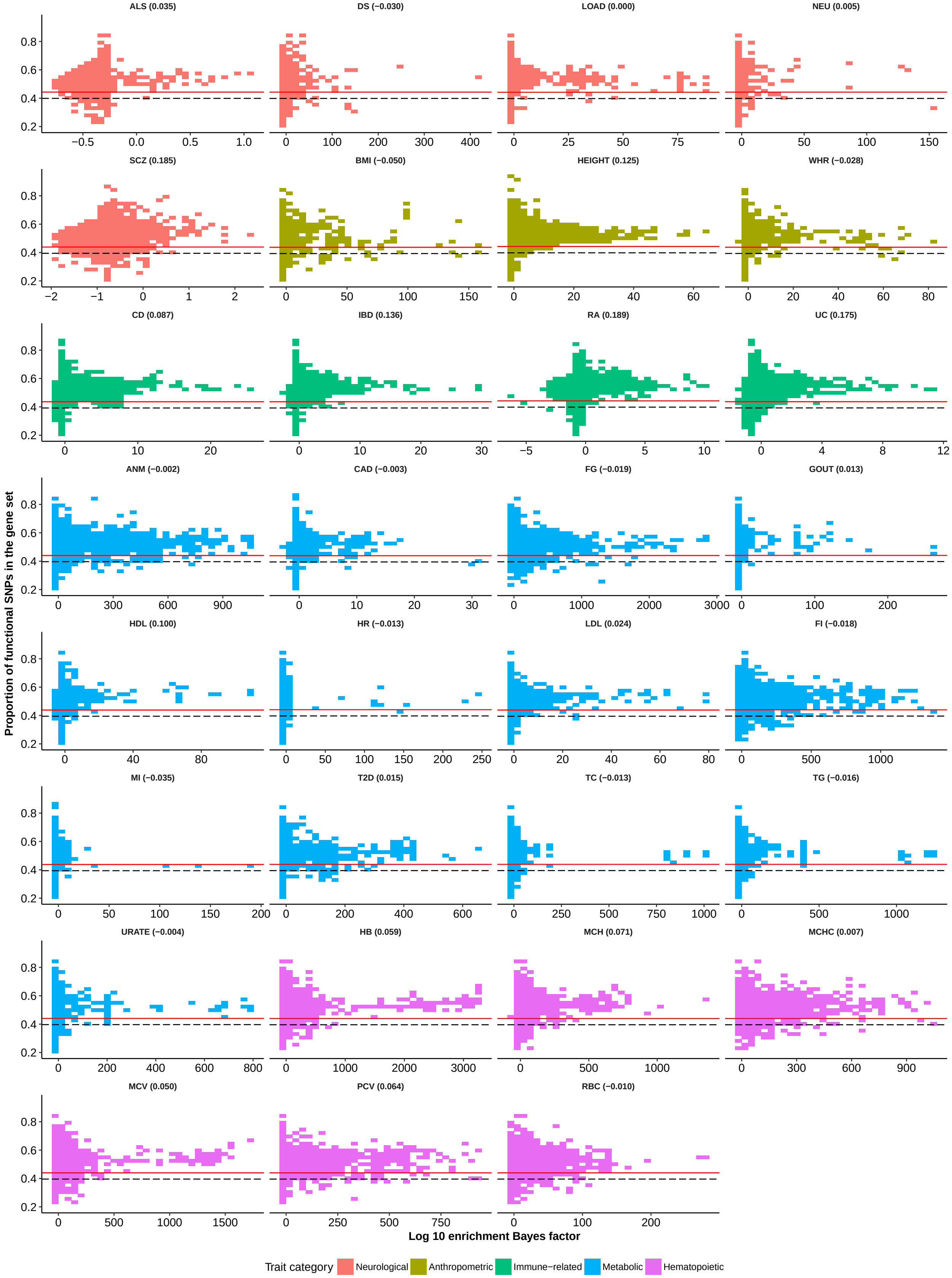

Functional category: Transcribed_Hoffman

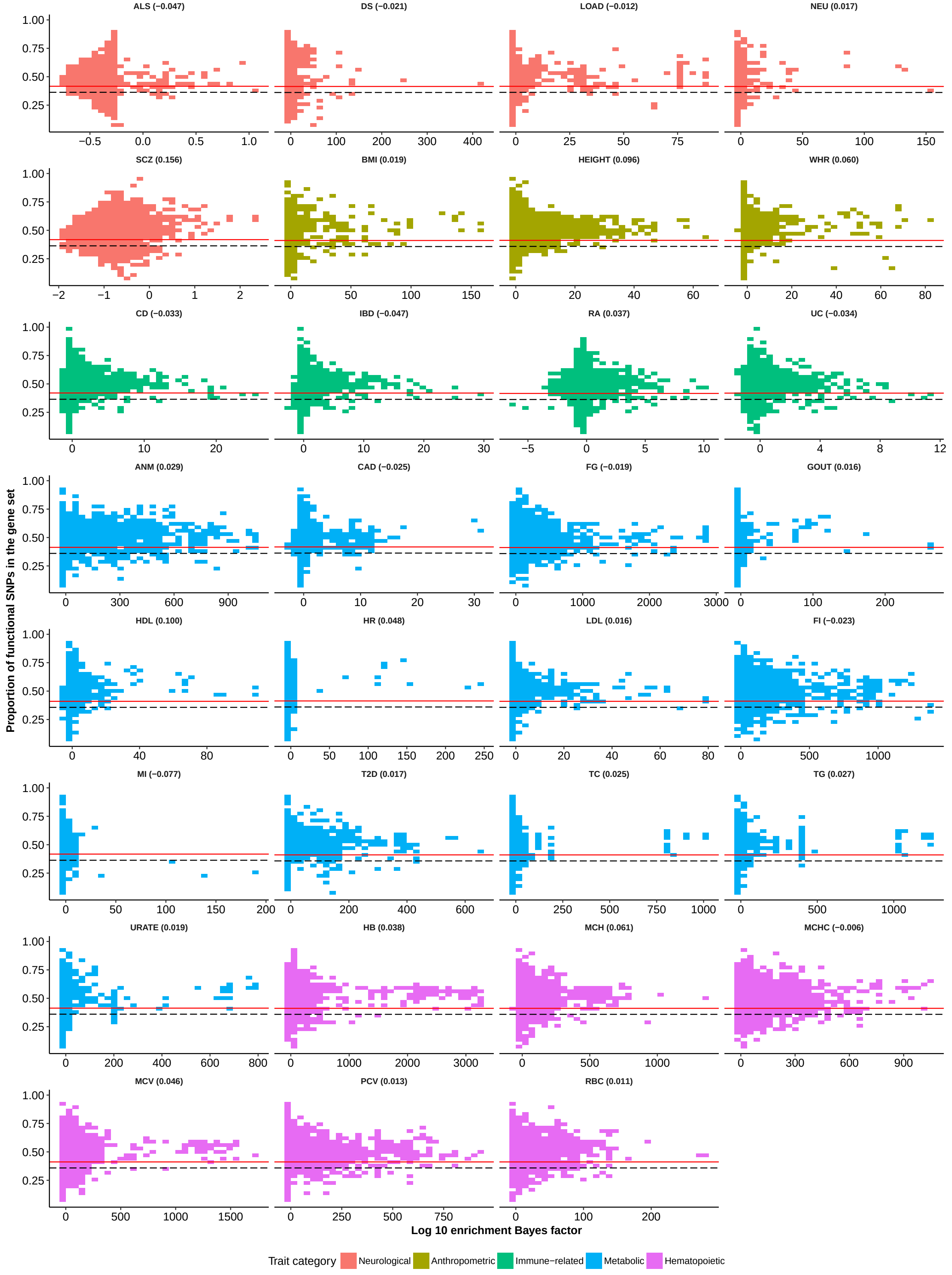

Functional category: Transcribed_Hoffman.extend.500

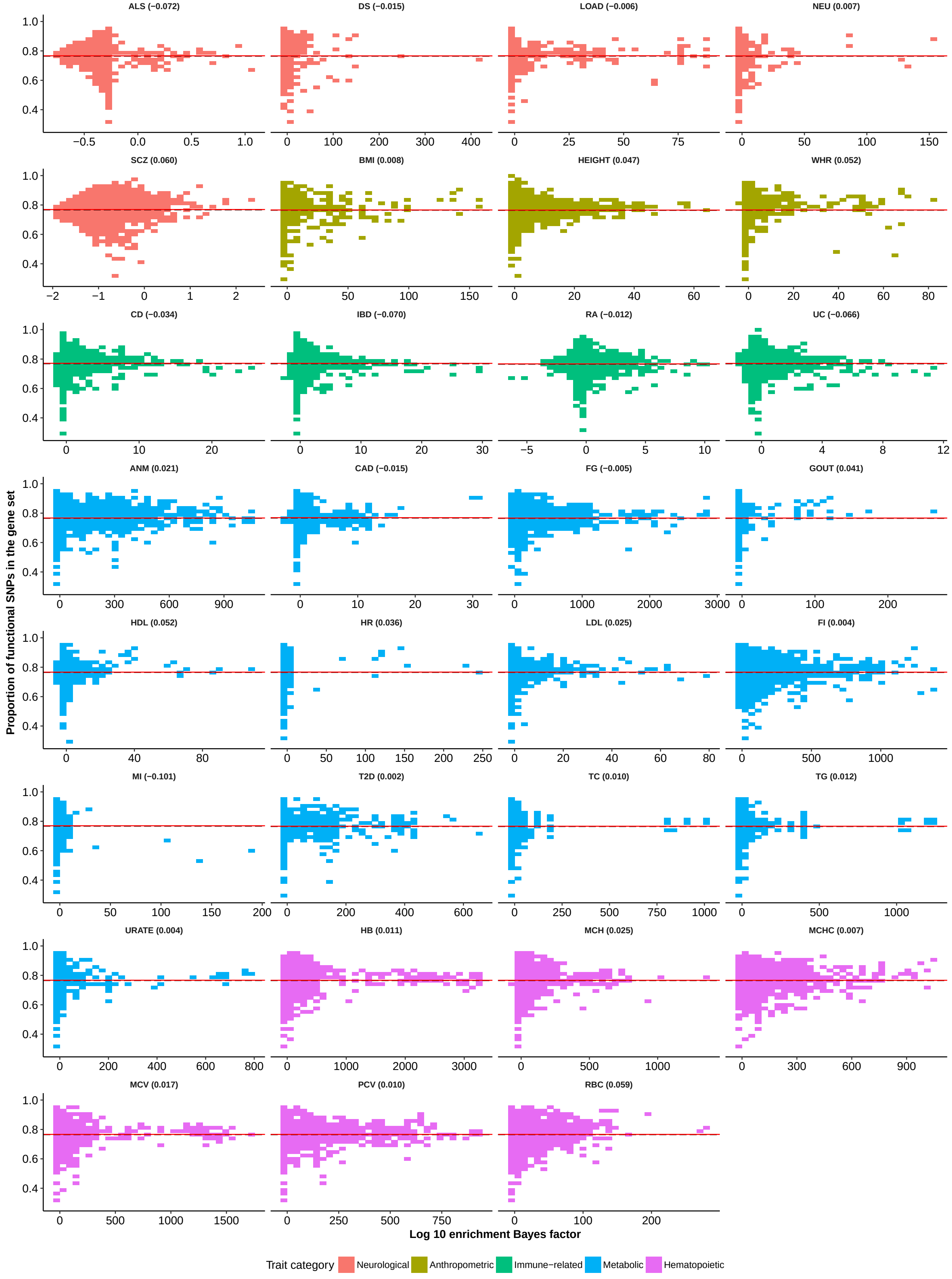

Functional category: TSS_Hoffman

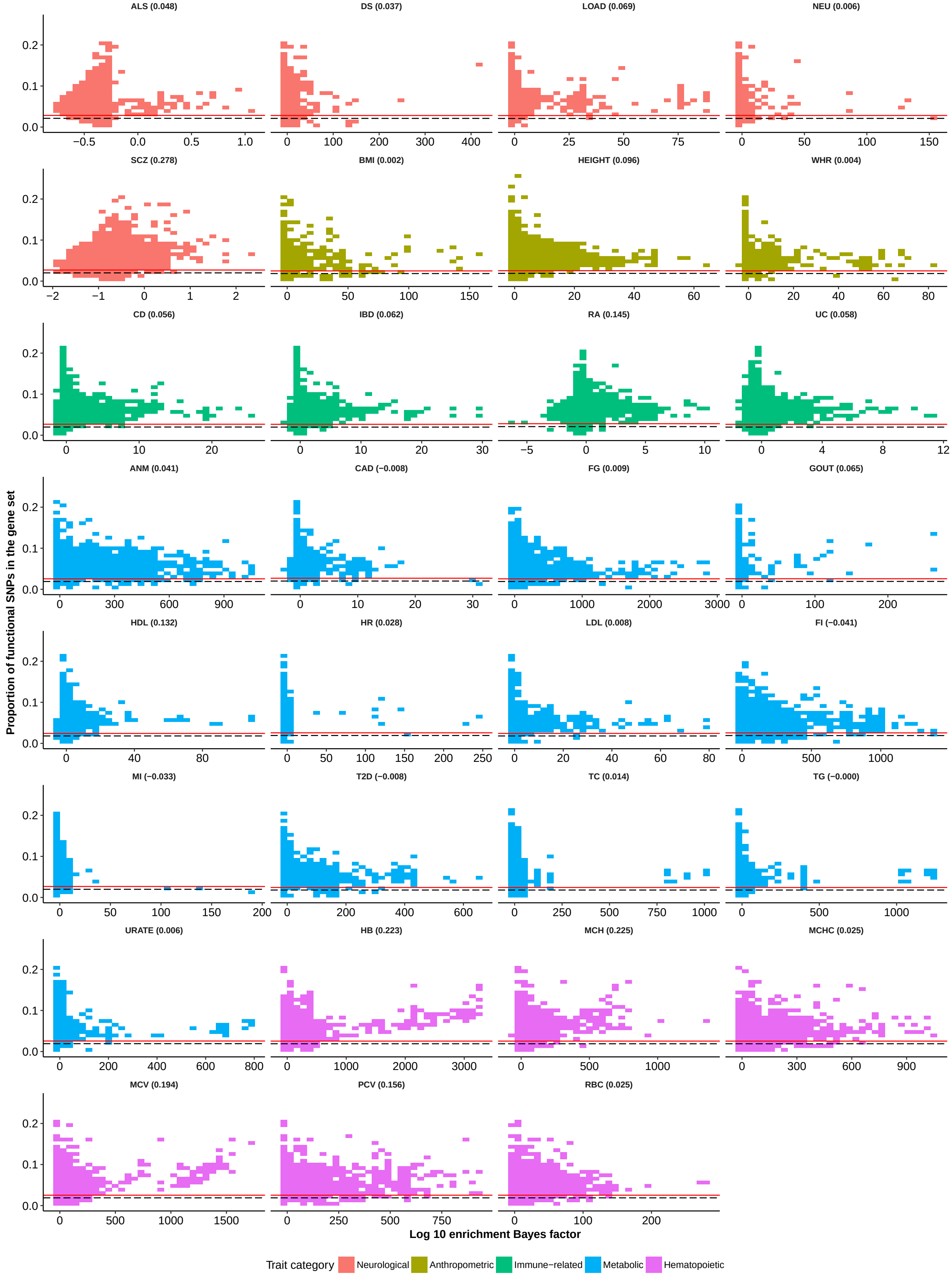

Functional category: TSS_Hoffman.extend.500

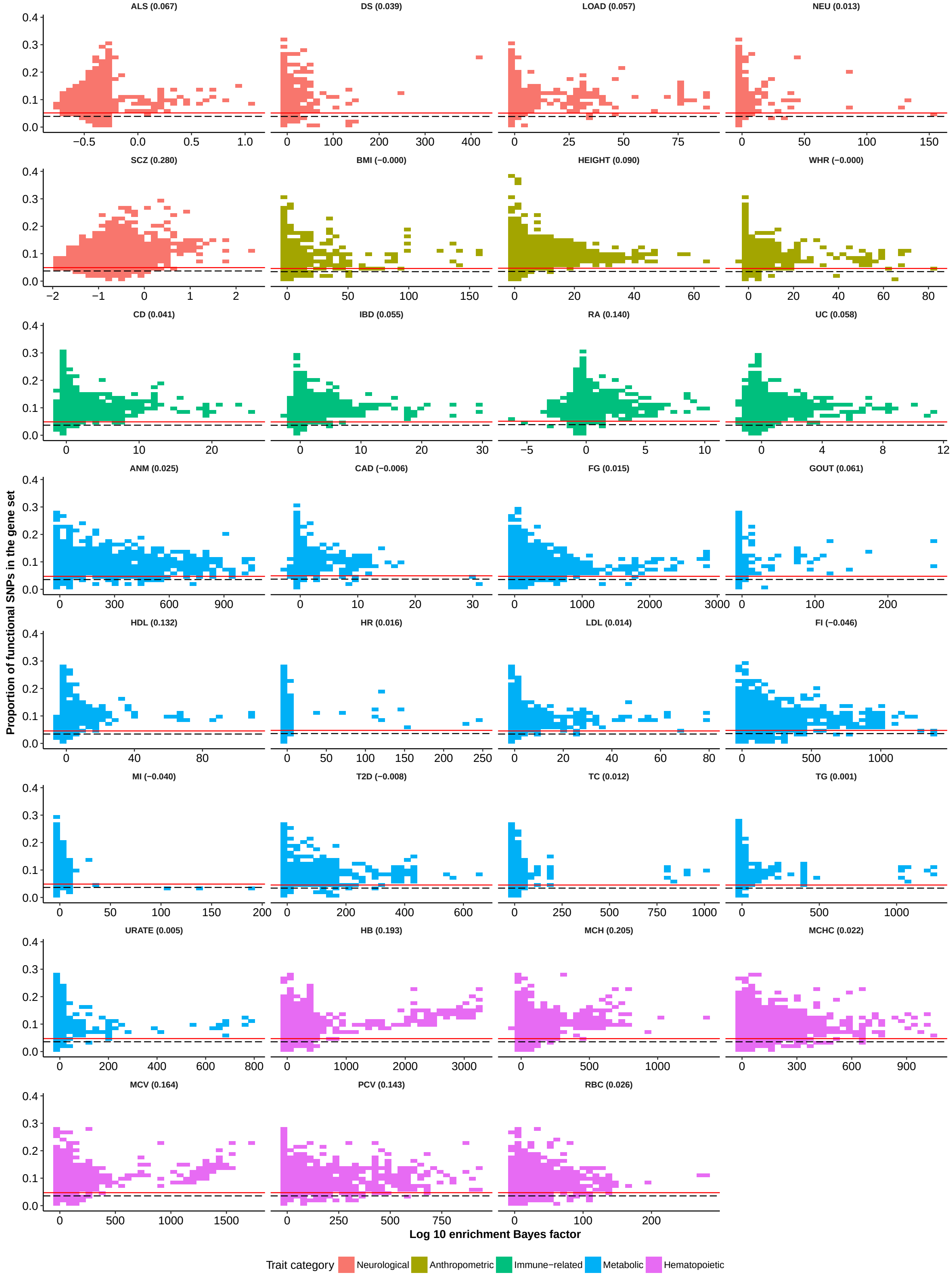

Functional category: UTR_3_UCSC

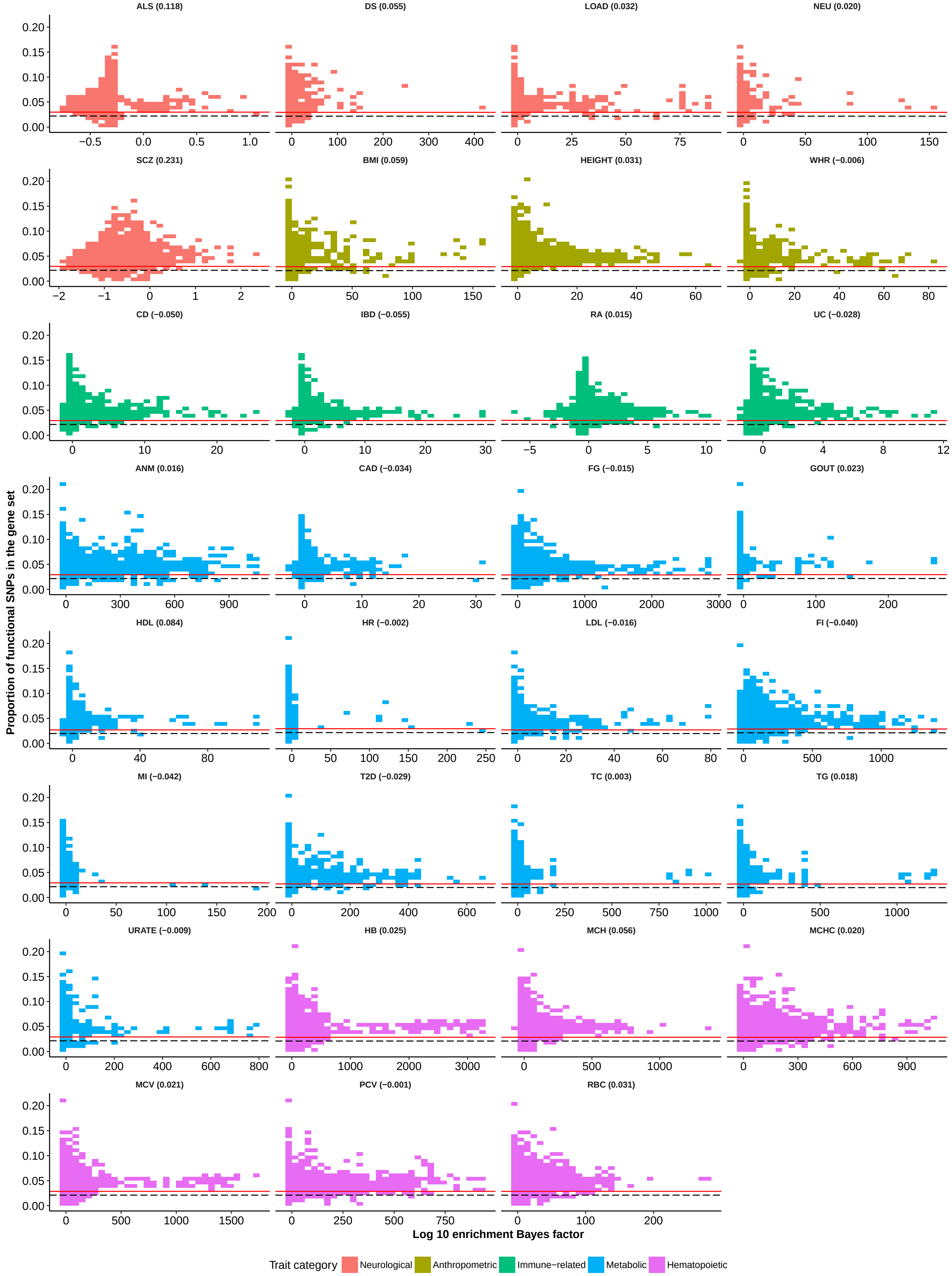

Functional category: UTR_3_UCSC.extend.500

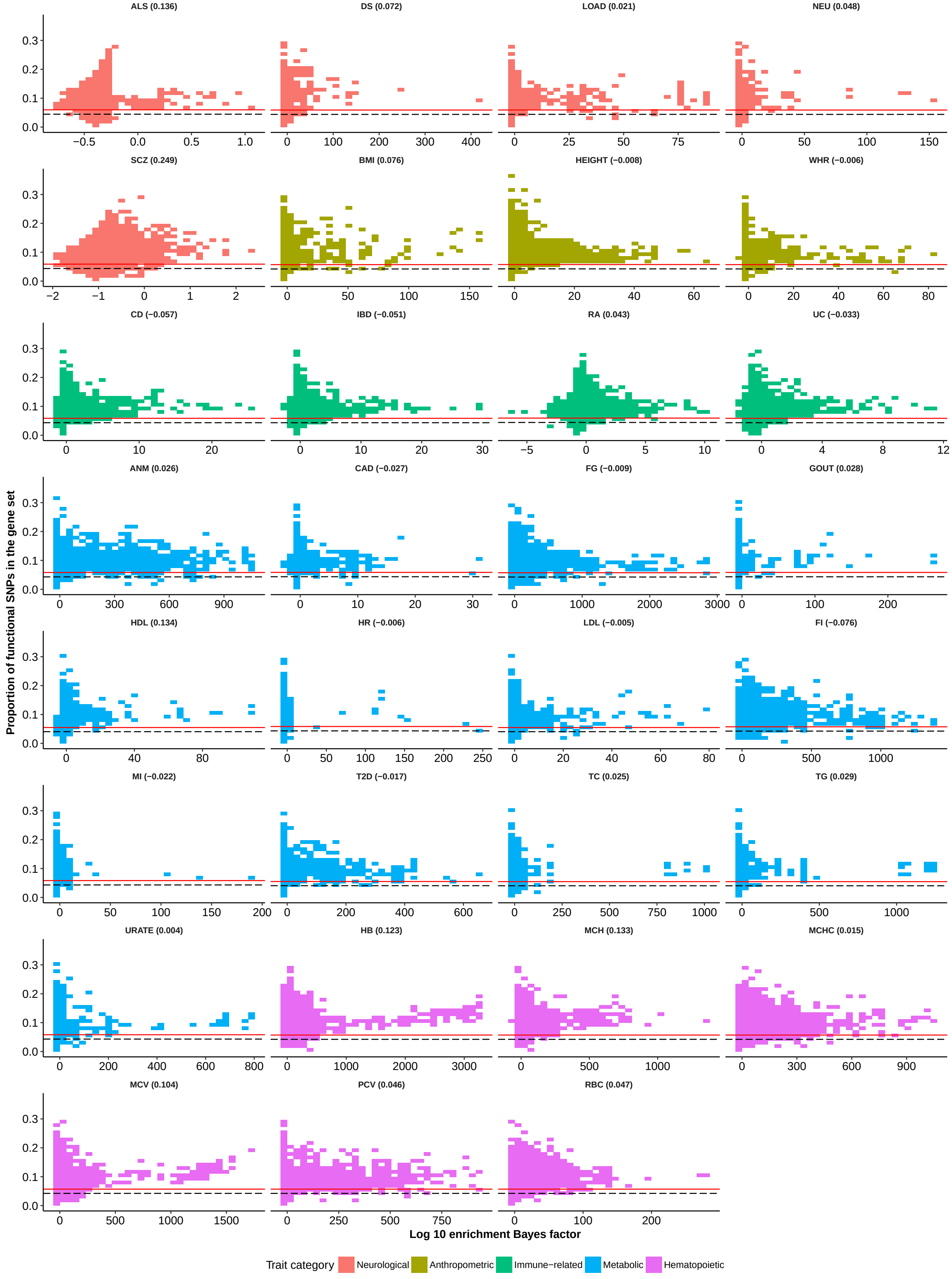

Functional category: UTR_5_UCSC

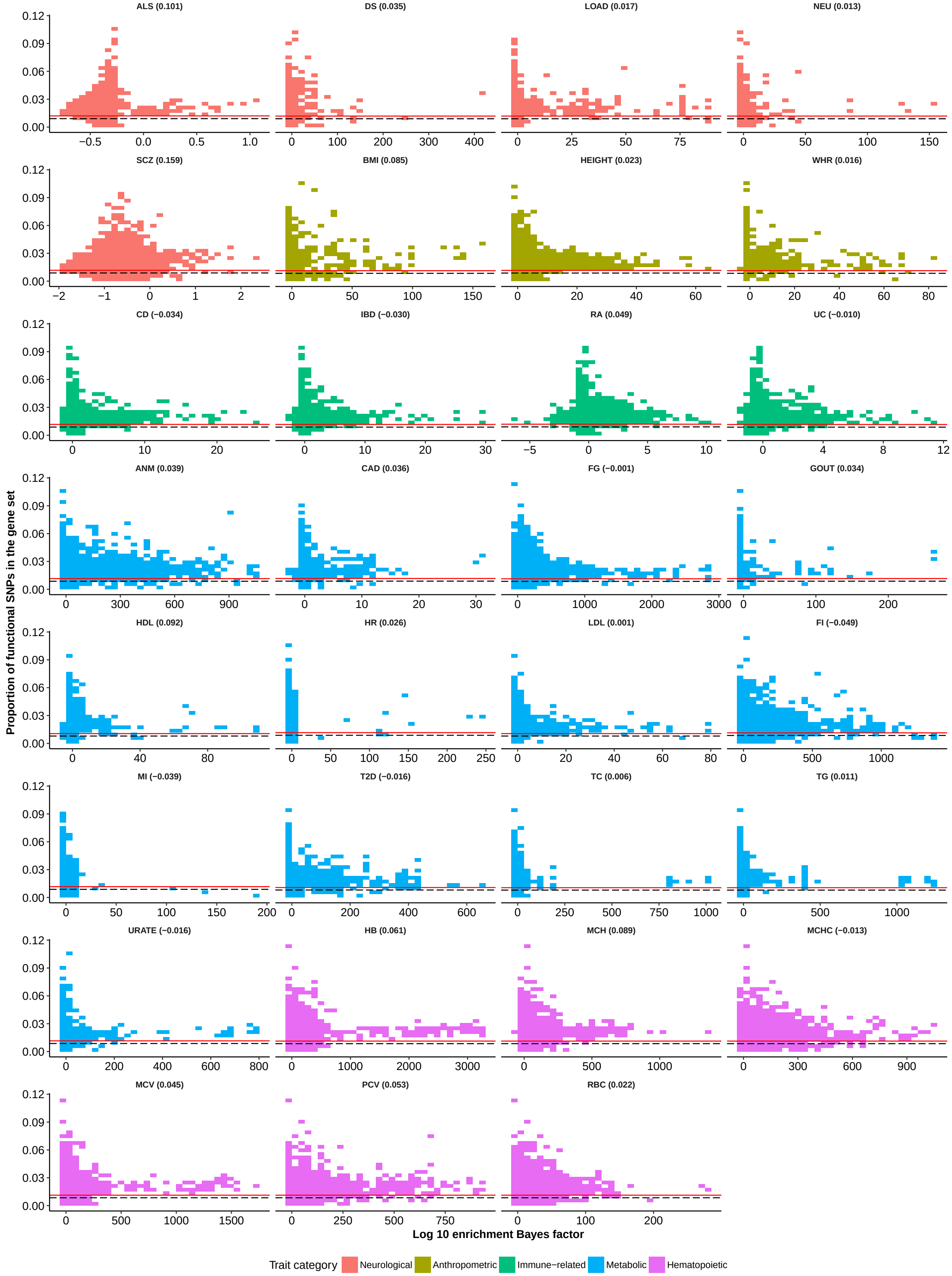

Functional category: UTR_5_UCSC.extend.500

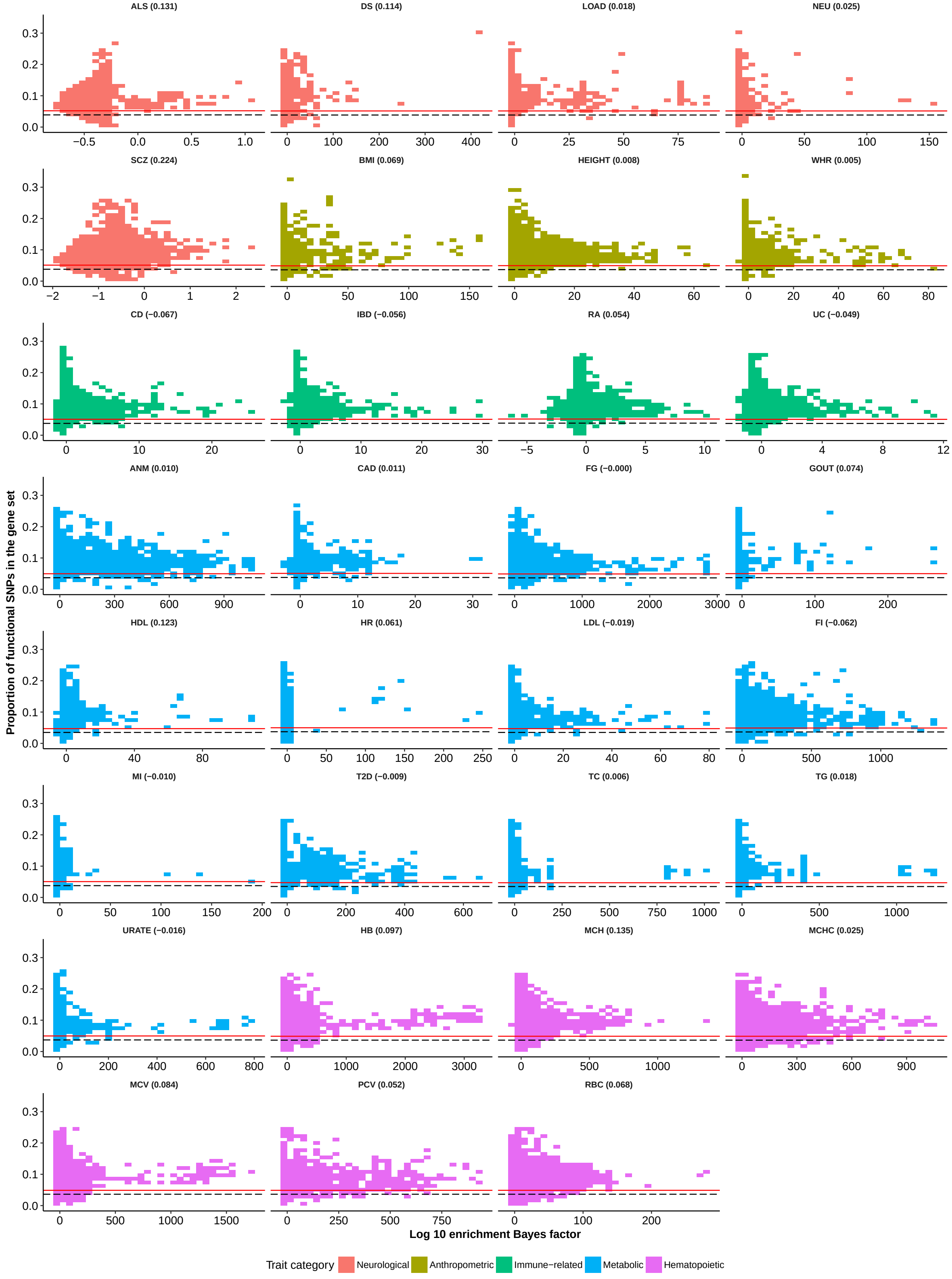

Functional category: WeakEnhancer_Hoffman

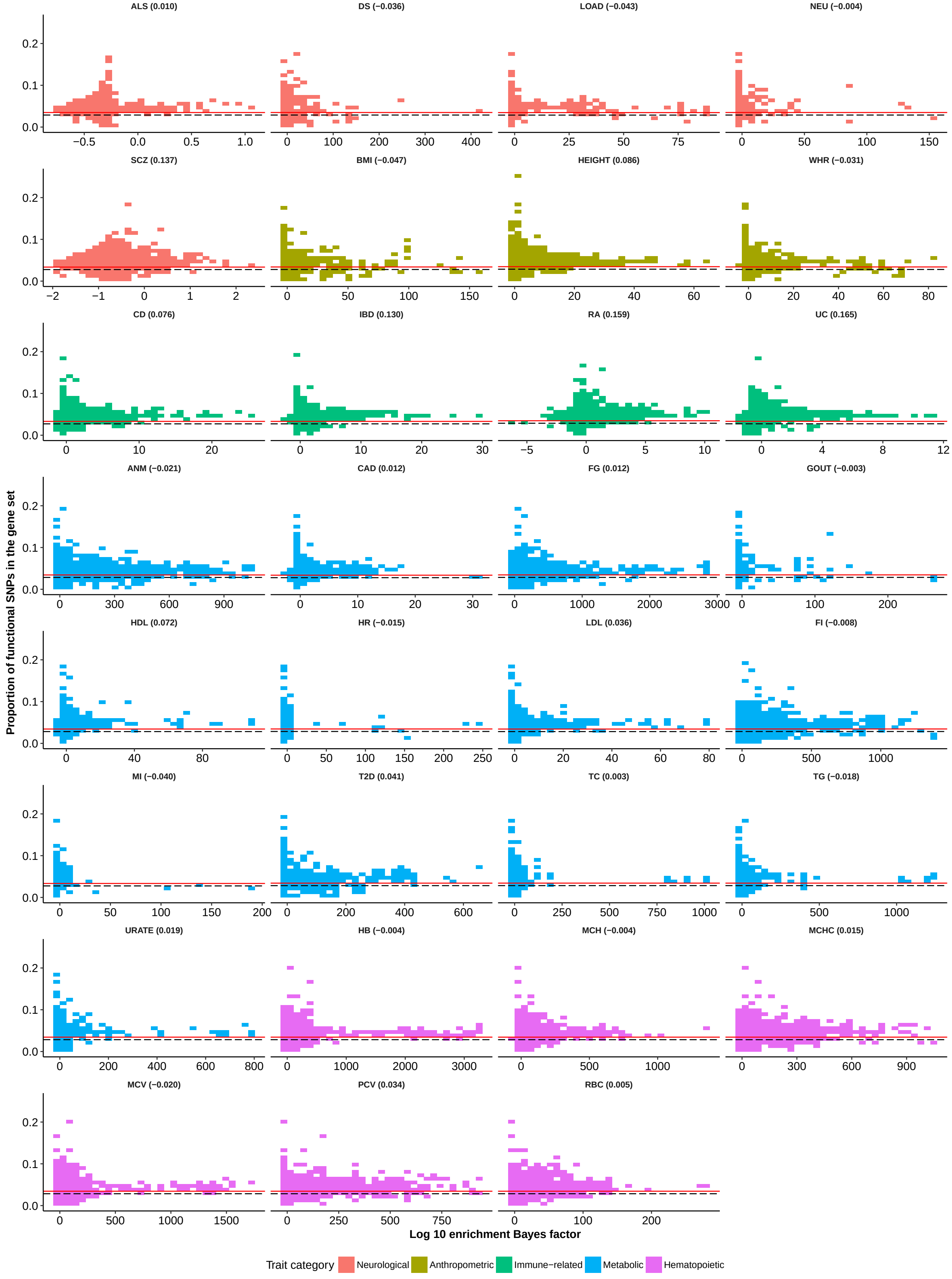

Functional category: WeakEnhancer_Hoffman.extend.500

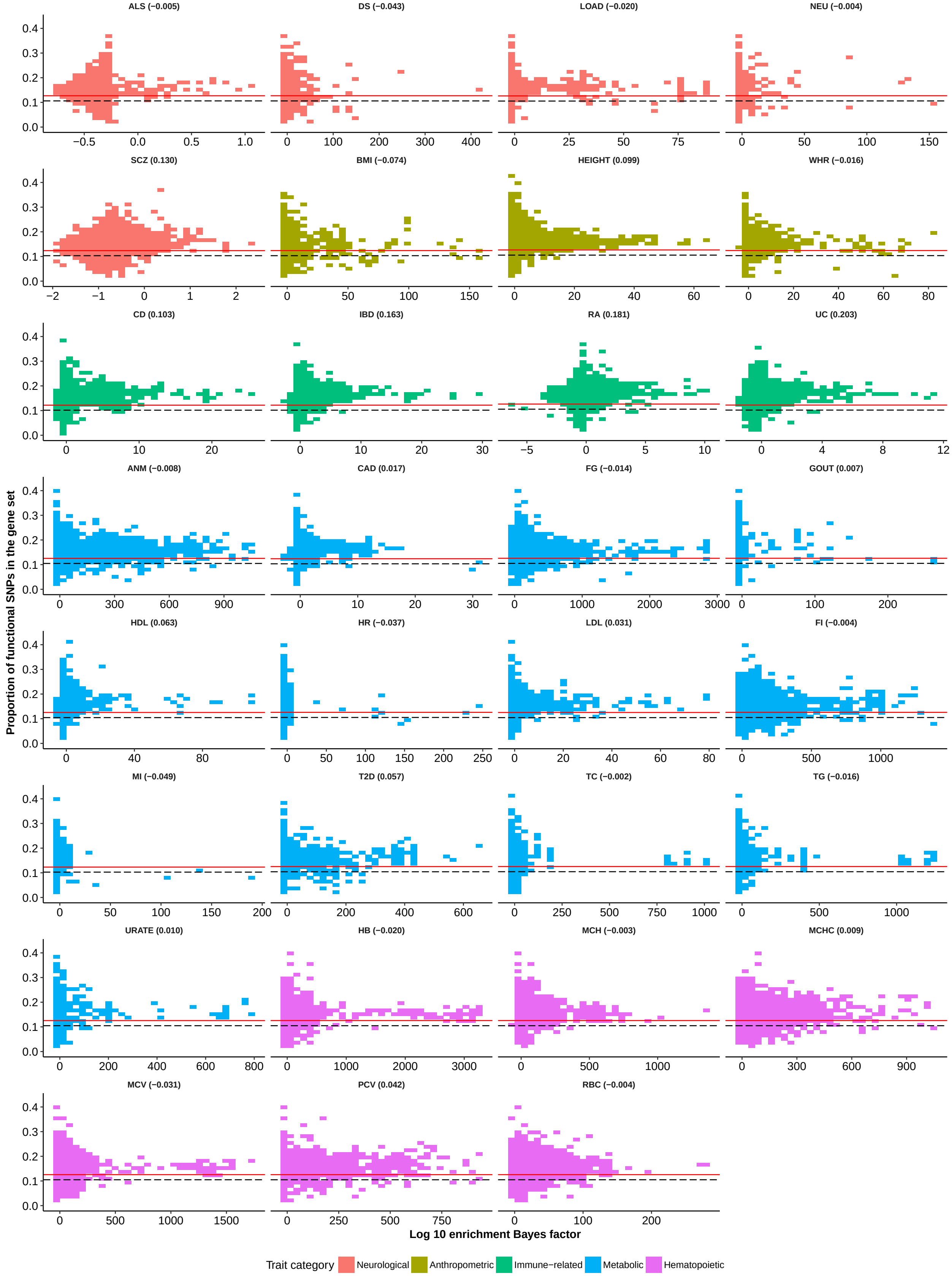

30. Supplementary Figure 22. Distribution of Bayes factors for enrichment of 3,913 biological pathways in 31 phenotypes. These results are generated from the Round 1 enrichment analyses (see **Supplementary Table 7** for details).

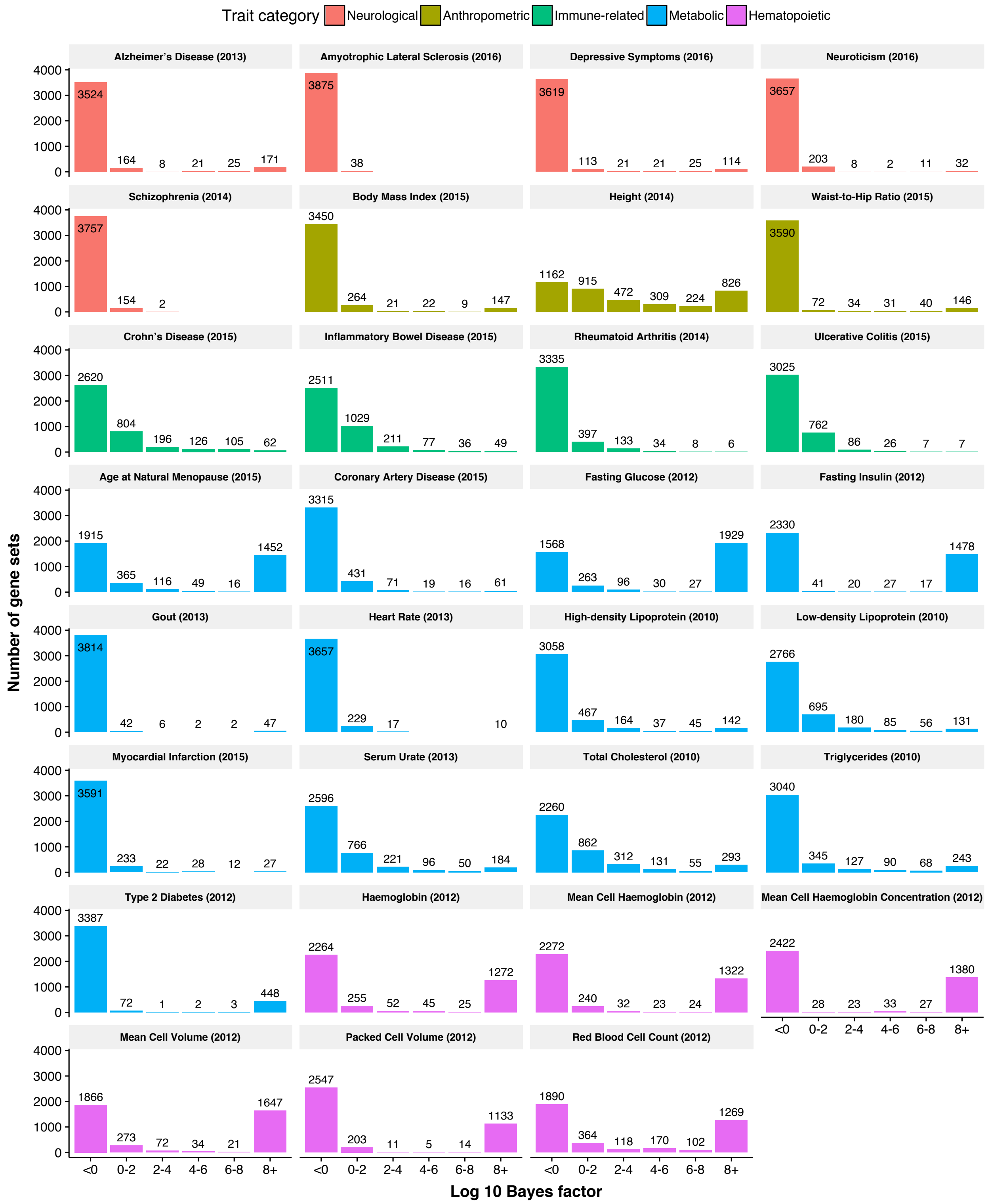

31. Supplementary Figure 23. Gene set overlap among top 6 most enriched pathways for each of 31 phenotypes. Each barplot below shows the gene set overlap of the top 6 pathways with at least 10 member genes that show the largest enrichment Bayes factors (BFs) for each trait in Round 2 analyses (see **Supplementary Table 7** for details). If multiple pathways from different databases have the same pathway description, only the one with the largest BF is displayed here. For each barplot, the yellow bars correspond to the pathway with the largest enrichment BF among the top 6 pathways. Full information about the enriched pathways can be found at `http://xiangzhu.github.io/rss-gsea/`. Intersections of top pathways are visualized as UpSet plots [80].

Adult height [5]

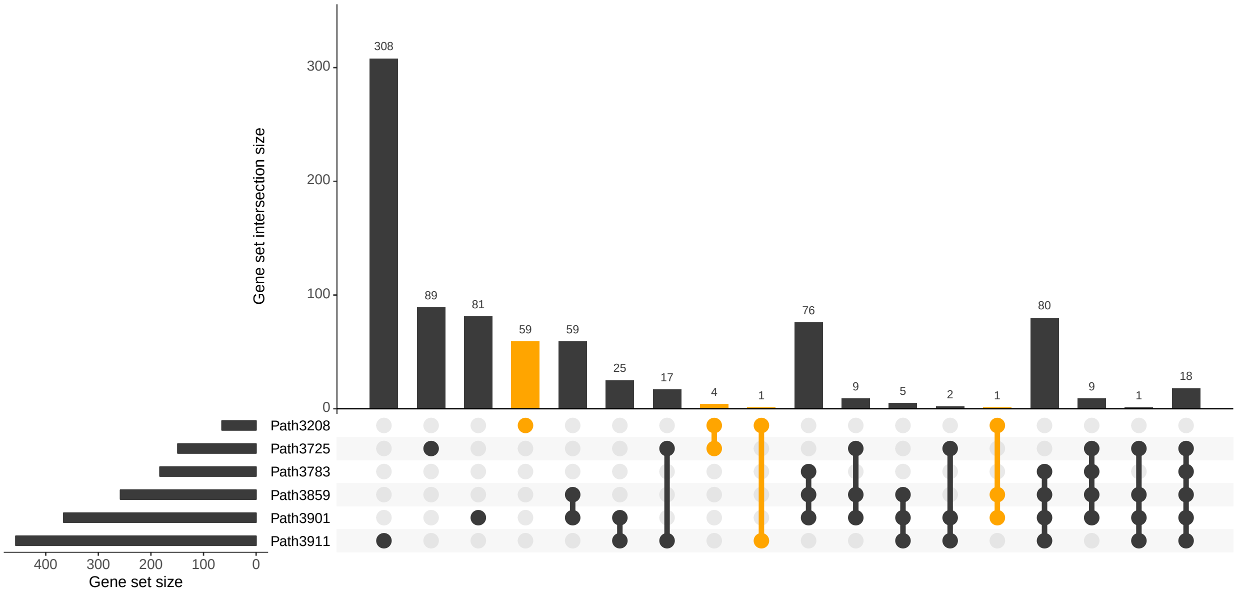

Body mass index [35]

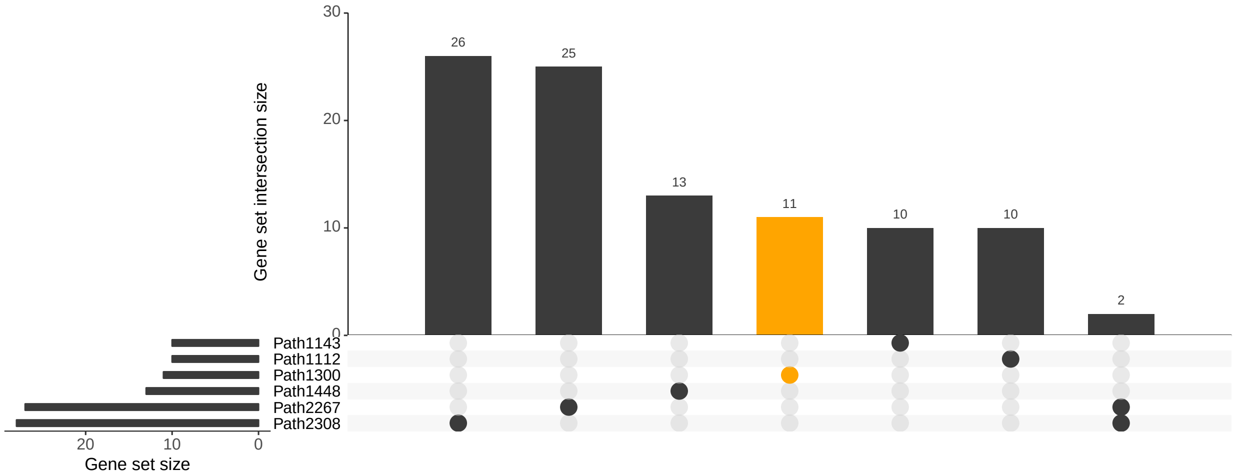

Waist-to-hip ratio adjusted for body mass index [36]

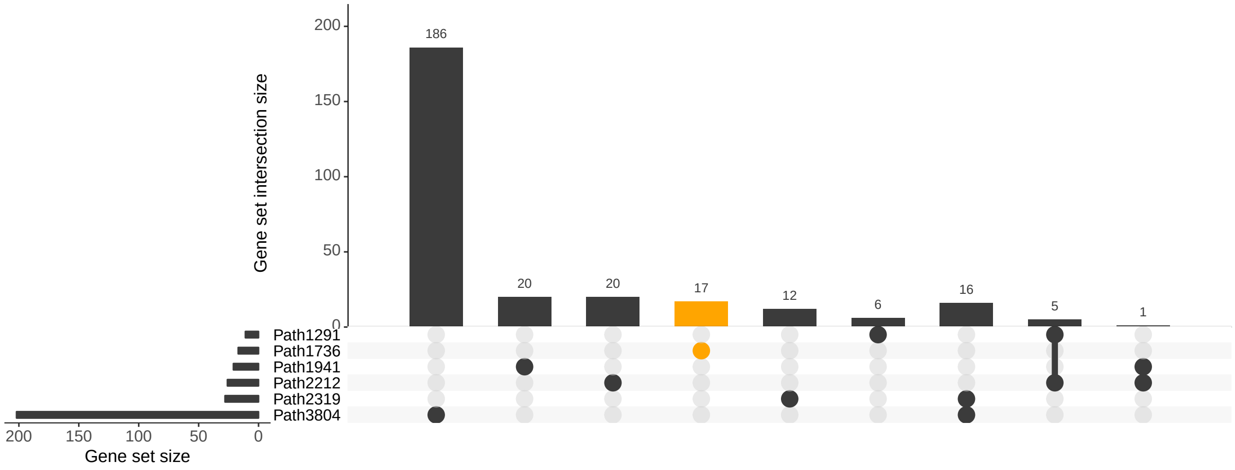

Amyotrophic lateral sclerosis [45]

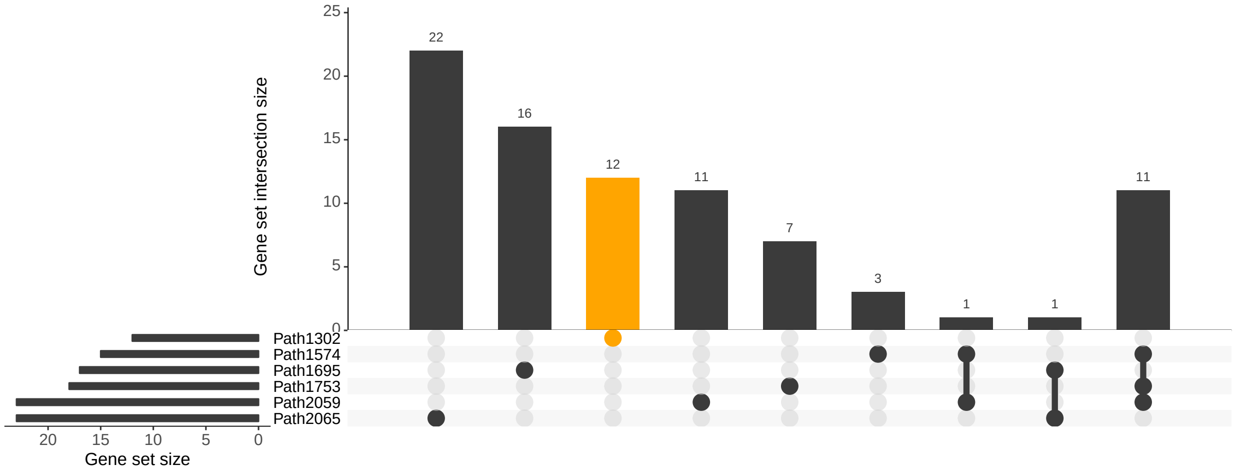

Mean cell haemoglobin concentration [46]

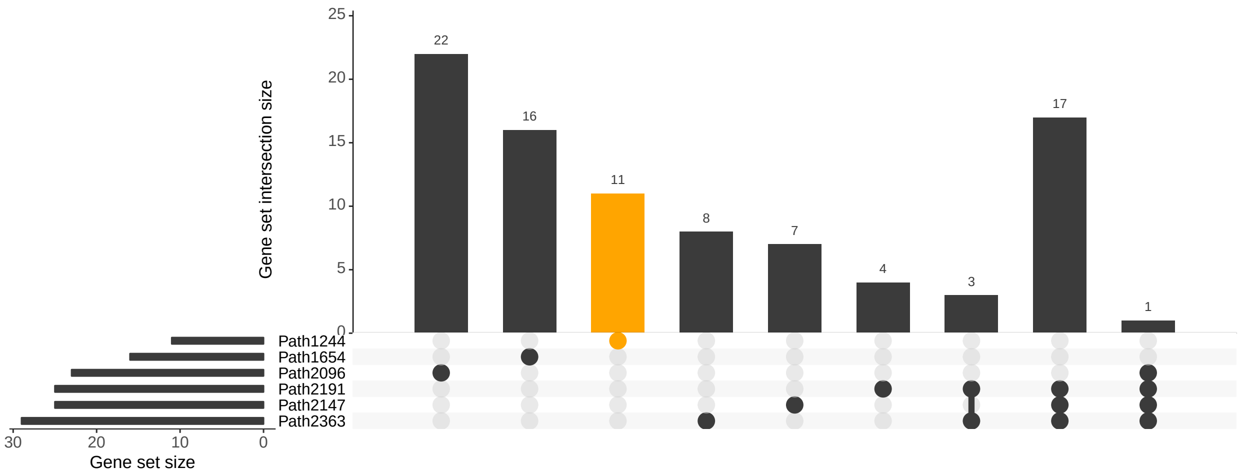

Packed cell volume [46]

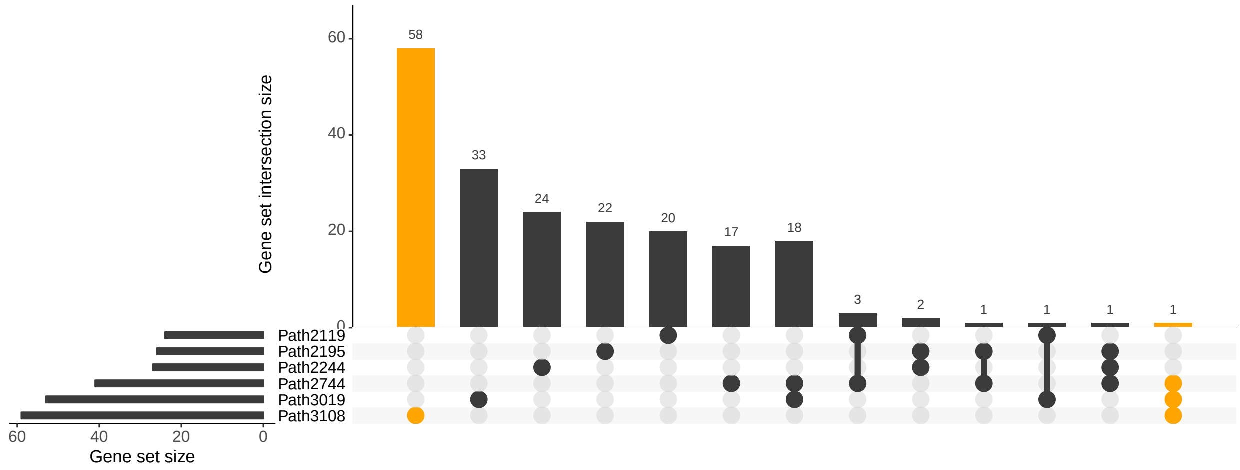

Alzheimer's disease [39]

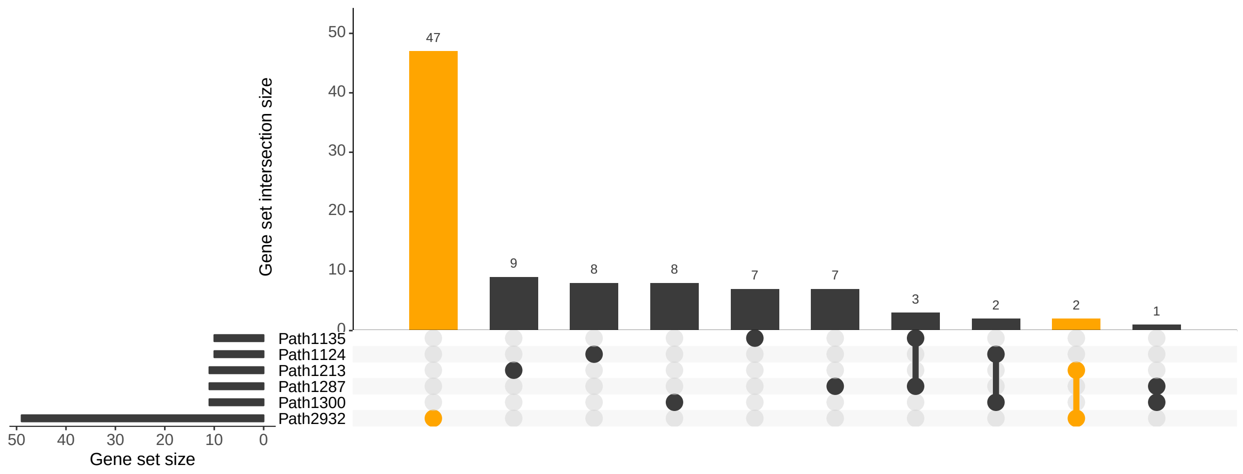

Heart rate [38]

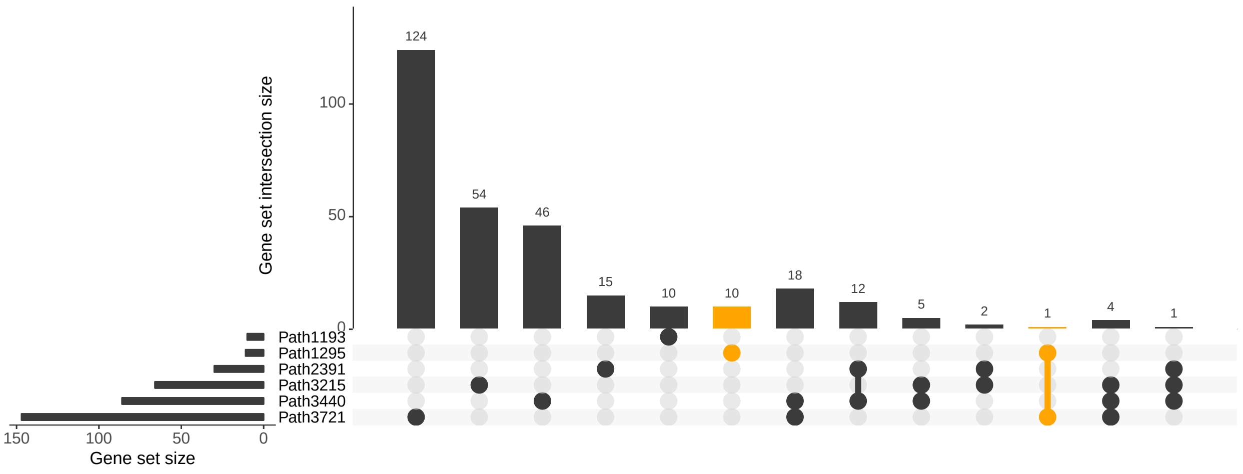

Coronary artery disease [18]

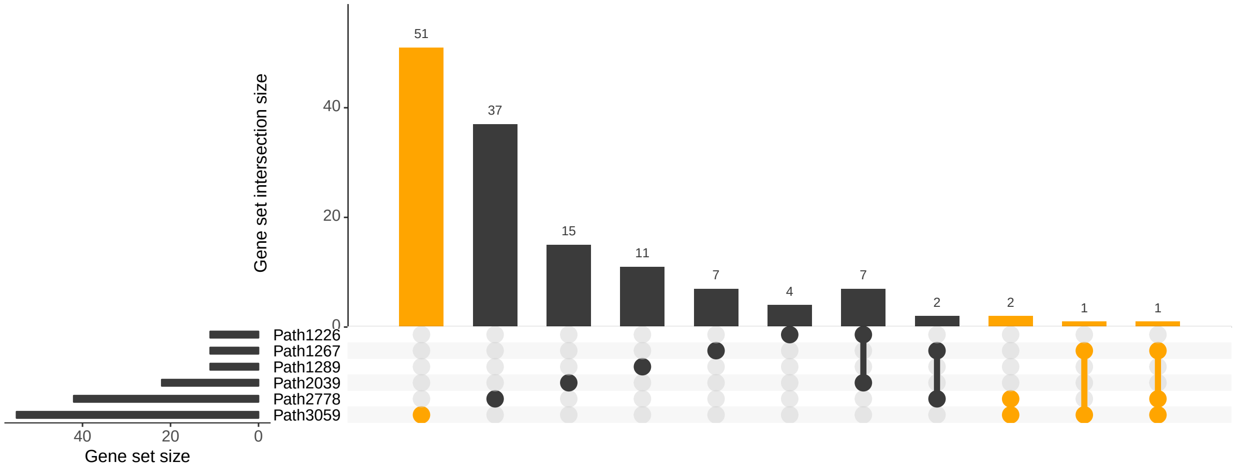

Myocardial infarction [18]

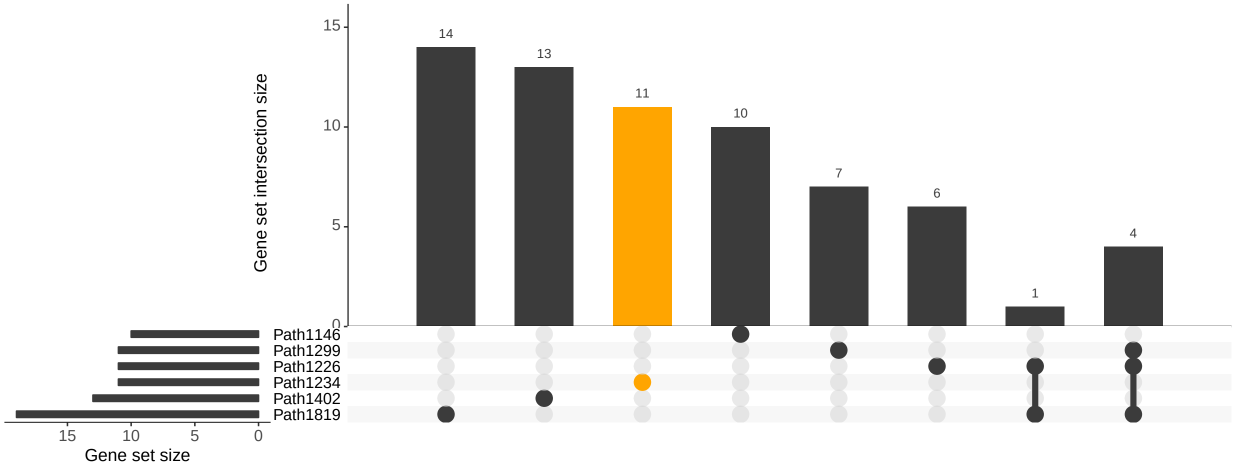

Serum urate concentrations [43]

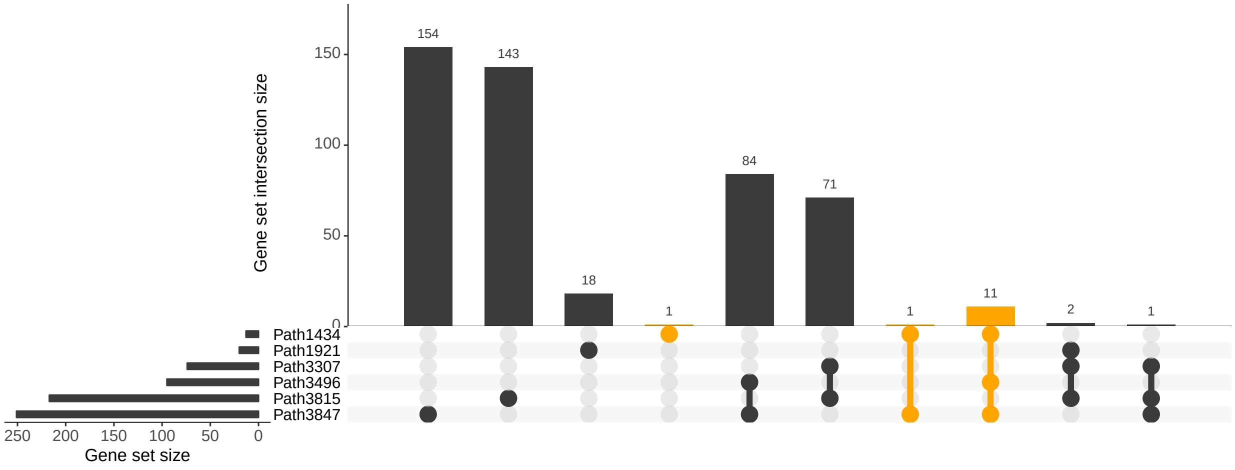

Gout [43]

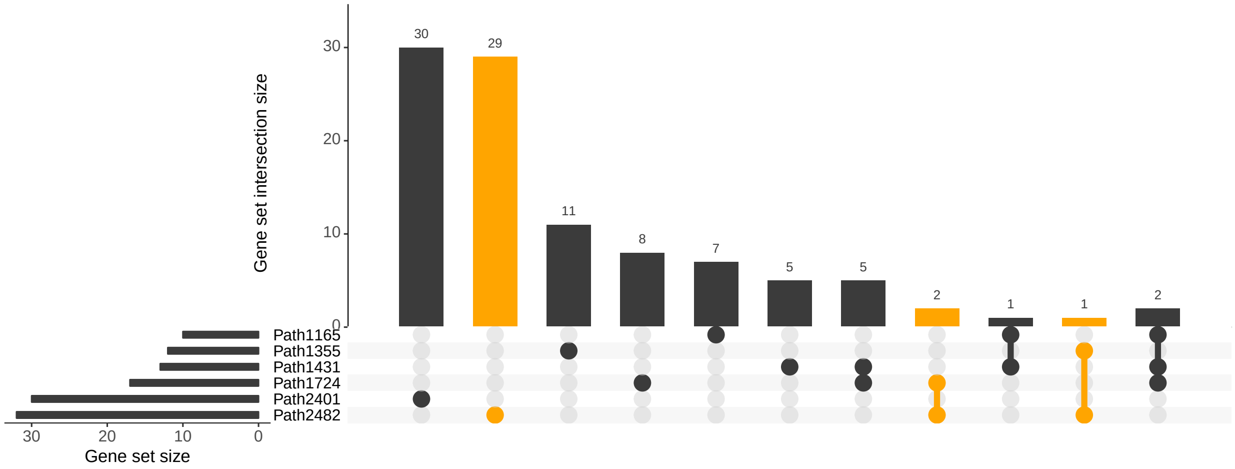

Total cholesterol [22]

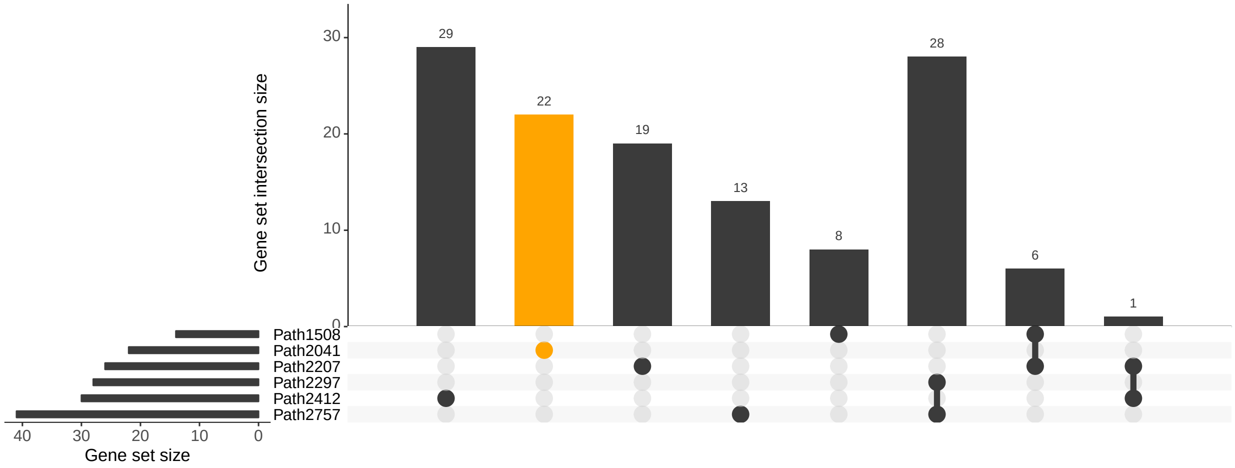

Triglycerides [22]

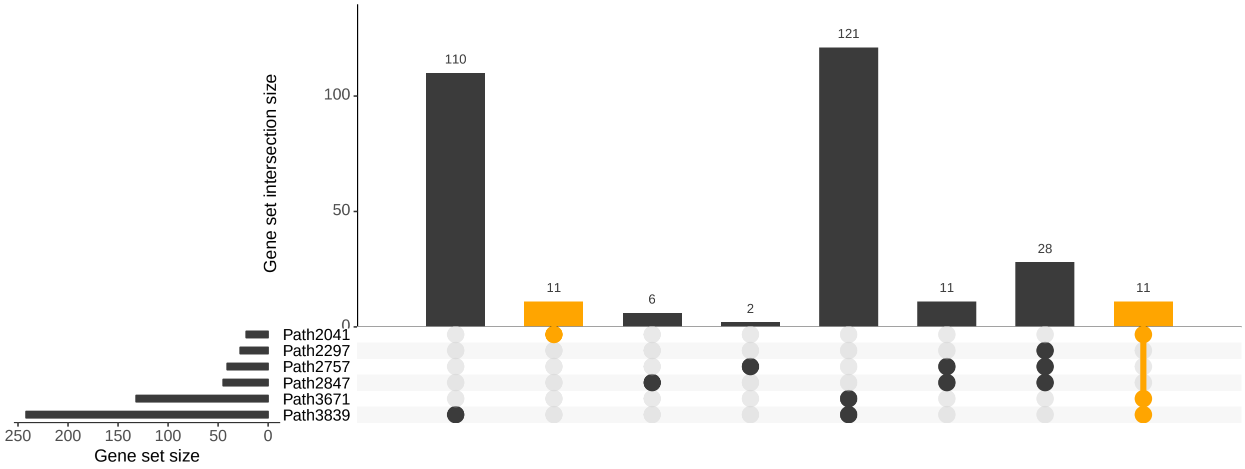

High-density lipoprotein [22]

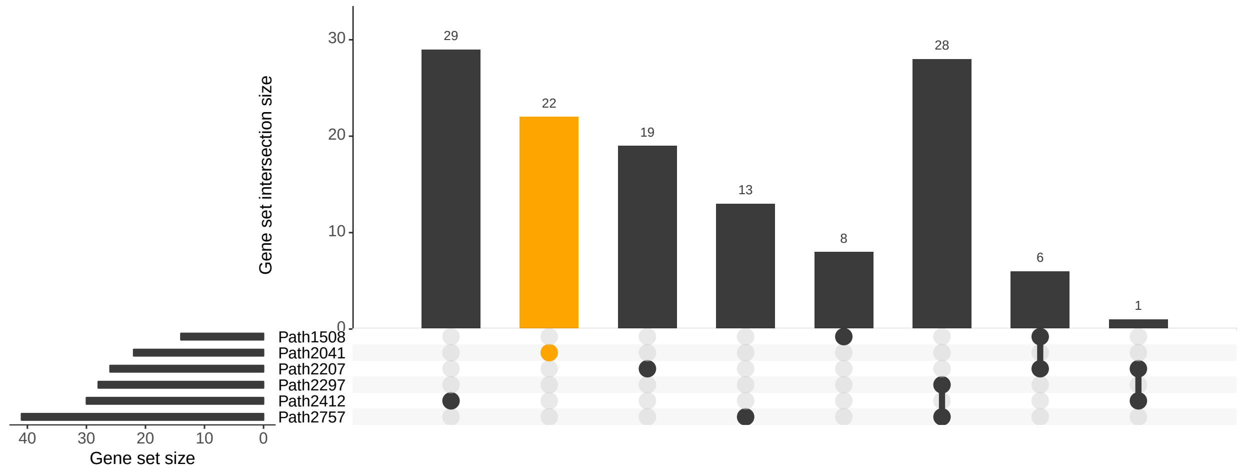

Low-density lipoprotein [22]

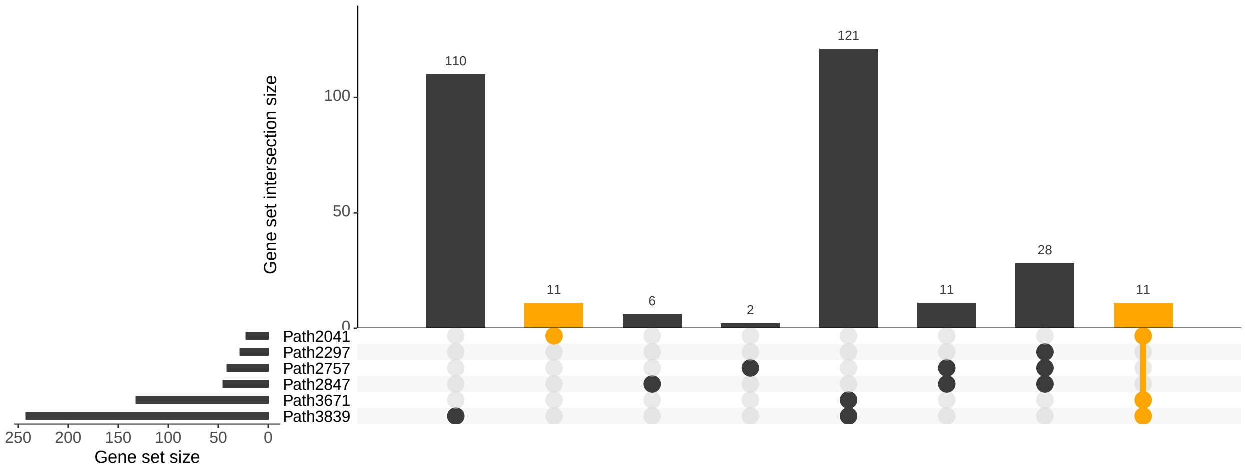

Depressive symptoms [40]

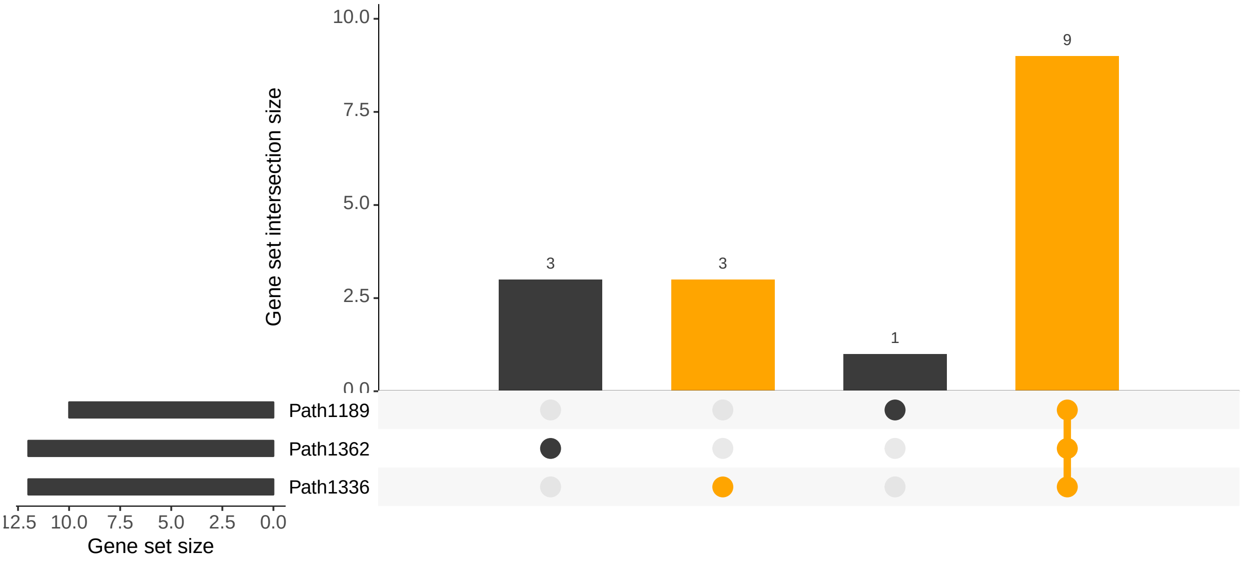

Neuroticism [40]

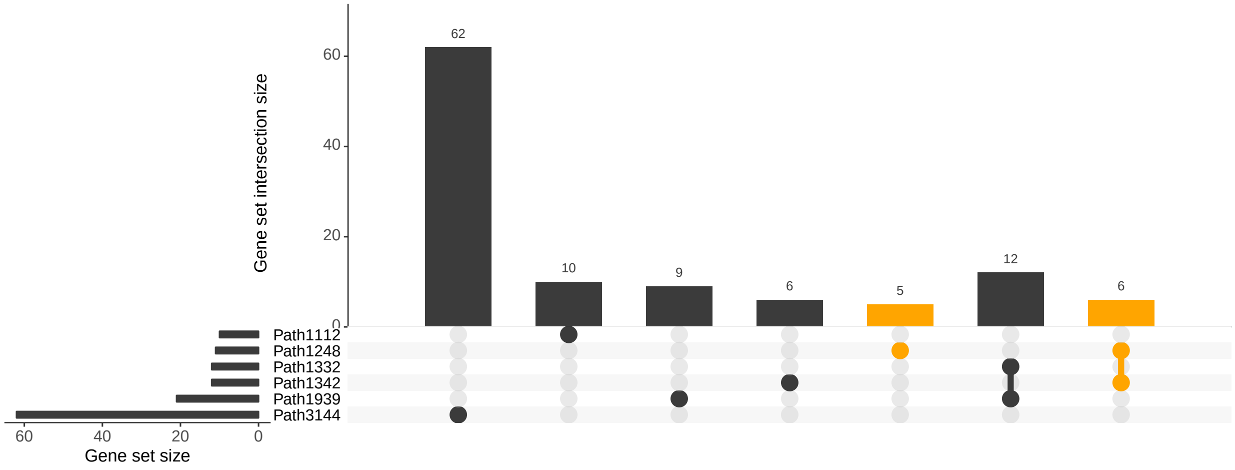

Schizophrenia [37]

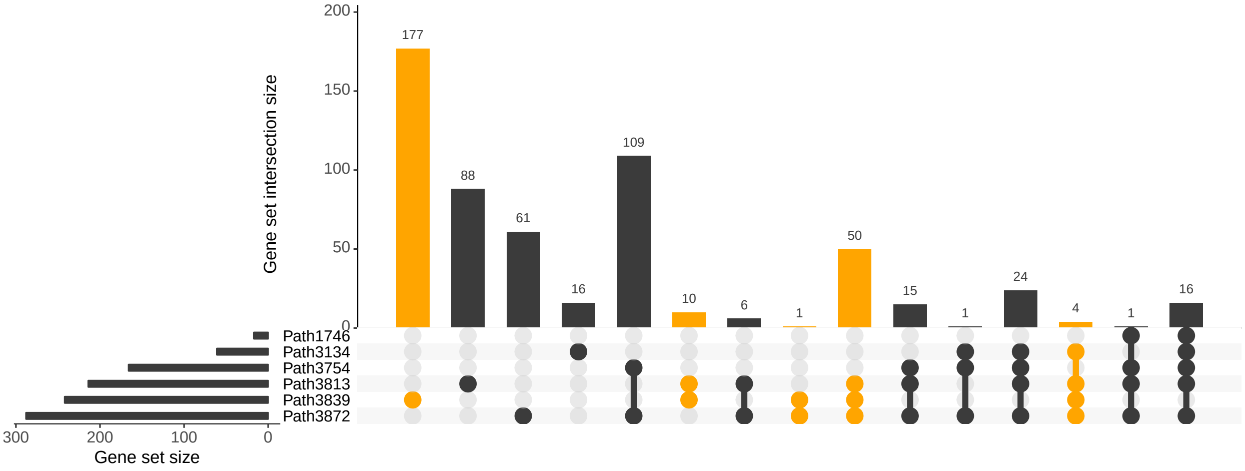

Rheumatoid arthritis [26]

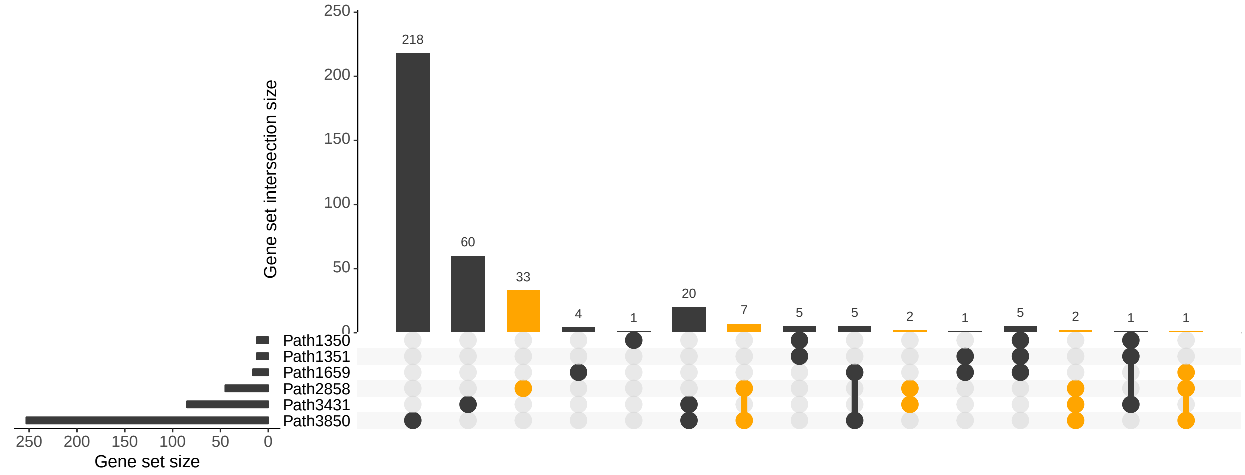

Fasting glucose levels [46]

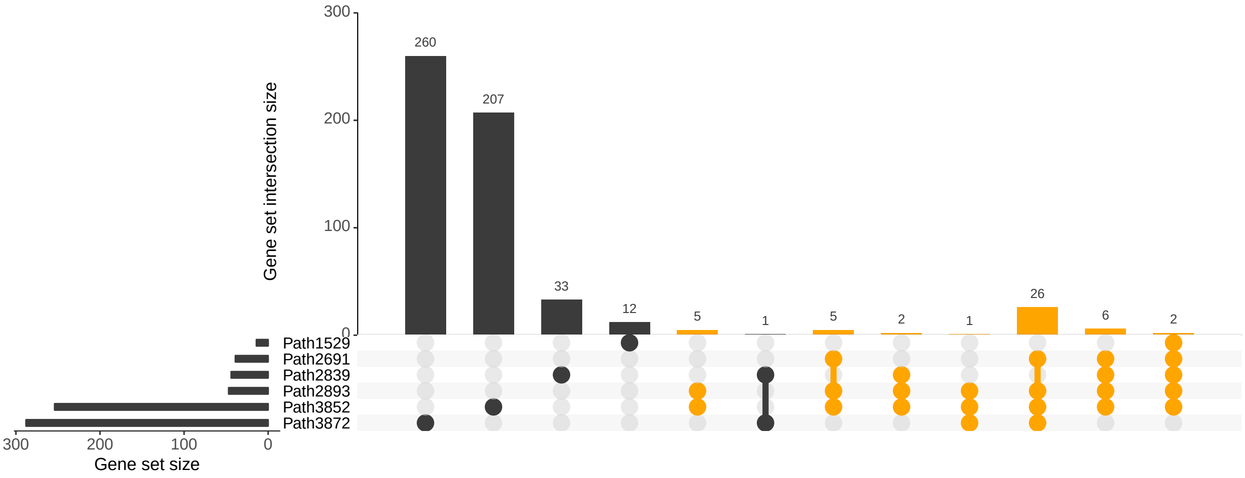

Fasting insulin levels [46]

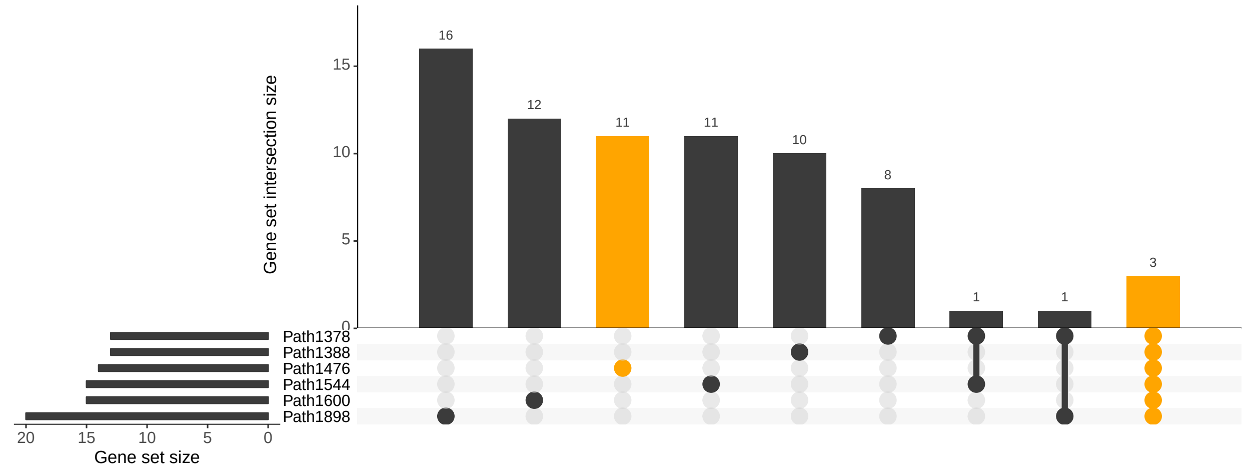

Haemoglobin [46]

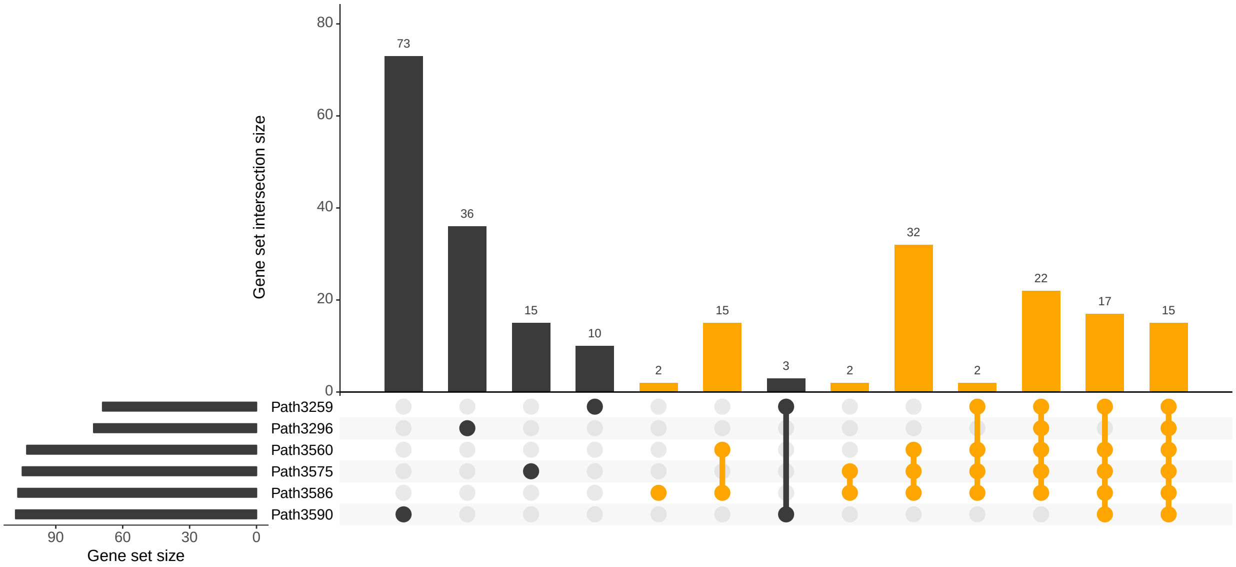

Red blood cell count [46]

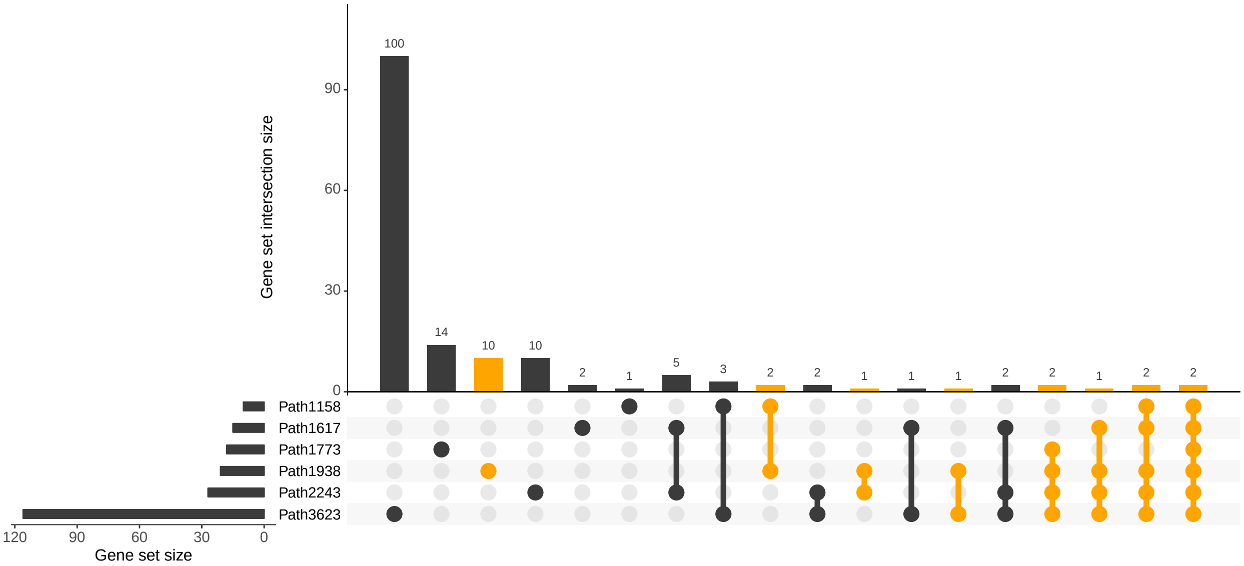

Mean cell haemoglobin [46]

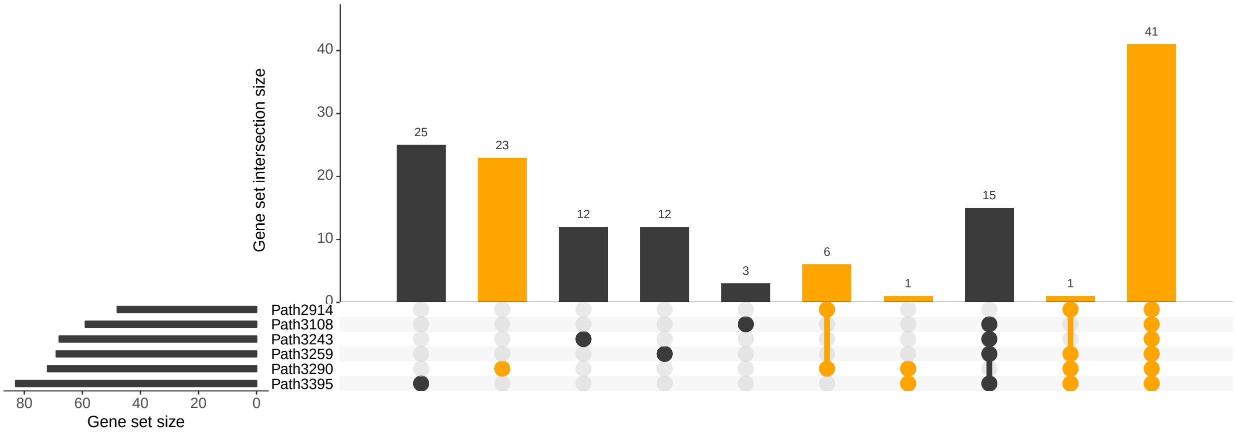

Mean cell volume [46]

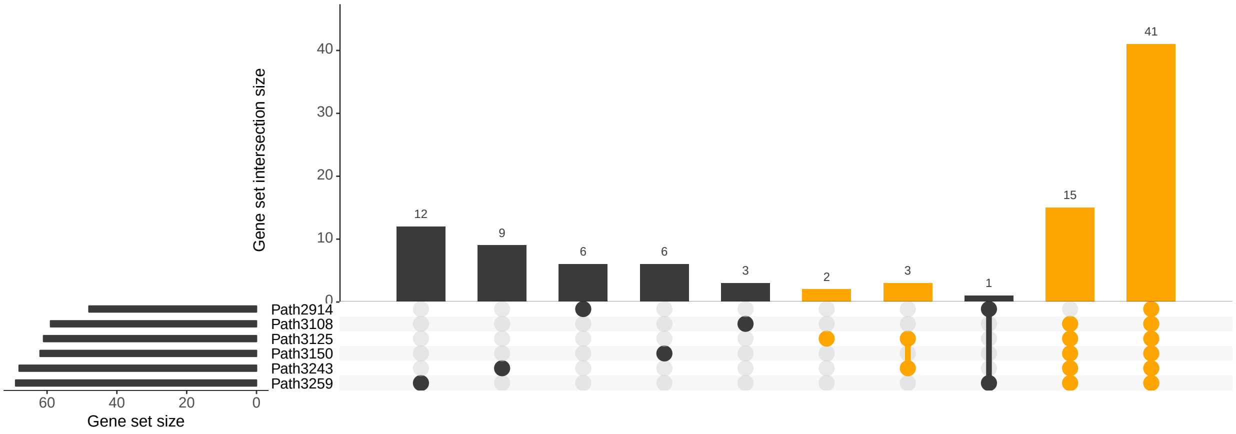

Age at natural menopause [42]

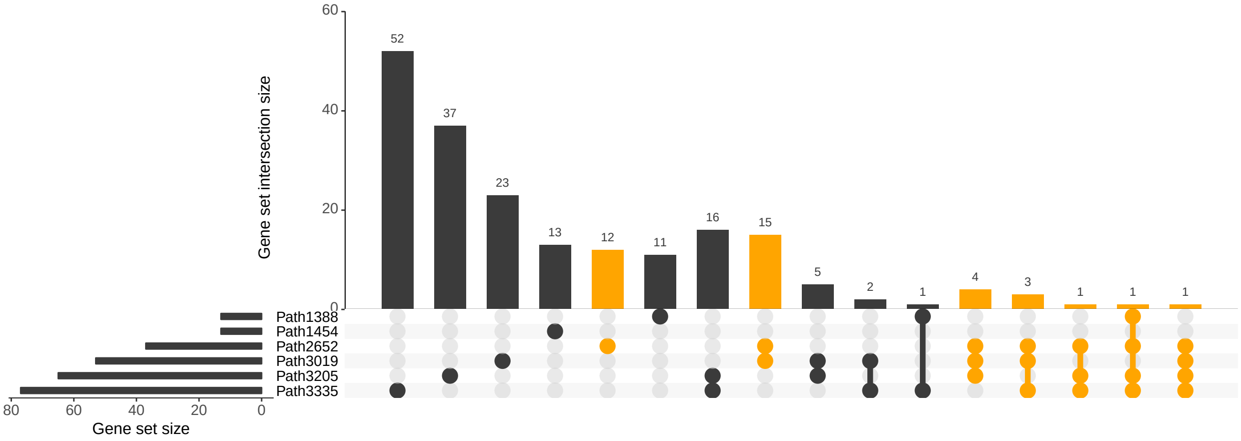

Inflammatory bowel disease [11]

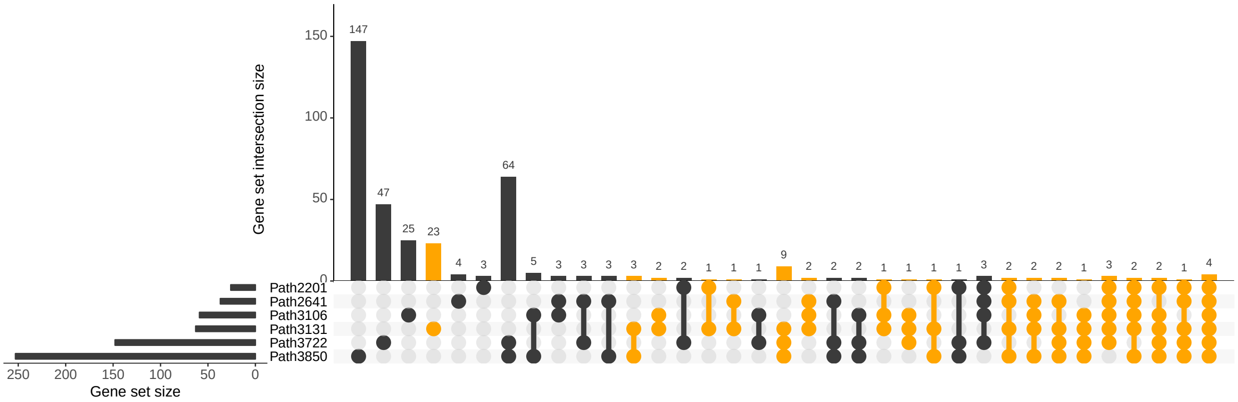

Crohn's disease [11]

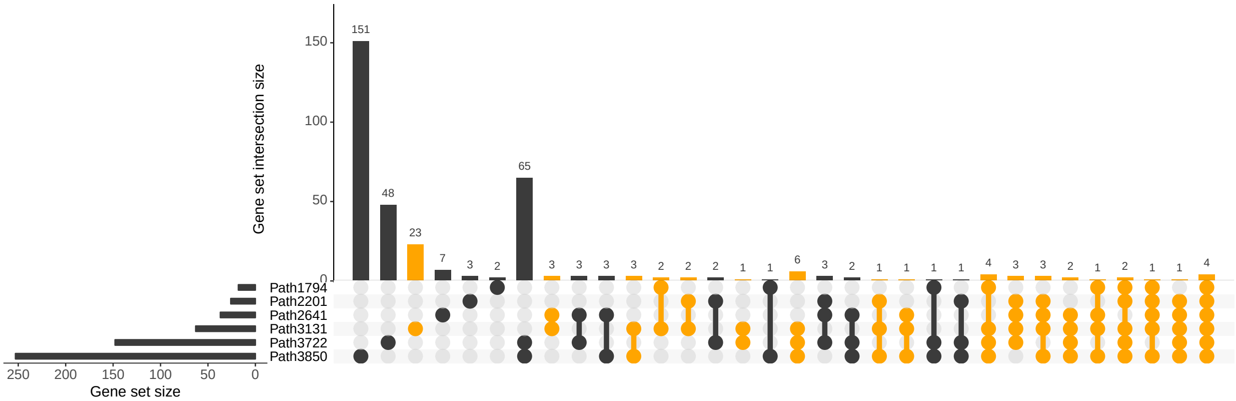

Ulcerative colitis [11]

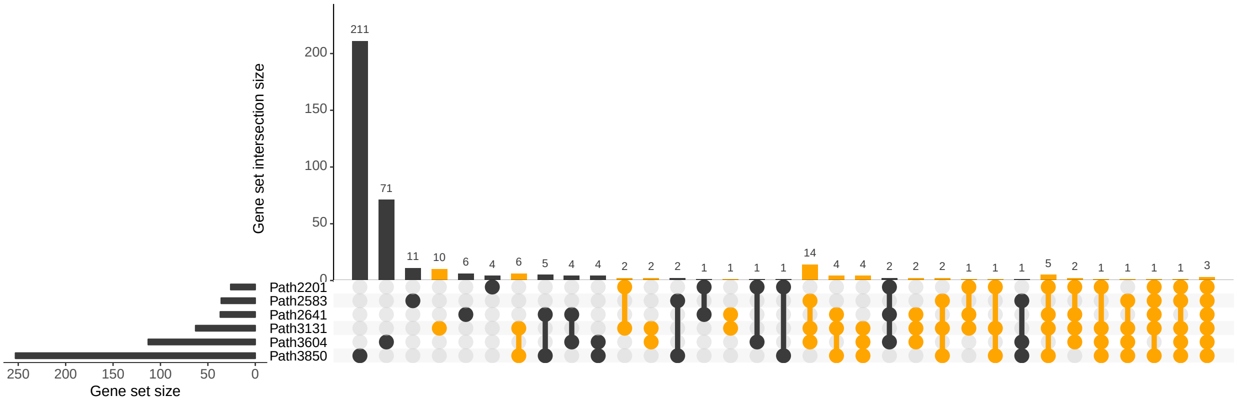

Type 2 diabetes [41]

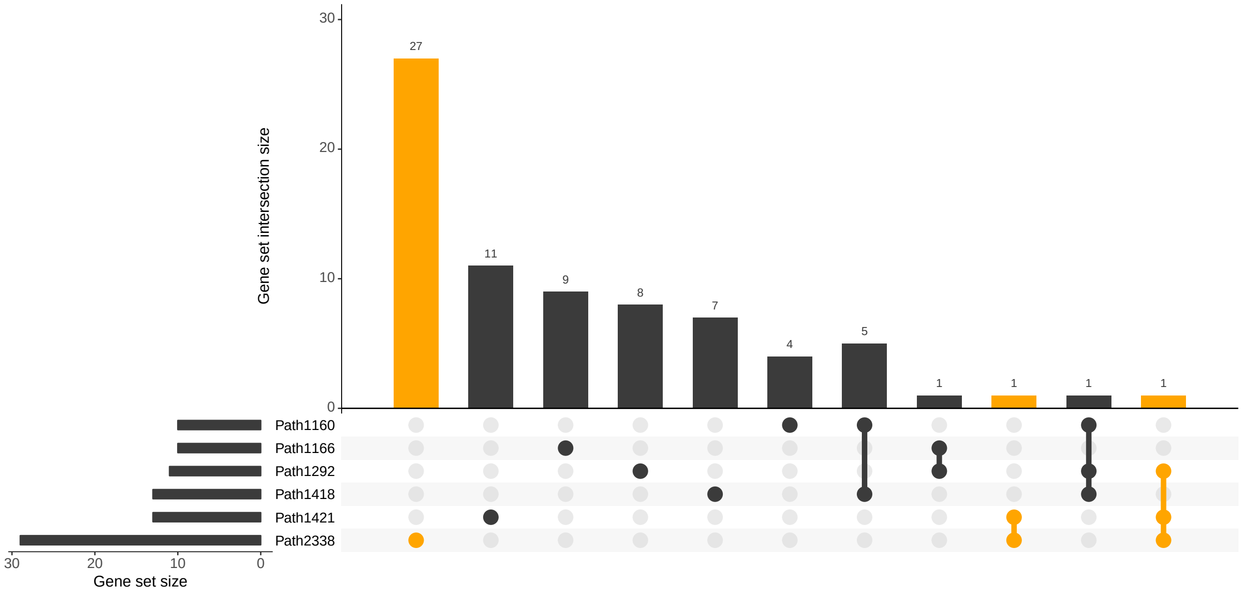

32. Supplementary Figure 24. Estimated genome-wide background parameter (θ_0) under the baseline (M_0) and enrichment (M_1) models across 31 traits. Each dot represents a trait, with the x-axis value being the posterior mean of θ_0 estimated under M_0 and a vertical point range indicating the (2.5, 50, 97.5)-percentile for posterior means of θ_0 across 100 top-ranked gene sets under M_1. The dashed line has slope one and intercept zero. The tight vertical point ranges demonstrate that RSS-E gives almost identical estimates of θ_0 in both baseline and enrichment analyses.

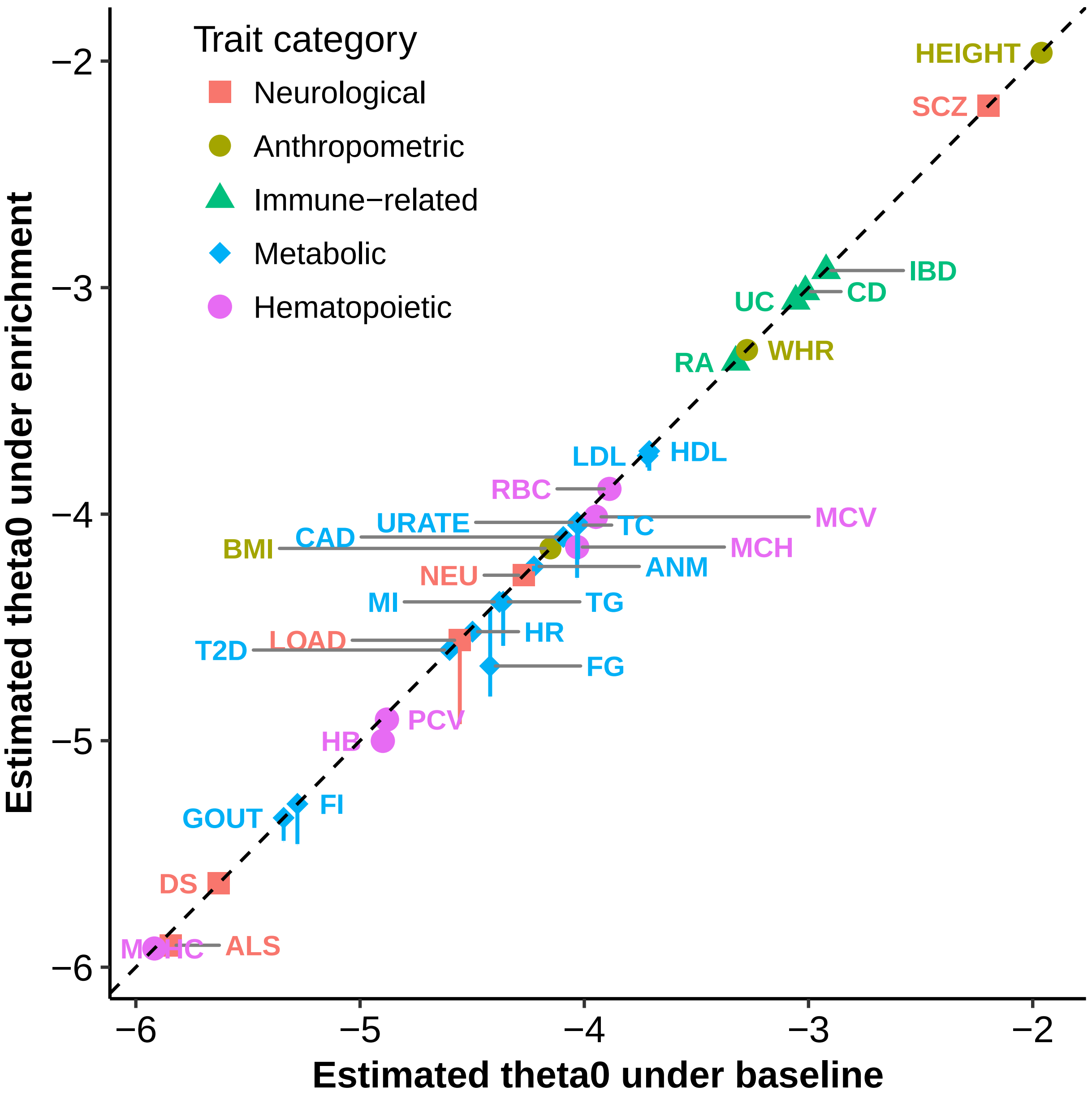

33. Supplementary Figure 25. Estimated enrichment parameter (θ) versus the background parameter (θ_0) under the enrichment model (M_1) across 31 traits. Each dot represents a trait, with horizontal and vertical point ranges indicating the (2.5, 50, 97.5)-percentiles for posterior means of θ_0 and θ across 100 top-ranked gene sets respectively. This plot demonstrates that RSS-E yields a positive estimate of enrichment parameter θ if the gene set is identified as enriched.

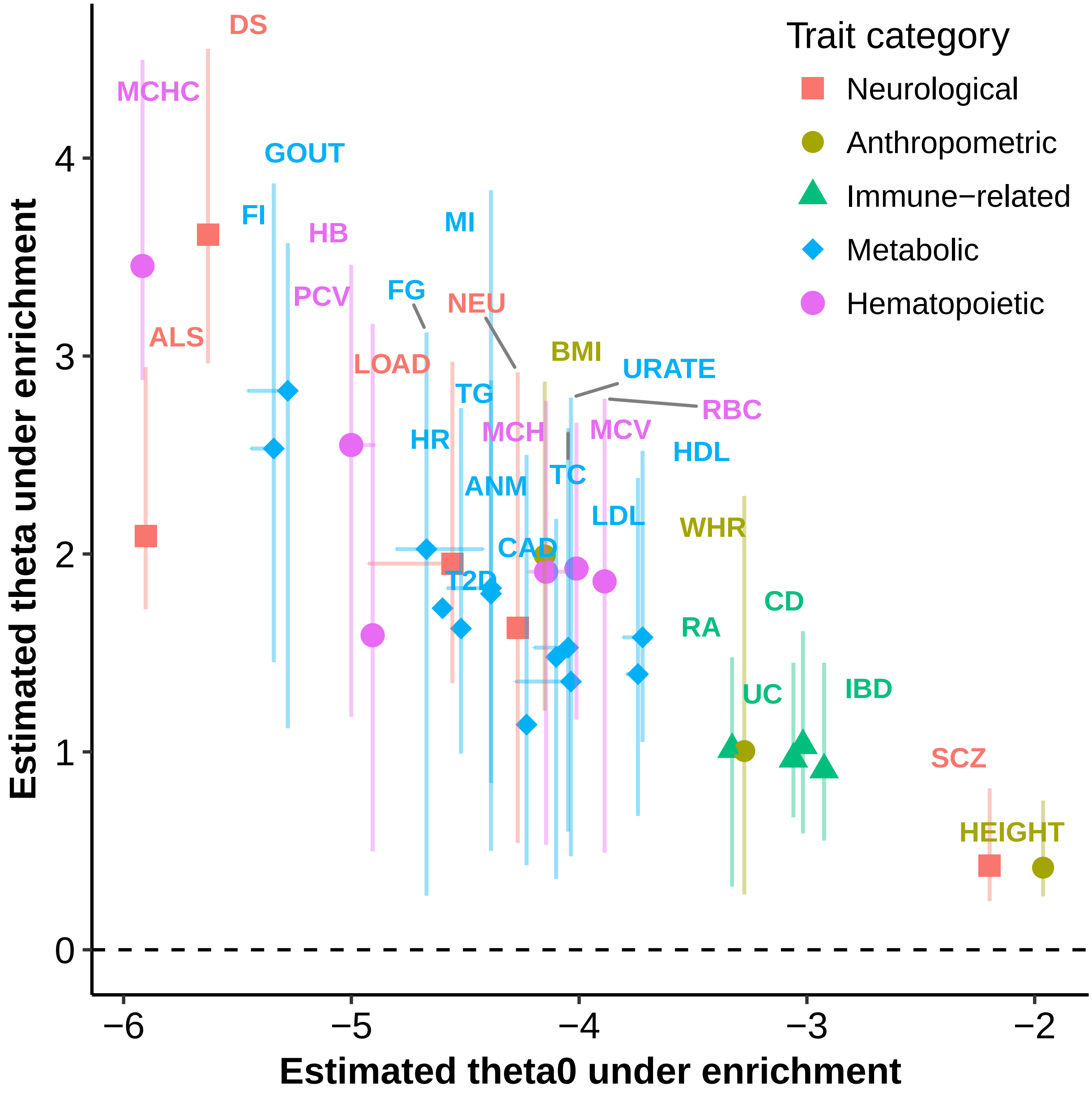

34. Supplementary Figure 26. Compare the number of trait-associated loci detected under the baseline hypothesis with the number of trait-associated loci detected under the enrichment hypothesis. The baseline hypothesis assumes that no pathways are enriched ($M_0 : \theta = 0$). The enrichment hypothesis assumes that a candidate pathway is enriched ($M_1 : \theta > 0$). For each trait, each dot corresponds to one of the top 10 most enriched pathways in Round 2 analyses (shown in **Supplementary Figure 17**). A positive value in x-axis indicates that more trait-associated loci ($P_1 > 0.9$) are identified under the enrichment hypothesis than under the null hypothesis. See **Supplementary Figure 10** for the definition of locus and P_1. Note that some dots are overlapped due to their similar values in x-axis.

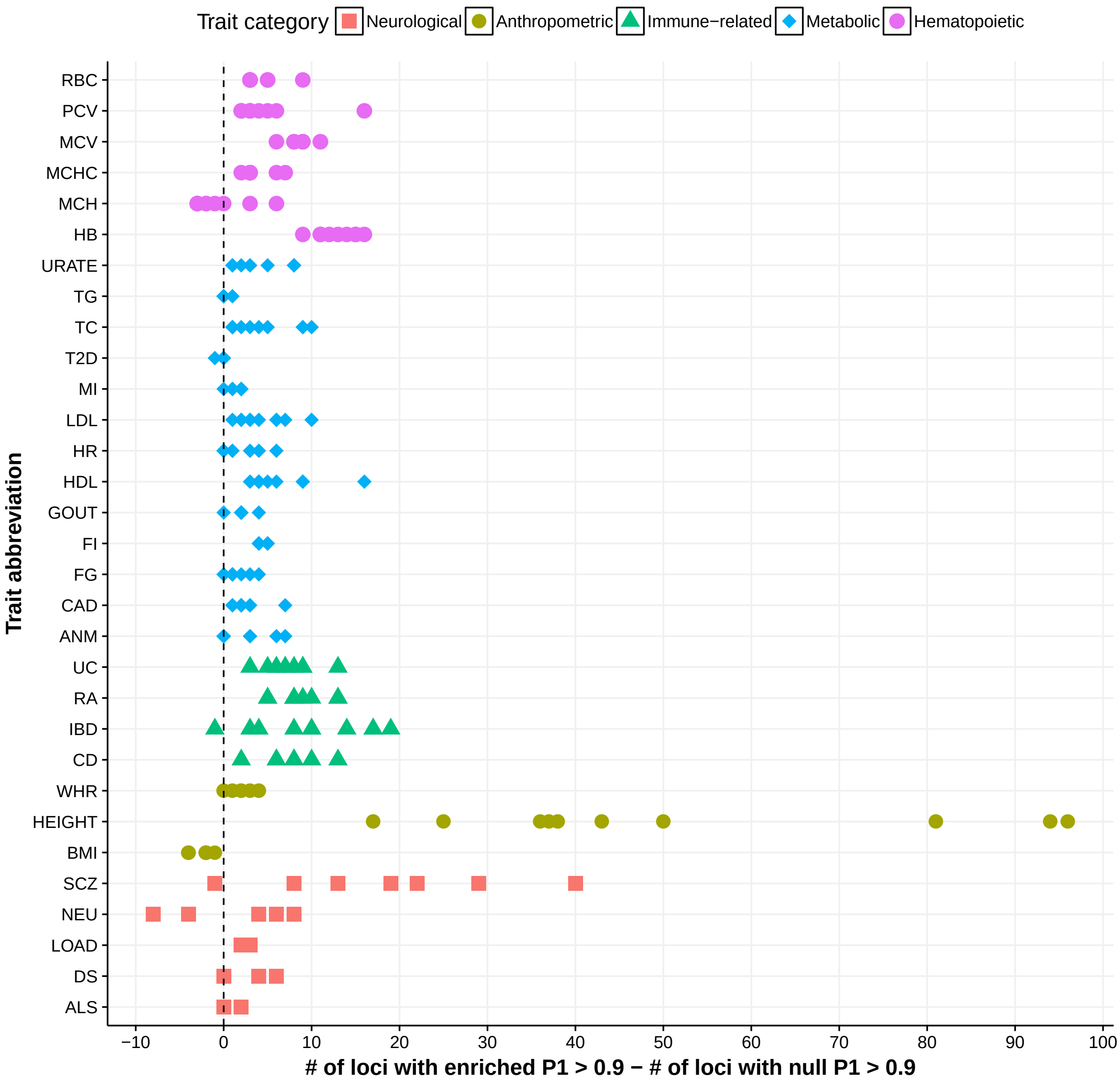

35. Supplementary Figure 27. Regional association plots of genes *LIPC* and *LPL* based on single-SNP summary data of low-density lipoprotein cholesterol levels. The “2010 data” were reported in [22]. The “2013 data” were reported in [24]. These regional plots are generated using the package `LocusZoom` [81].

(a) Regional association plot of *LIPC*.

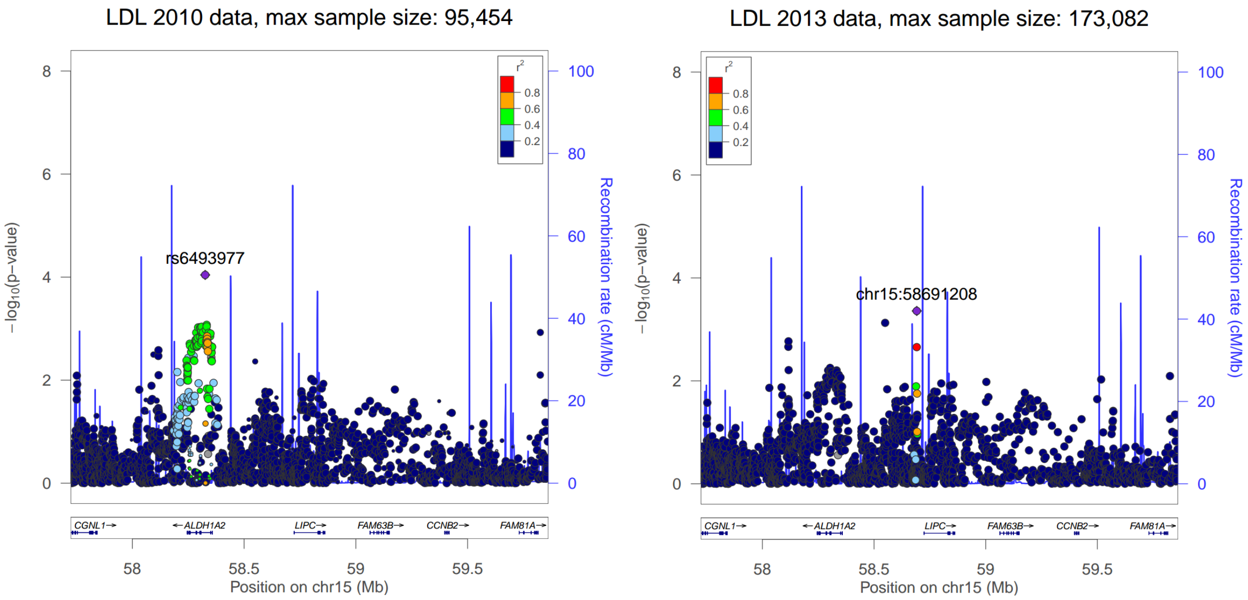

(b) Regional association plot of *LPL*.

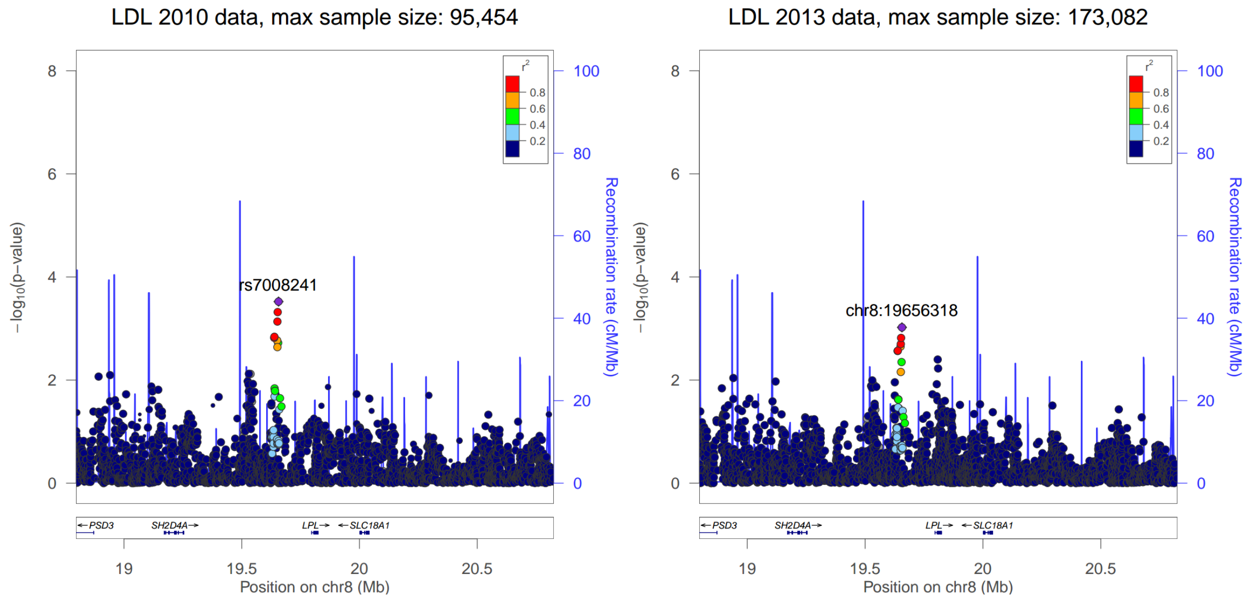

36. Supplementary Figure 28. Venn diagrams showing that three annotation methods yield different sets of genes for a given tissue. For each tissue, we use three complementary methods to define tissue-relevant genes based on GTEx RNA-seq data [34]. The first approach ("highly expressed", HE) uses the highest expressed genes in each tissue. The second approach ("selectively expressed", SE) uses a tissue-selectivity score [82] designed to identify genes that are much more strongly expressed in that tissue than in other tissues. The third approach ("distinctively expressed", DE) clusters the tissue samples and identifies genes that are most informative for distinguishing each cluster from others [79]. Note that the HE and SE methods define unique gene sets for a given tissue, whereas the DE method sometimes produce a gene set shared by multiple tissues. All tissue-based gene sets in the present study contain 100 member genes.

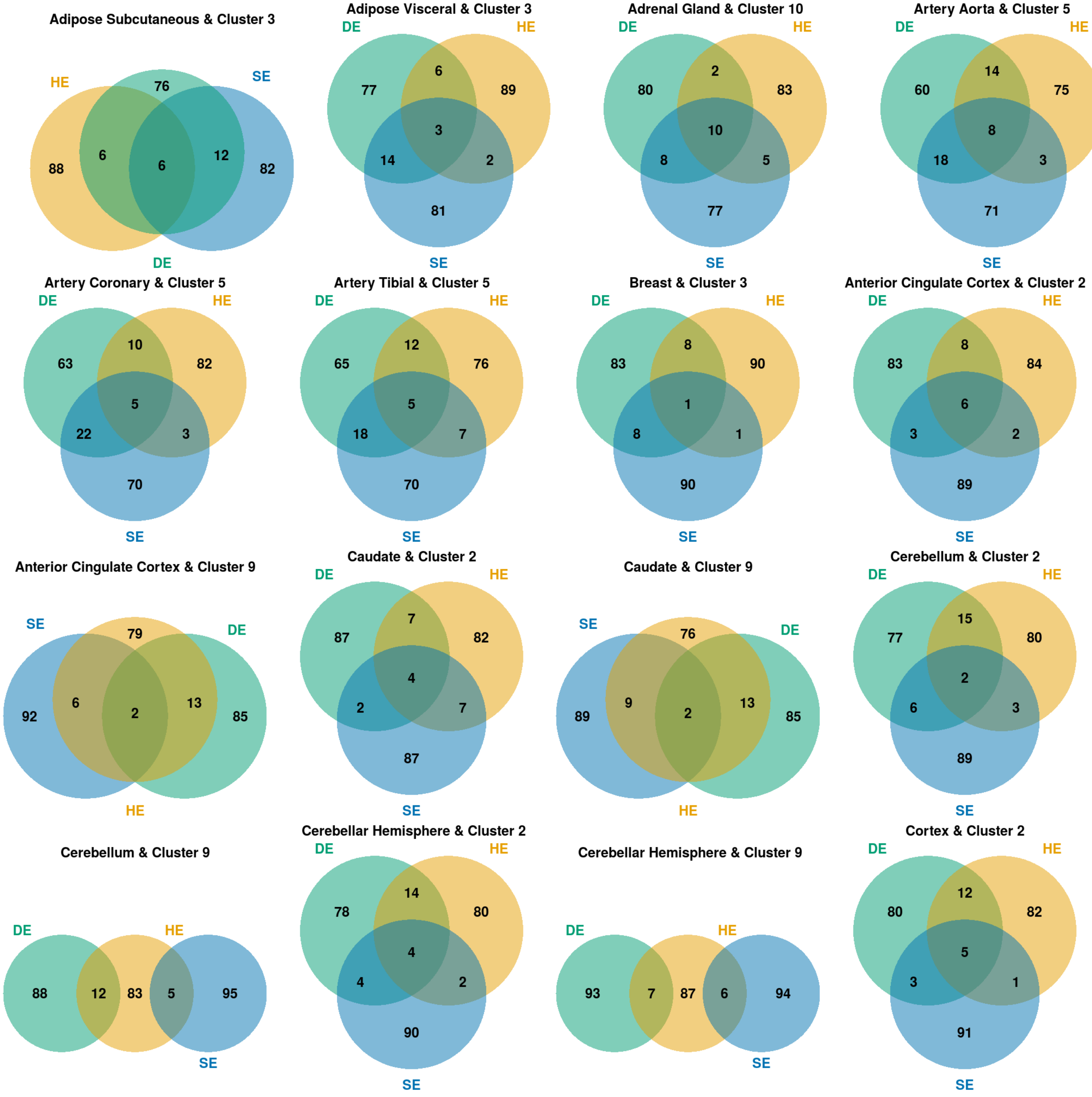

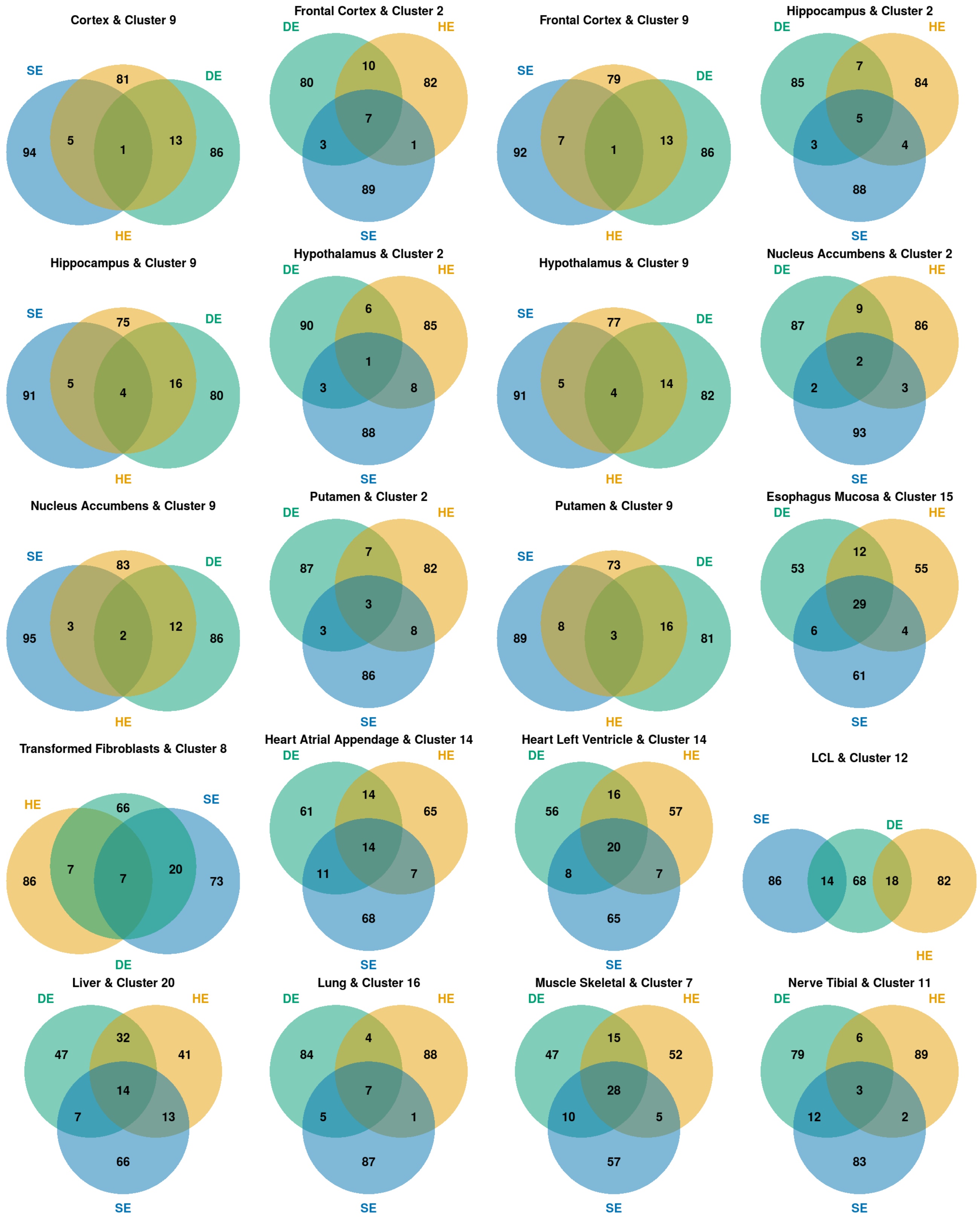
Cortex & Cluster 9
SE DE HE
81 94 5 1 13 86
Frontal Cortex & Cluster 2
DE HE SE
10 80 82 7 3 1 89
Frontal Cortex & Cluster 9
SE DE HE
79 92 7 1 13 86
Hippocampus & Cluster 2
DE HE SE
7 85 84 5 3 4 88
Hippocampus & Cluster 9
SE DE HE
75 91 5 4 16 80
Hypothalamus & Cluster 2
DE HE SE
6 90 85 1 3 8 88
Hypothalamus & Cluster 9
SE DE HE
77 91 5 4 14 82
Nucleus Accumbens & Cluster 2
DE HE SE
9 87 86 2 2 3 93
Nucleus Accumbens & Cluster 9
SE DE HE
83 95 3 2 12 86
Putamen & Cluster 2
DE HE SE
7 87 82 3 3 8 86
Putamen & Cluster 9
SE DE HE
73 89 8 3 16 81
Esophagus Mucosa & Cluster 15
DE HE SE
12 53 55 29 6 4 61
Transformed Fibroblasts & Cluster 8
HE SE DE
66 86 7 7 20 73
Heart Atrial Appendage & Cluster 14
DE HE SE
14 61 65 14 11 7 68
Heart Left Ventricle & Cluster 14
DE HE SE
16 56 57 20 8 7 65
LCL & Cluster 12
SE DE HE
86 14 68 18 82
Liver & Cluster 20
DE HE SE
32 47 41 14 7 13 66
Lung & Cluster 16
DE HE SE
4 84 88 7 5 1 87
Muscle Skeletal & Cluster 7
DE HE SE
15 47 52 28 10 5 57
Nerve Tibial & Cluster 11
DE HE SE
6 79 89 3 12 2 83

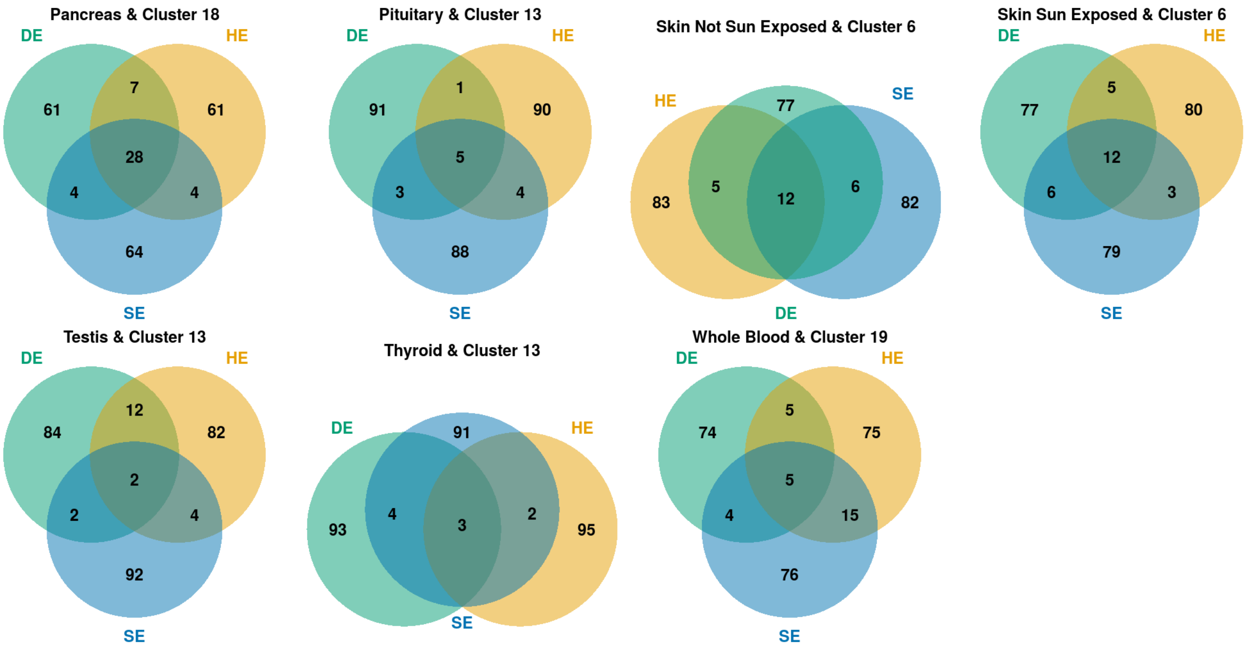
Pancreas & Cluster 18
DE
HE
SE
61
7
61
28
4
4
64
Pituitary & Cluster 13
DE
HE
SE
91
1
90
5
3
4
88
Skin Not Sun Exposed & Cluster 6
HE
SE
DE
77
83
5
12
6
82
Skin Sun Exposed & Cluster 6
DE
HE
SE
77
5
80
12
6
3
79
Testis & Cluster 13
DE
HE
SE
84
12
82
2
2
4
92
Thyroid & Cluster 13
DE
HE
SE
91
93
4
3
2
95
Whole Blood & Cluster 19
DE
HE
SE
74
5
75
5
4
15
76

37. Supplementary Figure 29. Regional association plots of genes *C2orf16* and *GCKR* based on single-SNP summary data of total cholesterol and triglycerides levels. The “2010 data” were reported in [22]. The “2013 data” were reported in [24]. These regional plots are generated using the package `LocusZoom` [81].

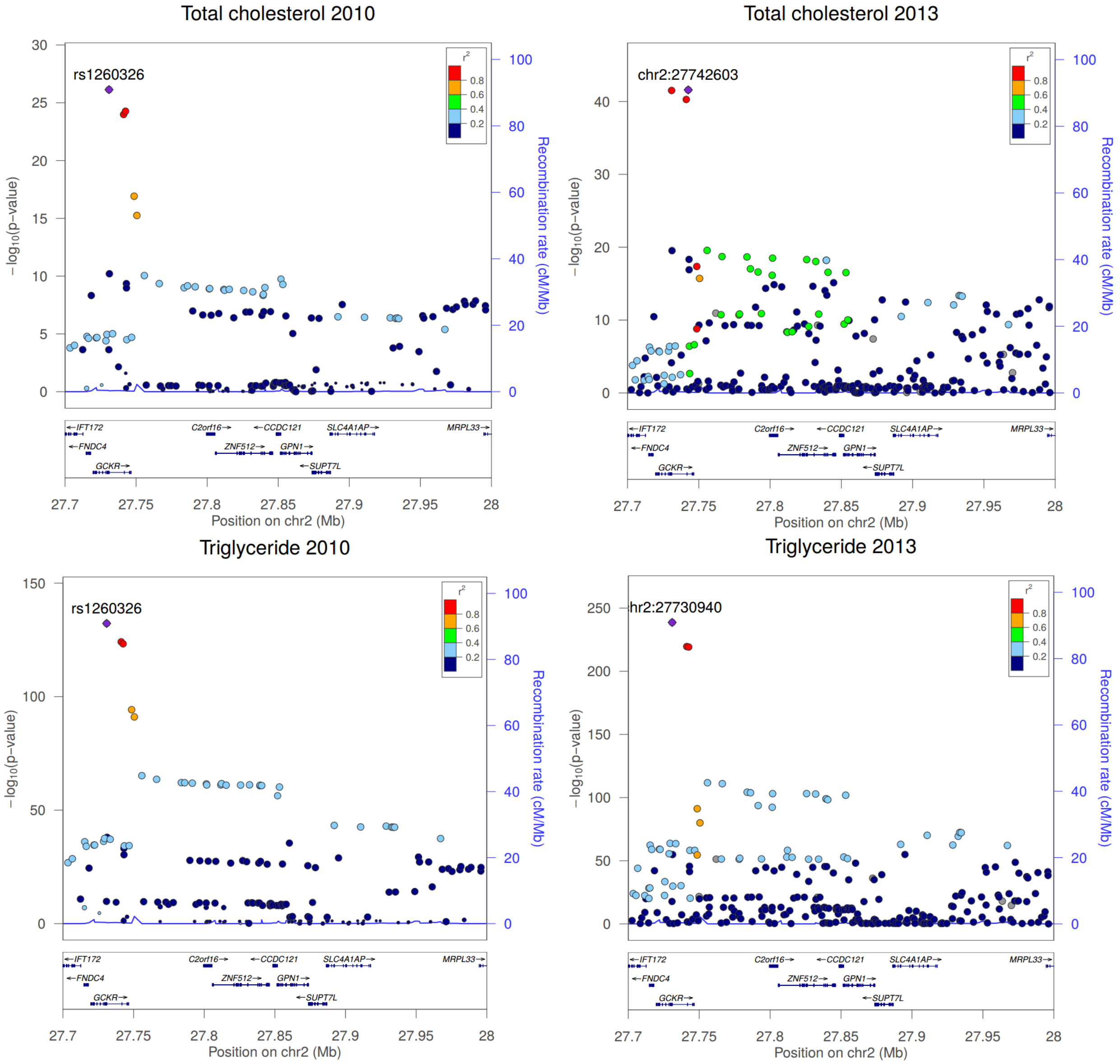

38. Supplementary Figure 30. Compare analyses of individual-level data [2] with analyses of summary-level data. Simulation details of Panel **(a)** are provided in **Supplementary Figure 6**. Simulation details of Panel **(b)** are provided in **Supplementary Figure 1**. For each simulated dataset, `varbvs` analyzes individual-level data and `rss-e` analyzes summary-level data. Note that `rss-e (original)` (shown in blue) is included here merely for testing purpose; `rss-e (squarem)` (shown in green) is the **only** algorithm used to generate results for the present study.

(a) Type 1 error rates of `varbvs` (orange), `rss-e (original)` (blue) and `rss-e (squarem)` (green) in baseline simulations (see **Supplementary Figure 6**). For each simulated dataset, a type 1 error is made if the enrichment Bayes factor (BF) is greater than the given cutoff for BF.

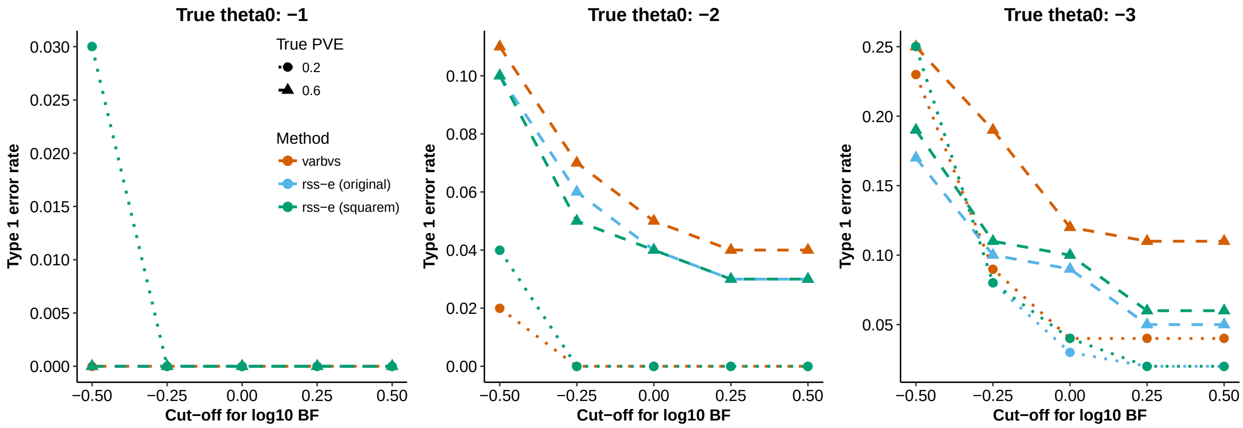

(b) Power of `varbvs` (orange), `rss-e (original)` (blue) and `rss-e (squarem)` (green) in enrichment simulations (see **Supplementary Figure 1**). For each scenario, the power is computed as the fraction of datasets whose enrichment BFs are greater than the given cutoff for BF.

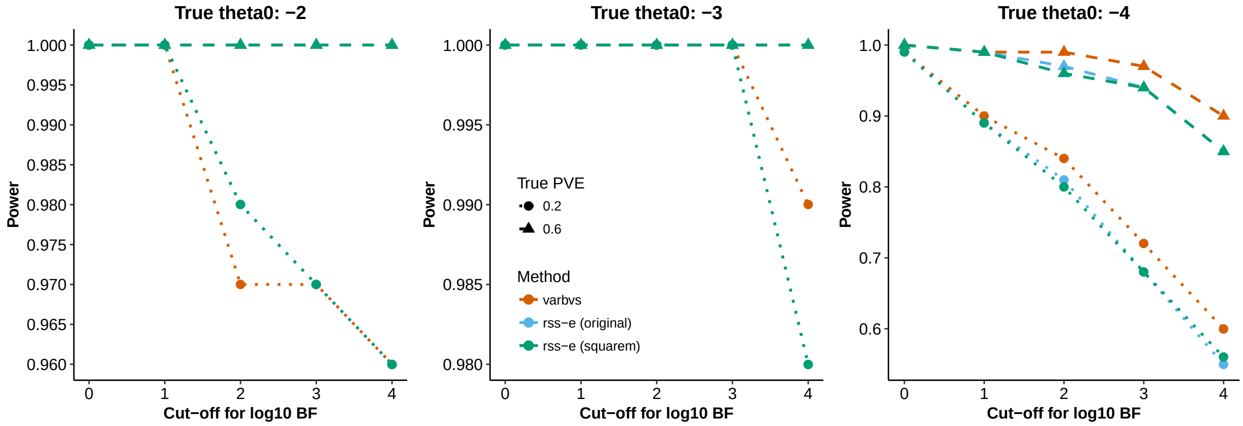

References.

[1] Zhu, X. & Stephens, M. Bayesian large-scale multiple regression with summary statistics from genome-wide association studies. *Annals of Applied Statistics* **11**, 1561–1592 (2017).

[2] Carbonetto, P. & Stephens, M. Integrated enrichment analysis of variants and pathways in genome-wide association studies indicates central role for IL-2 signaling genes in type 1 diabetes, and cytokine signaling genes in Crohn's disease. *PLoS Genetics* **9**, e1003770 (2013).

[3] Carbonetto, P. & Stephens, M. Scalable variational inference for Bayesian variable selection in regression, and its accuracy in genetic association studies. *Bayesian Analysis* **7**, 73–108 (2012).

[4] Varadhan, R. & Roland, C. Simple and globally convergent methods for accelerating the convergence of any EM algorithm. *Scandinavian Journal of Statistics* **35**, 335–353 (2008).

[5] Wood, A. R. *et al.* Defining the role of common variation in the genomic and biological architecture of adult human height. *Nature Genetics* **46**, 1173–1186 (2014).

[6] Vega, R. B. *et al.* Histone deacetylase 4 controls chondrocyte hypertrophy during skeletogenesis. *Cell* **119**, 555–566 (2004).

[7] Obri, A., Makinistoglu, M. P., Zhang, H. & Karsenty, G. HDAC4 integrates PTH and sympathetic signaling in osteoblasts. *The Journal of Cell Biology* **205**, 771–780 (2014).

[8] Cheloha, R. W., Gellman, S. H., Vilardaga, J.-P. & Gardella, T. J. PTH receptor-1 signalling – mechanistic insights and therapeutic prospects. *Nature Reviews Endocrinology* **11**, 712–724 (2015).

[9] Su, N., Jin, M. & Chen, L. Role of FGF/FGFR signaling in skeletal development and homeostasis: learning from mouse models. *Bone Research* **2**, 14003 (2014).

[10] Tang, S. Y., Herber, R.-P., Ho, S. P. & Alliston, T. Matrix metalloproteinase–13 is required for osteocytic perilacunar remodeling and maintains bone fracture resistance. *Journal of Bone and Mineral Research* **27**, 1936–1950 (2012).

[11] Liu, J. Z. *et al.* Association analyses identify 38 susceptibility loci for inflammatory bowel disease and highlight shared genetic risk across populations. *Nature Genetics* **47**, 979–986 (2015).

[12] Steinberg, M. W. *et al.* A crucial role for HVEM and BTLA in preventing intestinal inflammation. *Journal of Experimental Medicine* **205**, 1463–1476 (2008).

[13] Dubois, P. C. *et al.* Multiple common variants for celiac disease influencing immune gene expression. *Nature Genetics* **42**, 295–302 (2010).

[14] Anderson, C. A. *et al.* Meta-analysis identifies 29 additional ulcerative colitis risk loci, increasing the number of confirmed associations to 47. *Nature Genetics* **43**, 246–252 (2011).

[15] Jostins, L. *et al.* Host-microbe interactions have shaped the genetic architecture of inflammatory bowel disease. *Nature* **491**, 119–124 (2012).

[16] Strasser, A., Jost, P. J. & Nagata, S. The many roles of FAS receptor signaling in the immune system. *Immunity* **30**, 180–192 (2009).

[17] Neurath, M. F. Cytokines in inflammatory bowel disease. *Nature Reviews Immunology* **14**, 329–342 (2014).

[18] Nikpay, M. *et al.* A comprehensive 1000 genomes–based genome-wide association meta-analysis of coronary artery disease. *Nature Genetics* **47**, 1121–1130 (2015).

[19] Susan-Resiga, D. *et al.* Furin is the major processing enzyme of the cardiac-specific growth factor bone morphogenetic protein 10. *Journal of Biological Chemistry* **286**, 22785–22794 (2011).

[20] International Consortium for Blood Pressure Genome-Wide Association Studies. Genetic variants in novel pathways influence blood pressure and cardiovascular disease risk. *Nature* **478**, 103–109 (2011).

[21] Silence, J., Lupu, F., Collen, D. & Lijnen, H. Persistence of atherosclerotic plaque but reduced aneurysm formation in mice with stromelysin-1 (MMP-3) gene inactivation. *Arteriosclerosis, Thrombosis, and Vascular Biology* **21**, 1440–1445 (2001).

[22] Teslovich, T. M. *et al.* Biological, clinical and population relevance of 95 loci for blood lipids. *Nature* **466**, 707–713 (2010).

[23] Aseem, O. *et al.* Cubilin maintains blood levels of HDL and albumin. *Journal of the American Society of Nephrology* **25**, 1028–1036 (2014).

[24] Global Lipids Genetics Consortium. Discovery and refinement of loci associated with lipids levels. *Nature Genetics* **45**, 1274–1283 (2013).

[25] Kennedy, M. A. *et al.* ABCG1 has a critical role in mediating cholesterol efflux to HDL and preventing cellular lipid accumulation. *Cell Metabolism* **1**, 121–131 (2005).

[26] Okada, Y. *et al.* Genetics of rheumatoid arthritis contributes to biology and drug discovery. *Nature* **506**, 376–381 (2014).

[27] Anderson, G. D. *et al.* Selective inhibition of cyclooxygenase (COX)-2 reverses inflammation and expression of COX-2 and interleukin 6 in rat adjuvant arthritis. *Journal of Clinical Investigation* **97**, 2672–2679 (1996).

[28] Kivitz, A., Eisen, G. & Zhao, W. W. Randomized placebo-controlled trial comparing efficacy and safety of valdecoxib with naproxen in patients with osteoarthritis. *Journal of Family Practice* **51**, 530–537 (2002).

[29] Daynes, R. A. & Jones, D. C. Emerging roles of PPARs in inflammation and immunity. *Nature Reviews Immunology* **2**, 748–759 (2002).

[30] Széles, L., Töröcsik, D. & Nagy, L. PPARγ in immunity and inflammation: cell types and diseases. *Biochimica et Biophysica Acta (BBA)-Molecular and Cell Biology of Lipids* **1771**, 1014–1030 (2007).

[31] Su, A. I. *et al.* A gene atlas of the mouse and human protein-encoding transcriptomes. *Proceedings of the National Academy of Sciences of the United States of America* **101**, 6062–6067 (2004).

[32] Duff, M. O. *et al.* Genome-wide identification of zero nucleotide recursive splicing in Drosophila. *Nature* **521**, 376–379 (2015).

[33] Fagerberg, L. *et al.* Analysis of the human tissue-specific expression by genome-wide integration of transcriptomics and antibody-based proteomics. *Molecular & Cellular Proteomics* **13**, 397–406 (2014).

[34] The GTEx Consortium. The genotype-tissue expression (GTEx) pilot analysis: Multitissue gene regulation in humans. *Science* **348**, 648–660 (2015).

[35] Locke, A. E. *et al.* Genetic studies of body mass index yield new insights for obesity biology. *Nature* **518**, 197–206 (2015).

[36] Shungin, D. *et al.* New genetic loci link adipose and insulin biology to body fat distribution. *Nature* **518**, 187–196 (2015).

[37] Schizophrenia Working Group of the Psychiatric Genomics Consortium. Biological insights from 108 schizophrenia-associated genetic loci. *Nature* **511**, 421–427 (2014).

[38] Den Hoed, M. *et al.* Identification of heart rate-associated loci and their effects on cardiac conduction and rhythm disorders. *Nature Genetics* **45**, 621–631 (2013).

[39] Lambert, J.-C. *et al.* Meta-analysis of 74,046 individuals identifies 11 new susceptibility loci for Alzheimer's disease. *Nature Genetics* **45**, 1452–1458 (2013).

[40] Okbay, A. *et al.* Genetic variants associated with subjective well-being, depressive symptoms, and neuroticism identified through genome-wide analyses. *Nature Genetics* **48**, 624–633 (2016).

[41] Morris, A. P. *et al.* Large-scale association analysis provides insights into the genetic architecture and pathophysiology of type 2 diabetes. *Nature Genetics* **44**, 981–990 (2012).

[42] Day, F. R. *et al.* Large-scale genomic analyses link reproductive aging to hypothalamic signaling, breast cancer susceptibility and BRCA1-mediated DNA repair. *Nature Genetics* **47**, 1294–1303 (2015).

[43] Köttgen, A. *et al.* Genome-wide association analyses identify 18 new loci associated with serum urate concentrations. *Nature Genetics* **45**, 145–154 (2013).

[44] Manning, A. K. *et al.* A genome-wide approach accounting for body mass index identifies genetic variants influencing fasting glycemic traits and insulin resistance. *Nature Genetics* **44**, 659–669 (2012).

[45] van Rheenen, W. *et al.* Genome-wide association analyses identify new risk variants and the genetic architecture of amyotrophic lateral sclerosis. *Nature Genetics* **48**, 1043–1048 (2016).

[46] van der Harst, P. *et al.* Seventy-five genetic loci influencing the human red blood cell. *Nature* **492**, 369–375 (2012).

[47] Devlin, B. & Roeder, K. Genomic control for association studies. *Biometrics* **55**, 997–1004 (1999).

[48] Bulik-Sullivan, B. K. *et al.* LD score regression distinguishes confounding from polygenicity in genome-wide association studies. *Nature Genetics* **47**, 291–295 (2015).

[49] Price, A. L. *et al.* Principal components analysis corrects for stratification in genome-wide association studies. *Nature Genetics* **38**, 904–909 (2006).

[50] Wellcome Trust Case Control Consortium . Genome-wide association study of 14,000 cases of seven common diseases and 3,000 shared controls. *Nature* **447**, 661–678 (2007).

[51] Guan, Y. & Stephens, M. Bayesian variable selection regression for genome-wide association studies and other large-scale problems. *The Annals of Applied Statistics* **5**, 1780–1815 (2011).

[52] Zhou, X., Carbonetto, P. & Stephens, M. Polygenic modeling with Bayesian sparse linear mixed models. *PLoS Genetics* **9**, e1003264 (2013).

[53] Wen, X. & Stephens, M. Using linear predictors to impute allele frequencies from summary or pooled genotype data. *The Annals of Applied Statistics* **4**, 1158–1182 (2010).

[54] Lamparter, D., Marbach, D., Rueedi, R., Kutalik, Z. & Bergmann, S. Fast and rigorous computation of gene and pathway scores from SNP-based summary statistics. *PLoS Computational Biology* **12**, e1004714 (2016).

[55] Finucane, H. K. *et al.* Partitioning heritability by functional annotation using genome-wide association summary statistics. *Nature Genetics* **47**, 1228–1235 (2015).

[56] Sachs, M. C. plotROC: A tool for plotting roc curves. *Journal of Statistical Software, Code Snippets* **79**, 1–19 (2017).

[57] 1000 Genomes Project Consortium. A global reference for human genetic variation. *Nature* **526**, 68–74 (2015).

[58] Gao, X., Starmer, J. & Martin, E. R. A multiple testing correction method for genetic association studies using correlated single nucleotide polymorphisms. *Genetic Epidemiology* **32**, 361–369 (2008).

[59] Liu, J. Z. *et al.* A versatile gene-based test for genome-wide association studies. *The American Journal of Human Genetics* **87**, 139–145 (2010).

[60] Li, M.-X., Gui, H.-S., Kwan, J. S. & Sham, P. C. GATES: a rapid and powerful gene-based association test using

extended Simes procedure. *The American Journal of Human Genetics* **88**, 283–293 (2011).

[61] Wang, M. *et al.* COMBAT: A combined association test for genes using summary statistics. *Genetics* **207**, 883–891 (2017).

[62] Voight, B. F. *et al.* The metabochip, a custom genotyping array for genetic studies of metabolic, cardiovascular, and anthropometric traits. *PLoS Genetics* **8**, e1002793 (2012).

[63] Webber, W., Moffat, A. & Zobel, J. A similarity measure for indefinite rankings. *ACM Transactions on Information Systems (TOIS)* **28**, 20 (2010).

[64] Schmich, F. *gespeR: Gene-Specific Phenotype EstimatoR* (2015). URL http://www.cbg.ethz.ch/software/gespeR. R package version 1.8.0.

[65] Cortes, A. & Brown, M. A. Promise and pitfalls of the Immunochip. *Arthritis Research & Therapy* **13**, 101 (2011).

[66] Cerami, E. G. *et al.* Pathway Commons, a web resource for biological pathway data. *Nucleic Acids Research* **39**, D685–D690 (2011).

[67] Geer, L. Y. *et al.* The NCBI BioSystems database. *Nucleic Acids Research* **38**, D492–D496 (2010).

[68] Mi, H. & Thomas, P. PANTHER pathway: an ontology-based pathway database coupled with data analysis tools. *Protein Networks and Pathway Analysis* 123–140 (2009).

[69] Croft, D. *et al.* The Reactome pathway knowledgebase. *Nucleic Acids Research* **42**, D472–D477 (2014).

[70] Schaefer, C. F. *et al.* PID: the Pathway Interaction Database. *Nucleic Acids Research* **37**, D674–D679 (2009).

[71] Romero, P. *et al.* Computational prediction of human metabolic pathways from the complete human genome. *Genome Biology* **6**, R2 (2004).

[72] Hsu, S.-D. *et al.* miRTarBase update 2014: an information resource for experimentally validated mirna-target interactions. *Nucleic Acids Research* **42**, D78–D85 (2014).

[73] Wrzodek, C., Büchel, F., Ruff, M., Dräger, A. & Zell, A. Precise generation of systems biology models from KEGG pathways. *BMC Systems Biology* **7**, 15 (2013).

[74] Caspi, R. *et al.* The MetaCyc database of metabolic pathways and enzymes and the BioCyc collection of Pathway/Genome Databases. *Nucleic Acids Research* **42**, D459–D471 (2014).

[75] Pico, A. R. *et al.* Wikipathways: pathway editing for the people. *PLoS Biology* **6**, e184 (2008).

[76] Turner, S. D. qqman: an R package for visualizing GWAS results using QQ and Manhattan plots. *bioRxiv* 005165 (2014).

[77] Wickham, H. *ggplot2: Elegant Graphics for Data Analysis* (Springer-Verlag New York, 2009). URL http://ggplot2.org.

[78] Stephens, M. False discovery rates: a new deal. *Biostatistics* **18**, 275–294 (2017).

[79] Dey, K. K., Hsiao, C. J. & Stephens, M. Visualizing the structure of RNA-seq expression data using grade of membership models. *PLoS Genetics* **13**, e1006599 (2017).

[80] Lex, A., Gehlenborg, N., Strobelt, H., Vuillemot, R. & Pfister, H. UpSet: Visualization of intersecting sets. *IEEE Transactions on Visualization and Computer Graphics (InfoVis '14)* **20**, 1983–1992 (2014).

[81] Pruim, R. J. *et al.* LocusZoom: regional visualization of genome-wide association scan results. *Bioinformatics* **26**, 2336–2337 (2010).

[82] Yang, R. Y. *et al.* A systematic survey of human tissue-specific gene expression and splicing reveals new opportunities for therapeutic target identification and evaluation. *bioRxiv* (2018).

Xiang Zhu
Department of Statistics
Stanford University
390 Serra Mall
Stanford, California 94305
USA
E-mail: xiangzhu@stanford.edu

Matthew Stephens
Department of Statistics
University of Chicago
5747 S. Ellis Avenue
and
Department of Human Genetics
University of Chicago
920 E. 58th Street
Chicago, Illinois 60637
USA
E-mail: mstephens@uchicago.edu
